# Computational Prediction of One-Step Synthesis of Seven-membered Fused Rings by (5+2) Cycloaddition Utilising Cycloalkenes

**DOI:** 10.1038/srep12272

**Published:** 2015-07-22

**Authors:** Chen-Chen Zhou, Xiao-Na Ke, Xiu-Fang Xu

**Affiliations:** 1 Department of Chemistry, Key Laboratory of Advanced Energy Materials Chemistry (Ministry of Education), Nankai University, Tianjin, 300071, P. R. China

## Abstract

The (5+2) cycloaddition reaction utilising cycloalkenes is rare, although it is one of the most efficient methods of constructing seven-membered fused rings because of its high atom- and step-economy. In this study, we used quantum mechanical calculations to predict the plausibility of using the Rh-catalysed intermolecular (5+2) cycloaddition of 3-acyloxy-1,4-enynes and cycloalkenes to produce fused seven-membered carbocycles. The calculation results suggest a convenient, highly efficient and energetically practical approach. Strained cycloalkenes, such as cyclopropene, have been predicted to be active, and the desired bicyclic product should be favoured, accompanied by the formation of byproducts from rearrangement reactions. The energy barriers of the alkene insertion step were analysed by the distortion/interaction model to disclose the origins of the different reactivities of cycloalkenes with different ring sizes.

The construction of seven-membered fused rings has been an important issue in the synthesis of natural products and the development of new medicines^1^. Generally, these bicyclic compounds can be constructed through ring closure of monocyclic compounds^2^,^3^, rearrangement reactions^4^,^5^,^6^,^7^, intramolecular cycloaddition of five-carbon synthons bearing a tethered alkyne^8^,^9^,^10^,^11^,^12^ or modification of previously formed seven-membered rings^13^. However, these methods typically require complicated reactants with subtly positioned functional groups, a substantial amount of organic solvents, and a lengthy course or several synthesis and separation steps. Recently, a number of pericyclic (5+2) cycloadditions have been reported^14^, which provide the possibility of synthesising seven-membered fused ring compounds through the one-step intermolecular (5+2) cycloadditions of a five-carbon synthon and cycloalkenes.

Detailed DFT studies from Houk, Wender and co-workers have revealed that the catalytic cycle of the Rh-catalysed (5+2) reaction of vinylcyclopropane (VCP) with alkynes involves initial cyclopropane ring opening, alkyne insertion and reductive elimination, in which alkyne insertion is found to be not only rate determining but also regioselectivity determining^15^,^16^,^17^,^18^,^19^. More recently, we have investigated the mechanism of the Rh-catalysed (5+2) reaction of 3-acyloxy-1,4-enynes (ACE) with alkynes (Fig. 1a)^20^, suggesting that this reaction proceeds via three steps, i.e., acyloxy migration, alkyne insertion (2π insertion) and reductive elimination. Therein, the acyloxy migration is rate determining, and the alkyne insertion is regioselectivity determining. Interestingly, alkyne prefers to insert into a rhodium−allyl bond in the VCP-based (5+2) cycloadditions, but prefers to insert into a Rh−C(sp^2^) bond in the ACE-based (5+2) cycloadditions. This difference of mechanism results in not only different regiochemical control but also unique substituent effects on reactivity, when employing ACE instead of VCP as the 5-C synthon. In this study, we attempt to extend the scope of the two-carbon synthon to cycloalkenes, aiming to develop the most atom- and step-economical method to synthesise a fused bicyclic carbon skeleton including a seven-membered ring (Fig. 1b). Significantly, our computational results indicate that the rate-determining step for (5+2) cycloaddition between ACE and cycloalkene varies with the cycloalkene substrate.

ACE is selected as the model five-carbon synthon because its cycloaddition products possess two C = C double bonds that could be selectively functionalised. Density functional theory (DFT) methods are employed to compute this catalytic system because they better balance the computational costs and chemical accuracy^21^. The most efficient catalyst for alkynes, Rh(PPh_3_)_3_Cl, is used as the model catalyst, which releases two phosphine ligands and binds to ACE to initiate the reaction^20^.

The highly strained cyclopropene structure can be used as an excellent three-carbon component to construct six- or five-membered ring skeletons in the Rh(I)-catalyzed [3+2+1] or [3+2] cycloadditions of ene- and yne-cyclopropene systems^22^,^23^,^24^. Wender and coworkers firstly employed vinylcyclopropene as a five-carbon component in the Rh(I)-catalyzed intermolecular [5+2+1] carbocyclization^25^. Small cycloalkenes, namely cyclopropene^26^,^27^, cyclobutene and their derivatives^28^, have been reported to show a high reactivity in Diels-Alder reactions. Recent studies from the Houk group^28^,^29^ have accounted for this unusual high reactivity of strained cycloalkenes by a distortion/interaction model^30^. Because of the strain, the cycloalkene is pre-distorted to reduce the distortion energy, therefore lowering the overall activation energy. Both experimental^10^,^11^,^31^ and theoretical^20^ studies have suggested that Rh-catalysed (5+2) reaction of ACEs with alkynes proceeds via sequential steps of acyloxy migration, alkyne insertion (2π insertion) and reductive elimination. Because the 2π insertion step in the (5+2) cycloadditions is a bimolecular pericyclic process, similar to the Diels-Alder reaction, we expect that the cycloalkenes will also act as potential two-carbon synthons for Rh-catalysed (5+2) cycloaddition with ACE. Therefore, we envisioned that the designed (5+2) cycloadditions of ACE and cycloalkenes might take place via the same three steps, as the (5+2) cycloadditions of ACE and alkynes do. It is noteworthy that the two-carbon synthon is not involved in the initial acyloxy migration but in the following 2π insertion and reductive elimination two steps (Fig. 1a). As a result, if the cycloalkene substrates have similar reactivities to the alkynes in the latter two steps, the reaction should be plausible, at least theoretically. Cycloalkenes with different ring sizes are employed in the calculations as well as open-chain alkenes for comparison (Fig. 1b).

## Results and Discussion

**Figure 2:**
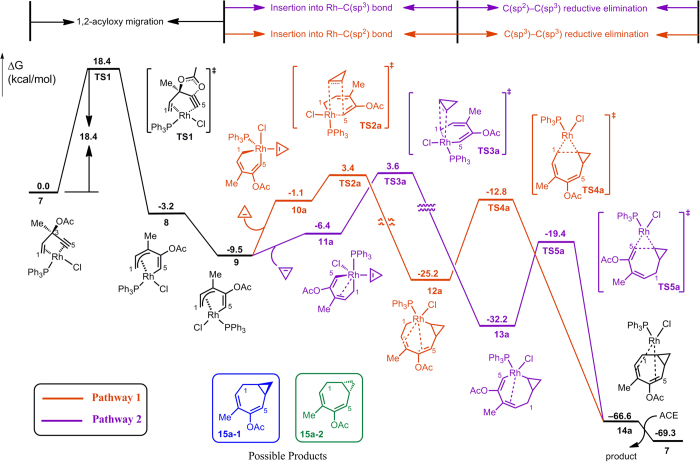
Two of the representative pathways of Rh-catalysed (5+2) cycloadditions of ACE and cyclopropene.

Two of the typical pathways of cycloaddition with cyclopropene are computed and presented in Fig. 2. The reaction is initiated at the ACE-Rh(PPh_3_)Cl π complex **7**^20^. The acyloxy group migrates from C3 to C4 through a five-membered ring transition state (**TS1**) with a barrier of 18.4 kcal/mol. The resulting complex **8** can convert into a more stable isomer **9** by exchanging the two ligands Cl and PPh_3_. The cycloalkene substrate could then be inserted into the metallacycle from either the Rh-C5(sp^2^) bond (pathway 1) or the Rh-C1(sp^3^) bond (pathway 2). The intermediates **12a** and **13a** from either of the 2π insertion pathways will undergo reductive elimination to generate the product complex **14a**, which then releases the product while rebinding to ACE, thus completing a catalytic cycle.

**Figure 3:**
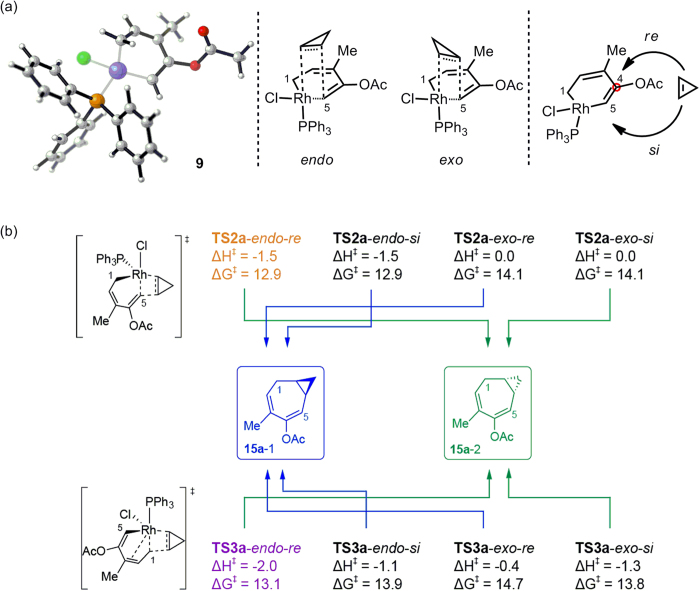
(**a**) The 3D model of intermediate 9 and the possible patterns for cyclopropene insertion. (**b**) Energy barriers for the various cyclopropene insertion transition states. Energy barriers are calculated relative to intermediate 9 in kcal/mol. The transition states TS2a and TS3a presented in Fig. 1 are highlighted accordingly (in orange and in purple). Blue and Green arrows indicate to which enantiomer the transition state will lead.

**Figure 4:**
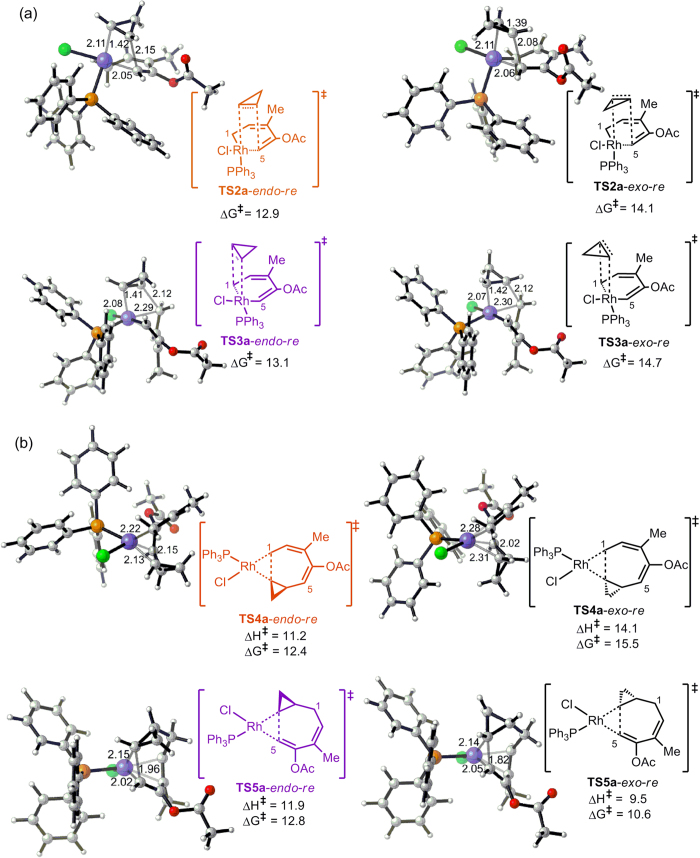
Geometries of the transition states for the cyclopropene insertion and reductive elimination steps. (**a**) Geometries of four possible transition states for the *re* cyclopropene insertion. **TS2a**-*endo*-*re* and **TS3a**-*endo*-*re* are the two transition states **TS2a** and **TS3a** shown in Fig. 1. Energy barriers are in kcal/mol and calculated relative to intermediate **9.** The four possible transition states for the *si* cyclopropene insertion are given in Figure S1 of the Supplementary Information. (**b**) Geometries of four reductive elimination transition states. **TS4a**-*endo*-*re* and **TS5a**-*endo*-*re* are the two transition states **TS4a** and **TS5a** shown in Fig. 1. Activation enthalpies and Gibbs free energies are in kcal/mol and calculated relative to the corresponding metallacycle intermediate **12a** and **13a**, respectively.

The conformation of the alkene insertion (2π insertion) step determines the chirality of the products. Intermediate **9** is a slightly twisted metallacycle. The cycloalkene could approach metal complex **9** in an *endo* or *exo* orientation to the metallacycle ring (Fig. 3a). The cycloalkene could also bind to the rhodium centre from both sides of the ring plane. To distinguish the two sides of the metallacycle, the conventional nomenclature of the pro-chirality of sp^2^ carbons is applied to this system, and the two different orientations are named *re* and *si* according to the pro-chirality of C4 of ACE (Fig. 3a). All of the orientations of the cyclopropene insertion were considered. The corresponding energy barriers are listed in Fig. 3b, and the transition states for the *re* insertion patterns are shown in Fig. 4a. Noticeably, these approaches with different orientations will lead to fused ring products with different chirality (**15a-1** and **15a-2** in Fig. 3b).

It was found that when cyclopropene inserts into the Rh-C5(sp^2^) bond (**TS2a**), the rhodium metallacycle in **TS2a** is generally planar, causing the *re* and *si* orientations to be more chemically and energetically similar, while the *endo* and *exo* orientations show a larger difference in energy. Generally, the *endo* transition states are lower in energy than the *exo* transition states, which is consistent with the previously reported results^32^,^33^. The energy difference between the *endo*- and *exo*-TSs is mainly caused by two factors. An examination on the geometries of the *endo* and *exo* transition states of the cyclopropene insertion reveals that, in the transition state **TS2a***-endo-re*, the methylene hydrogen of cyclopropene is positioned toward the C2 = C3 double bond (Fig. 4a), which will result in the CH-π interaction between the methylene hydrogen of cyclopropene and the C2 = C3 double bond. However, in the transition state **TS2a***-exo-re*, the methylene hydrogen of cyclopropene is positioned backward the five-carbon skeleton of ACE moiety (Fig. 4a). As a result, no CH-π interaction could be observed in **TS2a***-exo-re*. Therefore, the CH-π interaction between the methylene hydrogen of cyclopropene and the C2 = C3 double bond may be one of the main factors contributing to the lower energy of the *endo* transition state of the cyclopropene insertion relative to that of the *exo* transition state. Moreover, the consistent results obtained by the M06 and ONIOM methods^34^,^35^,^36^,^37^ confirmed that the stronger steric repulsion between the bulky PPh_3_ ligand and the cyclopropene in *exo*-TSs than in *endo*-TSs is the other factor contributing to the energy difference between *endo*- and *exo*-TSs (for details, see Table S1 in the Supporting Information). For the insertion of cyclopropene into the Rh-C1(sp^3^) bond (**TS3a**), the rhodium metallacycle is relatively more twisted because C1 must be sp^3^ hybridised to bind with the cyclopropene substrate. In that case, the two faces of the metallacycle are more chemically different and possess less orbital overlap with the cyclopropene substrate. This trend is clearly observed in the calculated energy barriers, as shown in Fig. 3b in which in **TS2a**, the *re*/*si* insertion barriers are identical, while in **TS3a**, the *re*/*si* insertion barriers are different.

All of the calculated cyclopropene insertion barriers (12.9–14.7 kcal/mol, Fig. 3b) are lower than that of the 1,2-acyloxy migration, regardless of the approach of cyclopropene to the rhodium complex. This result indicates that the cyclopropene insertion step should not be rate determining. However, the differences between the barriers of the favoured 2π insertion patterns (for example, **TS2a**-*endo*-*re* and **TS2a**-*endo*-*si*) are small because the *re* and *si* orientations are nearly mirror images. Thus, the reaction is not likely to be stereo-selective. However, it is expected that the introduction of chiral factors, such as chiral ligands or asymmetrically substituted cycloalkenes, to the reaction system will lead to stereo-selectivity.

For clarity, the following discussions will only consider pathways involved with the *re* patterns of the 2π insertion. The expanded eight-membered metallacycle intermediates **12a** and **13a** then undergo reductive elimination to generate the product complex **14a**. As shown in Fig. 2, **TS4a** and **TS5a** are the two reductive elimination transition states in the two different pathways. Generally, **TS4a** is higher in energy than **TS5a** because in **TS4a,** a C(sp^3^)-C(sp^3^) bond is formed, while in **TS5a,** a C(sp^2^)-C(sp^3^) bond is formed. The reductive elimination energy barriers are also generally low (10.6–15.5 kcal/mol, Fig. 4b); therefore, the rate-determining step is acyloxy migration for the (5+2) reaction with cyclopropene.

For the product complex **14a**, ACE binds with rhodium, releasing the product, thus, completing a catalytic cycle. The exchange energy of ACE and of the product is negative (−2.7 kcal/mol), which suggests that the regeneration of Rh-ACE complex **7** is favoured. The exchange energy is noteworthy because if the product complex is more stable while the first step is rate determining, then the overall activation barrier will be elevated according to the energy span model^38^. Negative exchange energy ensures that the catalytic cycle would proceed easily.

Due to the extreme strain of the cyclopropene ring, the C-C bond of the three-membered ring is less stable than open-chain alkenes, and the induction of possible rearrangement reactions is discussed in the supplementary information (Figures S2 and S3). The overall energy profile shows that these rearrangements are less favoured and should not affect the generation of the seven-membered fused ring product.

Typically, synthesized cyclopropenes have at least two substituents^27^,^39^,^40^. The presence of these substituents facilitates the use of these cycles and the synthesis. In this study, we computed the activation barrier of the cyclopropene insertion step for a substituted cyclopropene **a′**. The results are listed in entry a′ of Table 1. As expected, the activation barrier for cyclopropene insertion of the substituted cyclopropene **a′** increased to 21.0 kcal/ml (the 2π insertion step). This result should be primarily attributed to steric repulsion between substituents on cyclopropene and the bulky PPh_3_ ligand (see Figure S4 in the Supporting Information). Because an activation barrier of 21.0 kcal/mol is not high, the reaction with the substituted cyclopropene is predicted to occur using a higher temperature.

Energy profiles of the reactions with cycloalkenes of different sizes were calculated, and the most favoured pathways are listed in Table 1. From the calculated energies (Table 1), it can be observed that in most cases, pathway 1 is more favoured in the 2π insertion step, while pathway 2 is more favoured in reductive elimination. As the size of the cycloalkene ring increases, the energy barrier of the 2π insertion step also increases. The 2π insertion step is obviously influenced by the ring size of the cycloalkenes, while this trend is not observed for reductive elimination.

**Figure 5:**
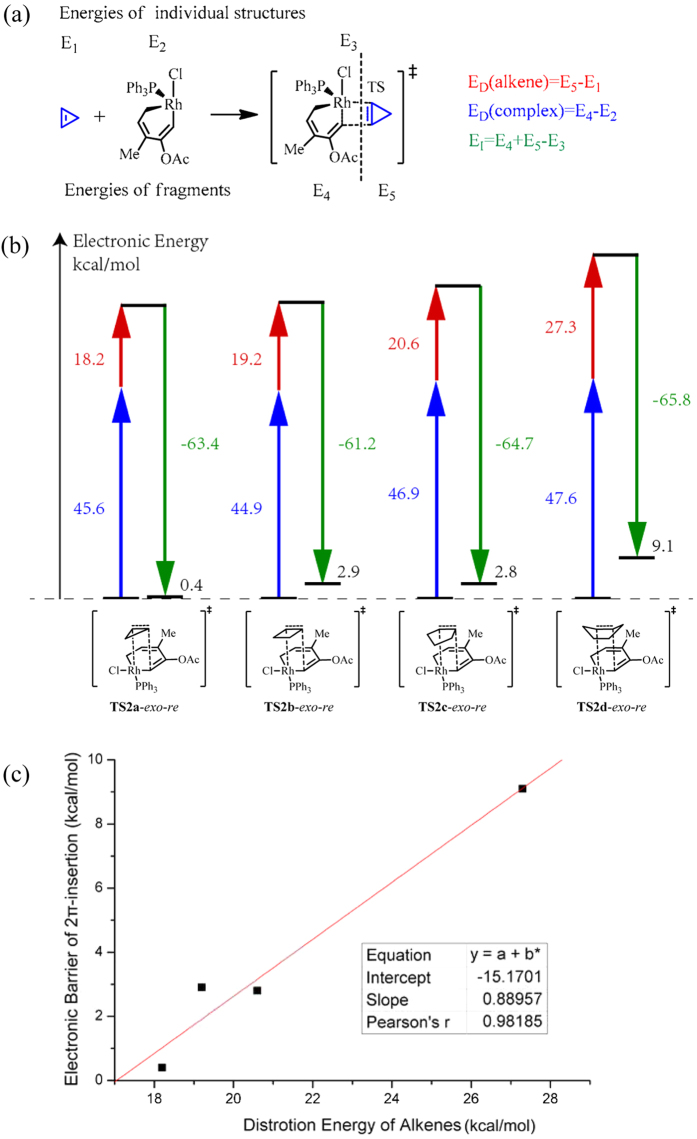
Distortion/Interaction (D/I) analysis of the 2π insertion step for different substrates. (**a**) The energy partitioning scheme for the D/I analysis. (**b**) The distortion energies and interaction energies. Red arrows indicate the distortion energies of alkenes, blue arrows indicate the distortion energies of the metallacycle fragments, and green arrows indicate the interaction energies. The overall electronic energies of activation are shown in black. Energy values are given in kcal/mol. (**c**) Linear fitting between electronic energies of activation and the corresponding distortion energies of the cycloalkenes for the 2π insertion step.

As the substrate changes, the rate-determining step also varies. Table 1 indicates that the rate-determining step is sensitive to the 2-carbon synthon. This result is primarily attributed to a combination of three factors: the steric repulsion between two substrates as well as that between substrates and the bulky PPh_3_ ligand, the distortion energy for the 2-carbon synthon to achieve the transition-state geometry, and the electronic effect of the 2-carbon synthon. Steric repulsion becomes stronger as the size of the 2-carbon synthon increases, thus leading to higher energy barriers of the 2π insertion and the reductive elimination steps. Therefore, the transition states of the 2π insertion or reductive elimination reach the highest point of the energy profile and become the rate-determining step. For example, the rate-determining step for the reaction with cyclobutene is the 2π insertion and that for cyclopentene is reductive elimination, while that for cyclopropene is acyloxy migration (entries a, b and c). Similarly, introduction of substituents to the 2-carbon synthon will also increase the activation barrier of the 2π insertion step due to increased steric repulsion. For example, the activation barrier of the 2π insertion for substituted cyclopropene **a′** is 21.0 kcal/mol (entry a′), which is 2.6 kcal/mol higher than that of the 1,2-acyloxy migration; therefore, the 2π insertion is the rate-determining step for this substituted cyclopropene. As illustrated in Fig. 5, the distortion energies required for cycloalkenes to achieve the transition-state geometries of the 2π insertion increase as the size of the cycloalkenes increases, leading to an increase of the activation barriers of the 2π insertion step. For example, the transition state of the 2π insertion for cyclohexene has the highest free energy; therefore, it is the rate-determining step. Generally, because alkene substrates are less electron-rich than alkyne substrates, the 2π insertion of an alkene requires a higher activation energy than that required for an alkyne. For example, the rate-determining step is the 2π insertion with an activation barrier of 20.5 kcal/mol for ethylene, (entry e). The activation barrier of the 2π insertion for acetylene is 14.2 kcal/mol, which is 4.2 kcal/mol lower than that of the 1,2-acyloxy migration (entry g). Therefore, the 1,2-acyloxy migration is the rate-determining step for acetylene.

The overall activation energy increases with the size of the cycloalkene ring. According to the overall activation free energies (the highlighted ΔG^‡^) listed in Table 1, cyclopropene should be as reactive as acetylene in this reaction, while cyclobutene may be as reactive as ethylene. Cyclopentene and cyclohexene are less reactive because of their larger ring size, similar to the open chain *cis*-2-butene. Therefore, our calculation results suggest that intermolecular (5+2) cycloaddition of ACE with cyclopropene should be plausible because acetylene and substituted acetylenes are good reactants for this type of reaction^10^. Reactions with cyclobutene might proceed by heating the reaction system to a higher temperature, while it will be more difficult for reactions with cyclopentene or cyclohexene to proceed under moderate conditions.

As discussed above, the 2π insertion step is obviously influenced by the ring size of the cycloalkenes. In this study, the distortion/interaction (D/I) model^30^ is applied to the 2π insertion step according to the following partitioning scheme (Fig. 5a) to investigate the origins of the different reactivities displayed by the substrates. To compare the distortion energies of the rhodium complex fragments (E_D_(complex)), the same geometry (**TS2x**-*exo*-*re*, **x** = **a**, **b**, **c** and **d**) of the transition states for the 2π insertion are adopted for different substrates in the analysis.

From the D/I analysis (Fig. 5b), we can observe that the activation barrier is significantly related to the distortion energy of the cycloalkene substrate (E_D_(alkene)). The variations in distortion energy of the metallacycle (E_D_(complex)) and the interaction energy (E_I_) are relatively small for different substrates. The linear fitting (Fig. 5c) of the electronic energies of activation and the distortion energies of the alkenes has a correlation coefficient of 0.98, also proving that the two quantities are related. The energies required for cycloalkenes to be distorted into the 2π insertion transition states increase as the ring size of the cycloalkenes increases from cyclopropene to cyclohexene, resulting in an increase in the activation barriers. This correlation is the same as that observed in the Diels-Alder cycloaddition^29^, and we suppose it may also occur in other pericyclic reactions.

## Conclusion

In this study, we explored the plausibility of the Rh-catalysed (5+2) cycloaddition reaction between ACE and cycloalkenes. The more strained cycloalkenes are predicted to be more reactive in this reaction, while those with a larger ring are less reactive. Cyclopropene is suggested to be as reactive as acetylene and is a potential reagent; cyclobutene is less reactive, but the reaction may be practical at a higher temperature. The rate-determining step varies with different cycloalkenes primarily because of the change of the energy barrier of the 2π insertion step. The distortion/interaction model was introduced to disclose the origins of the different reactivities of various cycloalkenes. The distortion of the alkene was found to be the primary factor influencing the reactivity. The reaction follows the mechanism of the (5+2) cycloaddition between ACE and alkynes and is stereochemically complicated in the cycloalkene insertion step. It is expected that stereo-selectivities might be realised by introducing chiral factors to the reaction systems.

## Methods

All of the calculations were performed using the Gaussian 09 software package. Geometry optimisations were performed with the B3LYP^41^,^42^,^43^ functional. The Stuttgart/Dresden effective core potential^44^ was used on rhodium, and the 6–31 G(d) basis set was employed for other atoms. We considered all of the possible isomers by rotating the bulky PPh_3_ group around the Rh-P bond and rotating the acyloxy group around the C3-O bond for the conformation search and, finally, screening out the most stable isomer for each species. The isomers with the lowest energy are presented. Frequency calculations were performed at 298.15 K to verify that the stationary points were the local minima or first-order saddle points, i.e., transition states, and to obtain thermal corrections. The harmonic oscillator approximation engaged in vibrational frequency calculations were replaced by Truhlar’s quasi-harmonic approximation^45^. Intrinsic Reaction Coordinate (IRC) calculations were performed to determine the connectivity of the minima and transition states. Single point energy calculations were performed with the M06 functional^46^, a mixed basis set of SDD for rhodium and 6–311+G(d, p) for other atoms, and the SMD^47^ solvation model with chloroform as the solvent (ε = 4.7113). Enthalpy and Gibbs free energy values are the sum of the electronic energy from the single point calculations and the thermal corrections obtained by the frequency calculations.

Because the molecular systems in this study are relatively large, dispersion effects may be important. To address this issue, we selected entry d as the test case and performed the DFT-D3 correction^48^,^49^ with M062X functional^50^. The results are listed in Table S4 of the Supporting Information. The results confirm that our conclusions are reliable based on the M06/SDD-6-311+G(d, p)/SMD(CHCl_3_)//B3LYP/SDD-6-31 G(d) computed energies. Thus, we did not apply the DFT-D3 correction for the remaining entries.

## Additional Information

**How to cite this article**: Zhou, C.-C. *et al.* Computational Prediction of One-Step Synthesis of Seven-membered Fused Rings by (5+2) Cycloaddition Utilising Cycloalkenes. *Sci. Rep.* **5**, 12272; doi: 10.1038/srep12272 (2015).

## Supplementary Material

Supplementary Information

## Figures and Tables

**Figure 1:**
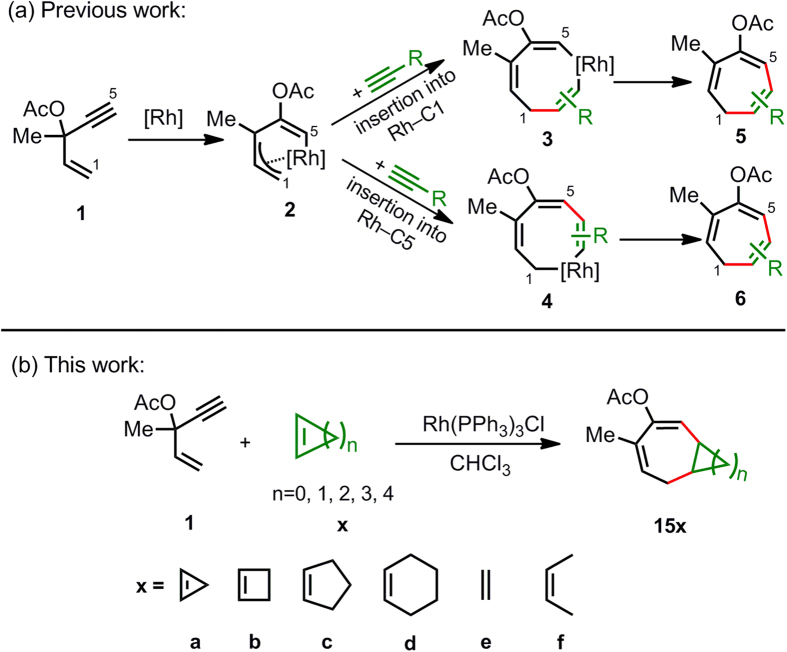
Proposed mechanisms. (**a**) Rh-catalysed (5+2) cycloadditions of 3-acyloxy-1,4-enyne (ACE) and alkynes. (**b**) Rh-catalysed (5+2) cycloadditions of 3-acyloxy-1,4-enyne (ACE) and alkenes.

**Table 1:**
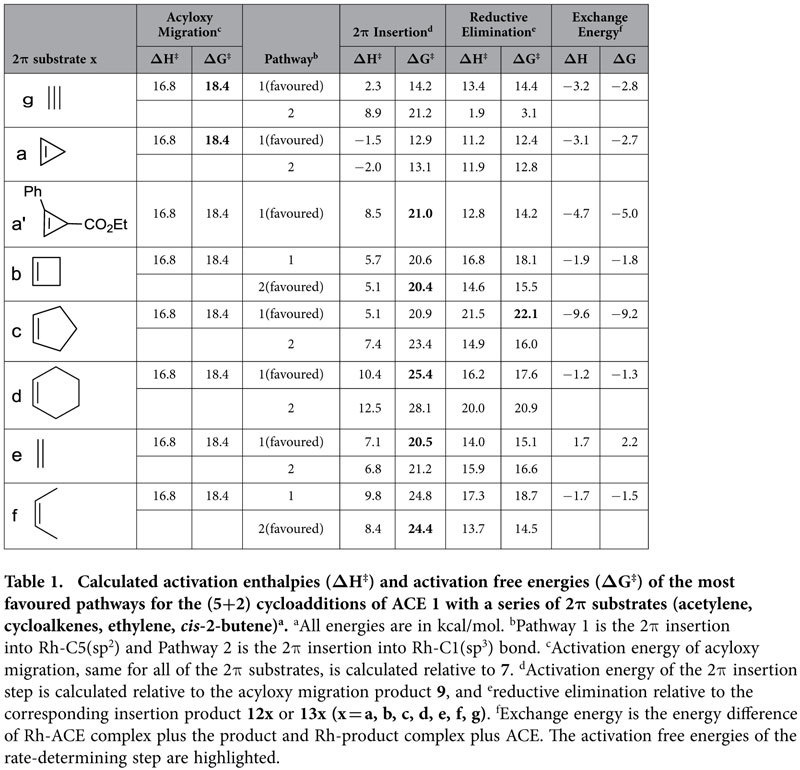
Calculated activation enthalpies (ΔH^‡^) and activation free energies (ΔG^‡^) of the most favoured pathways for the (5+2) cycloadditions of ACE 1 with a series of 2π substrates (acetylene, cycloalkenes, ethylene, *cis*-2-butene)^a^.
