# Supplementary material for: Computational Prediction of One-Step Synthesis of Seven-membered Fused Rings by (5+2) Cycloaddition Utilising Cycloalkenes
Source: Sci Rep. 2015 Jul 22;5:12272. doi: 10.1038/srep12272 (PMC4510518; doi:10.1038/srep12272)
Supplement: Supplementary Information [file srep12272-s1.doc]

Supplementary information

**Computational Prediction of One-Step Synthesis of Seven-membered Fused Ring by (5+2) Cycloaddition Utilizing Cycloalkenes**

Chen-Chen Zhou, Xiao-Na Ke & Xiu-Fang Xu*

Department of Chemistry, Key Laboratory of Advanced Energy Materials Chemistry (Ministry of Education), Nankai University, Tianjin, 300071, P. R. China

*e-mail: xxfang@nankai.edu.cn

Contents

**Figure S1:** 3D structures and Gibbs free energies of activation of the transition states for cyclopropene insertion from the *si* face of the metallacycle S2

**Figures S2 and S3:** The possible rearrangement pathways for reaction with cyclopropene S2-S3

**Figures S4:** 3D structures of the transition states for cyclopropene insertion of **a** and **a׳**………………………….S4

**Table S1.** The ONIOM(M06/SDD-6-311+G(d,p)/SMD(CHCl_3_): UFF) and the M06/SDD-6-311+G(d,p)/ SMD(CHCl_3_) computed single point energies on the B3LYP/6-31G(d) optimized geometries for four transition states………………………………………………………………………………………………………………..S4

[**Table S2:** The M06/SDD-6-311+G(d,p)/SMD(CHCl_3_)//B3LYP/SDD-6-31G(d) computed relative enthalpies, free energies of the 2π insertion and reductive elimination transition states in both *endo* and *exo* geometries for substrates beside cyclopropene (**a**).………………………………………………………………………..………..S5](#__RefHeading___Toc385845938)

[**Table S3.** 3D structures of transition states as well as Gibbs free energies of activation for the rate-determining steps of reactions with different substrates. S6](#__RefHeading___Toc385845939)

**Table S4.** M06/SDD-6-311+G(d,p)/SMD(CHCl_3_)//B3LYP/SDD-6-31G(d) and M062X-D3-corrected activation free energies (ΔG^‡^) of the sequential steps for (5 + 2) cycloaddition of cyclohexene (**d**) and ACE **1**……………..S8

[**Table S5:** The B3LYP/SDD-6-31G(d) computed energies, enthalpies, free energies of all stationary points discussed in the text. S9](#__RefHeading___Toc385845940)

[**Table S6:** The M06/SDD-6-311+G(d,p)/SMD(CHCl_3_)//B3LYP/SDD-6-31G(d) computed energies, enthalpies, free energies of all stationary points discussed in the text. S12](#__RefHeading___Toc385845941)

[**The Cartesian Coordinates of the stationary points discussed in the text** S16](#__RefHeading___Toc385845942)

# Figure S1. 3D structures and Gibbs free energies of activation of the transition states for cyclopropene insertion from the *si* face of the metallacycle. Gibbs free energies of activation are in kcal/mol and calculated relative to 9 in Figure 2.

# The possible rearrangement pathways for reaction with cyclopropene:

Due to the extreme strain of cyclopropene ring, the C-C bond of the three-membered ring is less stable than open-chain alkenes. Therefore rearrangement reactions need to be considered. Such possibilities were observed in the Intrinsic Reaction Coordinates (IRC) calculations, where the intermediate generated from **TS5a**-*exo*-*re* is not the expected product complex **14a**, but a rearrangement isomer **16** shown in Figure S2.

**Figure S2.** Additional reductive elimination step, 3D model of **16** and activation barrier in kcal/mol.

The rhodium atom has unexpectedly inserted into the C-C bond of the cyclopropene ring in the process of reductive elimination, as shown in Figure S2. In the geometry of **TS5a**-*exo*-*re*, the C-C bond of the three-membered ring to be cleaved is positioned rather close to the metal center as can be seen in Figure 4b of the text, leading the Rh atom to insert into the C-C bond to maintain its 16-electron configuration and form the intermediate **16**. Such complex can undergo reductive elimination again to generate the expected product with a considerably low barrier, 9.8 kcal/mol. So the expected product can still be obtained through this pathway.

**Figure S3.** Possible rearrangement pathways from **12a**. Energy barriers in kcal/mol relative to **7** in Figure 2 of the text. Geometries are optimized with B3LYP/SDD-6-31G(d) and single point energies are calculated with M06/ SDD-6-311+G(d,p)/SMD(chloroform).

We have made additional effort to investigate the possible rearrangement reactions. The intermediate **12a** (Fig. 2 of the text) possesses a properly laid conjugate system where the π electrons will readily redistribute, while **13a** (Fig 2 of the text) is less likely to rearrange. Two possible rearrangement pathways are computed for **12a** (Fig. S3). These pathways are energetically disfavored with higher activation barriers (**TS7a**, 19.8 kcal/mol; **TS10a**, 39.0 kcal/mol) compared with those of the reductive elimination (**TS4a,** 12.4 kcal/mol; **TS5a**, 12.8 kcal/mol, as shown in Fig. 2 of the text) which lead to the fused bicyclic product.

ΔG^‡^ = 12.9 kcal/mol ΔG^‡^ = 21.0 kcal/mol

**TS2a***-endo-re* **TS2a**׳*-endo-re*

**Figure S4.** 3D structures of the transition states for cyclopropene insertion of **a** and **a׳**.

**Table S1.** The ONIOM(M06/SDD-6-311+G(d,p)/SMD(CHCl_3_): UFF) and the M06/SDD-6-311+G(d,p)/SMD (CHCl_3_) computed single point energies on the B3LYP/6-31G(d) optimized geometries for four transition states.

| species | ONIOM(M06/SDD-6-311+G(d,p) /SMD(CHCl_3_): UFF) |  | M06/SDD-6-311+G(d,p)/SMD(CHCl_3_) |
| --- | --- | --- | --- |
|  | single point energy (hartree) |  | single point energy (hartree) |
| **TS2a***-endo-re* | -1837.399537 |  | -2184.446465 |
| **TS2a***-endo-si* | -1837.399534 |  | -2184.446463 |
| **TS2a***-exo-re* | -1837.284758 |  | -2184.443806 |
| **TS2a**-*exo-si* | -1837.284777 |  | -2184.443807 |

As well known, both electronic and steric effects are included in the total energy of a species with DFT method. To quantify electronic and steric effects, we performed ONIOM(M06/SDD-6-311+G(d,p)/SMD(CHCl_3_): UFF) single-point energy calculations on the B3LYP/6-31G(d) optimized geometries for four transition states (as listed in Table S1), where the bulky PPh_3_ group was added in the MM. In this way, electronic contribution of the bulky group can be eliminated. The computational results indicate that the M06 and ONIOM calculated energies are consistent: the *endo* transition states are lower in energy than the *exo* transition states, regardless of the computational methods. Therefore, the stronger steric repulsion between the bulky PPh_3_ ligand and the cyclopropene in *exo*-TSs than in *endo*-TSs should play an important role for the energy difference between the *exo*- and *endo*-TSs.

# Table S2: The M06/SDD-6-311+G(d,p)/SMD(CHCl3)//B3LYP/SDD-6-31G(d) computed relative enthalpies, free energies of the 2π insertion and reductive elimination transition states in both *endo* and *exo* geometries for substrates beside cyclopropene (a). Energies calculated with respect to the corresponding intermediate.

| pathway | TS | ΔH^‡^ | ΔG^‡^ | TS | ΔH^‡^ | ΔG^‡^ |
| --- | --- | --- | --- | --- | --- | --- |
| b-pathway1-*endo* | **TS2b***-endo-re* | 5.7 | 20.6 | **TS4b***-endo-re* | 16.8 | 18.1 |
| b-pathway1-*exo* | **TS2b-***exo-re* | 3.4 | 18.0 | **TS4b***-exo-re* | 24.3 | 25.0 |
| b-pathway2-*endo* | **TS3b-***endo-re* | 5.1 | 20.4 | **TS5b***-endo-re* | 14.6 | 15.5 |
| b-pathway2-*exo* | **TS3b***-exo-re* | 7.0 | 22.3 | **TS5b***-exo-re* | 12.9 | 13.7 |
| c-pathway1-*endo* | **TS2c***-endo-re* | 5.1 | 20.9 | **TS4c***-endo-re* | 21.5 | 22.1 |
| c-pathway1-*exo* | **TS2c***-exo-re* | 3.6 | 19.2 | **TS4c**-*exo-re* | 27.2 | 27.9 |
| c-pathway2-*endo* | **TS3c***-endo-re* | 7.4 | 23.4 | **TS5c***-endo-re* | 14.9 | 16.0 |
| c-pathway2-*exo* | **TS3c***-exo-re* | 11.2 | 27.0 | **TS5c***-exo-re* | 12.6 | 13.7 |
| d-pathway1-*endo* | **TS2d***-endo-re* | 17.1 | 32.0 | **TS4d***-endo-re* | 13.0 | 14.3 |
| d-pathway1-*exo* | **TS2d***-exo-re* | 10.4 | 25.4 | **TS4d***-exo-re* | 16.2 | 17.6 |
| d-pathway2-*endo* | **TS3d***-endo-re* | 12.5 | 28.1 | **TS5d***-endo-re* | 20.0 | 20.9 |
| d-pathway2-*exo* | **TS3d***-exo-re* | 15.5 | 31.3 | **TS5d***-exo-re* | 18.4 | 19.4 |
| f-pathway1-*endo* | **TS2f***-endo-re* | 11.6 | 26.7 | **TS4f***-endo-re* | 13.7 | 14.9 |
| f-pathway1-*exo* | **TS2f***-exo-re* | 9.8 | 24.8 | **TS4f***-exo-re* | 17.3 | 18.7 |
| f-pathway2-*endo* | **TS3f***-endo-re* | 8.4 | 24.4 | **TS5f***-endo-re* | 13.7 | 14.5 |
| f-pathway2-*exo* | **TS3f***-exo-re* | 11.8 | 27.5 | **TS5f***-exo-re* | 16.3 | 17.4 |

Reaction with ethylene (**e**) or acetylene (**g**) does not have the *endo/exo* issue and thus not listed in the table.

# Table S3. 3D structures of transition states as well as Gibbs free energies of activation for the rate-determining steps of reactions with different substrates. Gibbs free energies of activation are calculated with respect to the corresponding intermediate in kcal/mol.

| Substrate | TS of the rate-determining step |
| --- | --- |
|  |  |
|  |
|  |  |
|  |  |
|  |  |
|  |  |
|  |  |

**Table S4.** M06/SDD-6-311+G(d,p)/ SMD(CHCl_3_)// B3LYP/SDD-6-31G(d) and M062X-D3-corrected activation free energies (ΔG^‡^) of the sequential steps for (5 + 2) cycloaddition of cyclohexene (**d**) and ACE **1**.

| step |  | M06/SDD-6-311+G(d,p)/ SMD(CHCl_3_)// B3LYP/SDD-6-31G(d) |  | M062X-D3 corrected M06/SDD-6-311+G(d,p)/ SMD(CHCl_3_)//B3LYP/SDD-6-31G(d) |
| --- | --- | --- | --- | --- |
|  |  | ΔG^‡^ (kcal/mol) |  | ΔG^‡^ (kcal/mol) |
| Acyloxy migration^a^ |  | 18.4 |  | 18.5 |
| 2π Insertion^b^ | Pathway 1 | 25.4 |  | 23.4 |
|  | Pathway 2 | 28.1 |  | 26.2 |
| Reductive Elimination^c^ | Pathway 1 | 17.6 |  | 17.5 |
|  | Pathway 2 | 20.9 |  | 21.1 |

^a^Activation energy of acyloxy migration is calculated relative to **7**. ^b^ Activation energy of the 2π insertion step is calculated relative to the acyloxy migration product **9**; Pathway 1 is the 2π insertion into Rh-C5(sp^2^) and Pathway 2 is the 2π insertion into Rh-C1(sp^3^) bond. ^c^ Activation energy of reductive elimination is calculated relative to the corresponding insertion product **12d** or **13d**.

Because the molecular systems in this study are relatively large, dispersion effects may be important. To address this issue, we selected entry d as the test case and performed the M062X-D3 correction. The reason for using M062X is that M062X is a dispersion-corrected functional. The results in Table S4 showed that the activation barriers for all the three steps, including the 1,2-acyloxy migration, the 2π insertion and the reductive elimination, are little changed with dispersion correction. These results confirms that our conclusions are reliable based on the M06/SDD-6-311+G(d,p)/SMD(CHCl3)//B3LYP/SDD-6-31G(d) computed energies. Thus, we did not apply the M062X-D3 correction for the remaining entries.

# Table S5: The B3LYP/SDD-6-31G(d) computed energies, enthalpies, free energies of all stationary points discussed in the text.

| Entry | | E0 | | E0+ZPE | | U | | H | | G | | G(qh) |
| --- | --- | --- | --- | --- | --- | --- | --- | --- | --- | --- | --- | --- |
| **ACE** | | -461.218969 | | -461.059086 | | -461.047593 | | -461.046648 | | -461.096174 | | -461.095496 |
| **7** | | -2068.398859 | | -2067.959123 | | -2067.927504 | | -2067.926559 | | -2068.024607 | | -2068.016224 |
| **TS1** | | -2068.378324 | | -2067.938732 | | -2067.908301 | | -2067.907357 | | -2068.002912 | | -2067.994471 |
| **8** | | -2068.418251 | | -2067.977518 | | -2067.946096 | | -2067.945152 | | -2068.044113 | | -2068.034597 |
| **9** | | -2068.430490 | | -2067.989816 | | -2067.958360 | | -2067.957416 | | -2068.057171 | | -2068.046992 |
| **a** | | -116.616833 | | -116.560527 | | -116.557211 | | -116.556267 | | -116.584472 | | -116.584471 |
| **10a***-endo-re* | | -2185.048134 | | -2184.548774 | | -2184.513399 | | -2184.512455 | | -2184.619372 | | -2184.609233 |
| **10a***-endo-si* | | -2185.048134 | | -2184.548783 | | -2184.513402 | | -2184.512458 | | -2184.619422 | | -2184.609244 |
| **10a***-exo-re* | | -2185.050702 | | -2184.550585 | | -2184.515531 | | -2184.514586 | | -2184.620578 | | -2184.610703 |
| **10a***-exo-si* | | -2185.050702 | | -2184.550586 | | -2184.515531 | | -2184.514586 | | -2184.620593 | | -2184.610701 |
| **11a***-endo-re* | | -2185.057362 | | -2184.556682 | | -2184.521989 | | -2184.521045 | | -2184.625283 | | -2184.616690 |
| **11a***-exo-re* | | -2185.055693 | | -2184.555368 | | -2184.520562 | | -2184.519618 | | -2184.624566 | | -2184.615329 |
| **TS2a***-endo-re* | | -2185.042135 | | -2184.542667 | | -2184.508297 | | -2184.507353 | | -2184.612292 | | -2184.602152 |
| **TS2a***-endo-si* | | -2185.042135 | | -2184.542666 | | -2184.508296 | | -2184.507352 | | -2184.612290 | | -2184.602151 |
| **TS2a***-exo-re* | | -2185.039032 | | -2184.539921 | | -2184.505389 | | -2184.504445 | | -2184.609938 | | -2184.599830 |
| **TS2a***-exo-si* | | -2185.039032 | | -2184.539920 | | -2184.505388 | | -2184.504444 | | -2184.609934 | | -2184.599830 |
| **TS3a***-endo-re* | | -2185.044544 | | -2184.543737 | | -2184.509781 | | -2184.508837 | | -2184.611769 | | -2184.602697 |
| **TS3a***-endo-si* | | -2185.044072 | | -2184.542591 | | -2184.508869 | | -2184.507925 | | -2184.609266 | | -2184.601579 |
| **TS3a**-*exo*-*re* | | -2185.041314 | | -2184.540470 | | -2184.506511 | | -2184.505567 | | -2184.608939 | | -2184.599349 |
| **TS3a***-exo-si* | | -2185.044027 | | -2184.542761 | | -2184.508955 | | -2184.508011 | | -2184.609695 | | -2184.601743 |
| **12a***-endo-re* | | -2185.089203 | | -2184.586806 | | -2184.552578 | | -2184.551634 | | -2184.656498 | | -2184.646022 |
| **12a***-endo-si* | | -2185.089203 | | -2184.586807 | | -2184.552578 | | -2184.551634 | | -2184.656496 | | -2184.646023 |
| **12a***-exo-re* | | -2185.088119 | | -2184.585544 | | -2184.551507 | | -2184.550563 | | -2184.653923 | | -2184.644898 |
| **12a***-exo-si* | | -2185.089106 | | -2184.587150 | | -2184.552856 | | -2184.551912 | | -2184.656540 | | -2184.646760 |
| **13a***-endo-re* | | -2185.106999 | | -2184.603425 | | -2184.569115 | | -2184.568171 | | -2184.673128 | | -2184.662914 |
| **13a***-exo-re* | | -2185.110180 | | -2184.606543 | | -2184.572262 | | -2184.571318 | | -2184.676594 | | -2184.665973 |
| **TS4a***-endo-re* | | -2185.062150 | | -2184.559441 | | -2184.526453 | | -2184.525509 | | -2184.624880 | | -2184.617515 |
| **TS4a***-exo-re* | | -2185.055415 | | -2184.551802 | | -2184.518860 | | -2184.517916 | | -2184.617138 | | -2184.610009 |
| **TS5a***-endo-re* | | -2185.079246 | | -2184.575734 | | -2184.542236 | | -2184.541292 | | -2184.644064 | | -2184.634568 |
| **TS5a***-exo-re* | | -2185.084017 | | -2184.580595 | | -2184.547185 | | -2184.546241 | | -2184.648284 | | -2184.639253 |
| **14a** | | -2185.157155 | | -2184.650293 | | -2184.616735 | | -2184.615791 | | -2184.718970 | | -2184.709126 |
| **15a** | | -577.987065 | | -577.759567 | | -577.746725 | | -577.745780 | | -577.799520 | | -577.797613 |
| **16a** | | -2185.136538 | | -2184.631027 | | -2184.597524 | | -2184.596580 | | -2184.700206 | | -2184.689556 |
| **TS6a** | | -2185.112091 | | -2184.607704 | | -2184.574469 | | -2184.573525 | | -2184.675256 | | -2184.666108 |
| **TS7a** | | -2185.049862 | | -2184.550229 | | -2184.515791 | | -2184.514846 | | -2184.620408 | | -2184.610300 |
| **17a** | | -2185.146753 | | -2184.641583 | | -2184.608090 | | -2184.607146 | | -2184.709141 | | -2184.700228 |
| **TS8a** | | -2185.091045 | | -2184.587467 | | -2184.554287 | | -2184.553343 | | -2184.655599 | | -2184.645829 |
| **18a** | | -2185.153985 | | -2184.647221 | | -2184.613738 | | -2184.612794 | | -2184.715380 | | -2184.706002 |
| **TS9a** | | -2185.071645 | | -2184.569581 | | -2184.536223 | | -2184.535279 | | -2184.636897 | | -2184.628245 |
| **19a** | | -2185.124792 | | -2184.619398 | | -2184.585936 | | -2184.584992 | | -2184.688578 | | -2184.677850 |
| **TS10a** | | -2185.050827 | | -2184.546750 | | -2184.513644 | | -2184.512700 | | -2184.615598 | | -2184.605516 |
| **20a** | | -577.974593 | | -577.745603 | | -577.733666 | | -577.732722 | | -577.784038 | | -577.783011 |
| **Rh(PPh_3_)Cl** | | -1607.103923 | | -1606.827400 | | -1606.807761 | | -1606.806817 | | -1606.879305 | | -1606.873421 |
| **a’** | | -614.8744308 | | -614.6639 | | -614.650825 | | -614.64988 | | -614.705768 | | -614.7026328 |
| **10a׳***-endo-re* | | -2683.302579 | | -2682.649748 | | -2682.603802 | | -2682.602858 | | -2682.736699 | | -2682.719338 |
| **TS2a׳***-endo-re* | | -2683.284188 | | -2682.631486 | | -2682.586446 | | -2682.585502 | | -2682.715856 | | -2682.700489 |
| **12a׳***-endo-re* | | -2683.324353 | | -2682.669296 | | -2682.624287 | | -2682.623342 | | -2682.753285 | | -2682.73842 |
| **TS4a׳***-endo-re* | | -2683.293733 | | -2682.63855 | | -2682.594661 | | -2682.593717 | | -2682.719387 | | -2682.706526 |
| **14a׳** | | -2683.393183 | | -2682.734398 | | -2682.68992 | | -2682.688976 | | -2682.819031 | | -2682.803022 |
| **b** | | -155.970331 | | -155.883355 | | -155.879549 | | -155.878605 | | -155.908966 | | -155.908966 |
| **10b***-endo-re* | | -2224.380494 | | -2223.850612 | | -2223.814861 | | -2223.813916 | | -2223.921578 | | -2223.911463 |
| **10b***-exo-re* | | -2224.386184 | | -2223.856161 | | -2223.820289 | | -2223.819345 | | -2223.928342 | | -2223.917064 |
| **11b***-endo-re* | | -2224.402261 | | -2223.871302 | | -2223.835758 | | -2223.834813 | | -2223.941264 | | -2223.931924 |
| **11b***-exo-re* | | -2224.399801 | | -2223.869139 | | -2223.833502 | | -2223.832557 | | -2223.939001 | | -2223.929909 |
| **TS2b***-endo-re* | | -2224.378494 | | -2223.848839 | | -2223.813792 | | -2223.812848 | | -2223.918792 | | -2223.909018 |
| **TS2b***-exo-re* | | -2224.383817 | | -2223.854400 | | -2223.819230 | | -2223.818286 | | -2223.924744 | | -2223.914815 |
| **TS3b***-endo-re* | | -2224.380958 | | -2223.849463 | | -2223.814757 | | -2223.813812 | | -2223.918040 | | -2223.909345 |
| **TS3b**-*exo*-*re* | | -2224.378069 | | -2223.846955 | | -2223.812161 | | -2223.811217 | | -2223.916418 | | -2223.906777 |
| **12b***-endor-re* | | -2224.413147 | | -2223.880468 | | -2223.845315 | | -2223.844371 | | -2223.950447 | | -2223.940863 |
| **12b***-exo-re* | | -2224.426728 | | -2223.893922 | | -2223.858821 | | -2223.857877 | | -2223.963674 | | -2223.954200 |
| **13b**-*endo-re* | | -2224.429402 | | -2223.896102 | | -2223.860894 | | -2223.859950 | | -2223.966786 | | -2223.956366 |
| **13b***-exo-re* | | -2224.427454 | | -2223.894032 | | -2223.858867 | | -2223.857922 | | -2223.964937 | | -2223.954237 |
| **TS4b***-endo-re* | | -2224.373775 | | -2223.841357 | | -2223.807117 | | -2223.806172 | | -2223.910257 | | -2223.900600 |
| **TS4b***-exo-re* | | -2224.373694 | | -2223.841259 | | -2223.806796 | | -2223.805852 | | -2223.909919 | | -2223.900924 |
| **TS5b***-endo-re* | | -2224.396104 | | -2223.863192 | | -2223.828693 | | -2223.827749 | | -2223.933408 | | -2223.922782 |
| **TS5b***-exo-re* | | -2224.394223 | | -2223.861048 | | -2223.826613 | | -2223.825669 | | -2223.930221 | | -2223.920730 |
| **14b** | | -2224.474917 | | -2223.938512 | | -2223.904157 | | -2223.903213 | | -2224.007251 | | -2223.998135 |
| **15b** | | -617.304727 | | -617.047804 | | -617.034011 | | -617.033066 | | -617.088982 | | -617.087025 |
| **c** | | -195.323501 | | -195.206165 | | -195.201525 | | -195.200581 | | -195.233565 | | -195.233566 |
| **10c***-endo-re* | | -2263.731830 | | -2263.171646 | | -2263.135089 | | -2263.134144 | | -2263.242942 | | -2263.233201 |
| **10c**-*exo*-*re* | | -2263.738970 | | -2263.178232 | | -2263.141630 | | -2263.140686 | | -2263.249704 | | -2263.239816 |
| **11c***-endo-re* | | -2263.751481 | | -2263.190388 | | -2263.153776 | | -2263.152831 | | -2263.261175 | | -2263.252137 |
| **11c***-exo-re* | | -2263.750318 | | -2263.189020 | | -2263.152504 | | -2263.151560 | | -2263.259642 | | -2263.250810 |
| **TS2c***-endo-re* | | -2263.727413 | | -2263.167157 | | -2263.131351 | | -2263.130407 | | -2263.238027 | | -2263.227869 |
| **TS2c***-exo-re* | | -2263.734584 | | -2263.174112 | | -2263.138271 | | -2263.137327 | | -2263.244621 | | -2263.235047 |
| **TS3c***-endo-re* | | -2263.728036 | | -2263.166486 | | -2263.130887 | | -2263.129943 | | -2263.236385 | | -2263.227031 |
| **TS3c***-exo-re* | | -2263.722956 | | -2263.161551 | | -2263.125745 | | -2263.124801 | | -2263.233060 | | -2263.222164 |
| **12c***-endo-re* | | -2263.757942 | | -2263.194598 | | -2263.158733 | | -2263.157788 | | -2263.265322 | | -2263.255516 |
| **12c**-*exo*-*re* | | -2263.770345 | | -2263.207373 | | -2263.171446 | | -2263.170501 | | -2263.277742 | | -2263.268194 |
| **13c***-endo-re* | | -2263.766730 | | -2263.203128 | | -2263.166958 | | -2263.166014 | | -2263.275101 | | -2263.264378 |
| **13c***-exo-re* | | -2263.763682 | | -2263.200198 | | -2263.163858 | | -2263.162914 | | -2263.272013 | | -2263.261638 |
| **TS4c***-endo-re* | | -2263.710644 | | -2263.148405 | | -2263.112999 | | -2263.112055 | | -2263.218963 | | -2263.208803 |
| **TS4c***-exo-re* | | -2263.713446 | | -2263.150538 | | -2263.115329 | | -2263.114385 | | -2263.219432 | | -2263.210839 |
| **TS5c***-endo-re* | | -2263.732033 | | -2263.169266 | | -2263.133905 | | -2263.132961 | | -2263.239923 | | -2263.229501 |
| **TS5c***-exo-re* | | -2263.731383 | | -2263.168188 | | -2263.132701 | | -2263.131756 | | -2263.238695 | | -2263.228791 |
| **14c** | | -2263.816942 | | -2263.250949 | | -2263.215343 | | -2263.214399 | | -2263.321912 | | -2263.311806 |
| **15c** | | -656.644087 | | -656.357293 | | -656.342488 | | -656.341544 | | -656.400547 | | -656.397459 |
| **d** | | -234.643977 | | -234.497017 | | -234.491518 | | -234.490574 | | -234.525008 | | -234.525008 |
| **11d***-endo-re* | | -2303.068074 | | -2302.477307 | | -2302.439731 | | -2302.438787 | | -2302.548638 | | -2302.539871 |
| **11d**-*exo*-*re* | | -2303.065971 | | -2302.475713 | | -2302.437958 | | -2302.437014 | | -2302.548126 | | -2302.538554 |
| **TS2d***-endo-re* | | -2303.032568 | | -2302.443583 | | -2302.406315 | | -2302.405371 | | -2302.516734 | | -2302.505644 |
| **TS2d***-exo-re* | | -2303.039579 | | -2302.450221 | | -2302.413087 | | -2302.412142 | | -2302.522568 | | -2302.512191 |
| **TS3d***-endo-re* | | -2303.040479 | | -2302.449349 | | -2302.412577 | | -2302.411633 | | -2302.520886 | | -2302.510689 |
| **TS3d***-exo-re* | | -2303.036003 | | -2302.444522 | | -2302.407889 | | -2302.406944 | | -2302.515366 | | -2302.505824 |
| **12d***-endo-re* | | -2303.060515 | | -2302.468572 | | -2302.431751 | | -2302.430807 | | -2302.539171 | | -2302.530392 |
| **12d***-exo-re* | | -2303.065088 | | -2302.473595 | | -2302.436407 | | -2302.435463 | | -2302.545511 | | -2302.535581 |
| **13d***-endo-re* | | -2303.085213 | | -2302.492043 | | -2302.455002 | | -2302.454058 | | -2302.563922 | | -2302.553987 |
| **13d***-exo-re* | | -2303.082948 | | -2302.489937 | | -2302.452925 | | -2302.451981 | | -2302.561647 | | -2302.551767 |
| **TS4d***-endo-re* | | -2303.032166 | | -2302.439852 | | -2302.403978 | | -2302.403033 | | -2302.508582 | | -2302.500486 |
| **TS4d***-exo-re* | | -2303.034985 | | -2302.442577 | | -2302.406528 | | -2302.405583 | | -2302.511747 | | -2302.503435 |
| **TS5d***-endo-re* | | -2303.041750 | | -2302.449008 | | -2302.412636 | | -2302.411692 | | -2302.520218 | | -2302.510142 |
| **TS5d***-exo-re* | | -2303.041327 | | -2302.448590 | | -2302.412385 | | -2302.411441 | | -2302.518085 | | -2302.509712 |
| **14d** | | -2303.132950 | | -2302.537342 | | -2302.500911 | | -2302.499967 | | -2302.609151 | | -2302.598440 |
| **15d** | | -695.962622 | | -695.646421 | | -695.630711 | | -695.629767 | | -695.689943 | | -695.687546 |
| **e** | | -78.585824 | | -78.534577 | | -78.531535 | | -78.530590 | | -78.556762 | | -78.556761 |
| **10e** | | -2147.020061 | | -2146.524799 | | -2146.490060 | | -2146.489116 | | -2146.594946 | | -2146.584554 |
| **11e** | | -2147.024789 | | -2146.528504 | | -2146.494428 | | -2146.493484 | | -2146.596229 | | -2146.587906 |
| **TS2e** | | -2146.994455 | | -2146.499921 | | -2146.465949 | | -2146.465005 | | -2146.569284 | | -2146.559366 |
| **TS3e** | | -2146.997693 | | -2146.500879 | | -2146.467583 | | -2146.466639 | | -2146.568199 | | -2146.559374 |
| **12e** | | -2147.024274 | | -2146.526774 | | -2146.493255 | | -2146.492311 | | -2146.594405 | | -2146.585437 |
| **13e** | | -2147.044296 | | -2146.545928 | | -2146.512118 | | -2146.511174 | | -2146.615551 | | -2146.604902 |
| **TS4e** | | -2146.995047 | | -2146.496964 | | -2146.464437 | | -2146.463493 | | -2146.562358 | | -2146.554820 |
| **TS5e** | | -2147.008393 | | -2146.510200 | | -2146.477090 | | -2146.476146 | | -2146.577916 | | -2146.568724 |
| **14e** | | -2147.091093 | | -2146.589714 | | -2146.556684 | | -2146.555740 | | -2146.657521 | | -2146.648151 |
| **15e** | | -539.914351 | | -539.692012 | | -539.679631 | | -539.678687 | | -539.731303 | | -539.729479 |
| **f** | | -157.221726 | | -157.113032 | | -157.107556 | | -157.106612 | | -157.140152 | | -157.140152 |
| **10f***-exo-re* | | -2225.648983 | | -2225.097039 | | -2225.059170 | | -2225.058225 | | -2225.170783 | | -2225.159622 |
| **11f***-endo-re* | | -2225.651466 | | -2225.098714 | | -2225.061535 | | -2225.060591 | | -2225.169663 | | -2225.160807 |
| **11f***-exo-re* | -2225.648428 | | -2225.096105 | | -2225.058730 | | -2225.057786 | | -2225.168193 | | -2225.158366 | |
| **TS2f***-endo-re* | -2225.618666 | | -2225.067086 | | -2225.030486 | | -2225.029541 | | -2225.138103 | | -2225.128670 | |
| **TS2f***-exo-re* | -2225.621526 | | -2225.070014 | | -2225.033323 | | -2225.032379 | | -2225.141634 | | -2225.131580 | |
| **TS3f***-endo-re* | -2225.623538 | | -2225.069771 | | -2225.033771 | | -2225.032827 | | -2225.139362 | | -2225.130437 | |
| **TS3f***-exo-re* | -2225.618737 | | -2225.065301 | | -2225.029000 | | -2225.028056 | | -2225.136549 | | -2225.126193 | |
| **12f***-endo-re* | -2225.644831 | | -2225.090542 | | -2225.054188 | | -2225.053244 | | -2225.160717 | | -2225.151641 | |
| **12f***-exo-re* | -2225.651698 | | -2225.097761 | | -2225.061277 | | -2225.060332 | | -2225.167831 | | -2225.159077 | |
| **13f***-endo-re* | -2225.654889 | | -2225.099825 | | -2225.063322 | | -2225.062378 | | -2225.170937 | | -2225.161082 | |
| **13f***-exo-re* | -2225.661224 | | -2225.106416 | | -2225.069644 | | -2225.068700 | | -2225.178629 | | -2225.167873 | |
| **TS4f***-endo-re* | -2225.613811 | | -2225.059061 | | -2225.023718 | | -2225.022774 | | -2225.127125 | | -2225.119233 | |
| **TS4f***-exo-re* | -2225.615933 | | -2225.061054 | | -2225.025620 | | -2225.024676 | | -2225.129486 | | -2225.121094 | |
| **TS5f***-endo-re* | -2225.625983 | | -2225.071092 | | -2225.035230 | | -2225.034286 | | -2225.142344 | | -2225.131676 | |
| **TS5f***-exo-re* | -2225.620683 | | -2225.065994 | | -2225.030192 | | -2225.029248 | | -2225.135583 | | -2225.126674 | |
| **14f** | -2225.710520 | | -2225.152848 | | -2225.116785 | | -2225.115841 | | -2225.224005 | | -2225.213667 | |
| **15f** | -618.540171 | | -618.261717 | | -618.246445 | | -618.245501 | | -618.304402 | | -618.302127 | |
| **g** | -77.323802 | | -77.297151 | | -77.294219 | | -77.293275 | | -77.316177 | | -77.316177 | |
| **10g** | -2145.760817 | | -2145.290768 | | -2145.256220 | | -2145.255276 | | -2145.359967 | | -2145.350413 | |
| **11g** | -2145.752198 | | -2145.281730 | | -2145.247483 | | -2145.246538 | | -2145.349507 | | -2145.341380 | |
| **TS2g** | -2145.751052 | | -2145.281405 | | -2145.247717 | | -2145.246772 | | -2145.349916 | | -2145.340350 | |
| **TS3g** | -2145.737780 | | -2145.267331 | | -2145.233911 | | -2145.232967 | | -2145.334889 | | -2145.325761 | |
| **12g** | -2145.822314 | | -2145.347488 | | -2145.314120 | | -2145.313176 | | -2145.417455 | | -2145.406081 | |
| **13g** | -2145.812876 | | -2145.338713 | | -2145.305203 | | -2145.304259 | | -2145.407530 | | -2145.397540 | |
| **TS4g** | -2145.792541 | | -2145.317793 | | -2145.285280 | | -2145.284336 | | -2145.385090 | | -2145.375769 | |
| **TS5g** | -2145.801794 | | -2145.327759 | | -2145.295161 | | -2145.294217 | | -2145.394674 | | -2145.385697 | |
| **14g** | -2145.865942 | | -2145.388557 | | -2145.356022 | | -2145.355077 | | -2145.456113 | | -2145.446434 | |
| **15g** | -538.695210 | | -538.496941 | | -538.484988 | | -538.484044 | | -538.535430 | | -538.534050 | |

E0 is the electronic energy calculated by using B3LYP/SDD-6-31G(d). U, H and G are the thermal energy, enthalpy and Gibbs free energy obtained by adding the thermal corrections from frequency calculations to E0. G(qh) is the Gibbs free energy calculated using Truhlar’s quasi-harmonic approximation. All energies are in hartree.

# Table S6: The M06/SDD-6-311+G(d,p)/SMD(CHCl_3_)//B3LYP/SDD-6-31G(d) computed energies, enthalpies, free energies of all stationary points discussed in the text.

| Entry | E0 | E0+ZPE | U | H | G | G(qh) |
| --- | --- | --- | --- | --- | --- | --- |
| **ACE** | -461.058775 | -460.898891 | -460.887398 | -460.886453 | -460.935979 | -460.935302 |
| **7** | -2067.861460 | -2067.421723 | -2067.390104 | -2067.389159 | -2067.487207 | -2067.478824 |
| **TS1** | -2067.833278 | -2067.393686 | -2067.363255 | -2067.362311 | -2067.457866 | -2067.449424 |
| **8** | -2067.867557 | -2067.426824 | -2067.395402 | -2067.394458 | -2067.493419 | -2067.483903 |
| **9** | -2067.877475 | -2067.436801 | -2067.405345 | -2067.404401 | -2067.504156 | -2067.493977 |
| **a** | -116.565444 | -116.509137 | -116.505821 | -116.504877 | -116.533082 | -116.533082 |
| **10a***-endo-si* | -2184.452552 | -2183.953202 | -2183.917821 | -2183.916877 | -2184.023841 | -2184.013663 |
| **10a***-exo-si* | -2184.455999 | -2183.955882 | -2183.920827 | -2183.919882 | -2184.025889 | -2184.015997 |
| **11a***-endo-re* | -2184.462808 | -2183.962128 | -2183.927435 | -2183.926491 | -2184.030729 | -2184.022136 |
| **11a***-exo-re* | -2184.462444 | -2183.962119 | -2183.927313 | -2183.926369 | -2184.031317 | -2184.022080 |
| **TS2a***-endo-re* | -2184.446465 | -2183.946998 | -2183.912628 | -2183.911684 | -2184.016623 | -2184.006483 |
| **TS2a***-endo-si* | -2184.446463 | -2183.946994 | -2183.912624 | -2183.911680 | -2184.016618 | -2184.006479 |
| **TS2a***-exo-re* | -2184.443806 | -2183.944695 | -2183.910163 | -2183.909219 | -2184.014712 | -2184.004604 |
| **TS2a***-exo-si* | -2184.443807 | -2183.944694 | -2183.910162 | -2183.909218 | -2184.014708 | -2184.004604 |
| **TS3a***-endo-re* | -2184.448612 | -2183.947805 | -2183.913849 | -2183.912905 | -2184.015837 | -2184.006765 |
| **TS3a***-endo-si* | -2184.446732 | -2183.945251 | -2183.911529 | -2183.910585 | -2184.011926 | -2184.004239 |
| **TS3a***-exo-re* | -2184.445970 | -2183.945126 | -2183.911167 | -2183.910223 | -2184.013595 | -2184.004005 |
| **TS3a***-exo-si* | -2184.447089 | -2183.945824 | -2183.912018 | -2183.911074 | -2184.012758 | -2184.004806 |
| **12a***-endo-re* | -2184.493849 | -2183.991452 | -2183.957224 | -2183.956280 | -2184.061144 | -2184.050669 |
| **12a***-endo-si* | -2184.493847 | -2183.991451 | -2183.957222 | -2183.956278 | -2184.061140 | -2184.050667 |
| **12a***-exo-re* | -2184.494756 | -2183.992181 | -2183.958144 | -2183.957200 | -2184.060560 | -2184.051535 |
| **12a***-exo-si* | -2184.497538 | -2183.995582 | -2183.961288 | -2183.960344 | -2184.064972 | -2184.055192 |
| **13a***-endo-re* | -2184.507246 | -2184.003672 | -2183.969362 | -2183.968418 | -2184.073375 | -2184.063160 |
| **13a***-exo-re* | -2184.510748 | -2184.007111 | -2183.972830 | -2183.971886 | -2184.077162 | -2184.066541 |
| **TS4a***-endo-re* | -2184.476902 | -2183.974193 | -2183.941205 | -2183.940261 | -2184.039632 | -2184.032267 |
| **TS4a***-exo-re* | -2184.472176 | -2183.968563 | -2183.935621 | -2183.934677 | -2184.033899 | -2184.026770 |
| **TS5a***-endo-re* | -2184.487470 | -2183.983958 | -2183.950460 | -2183.949516 | -2184.052288 | -2184.042792 |
| **TS5a***-exo-re* | -2184.494447 | -2183.991025 | -2183.957615 | -2183.956671 | -2184.058714 | -2184.049683 |
| **14a** | -2184.566016 | -2184.059154 | -2184.025596 | -2184.024652 | -2184.127831 | -2184.117987 |
| **15a** | -577.768175 | -577.540677 | -577.527835 | -577.526890 | -577.580630 | -577.578723 |
| **TS6a** | -2184.517414 | -2184.013027 | -2183.979792 | -2183.978848 | -2184.080579 | -2184.071431 |
| **TS7a** | -2184.460014 | -2183.960381 | -2183.925943 | -2183.924998 | -2184.030560 | -2184.020451 |
| **17a** | -2184.513498 | -2184.008328 | -2183.974835 | -2183.973891 | -2184.075886 | -2184.066973 |
| **TS8a** | -2184.500375 | -2183.996797 | -2183.963617 | -2183.962673 | -2184.064929 | -2184.055159 |
| **TS9a** | -2184.476397 | -2183.974333 | -2183.940975 | -2183.940031 | -2184.041649 | -2184.032998 |
| **19a** | -2184.531537 | -2184.026143 | -2183.992681 | -2183.991737 | -2184.095323 | -2184.084595 |
| **TS10a** | -2184.467666 | -2183.963588 | -2183.930482 | -2183.929538 | -2184.032436 | -2184.022354 |
| **18a** | -2,184.558114 | -2184.051350 | -2184.017867 | -2184.016923 | -2184.119509 | -2184.110131 |
| **20a** | -577.758237 | -577.529248 | -577.517311 | -577.516367 | -577.567683 | -577.566656 |
| **Rh(PPh_3_)Cl** | -1606.728011 | -1606.451488 | -1606.431849 | -1606.430905 | -1606.503393 | -1606.497508 |
| **a’** | -614.634931 | -614.424400 | -614.411325 | -614.410381 | -614.466269 | -614.463132 |
| **10a׳***-endo-re* | -2682.522026 | -2681.869195 | -2681.823249 | -2681.822304 | -2681.956146 | -2681.938785 |
| **TS2a׳***-endo-re* | -2682.507368 | -2681.854666 | -2681.809627 | -2681.808683 | -2681.939036 | -2681.923670 |
| **12a׳***-endo-re* | -2682.549572 | -2681.894515 | -2681.849506 | -2681.848561 | -2681.978504 | -2681.963639 |
| **TS4a׳***-endo-re* | -2682.528196 | -2681.873012 | -2681.829124 | -2681.828180 | -2681.953850 | -2681.940989 |
| **14a׳** | -2682.621283 | -2681.962498 | -2681.918020 | -2681.917076 | -2682.047132 | -2682.031122 |
| **b** | -155.888574 | -155.801598 | -155.797792 | -155.796848 | -155.827209 | -155.827209 |
| **10b***-endo-re* | -2223.759076 | -2223.229194 | -2223.193443 | -2223.192498 | -2223.300160 | -2223.290044 |
| **10b***-exo-re* | -2223.762985 | -2223.232962 | -2223.197090 | -2223.196146 | -2223.305143 | -2223.293865 |
| **11b***-endo-re* | -2223.782774 | -2223.251815 | -2223.216271 | -2223.215326 | -2223.321777 | -2223.312437 |
| **11b***-exo-re* | -2223.781246 | -2223.250585 | -2223.214948 | -2223.214003 | -2223.320447 | -2223.311355 |
| **TS2b***-endo-re* | -2223.757846 | -2223.228191 | -2223.193144 | -2223.192200 | -2223.298144 | -2223.288370 |
| **TS2b***-exo-re* | -2223.761434 | -2223.232018 | -2223.196848 | -2223.195904 | -2223.302362 | -2223.292433 |
| **TS3b***-endo-re* | -2223.760240 | -2223.228745 | -2223.194039 | -2223.193094 | -2223.297322 | -2223.288627 |
| **TS3b***-exo-re* | -2223.756947 | -2223.225834 | -2223.191040 | -2223.190096 | -2223.295297 | -2223.285655 |
| **12b***-endo-re* | -2223.790211 | -2223.257532 | -2223.222379 | -2223.221435 | -2223.327511 | -2223.317927 |
| **12b***-exo-re* | -2223.802688 | -2223.269883 | -2223.234782 | -2223.233838 | -2223.339635 | -2223.330161 |
| **13b***-endo-re* | -2223.807284 | -2223.273984 | -2223.238776 | -2223.237832 | -2223.344668 | -2223.334248 |
| **13b***-exo-re* | -2223.804736 | -2223.271314 | -2223.236149 | -2223.235204 | -2223.342219 | -2223.331519 |
| **TS4b***-endo-re* | -2223.762207 | -2223.229789 | -2223.195549 | -2223.194604 | -2223.298689 | -2223.289032 |
| **TS4b***-exo-re* | -2223.763030 | -2223.230595 | -2223.196132 | -2223.195188 | -2223.299255 | -2223.290260 |
| **TS5b***-endo-re* | -2223.782924 | -2223.250012 | -2223.215513 | -2223.214569 | -2223.320228 | -2223.309603 |
| **TS5b***-exo-re* | -2223.783247 | -2223.250072 | -2223.215637 | -2223.214693 | -2223.319245 | -2223.309753 |
| **14b** | -2223.860607 | -2223.324203 | -2223.289848 | -2223.288904 | -2223.392942 | -2223.383826 |
| **15b** | -617.060816 | -616.803894 | -616.790101 | -616.789156 | -616.845072 | -616.843115 |
| **c** | -195.219120 | -195.101784 | -195.097144 | -195.096200 | -195.129184 | -195.129185 |
| **10c***-exo-re* | -2263.096523 | -2262.535785 | -2262.499183 | -2262.498239 | -2262.607257 | -2262.597369 |
| **TS2c***-endo-re* | -2263.089462 | -2262.529206 | -2262.493400 | -2262.492456 | -2262.600076 | -2262.589919 |
| **TS2c***-exo-re* | -2263.092131 | -2262.531659 | -2262.495818 | -2262.494874 | -2262.602168 | -2262.592594 |
| **TS3c***-endo-re* | -2263.086828 | -2262.525277 | -2262.489678 | -2262.488734 | -2262.595176 | -2262.585822 |
| **TS3c***-exo-re* | -2263.080982 | -2262.519577 | -2262.483771 | -2262.482827 | -2262.591086 | -2262.580190 |
| **12c***-endo-re* | -2263.115595 | -2262.552251 | -2262.516386 | -2262.515441 | -2262.622975 | -2262.613169 |
| **12c***-exo-re* | -2263.127349 | -2262.564377 | -2262.528450 | -2262.527505 | -2262.634746 | -2262.625198 |
| **13c***-endo-re* | -2263.124305 | -2262.560703 | -2262.524533 | -2262.523589 | -2262.632676 | -2262.621954 |
| **13c***-exo-re* | -2263.121263 | -2262.557779 | -2262.521439 | -2262.520495 | -2262.629594 | -2262.619219 |
| **TS4c***-endo-re* | -2263.079830 | -2262.517591 | -2262.482185 | -2262.481241 | -2262.588149 | -2262.577989 |
| **TS4c***-exo-re* | -2263.083280 | -2262.520372 | -2262.485163 | -2262.484219 | -2262.589266 | -2262.580674 |
| **TS5c***-endo-re* | -2263.098959 | -2262.536193 | -2262.500832 | -2262.499888 | -2262.606850 | -2262.596427 |
| **TS5c***-exo-re* | -2263.099982 | -2262.536787 | -2262.501300 | -2262.500355 | -2262.607294 | -2262.597390 |
| **14c** | -2263.166123 | -2262.600130 | -2262.564524 | -2262.563580 | -2262.671093 | -2262.660987 |
| **15c** | -656.378746 | -656.091952 | -656.077147 | -656.076203 | -656.135206 | -656.132118 |
| **d** | -234.519099 | -234.372139 | -234.366640 | -234.365696 | -234.400130 | -234.400130 |
| **11d***-endo-re* | -2302.407840 | -2301.817073 | -2301.779497 | -2301.778553 | -2301.888404 | -2301.879637 |
| **11d***-exo-re* | -2302.405112 | -2301.814854 | -2301.777099 | -2301.776155 | -2301.887267 | -2301.877696 |
| **TS2d***-endo-re* | -2302.370024 | -2301.781039 | -2301.743771 | -2301.742827 | -2301.854190 | -2301.843100 |
| **TS2d***-exo-re* | -2302.381037 | -2301.791679 | -2301.754545 | -2301.753600 | -2301.864026 | -2301.853649 |
| **TS3d***-endo-re* | -2302.379041 | -2301.787911 | -2301.751139 | -2301.750195 | -2301.859448 | -2301.849251 |
| **TS3d***-exo-re* | -2302.374397 | -2301.782916 | -2301.746283 | -2301.745338 | -2301.853760 | -2301.844218 |
| **12d***-endo-re* | -2302.403920 | -2301.811977 | -2301.775156 | -2301.774212 | -2301.882576 | -2301.873797 |
| **12d***-exo-re* | -2302.407274 | -2301.815781 | -2301.778593 | -2301.777649 | -2301.887697 | -2301.877767 |
| **13d***-endo-re* | -2302.422576 | -2301.829406 | -2301.792365 | -2301.791421 | -2301.901285 | -2301.891349 |
| **13d***-exo-re* | -2302.423356 | -2301.830345 | -2301.793333 | -2301.792389 | -2301.902055 | -2301.892175 |
| **TS4d***-endo-re* | -2302.382654 | -2301.790341 | -2301.754467 | -2301.753522 | -2301.859071 | -2301.850975 |
| **TS4d***-exo-re* | -2302.381256 | -2301.788848 | -2301.752799 | -2301.751854 | -2301.858018 | -2301.849706 |
| **TS5d***-endo-re* | -2302.389589 | -2301.796847 | -2301.760475 | -2301.759531 | -2301.868057 | -2301.857981 |
| **TS5d***-exo-re* | -2302.392910 | -2301.800173 | -2301.763968 | -2301.763024 | -2301.869668 | -2301.861295 |
| **14d** | -2302.477787 | -2301.882179 | -2301.845748 | -2301.844804 | -2301.953988 | -2301.943277 |
| **15d** | -695.678140 | -695.361940 | -695.346230 | -695.345286 | -695.405462 | -695.403065 |
| **e** | -78.540403 | -78.489156 | -78.486114 | -78.485169 | -78.511341 | -78.511340 |
| **10e** | -2146.429124 | -2145.933862 | -2145.899123 | -2145.898179 | -2146.004009 | -2145.993616 |
| **11e** | -2146.439054 | -2145.942768 | -2145.908692 | -2145.907748 | -2146.010493 | -2146.002171 |
| **TS2e** | -2146.407678 | -2145.913144 | -2145.879172 | -2145.878228 | -2145.982507 | -2145.972589 |
| **TS3e** | -2146.409837 | -2145.913023 | -2145.879727 | -2145.878783 | -2145.980343 | -2145.971518 |
| **12e** | -2146.442810 | -2145.945310 | -2145.911791 | -2145.910847 | -2146.012941 | -2146.003973 |
| **13e** | -2146.456002 | -2145.957634 | -2145.923824 | -2145.922880 | -2146.027257 | -2146.016609 |
| **TS4e** | -2146.420071 | -2145.921988 | -2145.889461 | -2145.888517 | -2145.987382 | -2145.979844 |
| **TS5e** | -2146.429851 | -2145.931658 | -2145.898548 | -2145.897604 | -2145.999374 | -2145.990181 |
| **14e** | -2146.511282 | -2146.009903 | -2145.976873 | -2145.975929 | -2146.077710 | -2146.068340 |
| **15e** | -539.706236 | -539.483897 | -539.471516 | -539.470572 | -539.523188 | -539.521364 |
| **f** | -157.133035 | -157.024341 | -157.018865 | -157.017921 | -157.051461 | -157.051461 |
| **11f***-endo-re* | -2225.028605 | -2224.475853 | -2224.438674 | -2224.437730 | -2224.546802 | -2224.537946 |
| **11f***-exo-re* | -2225.025394 | -2224.473071 | -2224.435696 | -2224.434752 | -2224.545159 | -2224.535332 |
| **TS2f***-endo-re* | -2224.992927 | -2224.441347 | -2224.404747 | -2224.403802 | -2224.512364 | -2224.502930 |
| **TS2f***-exo-re* | -2224.995806 | -2224.444294 | -2224.407603 | -2224.406659 | -2224.515914 | -2224.505861 |
| **TS3f***-endo-re* | -2224.999589 | -2224.445822 | -2224.409822 | -2224.408878 | -2224.515413 | -2224.506488 |
| **TS3f***-exo-re* | -2224.994189 | -2224.440752 | -2224.404451 | -2224.403507 | -2224.512000 | -2224.501644 |
| **12f***-endo-re* | -2225.024676 | -2224.470387 | -2224.434033 | -2224.433089 | -2224.540562 | -2224.531486 |
| **12f***-exo-re* | -2225.031601 | -2224.477664 | -2224.441180 | -2224.440235 | -2224.547734 | -2224.538980 |
| **13f***-endo-re* | -2225.031688 | -2224.476624 | -2224.440121 | -2224.439177 | -2224.547736 | -2224.537881 |
| **13f***-exo-re* | -2225.035174 | -2224.480367 | -2224.443595 | -2224.442651 | -2224.552580 | -2224.541823 |
| **TS4f***-endo-re* | -2225.002332 | -2224.447582 | -2224.412239 | -2224.411295 | -2224.515646 | -2224.507754 |
| **TS4f***-exo-re* | -2225.003945 | -2224.449066 | -2224.413632 | -2224.412688 | -2224.517498 | -2224.509106 |
| **TS5f***-endo-re* | -2225.009115 | -2224.454223 | -2224.418361 | -2224.417417 | -2224.525475 | -2224.514808 |
| **TS5f***-exo-re* | -2225.008045 | -2224.453356 | -2224.417554 | -2224.416610 | -2224.522945 | -2224.514035 |
| **14f** | -2225.091895 | -2224.534223 | -2224.498160 | -2224.497216 | -2224.605380 | -2224.595042 |
| **15f** | -618.291960 | -618.013506 | -617.998234 | -617.997290 | -618.056191 | -618.053916 |
| **g** | -77.291651 | -77.265000 | -77.262068 | -77.261124 | -77.284026 | -77.284026 |
| **10g** | -2145.177221 | -2144.707172 | -2144.672624 | -2144.671680 | -2144.776371 | -2144.766817 |
| **11g** | -2145.173230 | -2144.702762 | -2144.668515 | -2144.667570 | -2144.770539 | -2144.762412 |
| **TS2g** | -2145.166082 | -2144.696435 | -2144.662747 | -2144.661802 | -2144.764946 | -2144.755380 |
| **TS3g** | -2145.156168 | -2144.685719 | -2144.652299 | -2144.651355 | -2144.753277 | -2144.744149 |
| **12g** | -2145.234380 | -2144.759554 | -2144.726186 | -2144.725242 | -2144.829521 | -2144.818147 |
| **13g** | -2145.224140 | -2144.749977 | -2144.716467 | -2144.715523 | -2144.818794 | -2144.808804 |
| **TS4g** | -2145.212029 | -2144.737282 | -2144.704769 | -2144.703825 | -2144.804579 | -2144.795258 |
| **TS5g** | -2145.220026 | -2144.745991 | -2144.713393 | -2144.712449 | -2144.812906 | -2144.803929 |
| **14g** | -2145.286330 | -2144.808944 | -2144.776409 | -2144.775464 | -2144.876500 | -2144.866821 |
| **15g** | -538.488995 | -538.290726 | -538.278773 | -538.277829 | -538.329215 | -538.327835 |

E0 is the electronic energy calculated by using M06/SDD-6-311+G(d,p)/SMD(CHCl_3_) based on geometries obtained by B3LYP/SDD-6-31G(d). U, H, G, G(qh) are the results of adding corresponding corrections the same as in Table S3 to E0. All energies are in hartree.

The Cartesian Coordinates of the stationary points discussed in the text

For transition state structures, one imaginary frequency was observed and given below. For all minimum structures, no imaginary frequency was observed.

**ACE**

Atom X Y Z

C -1.675593 -1.181800 -0.075419

C -2.816500 -0.951445 -0.719410

H -3.092258 0.042837 -1.057849

H -3.512411 -1.759174 -0.927310

C -1.181120 2.242479 -0.759004

H -1.387959 3.202191 -1.175389

C -0.905490 1.171141 -0.276959

H -1.413724 -2.186587 0.252181

C -0.637931 -0.143125 0.320235

C -0.558577 -0.054796 1.858161

H -0.318481 -1.042382 2.266425

H 0.205351 0.660931 2.162003

H -1.529451 0.259826 2.251767

O 0.608906 -0.713968 -0.216917

C 1.781511 -0.041426 -0.086230

O 1.915749 1.014482 0.488109

C 2.890678 -0.806256 -0.775589

H 2.702452 -0.839694 -1.854194

H 3.843300 -0.310738 -0.584394

H 2.924068 -1.839955 -0.417480

**a**

Atom X Y Z

C -0.066279 1.075217 0.667821

H -0.102463 1.963495 1.293203

C -0.066279 -0.326321 1.237314

H -1.050465 -0.568364 1.667502

H 0.662540 -0.460251 2.047099

C 0.256675 -1.208013 0.000000

H -0.293160 -2.154410 0.000000

H 1.324580 -1.454014 0.000000

C -0.066279 -0.326321 -1.237314

H 0.662540 -0.460251 -2.047099

H -1.050465 -0.568364 -1.667502

C -0.066279 1.075217 -0.667821

H -0.102463 1.963495 -1.293203

**a′**

Atom X Y Z

C -0.13823000 2.14899700 -0.36485000

H -0.28685000 3.19723600 -0.15966600

C -0.83391000 0.90322300 -0.85323900

H -1.04297200 0.76376500 -1.91656300

C 0.59476400 1.07904600 -0.37489900

C 1.82426600 0.35158600 -0.16041200

C 1.88977700 -1.02290900 -0.44224200

C 2.96934900 1.00852000 0.32723500

C 3.07561100 -1.72669100 -0.23999100

H 1.00490600 -1.52877500 -0.81770200

C 4.15133200 0.30156400 0.52677700

H 2.91957600 2.07115600 0.54732400

C 4.20785200 -1.06728600 0.24386500

H 3.11650500 -2.79008400 -0.45917700

H 5.03087300 0.81587700 0.90442100

H 5.13186500 -1.61686800 0.40156500

C -1.82166600 0.22401700 0.05077800

O -1.82322200 0.28165300 1.26364800

O -2.72439600 -0.47930200 -0.67727400

C -3.74354000 -1.18412900 0.06647700

H -4.03946900 -2.00731400 -0.58930100

H -3.29910600 -1.58825400 0.97963900

C -4.92353500 -0.27766800 0.38771200

H -5.71491400 -0.85483100 0.88026800

H -4.61553400 0.52714100 1.06110200

H -5.33635100 0.16251900 -0.52616200

**b**

Atom X Y Z

C 0.814763 0.670381 -0.000129

C 0.814807 -0.670267 0.000027

H 1.601374 1.420746 0.000106

H 1.601600 -1.420456 0.000197

C -0.699714 0.786139 -0.000009

H -1.145981 1.246287 0.890382

H -1.146617 1.247006 -0.889614

C -0.699578 -0.786256 -0.000051

H -1.146039 -1.246722 0.890002

H -1.146002 -1.246849 -0.890100

**c**

Atom X Y Z

C 0.000067 -0.501079 0.647542

H -0.000652 -1.043814 1.580979

C 0.000067 0.861594 0.000000

H -0.913454 1.465481 0.000000

H 0.913553 1.465533 0.000000

C 0.000067 -0.501079 -0.647542

H -0.000652 -1.043814 -1.580979

**d**

Atom X Y Z

C -0.252003 0.724993 -1.192330

C 0.252003 1.481776 0.047617

C 0.120055 0.657852 1.306349

C -0.120055 -0.657852 1.306349

C -0.252003 -1.481776 0.047617

C 0.252003 -0.724993 -1.192330

H 1.305900 1.772709 -0.090886

H -0.298015 2.426159 0.163491

H -1.351064 0.722230 -1.190912

H 0.061375 1.243969 -2.106437

H -0.225700 -1.183531 2.254926

H 0.298015 -2.426159 0.163491

H -1.305900 -1.772709 -0.090886

H 1.351064 -0.722230 -1.190912

H -0.061375 -1.243969 -2.106437

H 0.225700 1.183531 2.254926

**e**

Atom X Y Z

C 0.665545 0.000000 0.000003

C -0.665545 0.000000 -0.000004

H 1.240366 0.922951 -0.000009

H 1.240366 -0.922952 0.000024

H -1.240366 -0.922951 0.000011

H -1.240366 0.922952 -0.000022

**f**

Atom X Y Z

C -0.000313 1.592775 -0.522108

C -0.000313 0.669344 0.664643

C 0.000313 -0.669344 0.664643

C 0.000313 -1.592775 -0.522108

H 0.879274 2.250953 -0.505491

H -0.880968 2.249526 -0.506875

H 0.000762 -1.169699 1.633857

H 0.880968 -2.249526 -0.506875

H -0.879274 -2.250953 -0.505491

H -0.000762 1.169699 1.633857

H -0.001011 -1.059291 -1.476702

H 0.001011 1.059291 -1.476702

**g**

Atom X Y Z

C 0.000000 0.000000 0.602513

C 0.000000 0.000000 -0.602513

H 0.000000 0.000000 1.668687

H 0.000000 0.000000 -1.668687

**15a**

Atom X Y Z

C -2.868825 -0.006166 0.080671

C -2.041676 0.774239 1.095847

C -1.041241 1.701956 0.452405

C 0.011472 1.287292 -0.276617

C -2.137127 -0.950903 -0.838898

H -1.491267 0.056702 1.720564

H -2.704817 1.332241 1.766788

H -2.538843 -1.048859 -1.848888

H -1.168577 2.772604 0.605100

C 0.240339 -0.144993 -0.553817

C -0.660140 -1.115966 -0.773253

H -0.264497 -2.106450 -0.995324

C 1.021385 2.263870 -0.838173

H 1.995279 2.168642 -0.341872

H 1.189783 2.101560 -1.909887

H 0.677395 3.292713 -0.695611

O 1.598139 -0.516634 -0.707469

C 2.367056 -0.570155 0.421117

O 1.947507 -0.357411 1.533119

C 3.789118 -0.934350 0.063525

H 4.386598 -1.000971 0.973344

H 3.810751 -1.890705 -0.469161

H 4.213870 -0.179403 -0.606611

H -3.696378 0.546002 -0.360265

C -3.048287 -1.494570 0.250390

H -2.582644 -1.960859 1.115267

H -3.984251 -1.952366 -0.057818

**15b**

Atom X Y Z

C -2.610235 0.453142 -0.153225

C -1.763954 1.111594 0.946918

C -0.604256 1.889812 0.387893

C 0.443032 1.357627 -0.269562

C -1.912179 -0.779953 -0.809625

H -1.389479 0.328293 1.623124

H -2.402154 1.771362 1.547170

H -2.231242 -0.867252 -1.858739

H -0.623503 2.970960 0.517076

C 0.542244 -0.090071 -0.531725

C -0.431014 -0.988679 -0.744434

H -0.101263 -2.010275 -0.933127

C 1.580437 2.224703 -0.764367

H 2.500972 2.046069 -0.194845

H 1.810039 2.030312 -1.819036

H 1.326950 3.283560 -0.656435

O 1.864945 -0.580677 -0.684014

C 2.636313 -0.684760 0.438311

O 2.252207 -0.413578 1.550430

C 4.015486 -1.183782 0.073705

H 4.612975 -1.294539 0.979254

H 3.944304 -2.143904 -0.447673

H 4.501574 -0.478492 -0.608819

H -2.943281 1.220358 -0.860591

C -3.722149 -0.498674 0.371561

H -4.617989 -0.483930 -0.258021

H -4.030878 -0.361738 1.413588

C -2.802252 -1.713687 0.069697

H -2.267969 -2.061279 0.959604

H -3.255111 -2.579107 -0.424721

**15c**

Atom X Y Z

C -2.248829 0.725719 0.047094

C -1.209399 1.373453 0.980645

C -0.077709 2.034704 0.244774

C 0.881205 1.365897 -0.421998

C -1.696994 -0.501334 -0.779723

H -0.804608 0.593204 1.641621

H -1.715347 2.107691 1.619317

H -1.842404 -0.260971 -1.843885

H -0.033320 3.122961 0.257458

C 0.830762 -0.101353 -0.512778

C -0.247316 -0.892967 -0.617849

H -0.041614 -1.960690 -0.681801

C 2.040395 2.072449 -1.087516

H 2.987311 1.866007 -0.572919

H 2.165328 1.755452 -2.130207

H 1.886912 3.155804 -1.072442

O 2.087051 -0.751618 -0.601360

C 2.860759 -0.788140 0.524093

O 2.520663 -0.340769 1.592710

C 4.177447 -1.467160 0.225576

H 4.772923 -1.522107 1.137518

H 4.002153 -2.473577 -0.168340

H 4.723194 -0.908272 -0.542123

H -2.576504 1.489106 -0.669313

C -3.491350 0.190205 0.799629

H -4.258645 0.960135 0.938876

H -3.195831 -0.156922 1.799956

C -2.648773 -1.679842 -0.434729

H -2.246873 -2.234689 0.424099

H -2.748522 -2.390974 -1.262805

C -3.968361 -1.009023 -0.033148

H -4.505917 -0.661416 -0.926058

H -4.640971 -1.677284 0.515835

**15d**

Atom X Y Z

C -1.903953 0.910700 0.465281

C -0.691338 1.527670 1.211256

C 0.389287 2.150810 0.359217

C 1.247451 1.433564 -0.394150

C -1.457477 0.258526 -0.876628

H -0.215641 0.719241 1.783488

H -1.056815 2.260512 1.940324

H -1.203876 1.101676 -1.535100

H 0.532965 3.228723 0.423935

C 1.009897 -0.010109 -0.565022

C -0.195710 -0.567856 -0.749693

H -0.249991 -1.647799 -0.870133

C 2.436529 2.048349 -1.094695

H 3.381711 1.675537 -0.679583

H 2.444767 1.812642 -2.166342

H 2.431656 3.136782 -0.980352

O 2.150419 -0.838904 -0.654627

C 2.846601 -1.065233 0.499461

O 2.509514 -0.650539 1.582511

C 4.071408 -1.902033 0.213198

H 4.596870 -2.107804 1.146304

H 3.782456 -2.841455 -0.269202

H 4.735160 -1.372262 -0.478402

H -2.607882 1.714019 0.195660

C -2.645058 -0.077737 1.384824

H -3.008368 0.453351 2.275005

H -1.929265 -0.831241 1.745266

C -2.615669 -0.520713 -1.538290

H -2.246697 -1.038873 -2.433071

H -3.363065 0.205692 -1.887949

C -3.803650 -0.791189 0.674846

H -4.574644 -0.057716 0.395088

H -4.282851 -1.503776 1.358295

C -3.305741 -1.508694 -0.587242

H -4.137918 -2.004006 -1.103761

H -2.603491 -2.304033 -0.296713

**15e**

Atom X Y Z

C -3.071217 -0.505467 -0.176312

C -2.628811 0.577489 0.830666

C -1.553083 1.481573 0.290725

C -0.378359 1.110018 -0.253081

C -2.124204 -1.706998 -0.221927

H -2.284030 0.088187 1.754523

H -3.492964 1.189300 1.112766

H -2.433509 -2.373706 -1.041568

H -1.763341 2.549435 0.323611

C 0.045089 -0.297008 -0.387933

C -0.651210 -1.444690 -0.371445

H -0.048277 -2.343307 -0.492337

C 0.588920 2.162107 -0.755881

H 1.488291 2.214336 -0.130308

H 0.916983 1.955846 -1.781435

H 0.117784 3.149128 -0.737879

O 1.430817 -0.459113 -0.664345

C 2.305483 -0.338009 0.376822

O 1.975172 -0.108517 1.515293

C 3.722517 -0.528262 -0.113529

H 4.409177 -0.478624 0.732231

H 3.821641 -1.492738 -0.621823

H 3.974133 0.250584 -0.841412

H -3.155398 -0.053174 -1.171620

H -4.071599 -0.871573 0.087163

H -2.257559 -2.307168 0.691875

**15f**

Atom X Y Z

C -2.502494 0.268040 0.495963

C -1.414747 1.117417 1.208910

C -0.484108 1.921608 0.331995

C 0.492551 1.371837 -0.418060

C -1.941378 -0.319131 -0.835093

H -0.789008 0.422414 1.786211

H -1.904119 1.779581 1.932880

H -1.864033 0.550830 -1.503165

H -0.555176 3.007864 0.374897

C 0.538381 -0.093276 -0.555313

C -0.540027 -0.876545 -0.705398

H -0.388646 -1.950344 -0.796283

C 1.532635 2.192475 -1.143743

H 2.534241 2.028096 -0.726000

H 1.585071 1.934555 -2.208964

H 1.310240 3.260552 -1.057348

O 1.814020 -0.691154 -0.648140

C 2.574656 -0.723411 0.487033

O 2.196071 -0.328544 1.563646

C 3.928379 -1.323836 0.188712

H 4.512682 -1.377596 1.107877

H 3.811022 -2.324624 -0.239554

H 4.455277 -0.713051 -0.552132

H -3.336266 0.927043 0.209005

C -3.046703 -0.783350 1.473638

H -3.374243 -0.307445 2.405744

H -2.274046 -1.518230 1.732634

C -2.887902 -1.330390 -1.499586

H -2.534974 -1.588758 -2.504533

H -3.899220 -0.917899 -1.595824

H -3.905009 -1.323452 1.060621

H -2.959980 -2.260573 -0.924471

**15g**

Atom X Y Z

C 2.241605 -1.512830 -0.467426

C 0.451423 1.012311 -0.361536

C 1.618981 1.378099 0.215854

C 3.033320 -0.611580 0.152036

H 2.700860 -2.323741 -1.030938

H 4.110818 -0.644522 0.004321

C -0.420500 2.025286 -1.073661

H 0.111579 2.975196 -1.181150

H -0.719208 1.681456 -2.070695

H -1.337163 2.225828 -0.505644

C 0.014738 -0.377820 -0.366493

H 1.977808 2.395417 0.069718

O -1.362572 -0.608295 -0.570046

C -2.229757 -0.262197 0.430380

O -1.888558 0.211088 1.486652

C 0.796228 -1.489104 -0.444669

H 0.272547 -2.427960 -0.612318

C -3.648472 -0.563132 0.008239

H -3.749012 -1.622205 -0.250522

H -4.329937 -0.311553 0.821510

H -3.904936 0.015827 -0.885372

C 2.448084 0.443916 1.058764

H 1.807186 -0.035401 1.813791

H 3.234597 0.991620 1.585520

**7a**

Atom X Y Z

C -2.023672 0.862966 -0.034869

C -1.616949 0.672901 -1.374616

Rh -0.790217 -0.891154 -0.190737

H -2.285541 0.206941 -2.096543

H -0.900416 1.365152 -1.806314

C -2.722720 -2.258765 -0.355976

H -2.582563 -3.273218 -0.661408

C -3.101867 -1.184290 0.092706

H -1.627995 1.686838 0.553111

C -3.311662 0.230447 0.473118

C -3.500030 0.425060 1.982134

H -3.622066 1.493796 2.188167

H -4.379861 -0.115228 2.333412

H -2.616921 0.058756 2.512929

O -4.435647 0.835155 -0.247366

C -5.651432 0.233618 -0.186900

O -5.878536 -0.779803 0.436052

C -6.660089 0.997560 -1.014102

H -6.367161 0.977248 -2.069358

H -7.643681 0.541361 -0.896319

H -6.691256 2.046807 -0.703959

P 1.281449 0.075228 -0.002506

Cl 0.237930 -3.035546 0.137258

C 2.536713 -0.645532 -1.146970

C 3.911926 -0.618880 -0.874463

C 2.090198 -1.207894 -2.352827

C 4.823027 -1.136005 -1.796658

H 4.276079 -0.208602 0.061738

C 3.004135 -1.715700 -3.276938

H 1.023714 -1.268018 -2.549847

C 4.372142 -1.680880 -3.000428

H 5.885746 -1.117261 -1.570036

H 2.644499 -2.153805 -4.203994

H 5.083545 -2.085977 -3.715206

C 1.338347 1.885717 -0.395103

C 0.831462 2.815302 0.530355

C 1.802839 2.356012 -1.632776

C 0.795819 4.176629 0.227004

H 0.485302 2.476822 1.503094

C 1.762707 3.719252 -1.936241

H 2.204769 1.658493 -2.360605

C 1.259619 4.632623 -1.009191

H 0.408144 4.880765 0.958428

H 2.132163 4.064983 -2.898001

H 1.232103 5.692836 -1.245469

C 2.041842 0.016723 1.683254

C 3.020547 0.947065 2.077948

C 1.648889 -0.979594 2.590730

C 3.594553 0.877678 3.347852

H 3.329302 1.735797 1.398946

C 2.225118 -1.043043 3.861335

H 0.908238 -1.713198 2.292770

C 3.196659 -0.117363 4.243368

H 4.349445 1.604171 3.636937

H 1.910912 -1.821563 4.551199

H 3.641453 -0.168556 5.233703

**8a**

Atom X Y Z

C -2.786353 0.689184 0.020748

C -1.857749 0.888119 -1.017047

C -1.522120 -0.119595 -1.985515

Rh -0.916160 -0.914084 -0.153050

Cl -0.040193 -3.088548 0.094092

H -2.302381 -0.753310 -2.400992

H -0.728994 0.127206 -2.688854

C -2.769369 -1.667196 -0.038182

H -3.093156 -2.701825 0.038528

C -3.532728 -0.576227 0.127769

H -1.296945 1.818060 -1.002631

C -3.066353 1.800415 0.998540

H -3.990081 2.312701 0.701249

H -3.206588 1.416732 2.014318

H -2.259217 2.538116 1.001669

O -4.873433 -0.597125 0.549832

C -5.785943 0.186208 -0.098200

O -5.502051 0.966931 -0.977258

C -7.171247 -0.068504 0.444016

H -7.187138 0.076968 1.528880

H -7.879546 0.607052 -0.036736

H -7.459743 -1.107334 0.251246

P 1.349315 0.073611 0.034762

C 2.664234 -0.695025 -1.007899

C 2.280673 -1.504580 -2.086887

C 4.030863 -0.476056 -0.770406

C 3.245563 -2.071032 -2.923213

H 1.228849 -1.713492 -2.250580

C 4.992215 -1.046736 -1.603933

H 4.347127 0.132531 0.071594

C 4.600863 -1.842706 -2.683989

H 2.934976 -2.702609 -3.751020

H 6.047054 -0.873915 -1.407231

H 5.351765 -2.289858 -3.330065

C 1.452238 1.868553 -0.411377

C 0.839701 2.822457 0.423138

C 2.022613 2.304207 -1.616563

C 0.812194 4.170829 0.068147

H 0.392609 2.510006 1.363538

C 1.985363 3.655034 -1.975866

H 2.506013 1.590819 -2.276271

C 1.383636 4.591758 -1.136265

H 0.344900 4.893253 0.732359

H 2.435081 3.972061 -2.913072

H 1.359851 5.641722 -1.414724

C 2.044578 0.024051 1.749653

C 2.890487 1.022683 2.261327

C 1.729317 -1.079201 2.561087

C 3.406069 0.921075 3.554654

H 3.142828 1.887448 1.655668

C 2.250351 -1.177327 3.852658

H 1.090717 -1.867752 2.174486

C 3.086498 -0.178386 4.353742

H 4.057742 1.702931 3.936127

H 1.998181 -2.037991 4.466278

H 3.487168 -0.255010 5.361105

**9a**

Atom X Y Z

C -2.461004 -2.004468 0.892421

C -1.451326 -1.290071 1.655152

Rh -0.631641 -1.051654 -0.216240

H -1.737637 -0.332464 2.091332

H -0.829688 -1.902311 2.306798

C -1.878566 0.473046 -0.434978

H -1.680668 1.509549 -0.705114

C -3.150986 0.007098 -0.353886

H -2.501507 -3.087900 0.974443

O -4.245833 0.773763 -0.799358

C -5.282650 0.988753 0.065658

O -5.334797 0.529939 1.182524

C -6.328411 1.864630 -0.581186

H -6.708676 1.387297 -1.490457

H -7.145984 2.032496 0.120613

H -5.886169 2.821428 -0.877278

P 1.248619 0.207432 -0.022221

Cl 0.423117 -3.218563 -0.544466

C -3.356594 -1.386422 0.022342

C -4.504178 -2.157989 -0.570864

H -4.670079 -1.897230 -1.621515

H -4.332608 -3.235088 -0.492303

H -5.424349 -1.922887 -0.020380

C 2.224136 -0.156691 1.503442

C 2.366918 -1.494581 1.908847

C 2.862343 0.854718 2.239738

C 3.133907 -1.807260 3.031817

H 1.883352 -2.284582 1.340378

C 3.624863 0.533553 3.364940

H 2.765104 1.893696 1.942574

C 3.761249 -0.796748 3.763868

H 3.237275 -2.845812 3.334374

H 4.110633 1.326301 3.927802

H 4.353354 -1.044562 4.640916

C 0.972218 2.030015 0.049749

C 1.440717 2.904122 -0.941155

C 0.203193 2.551794 1.105514

C 1.156557 4.270926 -0.871455

H 2.032561 2.522805 -1.766571

C -0.071103 3.916707 1.177746

H -0.179808 1.885753 1.873579

C 0.404910 4.780534 0.187229

H 1.528481 4.935498 -1.646634

H -0.661166 4.304177 2.003858

H 0.187498 5.843838 0.240747

C 2.453724 -0.019653 -1.398090

C 3.802322 0.347144 -1.263002

C 2.010267 -0.560176 -2.613199

C 4.685078 0.187831 -2.331037

H 4.165149 0.749472 -0.321686

C 2.895306 -0.716572 -3.681480

H 0.978876 -0.884631 -2.715774

C 4.232248 -0.342066 -3.542168

H 5.727745 0.471174 -2.214909

H 2.541014 -1.144962 -4.614803

H 4.923086 -0.471170 -4.371046

**10a***-endo-re*

Atom X Y Z

C 1.959459 -3.126433 -0.359789

C 1.611332 -2.696065 -1.571940

Rh 0.414386 -1.259646 -0.233357

C 0.877797 -1.284314 1.812667

C 2.320016 -1.422448 2.017507

C 3.256446 -0.584742 1.482119

Cl -1.244251 -3.105026 -0.228724

H 0.495285 -0.321578 2.160463

H 0.289436 -2.101978 2.233615

H 2.664900 -2.239205 2.651874

C 4.698568 -0.631034 1.919099

H 4.999011 0.323408 2.369924

H 5.367947 -0.810156 1.069539

H 4.856746 -1.418784 2.661330

C 1.762574 0.218339 -0.354015

H 1.605660 1.033008 -1.065311

C 2.864730 0.334432 0.425652

O 3.737487 1.447837 0.235866

C 4.706988 1.362686 -0.714109

O 4.936135 0.375104 -1.374963

C 5.454526 2.672720 -0.816844

H 6.258626 2.576734 -1.547213

H 5.865460 2.952217 0.158702

H 4.768244 3.469857 -1.121748

H 1.696619 -3.819609 0.424327

H 0.906806 -2.870604 -2.374559

P -1.339585 0.297627 0.004278

C -2.581231 0.054198 -1.333083

C -0.857836 2.074026 -0.167799

C -2.307716 0.262989 1.576628

C -2.142480 -0.379235 -2.593506

C -3.943594 0.321167 -1.138677

C -0.005307 2.651164 0.791289

C -1.276461 2.857190 -1.253301

C -2.777845 1.439841 2.183720

C -2.617409 -0.978153 2.159523

C -3.047288 -0.534599 -3.643063

H -1.091886 -0.608077 -2.752417

C -4.848817 0.157507 -2.188907

H -4.301165 0.649606 -0.167964

C 0.403996 3.978264 0.672151

H 0.339945 2.064943 1.636941

C -0.858165 4.185212 -1.374718

H -1.933753 2.435617 -2.006008

C -3.535829 1.376321 3.354671

H -2.555009 2.407723 1.748063

C -3.376985 -1.032614 3.328753

H -2.276463 -1.893937 1.685042

C -4.403375 -0.268493 -3.441192

H -2.694921 -0.876378 -4.612265

H -5.903684 0.360206 -2.024752

C -0.020191 4.749326 -0.413240

H 1.059978 4.406726 1.424851

H -1.194965 4.777088 -2.221550

C -3.834856 0.140971 3.931046

H -3.891314 2.295088 3.813571

H -3.609992 -1.998944 3.767767

H -5.110700 -0.398800 -4.255716

H 0.301937 5.782884 -0.507356

H -4.423428 0.093434 4.843512

C 3.087001 -2.674185 -1.251926

H 3.706342 -3.459118 -1.696468

H 3.605765 -1.734012 -1.090510

**10a***-exo-re*

Atom X Y Z

C 1.565086 -3.290700 -0.335274

C 2.248531 -2.380614 -1.052937

Rh 0.446939 -1.344205 -0.156097

C 0.968534 -1.304135 1.867711

C 2.417012 -1.364796 2.110113

C 3.320387 -0.523925 1.539817

Cl -1.380382 -3.012095 0.005924

H 0.538413 -0.354723 2.198212

H 0.416986 -2.138044 2.307426

H 2.783183 -2.128113 2.797166

C 4.766646 -0.501212 1.967364

H 5.044964 0.486609 2.355584

H 5.436432 -0.716469 1.126100

H 4.951027 -1.237931 2.754731

C 1.792601 0.162848 -0.321847

H 1.629112 0.912624 -1.098193

C 2.891516 0.337074 0.443378

O 3.765304 1.427725 0.152786

C 4.682629 1.242243 -0.834126

O 4.838885 0.202792 -1.436345

C 5.474287 2.508951 -1.062514

H 5.950397 2.830886 -0.130664

H 4.803441 3.313995 -1.380650

H 6.231454 2.333005 -1.827336

H 1.579898 -3.894459 0.559241

H 3.159042 -1.805169 -1.117329

P -1.312332 0.320143 0.004549

C -2.613792 0.026477 -1.264301

C -0.772902 2.056207 -0.331674

C -2.219280 0.463766 1.609964

C -2.248901 -0.569362 -2.480730

C -3.945925 0.416809 -1.068710

C 0.071438 2.702092 0.589980

C -1.119823 2.729041 -1.512374

C -2.678955 1.703229 2.088244

C -2.489035 -0.695859 2.356820

C -3.193947 -0.759195 -3.488576

H -1.226301 -0.902896 -2.636094

C -4.892812 0.217328 -2.074952

H -4.249022 0.867853 -0.129115

C 0.540452 3.991213 0.342556

H 0.359948 2.199263 1.507642

C -0.641419 4.018391 -1.761871

H -1.768805 2.252401 -2.239160

C -3.385025 1.781031 3.290084

H -2.486159 2.611584 1.527894

C -3.195545 -0.609967 3.557393

H -2.164113 -1.662297 1.984624

C -4.518986 -0.367702 -3.285956

H -2.898049 -1.227259 -4.423271

H -5.924144 0.516603 -1.908701

C 0.186449 4.653047 -0.836082

H 1.188012 4.474961 1.068576

H -0.924151 4.525548 -2.680451

C -3.642199 0.625501 4.029282

H -3.732557 2.747380 3.645814

H -3.396612 -1.515949 4.122698

H -5.258602 -0.525204 -4.066329

H 0.554612 5.656913 -1.030102

H -4.189645 0.687967 4.966091

C 1.491650 -3.438052 -1.827596

H 2.082573 -4.228544 -2.299151

H 0.562334 -3.210353 -2.350482

**11a***-endo-re*

Atom X Y Z

C 0.309016 -3.366332 -1.255820

C 1.991386 -2.684989 0.824560

C 1.992936 -1.502241 1.662068

C 2.467619 -0.258697 1.239978

C -0.564040 -2.426701 -1.761746

Rh 0.398818 -1.604100 -0.027211

Cl -1.173051 -2.798380 1.509117

H 2.807054 -2.798694 0.113468

H 1.668046 -3.603366 1.307226

H -1.634361 -2.324001 -1.880217

H 1.631793 -1.591336 2.683526

C 2.716820 -0.048731 -0.184883

C 1.834384 -0.606348 -1.034386

H 1.896187 -0.427509 -2.106357

C 2.699434 0.858816 2.216704

H 3.767017 0.889297 2.470498

H 2.430056 1.830424 1.789733

H 2.137195 0.701638 3.141020

O 3.708783 0.853761 -0.613144

C 4.989015 0.663855 -0.179604

O 5.311582 -0.187361 0.616993

C 5.916745 1.660779 -0.830749

H 6.918166 1.552096 -0.413015

H 5.946435 1.487913 -1.911982

H 5.550029 2.680151 -0.674322

H 0.240620 -4.324970 -0.760189

P -1.114260 0.399003 -0.044128

C -0.378006 1.824625 -0.958565

C 0.060500 2.993206 -0.318864

C -0.147435 1.688758 -2.339909

C 0.695868 4.005795 -1.043544

H -0.100468 3.122713 0.746237

C 0.475352 2.704959 -3.063078

H -0.463209 0.784208 -2.853136

C 0.900591 3.868164 -2.415987

H 1.024278 4.905893 -0.530321

H 0.633168 2.585401 -4.131710

H 1.389724 4.658728 -2.978617

C -2.768602 0.158977 -0.843922

C -3.510453 -0.980015 -0.482749

C -3.326780 1.077414 -1.746318

C -4.781882 -1.188770 -1.018972

H -3.089337 -1.698452 0.216392

C -4.598630 0.859335 -2.281883

H -2.775440 1.965141 -2.037730

C -5.328979 -0.273238 -1.920765

H -5.343098 -2.073405 -0.730006

H -5.016361 1.579833 -2.980438

H -6.318111 -0.441189 -2.338678

C -1.597112 1.111538 1.593105

C -2.481048 2.201916 1.673836

C -1.107971 0.545215 2.777486

C -2.842668 2.729532 2.912261

H -2.893110 2.636104 0.767405

C -1.476236 1.072723 4.018478

H -0.477032 -0.334466 2.727813

C -2.337359 2.167120 4.088281

H -3.525350 3.573824 2.959956

H -1.099395 0.614933 4.929150

H -2.625617 2.574658 5.053682

C 0.558672 -2.896224 -2.659974

H 1.409433 -2.262554 -2.902192

H 0.290916 -3.584970 -3.466793

**11a***-exo-re*

Atom X Y Z

C 0.500309 -3.171949 -1.510993

C 1.943184 -2.712529 0.823498

C 1.997325 -1.505520 1.627235

C 2.510317 -0.292951 1.162781

C -0.548605 -2.331597 -1.831536

Rh 0.386812 -1.603791 -0.048192

Cl -1.179515 -2.654529 1.616896

H 2.759776 -2.872772 0.122092

H 1.584547 -3.606019 1.326574

H -0.819465 -1.666355 -2.641934

H 1.647609 -1.552041 2.655319

C 2.737987 -0.125591 -0.273071

C 1.825586 -0.675356 -1.095612

H 1.884475 -0.562266 -2.178358

C 2.802208 0.840031 2.105310

H 3.877052 0.845660 2.328687

H 2.547881 1.808924 1.663105

H 2.263471 0.719665 3.049046

O 3.755130 0.728114 -0.739485

C 5.036313 0.495534 -0.328327

O 5.342486 -0.359892 0.469900

C 5.986318 1.453546 -1.005045

H 5.972415 1.288616 -2.087709

H 5.671286 2.487131 -0.829558

H 6.994757 1.297421 -0.620622

H 1.454984 -3.460988 -1.931489

P -1.088195 0.440100 -0.040789

C -0.299461 1.888236 -0.877454

C 0.153691 3.009031 -0.165529

C -0.041170 1.824138 -2.258614

C 0.832423 4.041821 -0.818433

H -0.032139 3.084636 0.900729

C 0.625820 2.860172 -2.911073

H -0.376278 0.963886 -2.831782

C 1.066841 3.973644 -2.191582

H 1.169658 4.904385 -0.249464

H 0.803384 2.795892 -3.981374

H 1.588780 4.780442 -2.698948

C -2.700810 0.231598 -0.924212

C -3.425472 -0.949348 -0.687544

C -3.245949 1.209950 -1.770247

C -4.671264 -1.138600 -1.286414

H -3.012216 -1.711667 -0.033019

C -4.490340 1.009631 -2.373189

H -2.703052 2.128796 -1.966962

C -5.205697 -0.163790 -2.132185

H -5.222232 -2.055082 -1.092958

H -4.898341 1.774695 -3.028766

H -6.173980 -0.318288 -2.601138

C -1.619736 1.130749 1.590965

C -2.572571 2.161897 1.658034

C -1.075636 0.630984 2.781200

C -2.954445 2.693801 2.888686

H -3.023240 2.546503 0.747859

C -1.460971 1.164880 4.013921

H -0.387972 -0.205162 2.744335

C -2.396261 2.197636 4.070229

H -3.693070 3.490195 2.925531

H -1.040424 0.758040 4.929618

H -2.699632 2.608107 5.029758

C -0.883771 -3.765608 -1.497702

H -1.364999 -4.013607 -0.557248

H -1.142822 -4.415065 -2.339979

**12a***-endo-re*

Atom X Y Z

C 1.639308 -2.559078 -1.187707

C 2.158745 -1.076880 -1.733837

Rh 0.456550 -1.396172 -0.104470

C 0.497068 -1.346605 2.050341

C 1.763469 -1.784061 1.589182

C 2.937795 -0.872370 1.523174

Cl -1.249089 -3.231433 -0.158934

H 0.390182 -0.339695 2.445487

H -0.214299 -2.075324 2.428711

H 1.983636 -2.849057 1.671773

C 3.987252 -0.981428 2.596655

H 3.571801 -0.743953 3.584562

H 4.827801 -0.309334 2.406900

H 4.370732 -2.009204 2.654444

C 1.997307 -0.081661 -0.628537

H 1.681257 0.901611 -0.980203

C 2.968484 -0.007861 0.491307

O 3.881417 1.064686 0.513768

C 4.785199 1.192510 -0.504460

O 4.952586 0.365016 -1.367881

C 5.529890 2.499999 -0.377716

H 6.312165 2.547262 -1.135988

H 5.966774 2.595438 0.621327

H 4.834410 3.335899 -0.510820

H 1.084940 -3.301394 -1.759138

H 1.919761 -0.778894 -2.749545

P -1.331934 0.291625 0.012410

C -2.505179 0.176882 -1.409210

C -0.683157 2.021956 -0.101496

C -2.461016 0.332639 1.475964

C -2.175785 -0.609639 -2.521749

C -3.723391 0.876090 -1.405573

C 0.111383 2.522861 0.946265

C -0.868103 2.821708 -1.239342

C -2.844683 1.518967 2.120418

C -2.980532 -0.892842 1.929890

C -3.039089 -0.682963 -3.617257

H -1.259490 -1.191490 -2.517636

C -4.585047 0.799924 -2.499413

H -4.003188 1.474479 -0.543486

C 0.685310 3.791001 0.866086

H 0.280391 1.920803 1.834465

C -0.283936 4.089097 -1.322885

H -1.474066 2.460941 -2.063677

C -3.726386 1.480793 3.203734

H -2.459977 2.475580 1.782203

C -3.864408 -0.921868 3.008838

H -2.692858 -1.815729 1.431626

C -4.242889 0.021833 -3.608639

H -2.775524 -1.305358 -4.467947

H -5.526444 1.342711 -2.482505

C 0.490019 4.579190 -0.271419

H 1.288248 4.160861 1.691075

H -0.441915 4.693228 -2.212417

C -4.237201 0.261751 3.650594

H -4.013524 2.407221 3.694506

H -4.260330 -1.875259 3.348453

H -4.918269 -0.041955 -4.457715

H 0.938868 5.566762 -0.335771

H -4.923009 0.234052 4.493356

C 3.048369 -2.275161 -1.487206

H 3.471764 -2.775068 -2.357106

H 3.764152 -2.123727 -0.681147

**12a***-exo-re*

Atom X Y Z

C -2.606958 -2.658498 0.101702

C -3.641457 -1.720890 0.651456

Rh -0.996868 -1.389699 -0.032903

C -1.324265 -1.424661 -2.059247

C -2.485645 -0.580785 -2.456678

C -3.046625 0.314977 -1.634465

Cl 0.371471 -3.304227 -0.013621

H -0.386164 -1.058951 -2.495665

H -1.449963 -2.470574 -2.358222

H -2.882624 -0.706960 -3.467099

C -4.169448 1.243874 -2.033381

H -3.854605 2.293981 -1.982525

H -5.033053 1.140052 -1.366577

H -4.490617 1.041013 -3.059611

C -2.865037 -0.448763 0.807884

H -2.650917 -0.082989 1.813699

C -2.546582 0.404436 -0.233269

O -2.082005 1.693866 0.120508

C -2.925942 2.506846 0.844637

O -4.036795 2.184781 1.190705

C -2.265092 3.831799 1.119957

H -2.019422 4.331030 0.178015

H -1.320317 3.680340 1.650628

H -2.938925 4.453246 1.710841

H -2.795778 -3.401407 -0.668504

H -4.692905 -1.677230 0.371243

P 1.159038 0.081161 -0.001182

C 2.039981 -0.343757 1.566405

C 1.134299 1.937231 0.029431

C 2.423420 -0.198086 -1.336103

C 1.259549 -0.592433 2.706832

C 3.435065 -0.433479 1.671360

C 0.653469 2.613122 -1.105611

C 1.610651 2.694582 1.108525

C 3.381921 0.779442 -1.660690

C 2.437423 -1.412225 -2.042128

C 1.856713 -0.908544 3.927200

H 0.174333 -0.545921 2.639804

C 4.033044 -0.758652 2.890609

H 4.056527 -0.258589 0.798955

C 0.679611 4.004955 -1.172058

H 0.270461 2.046733 -1.950314

C 1.623264 4.092610 1.047501

H 1.993296 2.197106 1.993756

C 4.324639 0.548456 -2.663255

H 3.394210 1.727606 -1.133849

C 3.381471 -1.637659 -3.046767

H 1.728496 -2.191476 -1.786790

C 3.247311 -0.994112 4.020240

H 1.236675 -1.101473 4.798440

H 5.115483 -0.831387 2.955084

C 1.167904 4.751136 -0.094527

H 0.320472 4.507965 -2.066313

H 2.005785 4.662315 1.890554

C 4.324991 -0.660086 -3.362603

H 5.057256 1.316487 -2.897612

H 3.375093 -2.584349 -3.580410

H 3.715442 -1.250805 4.966670

H 1.192010 5.836393 -0.147040

H 5.056720 -0.837398 -4.146481

C -3.106390 -2.857837 1.499440

H -3.779099 -3.698233 1.663775

H -2.476713 -2.632794 2.361389

**13a***-endo-re*

Atom X Y Z

C 1.491976 -2.902158 -1.648063

C 2.845579 -2.451193 -1.088440

C 2.755470 -1.956431 0.340118

C 3.043864 -0.685591 0.792708

C 0.298671 -2.108854 -1.203677

Rh 0.651345 -0.770834 0.286454

Cl -0.102248 -2.179478 2.161465

H 3.284685 -1.672016 -1.716914

H 3.543972 -3.298705 -1.097511

H -0.631738 -2.640982 -1.017302

H 2.662150 -2.728658 1.102066

C 2.948971 0.467212 -0.140264

C 1.742826 0.583070 -0.721905

H 1.471197 1.389529 -1.401419

C 3.408431 -0.435094 2.238411

H 4.502199 -0.413096 2.334349

H 3.019565 0.522083 2.602035

H 3.023261 -1.235025 2.876933

O 3.946789 1.452881 -0.208555

C 5.258363 1.077381 -0.283944

O 5.635397 -0.070852 -0.308991

C 6.146199 2.298354 -0.326183

H 7.187635 1.986172 -0.410510

H 5.874826 2.932984 -1.176075

H 6.008477 2.894410 0.582255

H 1.361071 -3.977315 -1.760861

P -1.386670 0.252712 -0.022002

C -1.451573 1.532180 -1.351647

C -1.888864 2.843698 -1.116239

C -1.062745 1.167884 -2.652216

C -1.931953 3.772749 -2.159366

H -2.199081 3.144072 -0.121065

C -1.113137 2.095908 -3.691825

H -0.719103 0.157133 -2.846888

C -1.545040 3.402292 -3.447337

H -2.271844 4.785722 -1.961115

H -0.809756 1.799532 -4.692370

H -1.579038 4.126236 -4.257021

C -2.873066 -0.785083 -0.363714

C -3.011886 -2.030501 0.273350

C -3.885948 -0.342358 -1.230943

C -4.143605 -2.813463 0.039260

H -2.239329 -2.376293 0.954829

C -5.013276 -1.132754 -1.460484

H -3.800217 0.617100 -1.729342

C -5.143767 -2.370375 -0.828264

H -4.237913 -3.774486 0.537540

H -5.787785 -0.778718 -2.135665

H -6.020847 -2.985744 -1.010912

C -1.769153 1.180764 1.522032

C -2.999019 1.068638 2.183252

C -0.770217 2.005187 2.068741

C -3.225775 1.769424 3.370021

H -3.777399 0.429833 1.779649

C -1.003132 2.708464 3.249723

H 0.191151 2.099868 1.568823

C -2.231478 2.589027 3.904775

H -4.181811 1.669868 3.876730

H -0.222709 3.342125 3.662153

H -2.409980 3.129943 4.830091

C 0.768752 -1.997663 -2.631072

H 1.296982 -1.102580 -2.948823

H 0.158819 -2.455527 -3.409439

**13a***-exo-re*

Atom X Y Z

C 1.634058 -2.615478 -1.904558

C 2.942399 -2.322451 -1.192861

C 2.776030 -1.878319 0.246828

C 3.052360 -0.615413 0.736451

C 0.425521 -1.872639 -1.413089

Rh 0.690005 -0.703195 0.231461

Cl -0.030077 -2.035104 2.191969

H 3.472727 -1.541007 -1.747248

H 3.585937 -3.214592 -1.187722

H -0.294311 -1.525487 -2.150682

H 2.693177 -2.668834 0.990466

C 2.970595 0.553957 -0.176808

C 1.763023 0.681193 -0.751531

H 1.483232 1.493799 -1.419043

C 3.406897 -0.395696 2.188604

H 4.500169 -0.400001 2.293683

H 3.036295 0.564894 2.561849

H 2.995605 -1.195345 2.810606

O 3.969621 1.538415 -0.223926

C 5.279204 1.157175 -0.300607

O 5.649405 0.006855 -0.338396

C 6.174287 2.372967 -0.327381

H 5.914068 3.013461 -1.176438

H 6.031863 2.965034 0.582838

H 7.214548 2.055122 -0.404688

H 1.735223 -2.780507 -2.977610

P -1.385378 0.267770 -0.042204

C -1.497290 1.613676 -1.306553

C -2.126255 2.838041 -1.033463

C -0.960497 1.401700 -2.587981

C -2.215447 3.825393 -2.018095

H -2.549413 3.023775 -0.051999

C -1.055122 2.386823 -3.570610

H -0.457324 0.468240 -2.816341

C -1.681728 3.602882 -3.287637

H -2.704446 4.768343 -1.788667

H -0.632746 2.205668 -4.555287

H -1.750618 4.372180 -4.051953

C -2.789053 -0.845885 -0.475372

C -2.830707 -2.140816 0.068640

C -3.827970 -0.420734 -1.320055

C -3.900008 -2.987461 -0.227156

H -2.033682 -2.473715 0.726724

C -4.891243 -1.276153 -1.614197

H -3.811470 0.574839 -1.751114

C -4.929277 -2.560488 -1.068690

H -3.923010 -3.985856 0.201098

H -5.687697 -0.936339 -2.270902

H -5.757130 -3.225814 -1.299703

C -1.864801 1.103064 1.525632

C -3.126421 0.940275 2.111843

C -0.915497 1.924243 2.156912

C -3.433520 1.591132 3.309107

H -3.866709 0.301796 1.641182

C -1.227569 2.576462 3.348402

H 0.070526 2.052592 1.716442

C -2.487873 2.408988 3.928103

H -4.413402 1.453556 3.758118

H -0.484291 3.207200 3.828334

H -2.728756 2.910653 4.861393

C 0.517826 -3.362212 -1.193426

H 0.695405 -3.727256 -0.186655

H -0.135751 -3.999888 -1.786973

**14a**

Atom X Y Z

C 3.159362 -2.516093 -1.476896

C 3.106786 -2.953740 -0.025328

C 2.694897 -1.995137 1.072864

C 2.783113 -0.596456 1.223387

C 2.495706 -1.261211 -1.985920

Rh 0.739014 -0.990998 0.555045

Cl -0.429708 -2.555768 1.931609

H 4.092153 -3.361253 0.259354

H 2.413554 -3.802246 0.044927

H 1.978278 -1.353787 -2.938070

H 2.668439 -2.515294 2.030155

C 2.372213 0.262430 0.139645

C 1.885411 -0.223408 -1.105597

H 1.403079 0.566796 -1.677845

C 3.064244 0.011494 2.579410

H 4.122292 0.302871 2.633330

H 2.459690 0.901545 2.768751

H 2.876054 -0.720087 3.369678

O 2.300606 1.648444 0.390816

C 3.395363 2.380525 0.011393

O 4.405288 1.887469 -0.431061

C 3.144479 3.850526 0.236624

H 4.021700 4.419730 -0.072720

H 2.265313 4.168277 -0.333369

H 2.932750 4.037820 1.294684

H 3.066673 -3.364093 -2.153878

P -1.282830 0.057322 -0.077755

C -1.079448 1.641319 -1.018894

C -1.114026 2.881249 -0.363657

C -0.784362 1.618089 -2.393783

C -0.866039 4.064584 -1.063756

H -1.342923 2.926396 0.696186

C -0.538230 2.800804 -3.092677

H -0.764660 0.672067 -2.927839

C -0.576804 4.029290 -2.429101

H -0.910336 5.016059 -0.539909

H -0.319927 2.760936 -4.156588

H -0.389777 4.950850 -2.973740

C -2.421828 -0.925839 -1.154842

C -2.363519 -2.327679 -1.091838

C -3.358024 -0.323756 -2.013988

C -3.223874 -3.105470 -1.869685

H -1.658193 -2.805571 -0.419342

C -4.213969 -1.105725 -2.791026

H -3.414719 0.757797 -2.088705

C -4.148302 -2.498852 -2.721008

H -3.167528 -4.188859 -1.807082

H -4.930348 -0.624428 -3.451752

H -4.814039 -3.106915 -3.327905

C -2.315505 0.568506 1.366343

C -3.692781 0.812573 1.256647

C -1.688182 0.753824 2.607378

C -4.424125 1.242900 2.364116

H -4.201843 0.654824 0.311167

C -2.420573 1.191837 3.712276

H -0.630585 0.529673 2.707728

C -3.789173 1.437156 3.592899

H -5.491796 1.421442 2.267037

H -1.922740 1.324280 4.669073

H -4.361484 1.769055 4.455175

C 4.017093 -1.402134 -2.011084

H 4.559011 -0.766102 -1.317062

H 4.507183 -1.545593 -2.970950

**16a**

Atom X Y Z

C 1.699446 -2.242549 -2.083841

C 2.790796 -2.801254 -1.171952

C 2.415355 -2.343679 0.255475

C 3.174682 -1.210079 0.821667

C 1.559766 -0.734571 -1.891446

Rh 0.603846 -1.207255 0.095494

Cl -0.354570 -2.485554 1.847025

H 3.785233 -2.449692 -1.472580

H 2.798659 -3.895299 -1.236723

H 0.891980 -0.257835 -2.606492

H 2.299753 -3.166116 0.961840

C 3.051406 -0.032932 0.142586

C 2.105039 0.150528 -0.963645

H 1.821457 1.191409 -1.111733

C 3.958043 -1.360490 2.094147

H 4.796848 -2.053707 1.945657

H 4.350646 -0.408237 2.458553

H 3.317092 -1.801499 2.868089

O 3.672193 1.127188 0.624382

C 4.527814 1.789501 -0.219957

O 4.783396 1.416341 -1.338436

C 5.077646 3.020142 0.458246

H 4.260725 3.697449 0.728669

H 5.590784 2.742696 1.384803

H 5.771091 3.524775 -0.215118

H 1.825591 -2.512418 -3.144434

P -1.365698 0.239714 0.020768

C -1.137114 1.717021 -1.072510

C -0.802648 2.974669 -0.545549

C -1.168473 1.565213 -2.471123

C -0.512536 4.048317 -1.391290

H -0.776758 3.121231 0.529784

C -0.883991 2.640133 -3.314205

H -1.439091 0.606560 -2.905446

C -0.552418 3.886140 -2.776690

H -0.262445 5.015226 -0.962388

H -0.923407 2.503235 -4.391646

H -0.329967 4.722970 -3.433000

C -2.924553 -0.528021 -0.609388

C -3.142040 -1.891516 -0.350861

C -3.907056 0.198271 -1.303946

C -4.319618 -2.509615 -0.776053

H -2.393938 -2.458077 0.195153

C -5.080447 -0.426096 -1.730509

H -3.755899 1.250896 -1.522248

C -5.289104 -1.781519 -1.467719

H -4.475052 -3.564485 -0.566237

H -5.830337 0.148426 -2.268169

H -6.202511 -2.266818 -1.801564

C -1.848790 0.990482 1.638680

C -3.108278 1.571836 1.853896

C -0.909569 1.000633 2.680299

C -3.415179 2.159936 3.080765

H -3.855809 1.557649 1.066660

C -1.215923 1.594982 3.906460

H 0.054411 0.520684 2.539783

C -2.468436 2.175345 4.108394

H -4.395820 2.602170 3.235721

H -0.480122 1.588572 4.705979

H -2.710975 2.630154 5.065212

C 0.357572 -2.613431 -1.448279

H 0.291485 -3.600696 -0.987061

H -0.516613 -2.396649 -2.066717

**17a**

Atom X Y Z

C -1.794439 0.617333 0.630195

C -2.678673 2.784757 0.142196

Rh -0.629262 -0.501157 -0.655617

C -0.991143 0.948975 -2.190650

C -2.010839 -0.061237 -2.324791

C -2.886412 -0.452785 -1.307677

Cl -0.814648 -2.593434 0.572450

H -1.230830 1.922727 -1.778365

H -0.285252 0.996338 -3.019968

H -1.929307 -0.751206 -3.165826

C -3.710425 -1.703579 -1.469260

H -4.764302 -1.452361 -1.632146

H -3.635407 -2.335272 -0.578904

H -3.355641 -2.287264 -2.324028

C -3.530608 1.918656 -0.415643

H -4.403686 2.159554 -1.007404

C -3.141957 0.487734 -0.104362

O -4.069983 -0.132811 0.846459

C -5.405760 -0.105129 0.632922

O -5.942639 0.353926 -0.353005

C -6.131615 -0.740264 1.798268

H -5.898966 -0.204676 2.724461

H -5.798167 -1.775082 1.928270

H -7.206141 -0.714959 1.612899

H -1.791240 0.013559 1.538176

H -2.766479 3.866292 0.068897

P 1.526854 0.012910 0.063338

C 2.091507 -0.518934 1.737079

C 2.057952 1.783473 -0.024289

C 2.669860 -0.887112 -1.077397

C 1.157616 -0.704868 2.765345

C 3.455392 -0.720925 2.007276

C 1.951449 2.474905 -1.244549

C 2.507551 2.483186 1.105742

C 3.774943 -0.292586 -1.704975

C 2.394957 -2.246971 -1.316609

C 1.580900 -1.071874 4.044642

H 0.099915 -0.595791 2.559209

C 3.874603 -1.091882 3.284473

H 4.191046 -0.594573 1.218181

C 2.299082 3.822129 -1.333758

H 1.597793 1.954834 -2.128499

C 2.842017 3.837290 1.017327

H 2.599015 1.974130 2.058962

C 4.585730 -1.040912 -2.561838

H 4.007956 0.752176 -1.527152

C 3.210934 -2.988209 -2.172102

H 1.550576 -2.723776 -0.823713

C 2.937419 -1.265504 4.306341

H 0.845478 -1.222743 4.829992

H 4.931901 -1.249871 3.479949

C 2.742104 4.509474 -0.200338

H 2.218429 4.336370 -2.287732

H 3.184850 4.362677 1.904690

C 4.304830 -2.387389 -2.799295

H 5.439548 -0.568583 -3.040657

H 2.988040 -4.037184 -2.347317

H 3.264452 -1.559685 5.300130

H 3.005708 5.561445 -0.268069

H 4.936787 -2.966161 -3.467615

C -1.541740 2.114285 0.874191

H -0.566034 2.453833 0.506722

H -1.557460 2.350899 1.949478

**18a**

Atom X Y Z

C -3.418834 0.576892 1.493845

C -5.590160 -0.298968 0.911089

Rh -0.749177 0.729079 -0.483898

C -2.869894 2.016467 1.332296

C -2.452215 2.106651 -0.118170

C -2.895109 1.034315 -0.893777

Cl 0.135971 2.396284 -1.863063

H -3.658774 2.760717 1.522585

H -2.053723 2.243680 2.027181

H -2.176991 3.062855 -0.552828

C -3.235209 1.070454 -2.362623

H -4.313672 1.241603 -2.498242

H -2.989055 0.128576 -2.858766

H -2.692278 1.881466 -2.854425

C -4.809471 -0.577185 -0.138061

H -5.118416 -1.078760 -1.044595

C -3.417272 -0.047968 0.058216

O -2.348631 -1.119076 0.109641

C -2.335236 -2.104606 -0.845395

O -3.087043 -2.134012 -1.792007

C -1.283284 -3.148269 -0.560391

H -1.161285 -3.321064 0.510402

H -0.319838 -2.810442 -0.954443

H -1.563329 -4.070065 -1.074019

H -2.750038 -0.014771 2.124188

H -6.645668 -0.553035 0.972658

P 1.423259 0.019801 0.071180

C 2.562700 -0.395502 -1.317385

C 1.386234 -1.514240 1.107181

C 2.391652 1.184663 1.137669

C 1.997583 -0.703393 -2.564419

C 3.958456 -0.430935 -1.173379

C 0.533656 -1.523565 2.225462

C 2.130875 -2.666690 0.818117

C 3.243837 0.717672 2.152473

C 2.289145 2.568097 0.917429

C 2.810916 -1.063213 -3.640215

H 0.921403 -0.630458 -2.695331

C 4.769887 -0.786749 -2.251719

H 4.414145 -0.171915 -0.222238

C 0.434534 -2.653173 3.036813

H -0.054761 -0.639724 2.458681

C 2.024654 -3.801613 1.627636

H 2.796600 -2.681438 -0.038776

C 3.979757 1.615310 2.928694

H 3.331991 -0.346969 2.344370

C 3.030432 3.459783 1.694243

H 1.632474 2.939665 0.137501

C 4.197431 -1.107357 -3.485010

H 2.360607 -1.291966 -4.602263

H 5.849648 -0.807202 -2.129543

C 1.178771 -3.798442 2.737345

H -0.225722 -2.641410 3.900066

H 2.607748 -4.687220 1.389034

C 3.874832 2.988407 2.701429

H 4.632875 1.238424 3.711573

H 2.941652 4.527473 1.511793

H 4.831527 -1.379263 -4.324749

H 1.098967 -4.681044 3.366243

H 4.446045 3.686693 3.307631

C -4.877148 0.441555 2.012025

H -5.337476 1.423696 2.188432

H -4.935807 -0.096178 2.968091

**19a**

Atom X Y Z

C -2.455143 -2.930705 -0.161917

C -2.307764 -2.099155 1.147571

Rh -0.022387 -1.498137 0.415368

C -0.650318 -1.882560 -1.500547

C -2.174607 -2.091212 -1.425752

C -2.864475 -0.738526 -1.380800

Cl 1.507133 -3.364802 0.677841

H -0.371817 -1.102712 -2.215462

H -0.122352 -2.810910 -1.748796

H -2.518086 -2.646495 -2.311893

C -3.649432 -0.253906 -2.563936

H -3.047927 -0.295136 -3.483115

H -3.990822 0.775435 -2.426976

H -4.531000 -0.886278 -2.740690

C -1.908064 -0.666249 0.875752

H -1.872873 -0.028412 1.761974

C -2.659623 -0.058272 -0.240831

O -3.105360 1.268957 -0.102894

C -4.137317 1.504785 0.758060

O -4.729982 0.637353 1.356161

C -4.429752 2.984868 0.829867

H -5.182238 3.170326 1.597170

H -4.801423 3.335442 -0.139507

H -3.514044 3.542856 1.046744

H -2.029311 -3.931228 -0.167804

H -1.885302 -2.569176 2.033866

P 1.198852 0.312847 -0.013826

C 2.158493 0.668113 1.521464

C 0.352404 1.905422 -0.413521

C 2.456934 0.159179 -1.353568

C 1.514402 0.525783 2.762974

C 3.511156 1.036537 1.495723

C -0.281071 2.048673 -1.660160

C 0.302248 2.971789 0.495871

C 2.933451 1.284103 -2.049748

C 2.989588 -1.106428 -1.648366

C 2.205931 0.757461 3.952093

H 0.468211 0.231862 2.801075

C 4.201110 1.265166 2.687596

H 4.028648 1.137883 0.547584

C -0.935992 3.233269 -1.991497

H -0.252446 1.237357 -2.380905

C -0.358802 4.157002 0.162983

H 0.790503 2.887125 1.461005

C 3.921853 1.142749 -3.024635

H 2.534710 2.270311 -1.835408

C 3.978369 -1.239221 -2.625467

H 2.638434 -1.980403 -1.106703

C 3.552058 1.126935 3.915657

H 1.695660 0.640020 4.904052

H 5.250063 1.546737 2.654359

C -0.976021 4.292134 -1.081071

H -1.414867 3.329284 -2.962165

H -0.376705 4.978516 0.874511

C 4.444072 -0.119441 -3.316203

H 4.281074 2.019755 -3.556548

H 4.381729 -2.224071 -2.844281

H 4.094154 1.300272 4.841204

H -1.481689 5.217872 -1.342590

H 5.211154 -0.227867 -4.078529

C -3.646677 -2.676609 0.688469

H -4.030987 -3.503882 1.278879

H -4.386414 -1.949959 0.368935

**20a**

Atom X Y Z

C -2.298034 -0.219638 0.889138

C -1.631504 -1.477374 0.361092

C -2.084822 -0.029805 -1.473629

C -1.963611 0.857046 -0.200093

C -0.461311 1.152717 -0.214278

H -1.829868 0.494149 -2.401846

H -3.064924 -0.511416 -1.570568

H -2.600484 1.744271 -0.147934

C 0.154489 2.409124 0.308669

H -0.362519 3.292010 -0.089397

H 1.213711 2.484469 0.048821

H 0.082410 2.455379 1.403660

C -0.975098 -1.000241 -0.980594

H -0.668675 -1.809645 -1.646679

C 0.101737 0.027835 -0.682304

O 1.440227 -0.286339 -0.883209

C 2.346825 -0.216528 0.139992

O 2.082329 0.118845 1.269550

C 3.710856 -0.626954 -0.361268

H 4.433466 -0.555837 0.452174

H 4.016838 0.017205 -1.192098

H 3.676524 -1.653556 -0.740915

H -3.317069 -0.235131 1.266791

H -2.151823 -2.431339 0.339828

C -1.207036 -0.891357 1.685669

H -1.531165 -1.435076 2.570847

H -0.231815 -0.424476 1.782227

**TS1a**

Imaginary frequency: -336.6645 cm-1

Atom X Y Z

C -2.023180 0.876135 -0.096576

C -1.665845 0.492982 -1.418461

Rh -0.846744 -0.928632 -0.092467

H -2.383085 -0.005446 -2.068276

H -0.938999 1.104239 -1.947304

C -2.713503 -1.942058 -0.148812

H -2.874145 -2.972148 -0.431225

C -3.495803 -0.989908 0.212751

H -1.560189 1.752167 0.350742

O -5.270951 -0.976822 0.045724

C -5.488762 0.225114 -0.244391

O -4.554671 1.117015 -0.075275

C -6.784456 0.682974 -0.838124

H -7.590827 0.015085 -0.530184

H -6.997588 1.713013 -0.545305

H -6.700376 0.645020 -1.931034

C -3.308955 0.440850 0.544774

C -3.397907 0.784716 2.031166

H -4.318771 0.395355 2.475683

H -2.544731 0.332106 2.543430

H -3.364889 1.870428 2.169990

Cl 0.124734 -3.080369 0.324733

P 1.284951 0.067258 -0.007760

C 1.331116 1.860139 -0.483741

C 1.722582 2.270745 -1.767302

C 0.863645 2.835161 0.415944

C 1.653903 3.616222 -2.138784

H 2.092401 1.539123 -2.478771

C 0.801708 4.179152 0.046458

H 0.567776 2.544755 1.420512

C 1.195121 4.574370 -1.234435

H 1.967214 3.913112 -3.136306

H 0.448206 4.917833 0.761301

H 1.146974 5.621058 -1.522566

C 2.523718 -0.687813 -1.154796

C 3.904464 -0.474862 -1.020877

C 2.056695 -1.474126 -2.218417

C 4.797616 -1.027686 -1.938837

H 4.287949 0.113185 -0.192947

C 2.952206 -2.021204 -3.139966

H 0.993501 -1.676075 -2.303408

C 4.322781 -1.798766 -3.002677

H 5.864888 -0.859663 -1.819841

H 2.576673 -2.634034 -3.955046

H 5.020259 -2.231388 -3.715325

C 2.123241 0.075246 1.645921

C 1.813972 -0.937956 2.567997

C 3.073362 1.048518 2.003547

C 2.443909 -0.975315 3.813939

H 1.095322 -1.705030 2.298791

C 3.698598 1.007889 3.250736

H 3.320360 1.849832 1.314162

C 3.384772 -0.004483 4.159719

H 2.195096 -1.768796 4.513654

H 4.429722 1.769193 3.510646

H 3.870993 -0.034836 5.131434

**TS2a***-endo-re*

Imaginary frequency: -219.1133 cm-1

Atom X Y Z

C 1.507005 -2.956951 -1.348335

C 2.063910 -1.719486 -1.750214

Rh 0.373886 -1.461354 -0.390071

C 0.900815 -1.749085 1.632475

C 2.330917 -1.834357 1.867187

C 3.242703 -0.892632 1.463773

Cl -1.422488 -3.110618 -0.326814

H 0.454666 -0.846297 2.054240

H 0.346984 -2.636791 1.937969

H 2.705178 -2.705196 2.405993

C 4.661881 -0.892820 1.980410

H 4.860207 0.009976 2.570678

H 5.393503 -0.905678 1.163233

H 4.841461 -1.761009 2.621175

C 1.843110 -0.036641 -0.427052

H 1.717052 0.796653 -1.115750

C 2.866131 0.084402 0.470438

O 3.693258 1.233185 0.454328

C 4.382922 1.566620 -0.679145

O 4.395983 0.910881 -1.692794

C 5.110637 2.873641 -0.467827

H 5.745232 3.079981 -1.330380

H 5.712953 2.835505 0.445031

H 4.380667 3.681501 -0.345690

H 0.915901 -3.721717 -1.837205

H 2.102058 -1.115595 -2.647884

P -1.326692 0.286841 0.033751

C -2.715388 0.229602 -1.178960

C -0.676917 2.008950 -0.147866

C -2.168474 0.310953 1.681347

C -2.523029 -0.416887 -2.408394

C -3.949027 0.846652 -0.919711

C 0.227801 2.509183 0.806817

C -0.972151 2.799424 -1.268900

C -2.489412 1.499721 2.357777

C -2.523838 -0.919574 2.261600

C -3.537479 -0.432865 -3.367092

H -1.587393 -0.931130 -2.606301

C -4.964340 0.824091 -1.876226

H -4.120942 1.338824 0.032743

C 0.801745 3.771192 0.653313

H 0.479850 1.915091 1.680076

C -0.388406 4.059248 -1.426082

H -1.665726 2.436133 -2.019923

C -3.146226 1.458513 3.589515

H -2.226268 2.460945 1.928832

C -3.183229 -0.953225 3.491117

H -2.294622 -1.844976 1.740701

C -4.759477 0.187023 -3.102384

H -3.376449 -0.944258 -4.312096

H -5.917080 1.300634 -1.661076

C 0.496073 4.550552 -0.465428

H 1.489212 4.142571 1.408600

H -0.634111 4.658122 -2.299066

C -3.493023 0.232719 4.159745

H -3.386730 2.387134 4.100630

H -3.453153 -1.912004 3.925641

H -5.552960 0.166698 -3.844694

H 0.942913 5.533907 -0.585645

H -4.002930 0.202360 5.119099

C 2.986961 -2.767545 -1.219428

H 3.636357 -3.258254 -1.948814

H 3.444898 -2.619356 -0.244445

**10a׳***-endo-re*

Atom X Y Z

C 2.38364500 -0.71288800 0.20632700

C 2.46254500 0.44232200 0.88925400

Rh 0.26409200 -0.01409600 0.65583800

C 0.00629200 1.06255400 2.44183300

C 0.79980100 2.29301200 2.55508600

C 0.93914200 3.24284900 1.58816300

Cl 0.12354500 -2.25224100 1.75437300

H -1.06601400 1.26939100 2.38613600

H 0.20462100 0.34685000 3.24187500

H 1.31314000 2.45713100 3.50341200

C 1.61149200 4.56388300 1.87683100

H 0.92761600 5.40226200 1.69793200

H 2.48386000 4.72613700 1.23027900

H 1.94276900 4.61155600 2.91826800

C 0.21661400 1.74391200 -0.29113900

H -0.13567200 1.74041200 -1.32439300

C 0.51308200 2.96021000 0.22663800

O 0.43153000 4.12258400 -0.59076600

C 1.21508800 4.21464600 -1.70188300

O 2.00748500 3.37542700 -2.06357100

C 0.94539700 5.52116900 -2.41324000

H 1.12450300 6.36463400 -1.73854500

H -0.10488500 5.56649000 -2.72027200

H 1.59158600 5.59991100 -3.28817100

H 2.75447700 1.47130900 0.76051700

P -2.10960500 -0.29563700 0.21255600

C -2.47878100 -1.88877400 -0.63701100

C -2.80569800 0.97093900 -0.94066100

C -3.26631400 -0.25241300 1.65400800

C -1.49621100 -2.48477800 -1.44045600

C -3.74049500 -2.49370700 -0.53781000

C -2.92486200 2.30450400 -0.50833000

C -3.14220300 0.66111200 -2.26659400

C -4.53333200 0.35049800 1.57497800

C -2.88326600 -0.87912400 2.85278600

C -1.77438700 -3.65807700 -2.14236400

H -0.50503300 -2.04742900 -1.50541500

C -4.01264100 -3.67239100 -1.23374300

H -4.50895700 -2.05169700 0.08844200

C -3.38462400 3.29504300 -1.37500500

H -2.66244100 2.57004800 0.51082700

C -3.59428900 1.65749100 -3.13605300

H -3.05742900 -0.35945900 -2.62437300

C -5.39496500 0.33293400 2.67345600

H -4.85269200 0.83528900 0.65890700

C -3.75060800 -0.89319700 3.94605900

H -1.91572700 -1.36822700 2.91617600

C -3.03200600 -4.25507500 -2.03890700

H -0.99928300 -4.10983500 -2.75477000

H -4.99111100 -4.13628300 -1.14217300

C -3.71929300 2.97427200 -2.69319700

H -3.47570900 4.31795500 -1.02013600

H -3.85431200 1.39793700 -4.15879000

C -5.00492400 -0.28602600 3.86203400

H -6.37148000 0.80372900 2.59626900

H -3.44052900 -1.38235700 4.86546800

H -3.24525200 -5.17468200 -2.57736300

H -4.07574900 3.74675500 -3.36941700

H -5.67604100 -0.29664900 4.71686400

C 3.03213600 -0.79405000 1.56988400

H 2.50911600 -1.26769600 2.39466200

C 2.36623400 -1.36271400 -1.09114100

C 2.45412300 -2.76443100 -1.17947100

C 2.26978200 -0.59245500 -2.26895300

C 2.45421900 -3.38437800 -2.42668300

H 2.50198900 -3.34633700 -0.26468600

C 2.26686100 -1.22267400 -3.51130300

H 2.21270100 0.49023500 -2.19575700

C 2.35872000 -2.61631800 -3.59244200

H 2.52692700 -4.46651600 -2.49162700

H 2.20148700 -0.62678900 -4.41746200

H 2.35965400 -3.10350500 -4.56405800

C 4.51893500 -0.96971500 1.64900400

O 5.08425900 -1.55617100 2.54825400

O 5.15889200 -0.38299700 0.60567900

C 6.59906000 -0.50147600 0.58147200

H 6.97536000 -0.41896700 1.60428200

H 6.93232700 0.36154300 -0.00052700

C 7.03709800 -1.80979100 -0.06104900

H 8.13054400 -1.84145900 -0.13487400

H 6.70876100 -2.66117700 0.54181400

H 6.62022600 -1.90677300 -1.06902300

**TS2a׳***-endo-re*

Atom X Y Z

C 2.30569700 -0.62916100 0.37045100

C 1.95146800 0.47681200 -0.43001500

Rh 0.16682500 -0.31828700 0.50578700

C -0.15694300 0.52463700 2.42986900

C 0.70001300 1.64879100 2.74985000

C 0.83159500 2.76942200 1.96991000

Cl -0.09971500 -2.55852800 1.41568700

H -1.20119400 0.81066400 2.29349000

H -0.06671600 -0.31664000 3.11561400

H 1.27858100 1.60086700 3.67262200

C 1.52237100 4.01147200 2.47797500

H 0.82870500 4.86045300 2.49965600

H 2.36213400 4.29950500 1.83355700

H 1.90276300 3.85718400 3.49160600

C 0.18656600 1.61719600 -0.15832100

H -0.13662600 1.79359500 -1.18178100

C 0.37514100 2.73725700 0.60229200

O 0.16516900 4.01614800 0.03359400

C 0.88892800 4.39897100 -1.06310400

O 1.74862200 3.73010900 -1.58417200

C 0.44415600 5.76923800 -1.51669800

H 1.07670100 6.10234000 -2.34017000

H 0.49688900 6.48244900 -0.68819100

H -0.59973300 5.72497600 -1.84578400

H 2.01836800 0.71369200 -1.48411600

P -2.24383000 -0.45186800 -0.11428000

C -2.63617500 -1.91801100 -1.16490700

C -2.78222600 0.96947600 -1.16806400

C -3.51907600 -0.51023600 1.22634100

C -1.59498100 -2.63752700 -1.76722000

C -3.96328100 -2.30665400 -1.40854800

C -2.96422300 2.23772500 -0.58647200

C -2.88764700 0.85181200 -2.56281600

C -4.76150200 0.13885200 1.12418100

C -3.24304000 -1.27136900 2.37566600

C -1.87449500 -3.71577200 -2.60948600

H -0.56438000 -2.37597900 -1.55138900

C -4.24025000 -3.38684600 -2.24603600

H -4.78237600 -1.77064800 -0.93854800

C -3.26768700 3.34778500 -1.37497500

H -2.87762900 2.35998800 0.48885000

C -3.18169200 1.96670400 -3.35186900

H -2.74748900 -0.11399300 -3.03721700

C -5.70204200 0.03511700 2.15080700

H -4.99982400 0.72923200 0.24580800

C -4.18881800 -1.37265000 3.39716600

H -2.29560000 -1.79607700 2.45615700

C -3.19620000 -4.09164200 -2.85055600

H -1.05679800 -4.27016100 -3.06166300

H -5.27160600 -3.68060800 -2.42235300

C -3.37689700 3.21600500 -2.76147600

H -3.41620900 4.31526600 -0.90287900

H -3.26459200 1.85319500 -4.42949200

C -5.41740600 -0.71849500 3.29069400

H -6.65783000 0.54391400 2.05591400

H -3.96046500 -1.96667000 4.27801000

H -3.41345800 -4.93591800 -3.49945700

H -3.61374800 4.08019600 -3.37640500

H -6.15008700 -0.79681500 4.08962300

C 2.99020800 0.69750200 0.63543700

H 2.73108900 1.27869700 1.51263000

C 2.89375500 -1.95808200 0.16988100

C 3.70681300 -2.51462000 1.16909800

C 2.68436900 -2.68239400 -1.01491700

C 4.29905000 -3.76337300 0.98671600

H 3.86548000 -1.96175300 2.09069000

C 3.27637200 -3.92981400 -1.19661600

H 2.06774700 -2.25254900 -1.80044800

C 4.08589300 -4.47517600 -0.19590800

H 4.92435800 -4.18291300 1.77027200

H 3.11088900 -4.47678600 -2.12128200

H 4.54694700 -5.44897200 -0.33798700

C 4.41959600 0.90472700 0.23318000

O 5.23702600 1.46385500 0.93570700

O 4.68545000 0.39846300 -0.99241900

C 6.05135700 0.49961400 -1.45804900

H 6.46511100 1.45465500 -1.12543900

H 5.96585600 0.49970400 -2.54752600

C 6.88812100 -0.67098500 -0.96276100

H 7.89383800 -0.61873300 -1.39636000

H 6.98156600 -0.64197500 0.12653400

H 6.43334500 -1.62395800 -1.25130300

**12a׳***-endo-re*

Atom X Y Z

C -1.97102400 -0.83945600 -0.20868200

C -1.79917100 0.36212700 0.83459200

Rh -0.03355300 -0.32435200 -0.65094500

C 0.59024300 0.55952200 -2.51082000

C -0.78423900 0.84795600 -2.34951500

C -1.22979100 2.18546100 -1.87806500

Cl 0.50478800 -2.55682700 -1.65131500

H 1.31941500 1.35071500 -2.35694400

H 0.89796400 -0.24202300 -3.17530700

H -1.49521800 0.26657800 -2.93772800

C -1.78961200 3.15504400 -2.88405600

H -1.04377100 3.41494400 -3.64627400

H -2.12859900 4.07742000 -2.40583600

H -2.64108800 2.70752500 -3.41475600

C -0.74444400 1.34029200 0.39200200

H -0.12211100 1.68657700 1.21748600

C -1.08525200 2.42267700 -0.56032000

O -1.19574300 3.73656600 -0.06978200

C -2.14908900 4.01121400 0.87269900

O -3.01205100 3.23473600 1.20472500

C -1.95806400 5.40710600 1.41483600

H -2.77426800 5.64711900 2.09696600

H -1.92381500 6.13383100 0.59700200

H -1.00130900 5.46665400 1.94520200

H -1.92134900 0.13950300 1.89084800

P 2.31875500 -0.08268500 0.08749400

C 2.91600000 -1.41231300 1.22461500

C 2.52380000 1.46352000 1.08214000

C 3.68095900 -0.00495500 -1.16373800

C 2.09112000 -2.50357500 1.52733300

C 4.20806500 -1.35781900 1.77666900

C 2.49572900 2.71205700 0.43448900

C 2.55532200 1.44206300 2.48541900

C 4.75152200 0.89987200 -1.08485800

C 3.65774600 -0.94606400 -2.20839500

C 2.54304800 -3.51262400 2.38305400

H 1.11624100 -2.58821200 1.06057000

C 4.65412300 -2.36422000 2.63100800

H 4.86908700 -0.53018500 1.53563700

C 2.52361600 3.89921300 1.16576400

H 2.45487700 2.76152600 -0.64967500

C 2.57482700 2.63237800 3.21774100

H 2.57099300 0.49414200 3.01314000

C 5.77293200 0.87167100 -2.03739600

H 4.79544200 1.63076900 -0.28415100

C 4.68438400 -0.97112600 -3.15299300

H 2.83786300 -1.65740700 -2.27337300

C 3.81967400 -3.44343300 2.93858300

H 1.89614500 -4.35818100 2.59996600

H 5.65504000 -2.31044600 3.05110100

C 2.56401300 3.86364400 2.56211800

H 2.51095700 4.85155700 0.64261600

H 2.60299900 2.59179600 4.30339300

C 5.74173800 -0.06213200 -3.07366500

H 6.59384700 1.58041700 -1.96416800

H 4.65384100 -1.70508300 -3.95386600

H 4.17087100 -4.23060900 3.60059300

H 2.58662500 4.78814800 3.13284300

H 6.53750900 -0.08259100 -3.81367800

C -3.01711100 0.21435200 -0.05553100

H -3.12052600 0.97670000 -0.82176000

C -2.28371100 -2.25570900 0.05762500

C -3.13489200 -2.94449400 -0.82461600

C -1.77362600 -2.93905900 1.17311700

C -3.44359100 -4.28475500 -0.61073500

H -3.53633800 -2.42267600 -1.68921700

C -2.09782900 -4.27563900 1.39606100

H -1.14484500 -2.40700100 1.88125300

C -2.92632800 -4.95542200 0.50110100

H -4.09003800 -4.80797900 -1.31035200

H -1.70651200 -4.78674900 2.27186000

H -3.17382600 -5.99969700 0.67193000

C -4.30622700 -0.14342900 0.62247300

O -4.41103500 -0.61676700 1.73457700

O -5.35394300 0.13209300 -0.18261700

C -6.65904200 -0.16808900 0.36243000

H -6.78319900 0.38357900 1.29969600

H -6.70364100 -1.23634600 0.59779800

C -7.68990700 0.23218800 -0.67664900

H -8.69729900 0.01929700 -0.30220600

H -7.62416900 1.30192700 -0.90037500

H -7.54391800 -0.32469500 -1.60800900

**TS4a׳***-endo-re*

Atom X Y Z

C 2.41960200 0.29214400 -0.05209600

C 2.10231700 -0.65690700 1.11971800

Rh 0.18437200 0.19974700 -0.45909900

C 1.72055200 -0.23999700 -2.07958500

C 1.79054000 -1.70950800 -2.20891100

C 1.10985200 -2.53809900 -1.40028900

Cl 0.06716000 2.40033200 -1.42541300

H 0.90194100 0.21424200 -2.64667400

H 2.63324500 0.28466000 -2.35713400

H 2.47380600 -2.12629900 -2.95039400

C 1.15830900 -4.03952600 -1.56042600

H 0.16366300 -4.45138100 -1.77070800

H 1.52201700 -4.52840500 -0.64990200

H 1.81322200 -4.31249400 -2.39330100

C 0.78560800 -1.31282100 0.91248500

H 0.17610100 -1.43566500 1.80136300

C 0.31878100 -1.96584200 -0.25705700

O -0.91382000 -2.67784700 -0.09552900

C -0.95274400 -3.74308100 0.76612400

O -0.02593200 -4.09042200 1.45946200

C -2.30995100 -4.40219700 0.72888500

H -2.70917600 -4.42671900 -0.28834500

H -3.00374300 -3.82234200 1.34668900

H -2.22910900 -5.41103700 1.13704400

H 2.32617000 -0.32091700 2.12927800

P -2.13962500 0.42106000 0.01887900

C -2.68226000 -0.40611300 1.58891200

C -3.23374000 -0.26720800 -1.30311300

C -2.83988200 2.12640800 0.23494200

C -3.84473300 -1.18253500 1.69778800

C -1.90356700 -0.20131400 2.74178100

C -2.67739600 -1.06914600 -2.30943200

C -4.61125800 0.01241000 -1.34292000

C -3.12348100 2.66789800 1.49652900

C -3.07633400 2.91400800 -0.90520600

C -4.21970800 -1.73470900 2.92597700

H -4.46233800 -1.36053000 0.82414900

C -2.27934200 -0.75097600 3.96888300

H -1.00166800 0.40275000 2.68043600

C -3.48097600 -1.59514100 -3.32348200

H -1.61150600 -1.26921100 -2.29943300

C -5.41320400 -0.51790600 -2.35377100

H -5.05601200 0.65940900 -0.59285800

C -3.63202700 3.96382700 1.61725800

H -2.96283100 2.08337600 2.39529600

C -3.59277200 4.20220500 -0.78167700

H -2.85081300 2.52374100 -1.89046500

C -3.43953800 -1.52194700 4.06367700

H -5.12680400 -2.33024600 2.99116300

H -1.66395800 -0.57825200 4.84759700

C -4.84954800 -1.32478400 -3.34544700

H -3.03284200 -2.20874000 -4.10048800

H -6.47607100 -0.29197400 -2.37187800

C -3.86988100 4.73370400 0.47976200

H -3.84784100 4.36338000 2.60471200

H -3.76735700 4.79532600 -1.67519800

H -3.73240100 -1.95359500 5.01672700

H -5.47383800 -1.73085900 -4.13698100

H -4.26809600 5.74058500 0.57359200

C 3.28952600 -0.92548900 0.24083600

H 3.28037900 -1.77136800 -0.43263000

C 2.98971000 1.65571200 0.05177700

C 4.08952300 2.02077700 -0.74278200

C 2.47083400 2.60045600 0.95083300

C 4.64863300 3.29446000 -0.64753500

H 4.51860400 1.29441400 -1.42857800

C 3.03728400 3.86764900 1.05712100

H 1.61364700 2.33588600 1.56159600

C 4.12435400 4.22213300 0.25428200

H 5.49725600 3.55960500 -1.27280900

H 2.62371900 4.58401400 1.76180800

H 4.56150600 5.21395100 0.33362900

C 4.62873200 -0.67609200 0.86734200

O 4.82468500 -0.16807700 1.95118900

O 5.61557100 -1.10698600 0.04989600

C 6.96317000 -0.90929100 0.53711600

H 7.11634900 0.15974700 0.71580100

H 7.06941700 -1.42442900 1.49721600

C 7.91244200 -1.45676500 -0.51247600

H 7.73738800 -2.52445200 -0.68030600

H 7.78694500 -0.93284900 -1.46568500

H 8.94836400 -1.32524700 -0.18112900

**14a׳**

Atom X Y Z

C -2.85349500 -0.04408300 -1.00767500

C -2.24298300 -0.04239800 -2.41050600

C -0.97816900 0.72234800 -2.74588200

C -0.34070200 1.86704300 -2.22553900

C -2.07813900 0.47758400 0.17000300

Rh 0.64187900 0.14318300 -1.29961800

Cl 1.51366400 -1.39186200 -2.90872000

H -3.01911400 0.28276700 -3.12350200

H -2.03373200 -1.08615700 -2.67580500

H -2.29012100 -0.03319600 1.10436700

H -0.71429300 0.54005800 -3.78753400

C -0.18898200 2.00429800 -0.79659500

C -0.69808300 1.04938800 0.12523000

H -0.29138500 1.19743800 1.12309500

C 0.40285600 2.81315200 -3.14149100

H -0.21545700 3.70309600 -3.32332500

H 1.35073500 3.14521100 -2.71229100

H 0.59621800 2.33627800 -4.10595500

O 0.62315400 3.05061000 -0.32682700

C -0.02454600 4.09171000 0.29520300

O -1.22518500 4.17405800 0.37478500

C 0.98002600 5.07494000 0.84027800

H 0.45454500 5.92534700 1.27596100

H 1.59701800 4.58425000 1.60068500

H 1.64973200 5.41452100 0.04362900

P 2.22225500 -0.56344100 0.31618800

C 2.31734400 0.49767000 1.83247000

C 3.29218100 1.49975300 1.95090500

C 1.34956700 0.38128600 2.84638400

C 3.30312600 2.35677900 3.05440000

H 4.05180600 1.60818000 1.18353100

C 1.36219500 1.23685700 3.94898500

H 0.59108100 -0.39440600 2.78612500

C 2.33922100 2.22928100 4.05632200

H 4.07505600 3.11804100 3.13332100

H 0.60945000 1.12489000 4.72473200

H 2.35188200 2.89299300 4.91658500

C 1.96236600 -2.26477000 0.99675000

C 1.34607900 -3.22690800 0.17922700

C 2.38351700 -2.63911800 2.28473600

C 1.15485300 -4.52978800 0.64378800

H 1.03824000 -2.95939400 -0.82674400

C 2.18669500 -3.94208600 2.74534800

H 2.85890600 -1.91381900 2.93753600

C 1.56995000 -4.89008100 1.92672100

H 0.67997300 -5.26252300 -0.00308600

H 2.51601500 -4.21380500 3.74487100

H 1.41561900 -5.90369400 2.28722700

C 3.96502600 -0.56183400 -0.29458200

C 4.97415300 -1.31078500 0.32970500

C 4.29546100 0.24481400 -1.39338500

C 6.28894300 -1.24634600 -0.13202500

H 4.73558500 -1.95545900 1.16977200

C 5.61357900 0.31352000 -1.84865500

H 3.51282800 0.79734300 -1.90359400

C 6.61188100 -0.43146900 -1.21958700

H 7.05992400 -1.83692600 0.35577900

H 5.85431200 0.93694700 -2.70545400

H 7.63610000 -0.38542400 -1.58014900

C -3.25064700 1.29173600 -0.37423600

H -2.99679000 2.18184800 -0.94073700

C -3.75693100 -1.23832200 -0.80553600

C -5.07680900 -1.21799600 -1.27821100

C -3.27010000 -2.41321600 -0.21798500

C -5.89538000 -2.34188700 -1.15397800

H -5.46949300 -0.31077100 -1.73091200

C -4.08701300 -3.53848800 -0.09366400

H -2.24550100 -2.44484000 0.14450600

C -5.40274000 -3.50610600 -0.56055300

H -6.91821200 -2.30657300 -1.52008200

H -3.69440100 -4.44088700 0.36799900

H -6.03927900 -4.38158100 -0.46304100

C -4.53263900 1.45954500 0.37020900

O -5.41943900 2.21065100 0.01500300

O -4.61209700 0.67925700 1.47014800

C -5.86986600 0.71267000 2.17838900

H -6.66939800 0.42995100 1.48611200

H -6.06258200 1.73875800 2.50750800

C -5.75981800 -0.25303600 3.34376900

H -6.69784200 -0.26259200 3.90991800

H -5.55936400 -1.26881400 2.98823800

H -4.95205100 0.04243700 4.02164800

**10b***-endo-re*

Atom X Y Z

C -0.507369 1.239095 -2.029418

C -1.659705 0.435374 -2.237635

Rh -0.732067 -0.136720 -0.338754

C -1.126553 1.355520 1.090189

C -2.445511 1.937391 1.215372

C -3.624196 1.242368 1.103892

Cl -0.320193 -2.443842 -1.196470

H -0.969513 0.537727 1.827586

H -0.335195 2.098630 1.200479

H -2.516698 3.001151 1.445867

C -4.943121 1.878287 1.471843

H -5.407227 1.355806 2.317428

H -5.654271 1.840732 0.638627

H -4.801503 2.924323 1.759081

C -2.617527 -0.715333 -0.099386

H -2.831896 -1.698114 -0.516834

C -3.613834 -0.104550 0.597409

O -4.797893 -0.851915 0.837770

C -5.741328 -0.889383 -0.148696

O -5.676848 -0.248991 -1.172160

C -6.858178 -1.831389 0.233184

H -7.247485 -1.580202 1.224722

H -6.474049 -2.855972 0.283487

H -7.653527 -1.772873 -0.510633

H 0.492359 1.129163 -2.436701

H -1.778065 -0.503808 -2.769019

P 1.736116 -0.024717 0.060368

C 1.893131 -0.637050 1.795210

C 2.676925 1.574779 0.042689

C 2.857490 -1.122767 -0.910657

C 1.410944 -1.930003 2.078365

C 2.377350 0.155889 2.845934

C 4.067949 1.606883 0.247782

C 2.008529 2.782666 -0.203267

C 3.888898 -1.859170 -0.310468

C 2.701955 -1.182170 -2.303492

C 1.433193 -2.416512 3.385335

H 1.015797 -2.547081 1.274995

C 2.389190 -0.336053 4.153985

H 2.750193 1.155734 2.648837

C 4.762992 2.815158 0.223912

H 4.612690 0.683087 0.415881

C 2.705926 3.993535 -0.230442

H 0.940457 2.777494 -0.384200

C 4.754681 -2.629227 -1.090469

H 4.011805 -1.844416 0.768011

C 3.571791 -1.944744 -3.080957

H 1.882666 -0.655444 -2.781404

C 1.919937 -1.621412 4.426460

H 1.062868 -3.417588 3.588997

H 2.768885 0.288768 4.958162

C 4.083154 4.012878 -0.014041

H 5.837787 2.820604 0.384610

H 2.170095 4.918669 -0.425562

C 4.600873 -2.670078 -2.476440

H 5.546032 -3.199444 -0.611054

H 3.433266 -1.986993 -4.157777

H 1.930799 -2.001646 5.444450

H 4.627130 4.953332 -0.036899

H 5.272452 -3.272296 -3.082455

C -1.328580 2.534791 -1.979831

C -2.591373 1.646780 -2.222933

H -3.382230 1.671777 -1.473637

H -3.037189 1.808486 -3.211472

H -1.310898 3.113113 -1.052658

H -1.098824 3.203675 -2.817920

**10b***-exo-re*

Atom X Y Z

C -0.534287 -0.220883 -2.229162

C -1.869191 -0.617851 -1.963994

Rh -0.806487 -0.294631 -0.072042

C -1.137371 1.783439 -0.100375

C -2.434154 2.298249 -0.509973

C -3.643173 1.768371 -0.145689

Cl -0.526968 -2.504794 1.079807

H -1.025153 1.788417 1.002379

H -0.316659 2.339837 -0.553953

H -2.448982 3.195464 -1.130102

C -4.936436 2.497072 -0.422019

H -5.451274 2.750587 0.512864

H -5.624637 1.883323 -1.014666

H -4.747022 3.427490 -0.965432

C -2.723671 -0.492975 0.430561

H -2.977972 -1.481451 0.809710

C -3.694224 0.461232 0.458284

O -4.915751 0.136474 1.103135

C -5.836609 -0.589656 0.401059

O -5.715450 -0.891342 -0.762918

C -7.006016 -0.944537 1.288103

H -7.786388 -1.415961 0.689682

H -7.395872 -0.050314 1.784014

H -6.675459 -1.634773 2.071933

H -0.150900 0.748465 -2.535470

H -2.789193 -0.050228 -2.016988

P 1.682263 0.051138 0.108285

C 2.192264 0.214454 1.880244

C 2.234165 1.649900 -0.643852

C 2.901365 -1.178897 -0.554007

C 1.241874 0.080010 2.902081

C 3.531031 0.465779 2.226402

C 2.389170 1.747909 -2.039205

C 2.380436 2.814665 0.125694

C 4.142018 -0.797729 -1.093327

C 2.597890 -2.547232 -0.450814

C 1.619062 0.211618 4.241469

H 0.213183 -0.166327 2.660174

C 3.904962 0.596727 3.562555

H 4.286124 0.554861 1.450988

C 2.696043 2.968252 -2.641365

H 2.292412 0.861186 -2.658969

C 2.682488 4.037403 -0.479511

H 2.264195 2.770433 1.203564

C 5.052884 -1.762369 -1.527888

H 4.403484 0.251344 -1.182326

C 3.515186 -3.506303 -0.884839

H 1.650764 -2.856798 -0.018378

C 2.947576 0.472944 4.573748

H 0.871429 0.096610 5.021321

H 4.944313 0.789667 3.814766

C 2.842548 4.118933 -1.862778

H 2.822916 3.018220 -3.719565

H 2.794766 4.925701 0.136386

C 4.741755 -3.119200 -1.426493

H 6.007102 -1.449339 -1.943808

H 3.264462 -4.559997 -0.796537

H 3.240597 0.571130 5.615654

H 3.080740 5.069689 -2.331683

H 5.452032 -3.868787 -1.765451

C -1.620797 -2.054155 -2.427993

H -1.725201 -2.831306 -1.668911

H -2.218007 -2.318785 -3.308549

C -0.157131 -1.607960 -2.769659

H 0.655999 -2.144651 -2.277215

H 0.036330 -1.585209 -3.849790

**11b***-endo-re*

Atom X Y Z

C 0.451715 -3.519119 -0.400723

C 2.053382 -2.279585 1.499030

C 2.007551 -0.936188 2.037040

C 2.420803 0.194562 1.330089

C -0.551493 -2.777483 -1.057491

Rh 0.442387 -1.467545 0.415264

Cl -1.125740 -2.328368 2.161064

H 2.880331 -2.527757 0.835638

H 1.758799 -3.070201 2.184332

H -1.616314 -2.716477 -0.863596

H 1.634351 -0.794399 3.048359

C 2.702720 0.069111 -0.101628

C 1.858873 -0.707352 -0.802593

H 1.946464 -0.804257 -1.883778

C 2.568873 1.526395 2.010402

H 3.627932 1.682482 2.253920

H 2.250871 2.348803 1.361370

H 1.999383 1.562229 2.942967

O 3.679714 0.877042 -0.715189

C 4.950974 0.850513 -0.221190

O 5.280371 0.228536 0.762790

C 5.861974 1.702572 -1.071855

H 6.856221 1.730105 -0.624771

H 5.922155 1.286245 -2.083193

H 5.459611 2.716637 -1.161208

H 0.380773 -4.183135 0.453908

P -1.147448 0.421447 -0.102093

C -0.431961 1.650669 -1.283164

C -0.046356 2.941435 -0.891693

C -0.158640 1.244495 -2.602068

C 0.577509 3.805439 -1.796154

H -0.240914 3.282739 0.119383

C 0.452129 2.111422 -3.507282

H -0.436457 0.245950 -2.928350

C 0.824289 3.396946 -3.106341

H 0.863668 4.802827 -1.472424

H 0.641501 1.780129 -4.524971

H 1.303911 4.071917 -3.809989

C -2.783491 -0.028800 -0.848825

C -3.535184 -1.022686 -0.195469

C -3.320577 0.591196 -1.986902

C -4.793538 -1.383935 -0.677496

H -3.131537 -1.510283 0.688830

C -4.579785 0.220658 -2.466858

H -2.765197 1.366863 -2.503450

C -5.318877 -0.766800 -1.815281

H -5.362022 -2.152619 -0.160769

H -4.981613 0.711283 -3.349756

H -6.298261 -1.052483 -2.190001

C -1.695277 1.458358 1.330626

C -2.615630 2.505972 1.150527

C -1.214127 1.191843 2.618893

C -3.020453 3.286350 2.232090

H -3.019759 2.709564 0.162901

C -1.625711 1.972360 3.703220

H -0.555412 0.346258 2.777850

C -2.522543 3.022628 3.511717

H -3.730431 4.094733 2.078157

H -1.253922 1.745865 4.698917

H -2.843967 3.627298 4.355719

C 1.287152 -3.677089 -1.671808

H 2.287695 -3.232794 -1.669733

H 1.366823 -4.719065 -2.003364

C 0.157652 -2.881947 -2.412483

H 0.444250 -1.957875 -2.924467

H -0.399446 -3.506771 -3.120802

**11b***-exo-re*

Atom X Y Z

C 0.417939 -3.226512 -1.295146

C 1.906171 -2.635657 0.998751

C 2.008117 -1.390619 1.734653

C 2.526610 -0.213778 1.192326

C -0.626054 -2.358357 -1.681830

Rh 0.379324 -1.512311 0.093078

Cl -1.206703 -2.388337 1.831483

H 2.710294 -2.860056 0.300103

H 1.532290 -3.488745 1.559093

H -0.658084 -1.591900 -2.451931

H 1.665036 -1.366933 2.766014

C 2.766911 -0.141520 -0.252983

C 1.839106 -0.713661 -1.038647

H 1.898146 -0.668412 -2.126745

C 2.827654 0.977758 2.057531

H 3.904850 0.997377 2.268614

H 2.568773 1.916577 1.556772

H 2.298940 0.920645 3.012608

O 3.810484 0.653443 -0.764905

C 5.082124 0.419005 -0.328671

O 5.364681 -0.392728 0.522363

C 6.057862 1.315680 -1.052077

H 6.040101 1.097064 -2.125071

H 5.770221 2.364851 -0.929347

H 7.061565 1.153260 -0.657950

H 1.432176 -3.328808 -1.669285

P -1.033947 0.579078 -0.066527

C -0.225153 1.909446 -1.066190

C 0.249491 3.099788 -0.494539

C 0.025964 1.680384 -2.431526

C 0.938400 4.038337 -1.267864

H 0.074887 3.304361 0.556310

C 0.701689 2.623015 -3.205493

H -0.319060 0.761879 -2.898460

C 1.162318 3.806985 -2.624629

H 1.292957 4.955694 -0.805036

H 0.872376 2.429612 -4.261278

H 1.692497 4.540697 -3.225717

C -2.714606 0.391693 -0.825139

C -3.573631 -0.559205 -0.244429

C -3.179974 1.160114 -1.902047

C -4.865481 -0.734715 -0.739369

H -3.225470 -1.157943 0.593354

C -4.473585 0.973447 -2.398059

H -2.543356 1.912174 -2.355544

C -5.318547 0.026502 -1.820002

H -5.517937 -1.471984 -0.279071

H -4.818598 1.576585 -3.234042

H -6.324561 -0.116003 -2.205869

C -1.461417 1.432637 1.518411

C -2.352890 2.519854 1.531090

C -0.909038 0.994640 2.728602

C -2.663842 3.166693 2.726040

H -2.809677 2.858140 0.605419

C -1.224763 1.642292 3.926325

H -0.270045 0.119889 2.736889

C -2.097337 2.729858 3.927209

H -3.354439 4.005896 2.720942

H -0.799385 1.282443 4.859358

H -2.346249 3.229618 4.859702

C -1.708083 -3.387318 -1.340930

H -2.433775 -3.113668 -0.576032

H -2.231096 -3.743658 -2.236997

C -0.550504 -4.341329 -0.893010

H -0.553488 -4.638637 0.157318

H -0.450354 -5.228911 -1.529746

**12b***-endo-re*

Atom X Y Z

C 0.861837 1.888575 1.299670

C 2.315786 1.710544 1.793430

Rh 0.725250 0.119617 0.245341

C 0.895643 1.061632 -1.609896

C 2.266953 1.585198 -1.896935

C 3.369728 1.158043 -1.264839

Cl 0.642819 -1.656738 1.990909

H 0.683006 0.233279 -2.308079

H 0.146572 1.834445 -1.811511

H 2.380882 2.320357 -2.699042

C 4.779185 1.533807 -1.663869

H 5.341007 0.655093 -2.005241

H 5.340261 1.959780 -0.823112

H 4.767252 2.261230 -2.481597

C 2.864831 0.439243 1.162064

H 2.997372 -0.390566 1.847558

C 3.236898 0.209332 -0.130862

O 3.747823 -1.067957 -0.445720

C 4.878958 -1.531325 0.186777

O 5.559317 -0.861215 0.922699

C 5.119749 -2.971143 -0.190100

H 5.067624 -3.099376 -1.275323

H 4.334170 -3.593747 0.252541

H 6.094113 -3.286822 0.184661

H 0.068349 1.878873 2.051673

H 2.428176 1.610980 2.877756

P -1.551086 -0.101342 -0.080756

C -1.675358 -1.439184 -1.342005

C -2.572008 1.300140 -0.727563

C -2.598406 -0.672162 1.327195

C -1.332519 -2.748061 -0.954231

C -1.962324 -1.179767 -2.691394

C -3.853770 1.081941 -1.262096

C -2.113674 2.617446 -0.594490

C -3.695459 -1.522609 1.125846

C -2.347330 -0.175595 2.613798

C -1.309787 -3.775888 -1.896956

H -1.067775 -2.953230 0.079235

C -1.934033 -2.213598 -3.630171

H -2.213577 -0.174575 -3.013614

C -4.642791 2.154881 -1.673885

H -4.241012 0.072359 -1.357078

C -2.908504 3.692758 -1.000006

H -1.134433 2.801975 -0.170848

C -4.528827 -1.863843 2.192345

H -3.895098 -1.934408 0.141374

C -3.186766 -0.511045 3.675607

H -1.478430 0.447087 2.793291

C -1.612187 -3.512869 -3.235373

H -1.047905 -4.782696 -1.583315

H -2.164407 -1.999431 -4.670402

C -4.171262 3.464090 -1.545237

H -5.629072 1.968264 -2.090188

H -2.536027 4.707576 -0.889308

C -4.279066 -1.355219 3.467709

H -5.371008 -2.529927 2.024455

H -2.974989 -0.125929 4.669196

H -1.591527 -4.315664 -3.967511

H -4.788663 4.299508 -1.864092

H -4.927352 -1.622950 4.297774

C 1.212471 3.287736 0.719273

C 2.670005 3.121760 1.239617

H 3.445246 3.149190 0.471866

H 2.940697 3.823982 2.033826

H 1.130880 3.409855 -0.362606

H 0.652837 4.101874 1.196828

**12b***-exo-re*

Atom X Y Z

C -0.820832 -2.034800 0.862509

C -2.331348 -2.172759 1.158734

Rh -0.823036 -0.141806 0.054119

C -0.819034 -1.178452 -1.747638

C -2.176494 -1.667687 -2.150937

C -3.324094 -1.273919 -1.582950

Cl -1.196643 2.096532 1.057974

H -0.477028 -0.424578 -2.476485

H -0.094693 -1.998891 -1.788949

H -2.226793 -2.363328 -2.993714

C -4.696978 -1.667710 -2.076894

H -5.278655 -0.782676 -2.362421

H -5.271218 -2.189632 -1.300087

H -4.623347 -2.322923 -2.950230

C -2.900858 -0.789805 0.869187

H -3.087939 -0.126173 1.705300

C -3.289507 -0.395582 -0.383627

O -3.881826 0.837735 -0.630546

C -4.714210 1.465123 0.280036

O -5.223434 0.909046 1.218800

C -4.876869 2.910096 -0.104614

H -5.693535 3.351268 0.468342

H -5.059707 3.011208 -1.178223

H -3.937862 3.426578 0.125924

H -0.348070 -2.818983 0.266954

H -2.888281 -2.960115 0.640501

P 1.479385 0.132775 -0.074448

C 1.990340 1.641763 -1.006407

C 2.360841 -1.239614 -0.943524

C 2.378622 0.282726 1.536194

C 1.045532 2.341073 -1.770500

C 3.318744 2.096814 -0.977851

C 2.450017 -2.502546 -0.330288

C 2.864823 -1.087249 -2.243244

C 3.652844 -0.274974 1.734944

C 1.791737 1.027471 2.573286

C 1.425865 3.466493 -2.505069

H 0.007532 2.024846 -1.767027

C 3.694766 3.222547 -1.709509

H 4.059402 1.577553 -0.376712

C 3.037344 -3.577498 -0.996527

H 2.070161 -2.644669 0.677094

C 3.446930 -2.167795 -2.911516

H 2.809603 -0.123242 -2.737770

C 4.320567 -0.099819 2.948708

H 4.128252 -0.850255 0.947876

C 2.466489 1.200772 3.782679

H 0.815888 1.480706 2.425787

C 2.748876 3.907863 -2.476800

H 0.682083 4.003699 -3.086960

H 4.724665 3.567722 -1.675822

C 3.536288 -3.413736 -2.291598

H 3.104631 -4.543565 -0.503337

H 3.832863 -2.029538 -3.917940

C 3.728325 0.635682 3.976280

H 5.304753 -0.539675 3.087456

H 1.999420 1.780000 4.574609

H 3.042345 4.787735 -3.043181

H 3.991759 -4.252282 -2.811267

H 4.248562 0.769188 4.921140

C -1.994116 -2.426483 2.654370

H -2.536470 -1.818447 3.386481

H -2.113002 -3.480905 2.920629

C -0.509356 -2.043923 2.384201

H -0.228883 -1.067703 2.792378

H 0.242878 -2.772980 2.709616

**13b***-endo-re*

Atom X Y Z

C -1.610085 -3.263018 0.183656

C -2.918563 -2.475545 0.037107

C -2.831955 -1.493895 -1.103694

C -3.052462 -0.136165 -1.057503

C -0.408922 -2.315053 0.404190

Rh -0.649571 -0.514054 -0.556653

Cl 0.150731 -1.352720 -2.733577

H -3.153084 -1.946430 0.965097

H -3.749132 -3.167483 -0.166882

H 0.516465 -2.721255 -0.011389

H -2.772599 -1.936215 -2.097430

C -2.930337 0.606822 0.223151

C -1.731766 0.478214 0.815960

H -1.458168 1.008757 1.726972

C -3.377630 0.637599 -2.315840

H -4.465946 0.763361 -2.390156

H -2.925604 1.635424 -2.314107

H -3.035288 0.097100 -3.202889

O -3.900270 1.540693 0.627043

C -5.223528 1.210637 0.561761

O -5.640701 0.143690 0.175352

C -6.070895 2.367516 1.036547

H -7.124289 2.090256 0.984335

H -5.804176 2.632244 2.065000

H -5.885878 3.250083 0.415138

H -1.494808 -3.961420 -0.653030

P 1.406053 0.290494 0.087901

C 1.450868 1.251579 1.667492

C 1.880169 2.585908 1.718373

C 1.027323 0.633345 2.856638

C 1.884942 3.284212 2.928871

H 2.214323 3.084916 0.815322

C 1.041241 1.329512 4.064824

H 0.685874 -0.395702 2.837640

C 1.467633 2.659578 4.103916

H 2.219458 4.317949 2.948827

H 0.712833 0.833856 4.974496

H 1.472638 3.203932 5.044342

C 2.807047 -0.901002 0.255653

C 2.990283 -1.876571 -0.740690

C 3.708699 -0.841474 1.330004

C 4.055566 -2.773384 -0.653501

H 2.301001 -1.926517 -1.579902

C 4.769991 -1.745975 1.411592

H 3.589337 -0.091423 2.104152

C 4.944926 -2.714004 0.422054

H 4.186188 -3.522060 -1.430184

H 5.459011 -1.689541 2.250186

H 5.770710 -3.417603 0.487740

C 1.980220 1.506959 -1.172509

C 3.331815 1.640972 -1.519762

C 1.031242 2.336594 -1.790575

C 3.724886 2.587956 -2.467748

H 4.078083 1.003264 -1.056989

C 1.426794 3.284832 -2.733169

H -0.021843 2.238195 -1.539285

C 2.775106 3.410587 -3.075137

H 4.774818 2.678184 -2.732914

H 0.680930 3.916167 -3.208093

H 3.082825 4.143295 -3.816224

C -1.352862 -3.823132 1.612023

H -2.236100 -3.996158 2.237924

H -0.744385 -4.732359 1.596359

C -0.506554 -2.553675 1.931117

H -1.102255 -1.796576 2.447228

H 0.435129 -2.699609 2.472453

**13b***-exo-re*

Atom X Y Z

C -1.640088 -2.916918 1.420473

C -2.943690 -2.423325 0.799801

C -2.759068 -1.801315 -0.564844

C -2.983155 -0.475301 -0.882252

C -0.412767 -1.978367 1.270719

Rh -0.629769 -0.701347 -0.345651

Cl 0.042363 -1.837945 -2.436964

H -3.407964 -1.692184 1.468286

H -3.646608 -3.263140 0.694074

H -0.149090 -1.380584 2.145690

H -2.678833 -2.486692 -1.406331

C -2.896433 0.557223 0.183985

C -1.697784 0.575413 0.786619

H -1.410040 1.274528 1.570446

C -3.292936 -0.049791 -2.298887

H -4.382575 0.014272 -2.422633

H -2.868991 0.931458 -2.536945

H -2.907274 -0.780737 -3.014693

O -3.882568 1.542553 0.356627

C -5.197978 1.175382 0.375580

O -5.585784 0.035300 0.269294

C -6.075628 2.393036 0.542953

H -5.813733 2.922823 1.464686

H -5.917233 3.088124 -0.288466

H -7.121085 2.084457 0.574276

H -1.811941 -3.155544 2.480681

P 1.401832 0.339515 0.063697

C 1.475319 1.221245 1.687881

C 1.266377 2.604651 1.785452

C 1.664196 0.483623 2.870545

C 1.243789 3.234215 3.032653

H 1.125111 3.197007 0.887700

C 1.645816 1.115170 4.114021

H 1.847422 -0.585326 2.821425

C 1.431834 2.492972 4.199185

H 1.083107 4.307578 3.087395

H 1.799994 0.529171 5.016027

H 1.415657 2.984300 5.168164

C 3.000500 -0.582640 -0.003279

C 3.157196 -1.616596 -0.941672

C 4.090603 -0.229687 0.811659

C 4.376357 -2.288560 -1.047988

H 2.329498 -1.885534 -1.591522

C 5.305444 -0.908031 0.700506

H 3.998610 0.575142 1.532803

C 5.450180 -1.941561 -0.226870

H 4.480913 -3.087753 -1.776686

H 6.137682 -0.625082 1.339626

H 6.396047 -2.470392 -0.310301

C 1.600022 1.675669 -1.197943

C 2.851034 2.003202 -1.741441

C 0.463833 2.376836 -1.636507

C 2.962022 3.011960 -2.699902

H 3.739464 1.467553 -1.424614

C 0.579742 3.388903 -2.590009

H -0.514105 2.131730 -1.231060

C 1.829058 3.706636 -3.126062

H 3.937074 3.250570 -3.115987

H -0.308551 3.920577 -2.920139

H 1.918107 4.487913 -3.876007

C 0.465528 -3.216866 0.949533

H 1.148189 -3.151862 0.101733

H 1.037551 -3.534741 1.833364

C -0.824560 -4.050780 0.736489

H -1.059892 -4.160118 -0.326303

H -0.866331 -5.035772 1.212777

**14b**

Atom X Y Z

C -3.834489 -1.932542 0.544196

C -3.245970 -2.351039 -0.802723

C -2.759041 -1.249580 -1.724149

C -2.710932 0.153058 -1.585628

C -2.902418 -1.056077 1.419477

Rh -0.775178 -0.608571 -0.888469

Cl 0.325676 -1.908688 -2.572182

H -4.011597 -2.921604 -1.351352

H -2.418545 -3.059717 -0.655998

H -3.532122 -0.495578 2.126867

H -2.707263 -1.575477 -2.762165

C -2.283629 0.740482 -0.331333

C -1.968931 -0.038755 0.816674

H -1.435689 0.535043 1.573365

C -2.837654 1.041454 -2.804055

H -3.850511 1.465735 -2.841687

H -2.128224 1.871545 -2.787863

H -2.683537 0.459004 -3.716182

O -2.043309 2.126496 -0.333098

C -2.837716 2.891255 0.486251

O -3.759141 2.449110 1.127617

C -2.378839 4.327568 0.457978

H -1.387340 4.399512 0.918660

H -2.289212 4.682322 -0.573354

H -3.088447 4.945260 1.009597

H -4.839560 -1.521607 0.393512

P 1.297389 -0.038995 0.085538

C 1.233572 1.281979 1.384314

C 1.532347 2.617907 1.077133

C 0.793473 0.976163 2.684882

C 1.402119 3.618457 2.044135

H 1.877228 2.880264 0.082366

C 0.665559 1.975781 3.650221

H 0.564400 -0.051834 2.951919

C 0.968657 3.301868 3.332707

H 1.652585 4.645159 1.789260

H 0.330571 1.716519 4.650994

H 0.872304 4.079827 4.085190

C 2.224147 -1.405323 0.922244

C 2.037056 -2.721889 0.469593

C 3.127372 -1.171607 1.973842

C 2.735641 -3.776865 1.060029

H 1.361837 -2.914032 -0.358406

C 3.821224 -2.230339 2.562200

H 3.286182 -0.163729 2.344180

C 3.625233 -3.535960 2.108111

H 2.580974 -4.789192 0.696500

H 4.513636 -2.032371 3.376303

H 4.163846 -4.359945 2.568734

C 2.491403 0.656171 -1.141600

C 3.877661 0.642665 -0.927107

C 1.987704 1.252006 -2.307300

C 4.741389 1.221645 -1.857626

H 4.288379 0.170192 -0.040369

C 2.853227 1.837992 -3.232573

H 0.918597 1.232201 -2.495327

C 4.231019 1.823352 -3.009963

H 5.813904 1.197479 -1.683490

H 2.449995 2.289728 -4.134893

H 4.905677 2.270305 -3.735420

C -2.403310 -2.344947 2.129832

H -1.502586 -2.745780 1.654336

H -2.240288 -2.295653 3.211529

C -3.695097 -3.047755 1.623991

H -3.605090 -4.078394 1.264122

H -4.499482 -3.004447 2.365517

**TS2b***-endo-re*

Imaginary frequency: -186.726 cm-1

Atom X Y Z

C -0.743432 1.085119 -2.087753

C -2.073436 0.524175 -2.023597

Rh -0.668255 -0.091212 -0.345929

C -1.023396 1.310749 1.179429

C -2.344299 1.884200 1.355606

C -3.534305 1.214242 1.202884

Cl -0.318559 -2.234835 -1.586528

H -0.865827 0.464213 1.882260

H -0.233472 2.048248 1.334222

H -2.406598 2.927417 1.670739

C -4.839071 1.822691 1.665064

H -5.273047 1.242724 2.488671

H -5.583506 1.846999 0.860379

H -4.682971 2.845360 2.021258

C -2.613251 -0.610936 -0.259949

H -2.838285 -1.570186 -0.719828

C -3.560789 -0.070970 0.565254

O -4.723111 -0.841964 0.814321

C -5.670201 -0.914121 -0.170540

O -5.620117 -0.289625 -1.203608

C -6.765464 -1.872490 0.231149

H -7.153249 -1.616519 1.221974

H -6.360556 -2.888577 0.291674

H -7.566343 -1.839608 -0.508261

H 0.043755 0.763684 -2.764941

H -2.430471 -0.338548 -2.576403

P 1.726678 -0.030531 0.078648

C 1.840423 -0.870264 1.718792

C 2.644221 1.565075 0.278239

C 2.891588 -0.977945 -0.994798

C 1.418249 -2.212429 1.790399

C 2.204048 -0.206943 2.899935

C 3.971862 1.605226 0.738999

C 2.028926 2.764027 -0.105424

C 3.919308 -1.771247 -0.464750

C 2.786923 -0.843094 -2.386602

C 1.388853 -2.875382 3.017187

H 1.104793 -2.729068 0.886418

C 2.164300 -0.875463 4.126592

H 2.520800 0.830441 2.869660

C 4.653324 2.817540 0.834061

H 4.478743 0.685888 1.016328

C 2.714421 3.978838 -0.016930

H 1.012928 2.741861 -0.482133

C 4.828071 -2.409269 -1.311601

H 4.006526 -1.905777 0.608835

C 3.699081 -1.474960 -3.230203

H 1.977600 -0.262004 -2.814577

C 1.761124 -2.209713 4.188226

H 1.066020 -3.912180 3.056596

H 2.451434 -0.349051 5.033095

C 4.025047 4.008458 0.457761

H 5.678355 2.832199 1.194767

H 2.222295 4.898728 -0.321448

C 4.722510 -2.259694 -2.694669

H 5.616095 -3.025673 -0.886919

H 3.599472 -1.367184 -4.306749

H 1.732330 -2.727314 5.143328

H 4.559504 4.951995 0.528345

H 5.428381 -2.759169 -3.352882

C -1.285840 2.526658 -2.007414

C -2.717940 1.907331 -1.971075

H -3.343845 2.140286 -1.109454

H -3.293132 2.092130 -2.884976

H -1.001178 3.123717 -1.138287

H -1.079067 3.105933 -2.914971

**TS2b***-exo-re*

Imaginary frequency: -204.8709 cm-1

Atom X Y Z

C -0.729339 -0.595541 -2.108213

C -2.112986 -0.789458 -1.749275

Rh -0.740845 -0.240402 -0.036067

C -1.057295 1.817864 -0.321958

C -2.372103 2.308742 -0.707471

C -3.575000 1.829034 -0.254391

Cl -0.518816 -2.423436 1.192091

H -0.887083 1.947454 0.767725

H -0.260740 2.323378 -0.867903

H -2.407148 3.167095 -1.380507

C -4.860970 2.588188 -0.490050

H -5.306285 2.909395 0.459570

H -5.606726 1.972815 -1.006532

H -4.675145 3.482549 -1.092135

C -2.714102 -0.456833 0.300728

H -2.982876 -1.433565 0.696668

C -3.638379 0.548481 0.389801

O -4.824406 0.281454 1.115087

C -5.791573 -0.469926 0.505399

O -5.732557 -0.840254 -0.643424

C -6.917896 -0.751622 1.470060

H -7.729333 -1.256063 0.944408

H -7.277866 0.179078 1.919394

H -6.553525 -1.387487 2.284233

H -0.320276 0.247309 -2.663558

H -2.935830 -0.132516 -2.003932

P 1.685743 0.081984 0.095153

C 2.273889 0.208901 1.842531

C 2.250427 1.670979 -0.667626

C 2.838556 -1.174022 -0.633222

C 1.346077 0.213433 2.893411

C 3.643152 0.303955 2.142792

C 2.251006 1.798577 -2.069097

C 2.562050 2.798943 0.105200

C 4.019399 -0.820235 -1.307305

C 2.547299 -2.535761 -0.442469

C 1.777013 0.329204 4.217469

H 0.288215 0.089564 2.684245

C 4.071196 0.418709 3.464403

H 4.377699 0.280406 1.342900

C 2.572819 3.011611 -2.676746

H 2.013929 0.939432 -2.690502

C 2.876787 4.016650 -0.504858

H 2.564925 2.730303 1.187953

C 4.882300 -1.806271 -1.790768

H 4.273360 0.223135 -1.459998

C 3.416602 -3.515466 -0.924964

H 1.646414 -2.821481 0.093394

C 3.137577 0.434655 4.504692

H 1.046313 0.322439 5.021444

H 5.133501 0.488992 3.682732

C 2.885653 4.127069 -1.894863

H 2.577878 3.085558 -3.761023

H 3.117377 4.877960 0.112640

C 4.582816 -3.156137 -1.603011

H 5.790496 -1.514379 -2.311852

H 3.176720 -4.563706 -0.767715

H 3.472632 0.519100 5.535090

H 3.133544 5.073421 -2.367722

H 5.255539 -3.922141 -1.979870

C -2.034216 -2.297781 -2.018981

H -2.181414 -2.949646 -1.156839

H -2.705977 -2.602681 -2.828879

C -0.550810 -2.081298 -2.465847

H 0.215722 -2.638426 -1.925198

H -0.406278 -2.228009 -3.543602

**TS3b***-endo-re*

Imaginary frequency: -239.1082 cm-1

Atom X Y Z

C 1.212866 -3.290974 -0.336579

C 2.409186 -2.324376 0.957221

C 2.280265 -1.096444 1.786274

C 2.565294 0.186952 1.358752

C 0.000694 -2.685575 -0.908025

Rh 0.483174 -1.125266 0.367937

Cl -0.770216 -2.256151 2.201781

H 3.232546 -2.252758 0.250263

H 2.466614 -3.191352 1.608384

H -1.001329 -3.039979 -0.676138

H 2.030862 -1.246503 2.833529

C 2.700177 0.432390 -0.092313

C 1.775030 -0.141530 -0.866928

H 1.734837 0.007662 -1.944147

C 2.765464 1.318965 2.331443

H 3.830398 1.584070 2.377111

H 2.220367 2.219436 2.026721

H 2.451939 1.036401 3.340897

O 3.678122 1.338967 -0.558053

C 4.986987 0.999750 -0.379819

O 5.353559 -0.032012 0.134190

C 5.889991 2.087644 -0.912344

H 6.931632 1.800035 -0.765687

H 5.693353 2.250403 -1.977141

H 5.686888 3.032150 -0.396726

H 1.150263 -4.162212 0.308661

P -1.244572 0.327324 -0.082194

C -0.822205 1.637840 -1.315665

C -0.625107 2.974258 -0.942680

C -0.611401 1.275547 -2.657966

C -0.235160 3.926171 -1.888938

H -0.779036 3.279677 0.086873

C -0.233639 2.228136 -3.603076

H -0.752977 0.243462 -2.967218

C -0.042265 3.558481 -3.220036

H -0.087895 4.957841 -1.580664

H -0.085225 1.930505 -4.637760

H 0.255847 4.300935 -3.955309

C -2.809246 -0.412508 -0.748378

C -3.305339 -1.580413 -0.142452

C -3.546037 0.178505 -1.787492

C -4.505158 -2.143826 -0.578290

H -2.755552 -2.037539 0.675869

C -4.745010 -0.393180 -2.220695

H -3.192431 1.085569 -2.265379

C -5.226310 -1.556587 -1.620111

H -4.874654 -3.046391 -0.098778

H -5.301001 0.076419 -3.028066

H -6.158542 -2.000820 -1.959108

C -1.848093 1.281673 1.380935

C -3.041981 2.019237 1.316517

C -1.106987 1.287433 2.569423

C -3.475181 2.758078 2.416742

H -3.639504 2.010079 0.409685

C -1.543253 2.027993 3.670564

H -0.205578 0.689040 2.638345

C -2.725197 2.764784 3.596055

H -4.402030 3.322241 2.355512

H -0.964796 2.014687 4.590292

H -3.067658 3.335467 4.455256

C 1.892876 -3.313360 -1.719826

H 2.784941 -2.687090 -1.820579

H 2.135133 -4.325595 -2.059992

C 0.584146 -2.728197 -2.332178

H 0.678301 -1.780522 -2.870711

H 0.057781 -3.440506 -2.978417

**TS3b***-exo-re*

Imaginary frequency: -245.414 cm-1

Atom X Y Z

C 1.259718 -3.028987 -1.155741

C 2.418750 -2.458248 0.397880

C 2.317819 -1.423614 1.459880

C 2.610861 -0.084895 1.270272

C 0.065076 -2.282962 -1.574008

Rh 0.518901 -1.121780 0.095749

Cl -0.725704 -2.435922 1.829940

H 3.278682 -2.265063 -0.238072

H 2.394760 -3.459198 0.821545

H 0.030503 -1.675250 -2.479355

H 2.061767 -1.763843 2.459574

C 2.765530 0.410381 -0.119579

C 1.825946 0.016017 -0.980298

H 1.794317 0.317671 -2.025692

C 2.816662 0.857694 2.426712

H 3.875377 1.142372 2.493671

H 2.243669 1.783547 2.302960

H 2.536717 0.390976 3.375931

O 3.808449 1.318406 -0.411733

C 5.077162 0.816782 -0.445144

O 5.348087 -0.349021 -0.270956

C 6.071615 1.917659 -0.730377

H 5.834431 2.399355 -1.684628

H 6.013367 2.688139 0.045621

H 7.078085 1.499181 -0.764032

H 2.150535 -3.070607 -1.776873

P -1.166817 0.445466 -0.067493

C -0.721613 1.873386 -1.155920

C -0.440711 3.144618 -0.636533

C -0.570491 1.664796 -2.538664

C -0.031661 4.182731 -1.478287

H -0.541565 3.331626 0.427114

C -0.174323 2.704091 -3.379086

H -0.769960 0.684343 -2.962663

C 0.098134 3.968491 -2.849858

H 0.181746 5.161097 -1.056037

H -0.073583 2.524761 -4.446241

H 0.411435 4.778131 -3.503347

C -2.803221 -0.128374 -0.725699

C -3.381010 -1.271400 -0.145712

C -3.515415 0.562825 -1.718335

C -4.637297 -1.712691 -0.561637

H -2.845339 -1.807719 0.632407

C -4.771071 0.111796 -2.133869

H -3.101263 1.456806 -2.170895

C -5.334450 -1.027236 -1.559000

H -5.069910 -2.597644 -0.102505

H -5.307394 0.658313 -2.905371

H -6.311638 -1.376144 -1.882574

C -1.663607 1.247263 1.522537

C -2.782998 2.095167 1.575615

C -0.924976 1.019847 2.689961

C -3.142304 2.715527 2.771166

H -3.379361 2.265621 0.683920

C -1.288387 1.640975 3.887682

H -0.088379 0.331556 2.664377

C -2.393501 2.490554 3.929940

H -4.010878 3.368083 2.800012

H -0.713449 1.447137 4.789125

H -2.678535 2.969201 4.863148

C -0.831899 -3.521596 -1.378950

H -1.670637 -3.424277 -0.689866

H -1.192359 -3.918849 -2.335732

C 0.418750 -4.280302 -0.841276

H 0.366382 -4.555888 0.213835

H 0.716336 -5.151487 -1.435159

**TS4b***-endo-re*

Imaginary frequency: -423.3531 cm-1

Atom X Y Z

C 1.046789 2.161003 0.645096

C 2.185698 1.636889 1.584908

Rh 0.818477 -0.035912 0.037484

C 1.054098 1.737917 -1.330453

C 2.486090 1.848644 -1.741981

C 3.424561 1.020646 -1.263372

Cl 0.827030 -2.341769 0.625019

H 0.547738 0.974583 -1.945876

H 0.482312 2.655880 -1.470061

H 2.765642 2.629933 -2.448397

C 4.865746 1.050113 -1.714332

H 5.134122 0.120125 -2.229088

H 5.548853 1.150693 -0.861528

H 5.040984 1.882154 -2.403367

C 2.675194 0.290706 1.103616

H 2.828757 -0.484015 1.845932

C 3.079404 0.017327 -0.206160

O 3.629567 -1.241244 -0.508211

C 4.577742 -1.818693 0.294260

O 5.144566 -1.249666 1.196686

C 4.788234 -3.250254 -0.126865

H 4.973033 -3.312438 -1.203690

H 3.870439 -3.811144 0.081203

H 5.624982 -3.676748 0.427729

H 0.032766 2.247293 1.033088

H 1.844615 1.525569 2.618903

P -1.566437 -0.163310 -0.047315

C -2.245895 -1.048399 -1.519956

C -2.402194 1.488783 -0.114159

C -2.361309 -0.963395 1.414144

C -1.471543 -2.062501 -2.108274

C -3.511093 -0.757833 -2.059690

C -2.942248 2.079545 1.038958

C -2.403222 2.230227 -1.309691

C -3.656875 -1.498426 1.357665

C -1.663280 -0.992137 2.630391

C -1.958295 -2.770372 -3.209063

H -0.498679 -2.303628 -1.691127

C -3.991144 -1.467649 -3.161303

H -4.120219 0.031046 -1.629165

C -3.471389 3.372165 0.997172

H -2.960155 1.526390 1.972646

C -2.937445 3.518185 -1.350887

H -2.000279 1.793471 -2.218942

C -4.245293 -2.043088 2.499640

H -4.205647 -1.502694 0.421145

C -2.258127 -1.528359 3.773695

H -0.644333 -0.620196 2.667765

C -3.215571 -2.475437 -3.738413

H -1.348791 -3.553210 -3.652271

H -4.970723 -1.230740 -3.568318

C -3.472229 4.094912 -0.196026

H -3.889833 3.808748 1.900224

H -2.938505 4.069702 -2.287436

C -3.549133 -2.055101 3.710312

H -5.246561 -2.461657 2.441320

H -1.703723 -1.550577 4.707986

H -3.589772 -3.025933 -4.597570

H -3.888714 5.097992 -0.228485

H -4.008047 -2.482219 4.598102

C 1.795555 3.530958 0.560402

C 2.994985 2.949357 1.356796

H 3.893414 2.809202 0.752313

H 3.265656 3.486590 2.270995

H 2.029718 3.939580 -0.426416

H 1.236582 4.294906 1.113659

**TS4b***-exo-re*

Imaginary frequency: -446.5746 cm-1

Atom X Y Z

C -1.186795 -2.486749 0.355070

C -2.506871 -2.181992 1.115140

Rh -0.884905 -0.171053 0.169742

C -0.995336 -1.767528 -1.484782

C -2.394992 -1.873913 -1.979823

C -3.358253 -1.068498 -1.504965

Cl -1.031767 1.913479 1.313802

H -0.544102 -0.843903 -1.876867

H -0.330447 -2.572866 -1.788483

H -2.626842 -2.607897 -2.750916

C -4.766781 -1.036840 -2.046644

H -4.993256 -0.058938 -2.488599

H -5.505109 -1.205243 -1.252688

H -4.903344 -1.798429 -2.820699

C -2.819429 -0.713347 0.953200

H -3.048134 -0.120484 1.832160

C -3.084902 -0.195221 -0.318965

O -3.600235 1.099994 -0.482099

C -4.606839 1.571562 0.322227

O -5.263328 0.880550 1.063542

C -4.753491 3.058200 0.126922

H -5.643155 3.411378 0.649832

H -4.813106 3.304559 -0.937595

H -3.859922 3.548107 0.529466

H -1.225948 -3.451477 -0.149199

H -3.365554 -2.817257 0.861038

P 1.498194 0.184281 -0.053252

C 1.955229 1.807197 -0.802237

C 2.320402 -1.045844 -1.167680

C 2.502469 0.092200 1.498106

C 0.979632 2.529829 -1.504723

C 3.259265 2.320377 -0.719265

C 2.444173 -2.382266 -0.745738

C 2.726938 -0.713732 -2.468277

C 3.813997 -0.411951 1.518989

C 1.939868 0.574797 2.692276

C 1.308638 3.734727 -2.129465

H -0.040832 2.160501 -1.534495

C 3.582675 3.527662 -1.338532

H 4.022608 1.784228 -0.163290

C 2.972766 -3.352964 -1.595937

H 2.135744 -2.663829 0.257102

C 3.247851 -1.689925 -3.322504

H 2.643071 0.310351 -2.817022

C 4.544893 -0.436257 2.708342

H 4.267488 -0.793853 0.609941

C 2.677458 0.550538 3.877243

H 0.932985 0.981667 2.683995

C 2.608716 4.234954 -2.047995

H 0.542334 4.287819 -2.665687

H 4.594293 3.917679 -1.262887

C 3.374488 -3.009726 -2.890074

H 3.070819 -4.377900 -1.247613

H 3.559231 -1.412184 -4.326002

C 3.978187 0.043629 3.890402

H 5.557557 -0.831131 2.708198

H 2.229545 0.929291 4.792093

H 2.861898 5.177008 -2.527283

H 3.783936 -3.766497 -3.553707

H 4.547623 0.022676 4.815901

C -1.776905 -2.566446 2.435528

H -1.843652 -1.825711 3.238175

H -2.105296 -3.535669 2.820951

C -0.405330 -2.640180 1.702248

H 0.275574 -1.832957 1.973990

H 0.130017 -3.593125 1.795864

**TS5b***-endo-re*

Imaginary frequency: -330.7794 cm-1

Atom X Y Z

C -1.846604 -2.846695 -0.774570

C -2.984603 -2.076757 -1.456830

C -2.555770 -0.695180 -1.935571

C -2.781603 0.486094 -1.215232

C -1.195709 -1.973554 0.319176

Rh -0.592074 -0.174709 -0.884455

Cl 0.235351 0.762616 -2.930354

H -3.822090 -1.957032 -0.759615

H -3.360920 -2.649281 -2.312947

H -0.149317 -2.226358 0.496765

H -2.419486 -0.572312 -3.007566

C -2.809235 0.447122 0.254074

C -1.721877 -0.156729 0.801933

H -1.488837 -0.127030 1.862597

C -3.064857 1.794070 -1.917099

H -4.148710 1.900652 -2.063786

H -2.712274 2.654577 -1.339654

H -2.581645 1.809137 -2.897122

O -3.687098 1.247049 0.998160

C -5.030678 1.041500 0.827881

O -5.493890 0.171977 0.128356

C -5.819262 2.050716 1.626047

H -5.640018 3.056844 1.231283

H -6.881943 1.814918 1.560771

H -5.494215 2.048131 2.671107

H -1.147261 -3.224757 -1.527194

P 1.520732 0.137050 0.106421

C 1.531843 -0.141503 1.939904

C 1.334462 0.928270 2.827341

C 1.613987 -1.442033 2.468498

C 1.225360 0.704500 4.201535

H 1.272024 1.942705 2.446211

C 1.507427 -1.663848 3.842534

H 1.785271 -2.287699 1.808738

C 1.310870 -0.590761 4.714183

H 1.078726 1.547823 4.871195

H 1.583955 -2.676292 4.230329

H 1.229527 -0.762858 5.783980

C 2.883719 -0.950603 -0.504275

C 2.800482 -1.463477 -1.809318

C 4.007244 -1.265903 0.280426

C 3.820961 -2.271938 -2.314535

H 1.946567 -1.210458 -2.429878

C 5.023160 -2.075683 -0.229354

H 4.086791 -0.890976 1.296196

C 4.931435 -2.581333 -1.527936

H 3.743803 -2.658762 -3.327046

H 5.884374 -2.312017 0.390154

H 5.722111 -3.213788 -1.923016

C 2.157768 1.862203 -0.053610

C 3.522284 2.169437 0.053753

C 1.237596 2.902887 -0.248561

C 3.955113 3.493693 -0.022062

H 4.252733 1.377374 0.183388

C 1.672438 4.227595 -0.314256

H 0.185336 2.666619 -0.371978

C 3.031323 4.525418 -0.201628

H 5.015791 3.717652 0.055097

H 0.949416 5.023535 -0.470771

H 3.371034 5.555946 -0.263140

C -2.287264 -3.841582 0.333911

H -3.303721 -4.244543 0.263588

H -1.585485 -4.673622 0.449401

C -2.062087 -2.720287 1.390682

H -2.994269 -2.208475 1.639556

H -1.545481 -2.984028 2.319170

**TS5b***-exo-re*

Imaginary frequency: -333.8465 cm-1

Atom X Y Z

C -2.522792 -2.668059 0.731916

C -2.915200 -2.602465 -0.757877

C -2.567841 -1.323855 -1.502719

C -2.877783 -0.000771 -1.124137

C -1.311016 -1.759597 1.032189

Rh -0.659527 -0.496729 -0.717484

Cl 0.095736 -0.270391 -2.993993

H -3.996452 -2.777326 -0.860079

H -2.429765 -3.422299 -1.300473

H -1.177385 -1.650594 2.111681

H -2.515095 -1.465415 -2.580812

C -2.846095 0.412869 0.275207

C -1.726966 0.010398 0.947727

H -1.440283 0.454630 1.895887

C -3.277374 1.013904 -2.171260

H -4.360539 0.957731 -2.341889

H -3.029164 2.036023 -1.867869

H -2.765042 0.794158 -3.111571

O -3.658573 1.433805 0.767972

C -5.019883 1.306932 0.637832

O -5.560638 0.347885 0.143229

C -5.708094 2.528122 1.195442

H -5.401244 2.695675 2.232757

H -5.416499 3.414718 0.622097

H -6.788586 2.391885 1.140682

H -3.391695 -2.520169 1.381338

P 1.450770 0.221055 0.073912

C 1.477090 0.642692 1.879287

C 1.442280 1.974588 2.319466

C 1.421168 -0.380933 2.842122

C 1.358360 2.273887 3.681680

H 1.484790 2.783912 1.598063

C 1.344643 -0.080983 4.202698

H 1.455249 -1.420187 2.528965

C 1.311000 1.249452 4.627501

H 1.336193 3.312581 4.000779

H 1.313760 -0.887823 4.930378

H 1.250567 1.483883 5.686801

C 2.838469 -0.985608 -0.119591

C 2.834160 -1.816929 -1.252739

C 3.900421 -1.085671 0.794582

C 3.871321 -2.727413 -1.461218

H 2.025968 -1.733710 -1.974016

C 4.932268 -2.002615 0.583266

H 3.923569 -0.454445 1.677427

C 4.919401 -2.826141 -0.543881

H 3.856314 -3.361349 -2.343780

H 5.745865 -2.070732 1.300861

H 5.722784 -3.539933 -0.706362

C 2.078608 1.767098 -0.716705

C 3.436716 2.117977 -0.674337

C 1.169518 2.631037 -1.343867

C 3.874186 3.315383 -1.240856

H 4.157545 1.452961 -0.208521

C 1.608993 3.832353 -1.903399

H 0.125115 2.344896 -1.413303

C 2.960621 4.176259 -1.853698

H 4.929599 3.572735 -1.207398

H 0.895456 4.489565 -2.393034

H 3.303532 5.106884 -2.298283

C -0.371004 -3.013724 0.685718

H -0.090494 -3.099563 -0.365027

H 0.526484 -3.119257 1.300640

C -1.605306 -3.885707 1.033709

H -1.766723 -4.786368 0.431287

H -1.619736 -4.157859 2.094783

**10c***-endo-re*

Atom X Y Z

C 0.391588 1.473529 1.791036

C 1.782242 1.245635 1.726502

Rh 0.735780 -0.060850 0.243019

C 1.152734 0.785959 -1.622039

C 2.471298 1.256024 -1.980319

C 3.651982 0.679001 -1.581364

Cl 0.349792 -1.777277 2.052508

H 1.014344 -0.289287 -1.917812

H 0.366038 1.375496 -2.090999

H 2.542156 2.120661 -2.641573

C 4.972619 1.121344 -2.164576

H 5.443311 0.311305 -2.735272

H 5.679024 1.416312 -1.379951

H 4.832203 1.971269 -2.839014

C 2.627433 -0.676879 0.266392

H 2.831949 -1.431280 1.024979

C 3.639918 -0.356774 -0.584926

O 4.833430 -1.122734 -0.496479

C 5.745508 -0.772095 0.457533

O 5.642343 0.200323 1.168272

C 6.881931 -1.766495 0.483009

H 7.293736 -1.903015 -0.521703

H 6.509802 -2.740716 0.818315

H 7.657249 -1.415307 1.164811

H -0.238188 0.937601 2.494595

H 2.278686 0.545214 2.388569

P -1.733437 -0.136320 -0.124834

C -1.920874 -1.551484 -1.304076

C -2.742375 1.216194 -0.896837

C -2.800720 -0.539858 1.329696

C -1.303429 -2.776612 -0.982039

C -2.569525 -1.424695 -2.542626

C -4.142582 1.102180 -0.977641

C -2.137377 2.377038 -1.398066

C -3.339197 -1.811445 1.559020

C -3.065858 0.485917 2.252351

C -1.362077 -3.850002 -1.872170

H -0.777349 -2.884400 -0.036646

C -2.612590 -2.500054 -3.433072

H -3.043412 -0.489514 -2.819877

C -4.907901 2.114227 -1.555046

H -4.635169 0.219490 -0.580960

C -2.904650 3.394331 -1.973489

H -1.063032 2.495921 -1.329295

C -4.123329 -2.052901 2.689118

H -3.148051 -2.619069 0.861620

C -3.847044 0.241859 3.380739

H -2.673784 1.485489 2.084467

C -2.013546 -3.715411 -3.100079

H -0.887410 -4.790171 -1.604506

H -3.118197 -2.383698 -4.388150

C -4.290193 3.264017 -2.055614

H -5.988022 2.007574 -1.609167

H -2.415859 4.288086 -2.351945

C -4.377140 -1.030941 3.603462

H -4.531964 -3.046318 2.853477

H -4.042335 1.046557 4.084643

H -2.050264 -4.550694 -3.794356

H -4.888124 4.054387 -2.501273

H -4.984344 -1.223041 4.483899

C 1.371433 3.395992 0.692057

H 1.571295 4.459797 0.854650

C 0.096200 2.916816 1.421811

C 2.496649 2.488718 1.242235

H 3.291633 2.286820 0.520580

H 2.976476 2.946226 2.121163

H 1.265893 3.241938 -0.386168

H -0.822428 3.066469 0.848411

H -0.033860 3.467117 2.368301

**10c***-exo-re*

Atom X Y Z

C 0.586091 1.019515 -1.966020

C 1.910680 1.260215 -1.538678

Rh 0.790577 0.242152 0.074246

C 1.082215 -1.671893 -0.754974

C 2.373045 -2.018225 -1.332091

C 3.587224 -1.696836 -0.789137

Cl 0.524624 1.765059 2.047378

H 0.959423 -2.104753 0.257126

H 0.254360 -1.988834 -1.389934

H 2.375879 -2.605366 -2.251418

C 4.871234 -2.281782 -1.326567

H 5.374199 -2.890013 -0.564794

H 5.575190 -1.497852 -1.630142

H 4.672364 -2.921181 -2.191748

C 2.702556 0.174185 0.628640

H 2.969531 0.921831 1.374141

C 3.654967 -0.732498 0.281139

O 4.876813 -0.717414 1.003340

C 5.805200 0.229972 0.674954

O 5.686645 1.005474 -0.244355

C 6.982236 0.153336 1.618193

H 7.754855 0.850849 1.292931

H 7.380980 -0.865528 1.648144

H 6.658329 0.406690 2.633390

H 0.331664 0.182394 -2.610194

H 2.738756 0.624735 -1.824618

P -1.712101 -0.144537 0.108010

C -2.206922 -1.027843 1.658259

C -2.274667 -1.293030 -1.232108

C -2.939334 1.242612 0.028733

C -1.242743 -1.351954 2.622840

C -3.546980 -1.381088 1.892496

C -2.403352 -0.811669 -2.548245

C -2.455130 -2.666229 -1.004600

C -4.178661 1.123608 -0.623087

C -2.638020 2.437869 0.703821

C -1.606987 -2.032033 3.788276

H -0.212133 -1.041382 2.487402

C -3.908429 -2.058967 3.055382

H -4.312361 -1.121685 1.167024

C -2.718375 -1.674078 -3.598201

H -2.274183 0.246888 -2.753089

C -2.764844 -3.530034 -2.058635

H -2.358145 -3.066542 -0.000951

C -5.091763 2.179644 -0.606643

H -4.436317 0.210880 -1.149817

C -3.557455 3.488110 0.718791

H -1.688973 2.535754 1.222763

C -2.937042 -2.389360 4.004940

H -0.848122 -2.268833 4.528817

H -4.948850 -2.324568 3.222992

C -2.898935 -3.038410 -3.356877

H -2.824349 -1.278593 -4.604959

H -2.903053 -4.589309 -1.858998

C -4.783589 3.364819 0.063096

H -6.045243 2.071760 -1.117272

H -3.309574 4.404988 1.246850

H -3.220243 -2.914816 4.913115

H -3.142965 -3.710580 -4.174948

H -5.495812 4.185713 0.074919

C 0.706915 3.346662 -1.294969

H 0.673761 4.357281 -1.713602

C 2.130564 2.744357 -1.310510

H 2.706971 2.968616 -0.409879

H 2.711208 3.118398 -2.168514

C -0.147903 2.346496 -2.107417

H -1.195241 2.330959 -1.794115

H -0.138800 2.605738 -3.179268

H 0.345389 3.394263 -0.264677

**11c***-endo-re*

Atom X Y Z

C -0.526208 -3.457796 -0.379468

C -2.066705 -1.765651 -1.999004

C -1.976526 -0.341280 -2.235935

C -2.363346 0.625869 -1.306361

C 0.508547 -2.909267 0.386378

Rh -0.464760 -1.249724 -0.738493

Cl 1.129176 -1.743797 -2.605777

H -2.914601 -2.124674 -1.417695

H -1.780236 -2.399629 -2.834544

H 1.527004 -2.846226 0.021862

H -1.580847 0.001377 -3.189153

C -2.680315 0.209040 0.062433

C -1.884375 -0.733097 0.596813

H -2.012858 -1.049835 1.629967

C -2.455918 2.077182 -1.686527

H -3.505758 2.319911 -1.896537

H -2.117570 2.729755 -0.875162

H -1.875163 2.290884 -2.587886

O -3.639444 0.912203 0.818393

C -4.900279 1.042207 0.314871

O -5.237527 0.641364 -0.775676

C -5.788840 1.757388 1.304379

H -5.361446 2.732611 1.559427

H -6.782591 1.885294 0.873940

H -5.856517 1.179362 2.232042

H -0.365536 -3.833134 -1.384440

P 1.167465 0.462952 0.154866

C 0.456196 1.584943 1.444540

C 0.266350 2.958314 1.226340

C -0.004132 1.034015 2.654117

C -0.350826 3.756884 2.193332

H 0.607479 3.413153 0.302817

C -0.606355 1.833863 3.624731

H 0.115437 -0.028611 2.844380

C -0.783970 3.200286 3.396389

H -0.483847 4.818929 2.004009

H -0.942377 1.387248 4.556877

H -1.257792 3.823680 4.149836

C 2.713718 -0.207858 0.925012

C 3.495865 -1.069334 0.133134

C 3.147541 0.104228 2.221248

C 4.681619 -1.604574 0.635456

H 3.172048 -1.316545 -0.875190

C 4.334945 -0.439612 2.720286

H 2.569861 0.777560 2.845508

C 5.103727 -1.294838 1.931139

H 5.275543 -2.266554 0.010807

H 4.658193 -0.186251 3.726774

H 6.027034 -1.715396 2.320806

C 1.876898 1.643275 -1.080790

C 2.959534 2.473616 -0.742297

C 1.336998 1.725395 -2.370532

C 3.474221 3.378928 -1.669186

H 3.404504 2.409002 0.246448

C 1.854986 2.632316 -3.298984

H 0.541402 1.049310 -2.658636

C 2.920297 3.461774 -2.949895

H 4.312211 4.014087 -1.394239

H 1.434593 2.674946 -4.300144

H 3.326970 4.162462 -3.674417

C -1.195478 -3.691401 1.958828

H -1.223180 -4.571630 2.609090

C -1.608257 -4.051813 0.503140

H -2.616388 -3.700737 0.253836

H -1.614760 -5.140405 0.351519

C 0.241249 -3.101722 1.870027

H 0.362062 -2.187737 2.461461

H 0.984079 -3.812335 2.259582

H -1.891916 -2.968206 2.391022

**11c***-exo-re*

Atom X Y Z

C 0.366606 3.209285 1.123201

C 1.879414 2.520141 -1.145663

C 2.023664 1.257131 -1.850323

C 2.587269 0.119381 -1.277533

C -0.596863 2.307531 1.600603

Rh 0.344709 1.439015 -0.228008

Cl -1.144606 2.221348 -2.102075

H 2.674928 2.778446 -0.447628

H 1.509799 3.349952 -1.742828

H -0.379740 1.569302 2.368491

H 1.703351 1.201849 -2.887503

C 2.753699 0.076000 0.175130

C 1.790034 0.650268 0.919942

H 1.810369 0.596888 2.009702

C 2.993768 -1.058501 -2.115581

H 4.079723 -1.022707 -2.275485

H 2.761120 -2.010056 -1.626106

H 2.506803 -1.034563 -3.093458

O 3.776386 -0.709481 0.746129

C 5.070341 -0.421285 0.416091

O 5.388927 0.432020 -0.378727

C 6.017540 -1.318657 1.176002

H 5.890982 -1.169803 2.253464

H 5.794554 -2.369521 0.963749

H 7.044019 -1.090170 0.887567

H 1.386838 3.236375 1.492385

P -1.028647 -0.695345 0.075566

C -0.508998 -1.546999 1.629581

C 0.624416 -2.376512 1.664914

C -1.136482 -1.226219 2.846973

C 1.095671 -2.893467 2.873270

H 1.143155 -2.625456 0.745352

C -0.661414 -1.739980 4.054645

H -2.012331 -0.584426 2.851873

C 0.454583 -2.578986 4.072120

H 1.969082 -3.540035 2.873938

H -1.168685 -1.485968 4.981663

H 0.821547 -2.982267 5.012023

C -2.882013 -0.692253 0.186919

C -3.602828 0.326546 -0.454620

C -3.587491 -1.734674 0.813129

C -5.000634 0.308000 -0.453312

H -3.069884 1.119120 -0.971792

C -4.981931 -1.744814 0.815775

H -3.051742 -2.540379 1.305358

C -5.692877 -0.721135 0.184135

H -5.544132 1.103679 -0.955837

H -5.512579 -2.555673 1.308237

H -6.779745 -0.731233 0.185826

C -0.779876 -1.939321 -1.277226

C -0.846274 -3.327392 -1.075419

C -0.618657 -1.448831 -2.583374

C -0.727544 -4.205729 -2.153580

H -0.989388 -3.729071 -0.077110

C -0.514526 -2.331714 -3.660895

H -0.600051 -0.375561 -2.755111

C -0.560488 -3.710429 -3.448788

H -0.773477 -5.277921 -1.980732

H -0.400365 -1.937077 -4.667172

H -0.473873 -4.396220 -4.287488

C -1.809423 4.174286 0.622967

H -2.412494 5.019240 0.970973

C -1.981944 2.926582 1.524568

H -2.756762 2.244818 1.163963

H -2.266205 3.213836 2.549895

C -0.292956 4.491964 0.646638

H 0.076570 4.844476 -0.321442

H -0.059136 5.275934 1.383737

H -2.111381 3.934151 -0.397299

**12c***-endo-re*

Atom X Y Z

C 0.849006 1.669220 1.350720

C 2.363671 1.646518 1.642658

Rh 0.771084 -0.038767 0.144857

C 0.867672 1.110636 -1.586466

C 2.266083 1.469765 -1.985591

C 3.357540 0.954733 -1.405887

Cl 0.857139 -2.122239 1.527978

H 0.450219 0.441259 -2.359660

H 0.234484 2.003532 -1.575679

H 2.396818 2.169525 -2.816772

C 4.772475 1.198635 -1.878379

H 5.240677 0.266193 -2.217271

H 5.405313 1.598006 -1.075684

H 4.784788 1.905585 -2.713623

C 2.851512 0.327492 1.044898

H 2.975126 -0.477622 1.759689

C 3.198114 0.038533 -0.248321

O 3.663453 -1.252888 -0.549844

C 4.643963 -1.856126 0.209159

O 5.314934 -1.275630 1.024703

C 4.732500 -3.316714 -0.147148

H 4.729711 -3.454520 -1.232149

H 3.847818 -3.824199 0.254576

H 5.634130 -3.745717 0.291963

H 0.238815 1.335127 2.195281

H 2.541769 1.559465 2.721640

P -1.556194 -0.189142 -0.074527

C -2.007261 -1.477001 -1.324666

C -2.436493 1.338309 -0.636094

C -2.528864 -0.663366 1.425758

C -1.200130 -2.625036 -1.408741

C -3.134595 -1.376952 -2.157301

C -3.096684 2.158806 0.293083

C -2.384155 1.755279 -1.978335

C -3.884835 -1.011223 1.302395

C -1.944690 -0.648083 2.699060

C -1.513810 -3.642190 -2.311851

H -0.343350 -2.733618 -0.749452

C -3.441853 -2.397282 -3.059130

H -3.774154 -0.501566 -2.111603

C -3.690936 3.357000 -0.107939

H -3.154641 1.860444 1.334622

C -2.984858 2.949568 -2.378205

H -1.883418 1.141133 -2.719166

C -4.640372 -1.326377 2.430329

H -4.354064 -1.043981 0.323810

C -2.706392 -0.958813 3.828653

H -0.888107 -0.437537 2.805382

C -2.630809 -3.530513 -3.141114

H -0.880854 -4.523920 -2.362684

H -4.316877 -2.303740 -3.696961

C -3.638949 3.755586 -1.444081

H -4.198742 3.975165 0.627471

H -2.940640 3.248374 -3.422105

C -4.052863 -1.296347 3.697995

H -5.686454 -1.598796 2.318495

H -2.236591 -0.951140 4.808269

H -2.870516 -4.322638 -3.845483

H -4.106153 4.685474 -1.756653

H -4.641815 -1.544331 4.577065

C 1.801312 3.725069 0.389265

H 1.829839 4.808028 0.554286

C 0.500041 3.097580 0.924884

C 2.945147 2.997020 1.133717

H 3.828097 2.860435 0.503396

H 3.265610 3.585773 2.001110

H 1.889347 3.562525 -0.687804

H -0.333900 3.163797 0.220392

H 0.180480 3.638989 1.830216

**12c***-exo-re*

Atom X Y Z

C 0.880523 -2.176854 0.120605

C 2.419020 -2.327660 0.055880

Rh 0.803061 -0.098378 0.049911

C 0.725472 -0.231154 2.129464

C 2.076174 -0.361004 2.765053

C 3.228119 -0.167989 2.110134

Cl 1.158025 1.229196 -2.032099

H 0.280888 0.728685 2.442984

H 0.053816 -1.011949 2.503612

H 2.113730 -0.585540 3.835025

C 4.592932 -0.161796 2.758045

H 5.088778 0.807175 2.621751

H 5.251900 -0.921841 2.318136

H 4.513145 -0.354458 3.832285

C 2.897926 -0.920378 -0.291960

H 3.105554 -0.705945 -1.333181

C 3.198382 0.040662 0.639366

O 3.711390 1.283656 0.277955

C 4.583852 1.448818 -0.784724

O 5.205729 0.548308 -1.286369

C 4.626324 2.900194 -1.176377

H 5.462115 3.072263 -1.855831

H 4.708334 3.541251 -0.293891

H 3.681617 3.138475 -1.679079

H 0.431873 -2.557317 1.040163

H 2.849568 -2.651614 1.008009

P -1.530160 0.175790 0.027275

C -2.004181 1.949399 0.230918

C -2.395077 -0.685564 1.414465

C -2.496165 -0.356233 -1.461738

C -1.015107 2.905218 0.503182

C -3.341226 2.365239 0.118502

C -2.476359 -2.089940 1.398328

C -2.886572 0.003003 2.532532

C -3.793686 -0.885529 -1.352062

C -1.944314 -0.165767 -2.739704

C -1.357658 4.248457 0.676122

H 0.027508 2.607030 0.552681

C -3.680720 3.706346 0.291020

H -4.118552 1.642859 -0.112396

C -3.044091 -2.782502 2.466843

H -2.104980 -2.644987 0.541921

C -3.449848 -0.693865 3.605271

H -2.835806 1.086064 2.569386

C -4.520494 -1.220929 -2.495852

H -4.240411 -1.043590 -0.376271

C -2.677857 -0.502379 -3.878633

H -0.946978 0.251885 -2.839115

C -2.689255 4.650231 0.573168

H -0.579401 4.978556 0.880064

H -4.718501 4.015434 0.199127

C -3.531179 -2.085621 3.576076

H -3.105213 -3.867020 2.432975

H -3.826776 -0.142831 4.462744

C -3.964077 -1.031951 -3.761852

H -5.521887 -1.630912 -2.393588

H -2.236480 -0.349371 -4.859760

H -2.955137 5.695865 0.703365

H -3.971362 -2.625727 4.409871

H -4.530292 -1.295619 -4.651324

C 1.533467 -3.070936 -2.076804

H 1.386195 -3.897400 -2.780341

C 2.694105 -3.316472 -1.096603

H 3.684391 -3.177111 -1.545504

H 2.647121 -4.342956 -0.709496

C 0.321520 -2.854325 -1.148429

H -0.481863 -2.295842 -1.637209

H -0.100100 -3.831476 -0.861919

H 1.718771 -2.166234 -2.668751

**13c***-endo-re*

Atom X Y Z

C 1.689739 3.077396 -0.486043

C 2.968487 2.221986 -0.520165

C 2.798569 1.045822 -1.445036

C 2.969899 -0.289286 -1.159172

C 0.511233 2.264631 0.083266

Rh 0.609758 0.327081 -0.671702

Cl -0.240474 0.939586 -2.908921

H 3.239109 1.881076 0.483396

H 3.802970 2.829663 -0.901267

H -0.426081 2.615486 -0.354508

H 2.722679 1.301893 -2.501173

C 2.857219 -0.790225 0.233707

C 1.677394 -0.510781 0.809830

H 1.393955 -0.863596 1.800765

C 3.216159 -1.290215 -2.266017

H 4.293076 -1.494433 -2.332444

H 2.708313 -2.243257 -2.080384

H 2.885928 -0.893800 -3.230460

O 3.801446 -1.676756 0.779075

C 5.133475 -1.417467 0.630166

O 5.578742 -0.458789 0.043072

C 5.950585 -2.498789 1.297181

H 5.729121 -3.471158 0.844466

H 7.011392 -2.271237 1.186967

H 5.690174 -2.567330 2.358430

H 1.462523 3.395943 -1.509875

P -1.465473 -0.299323 0.120705

C -1.460067 -1.114864 1.782726

C -1.570013 -2.507820 1.909926

C -1.261489 -0.348408 2.944562

C -1.487333 -3.116837 3.164458

H -1.722483 -3.123655 1.030430

C -1.186591 -0.958034 4.197072

H -1.172958 0.729867 2.873834

C -1.297246 -2.345739 4.311047

H -1.575972 -4.197184 3.241131

H -1.039568 -0.346568 5.083355

H -1.235425 -2.820694 5.286375

C -2.819322 0.952601 0.216310

C -2.954307 1.869729 -0.841284

C -3.745816 0.985194 1.271396

C -3.989763 2.805047 -0.829608

H -2.258026 1.837289 -1.675315

C -4.777383 1.926846 1.276713

H -3.670419 0.277924 2.090019

C -4.899641 2.840606 0.229227

H -4.082380 3.507025 -1.653859

H -5.485385 1.941237 2.101165

H -5.702224 3.573421 0.235906

C -2.152633 -1.609528 -0.983337

C -3.532507 -1.846069 -1.071605

C -1.275316 -2.413014 -1.726772

C -4.022007 -2.869089 -1.884712

H -4.227598 -1.227560 -0.512353

C -1.766084 -3.439136 -2.534768

H -0.205132 -2.229544 -1.688714

C -3.140717 -3.667927 -2.615990

H -5.093564 -3.037794 -1.949329

H -1.074776 -4.048227 -3.110468

H -3.524229 -4.461048 -3.252150

C 1.507744 3.672049 1.871907

H 1.106086 4.411439 2.573086

C 1.788491 4.276302 0.480309

H 2.756496 4.788788 0.423421

H 1.015271 5.012814 0.226116

C 0.518610 2.498343 1.615842

H 0.827844 1.609149 2.167956

H -0.492625 2.754374 1.957171

H 2.437220 3.292329 2.311973

**13c***-exo-re*

Atom X Y Z

C -2.255031 -2.396646 0.637593

C -3.031658 -2.230281 -0.683046

C -2.715405 -0.992055 -1.501744

C -2.883685 0.339329 -1.165018

C -0.791547 -1.960873 0.455497

Rh -0.603911 -0.254254 -0.712602

Cl 0.177817 -0.291036 -3.046530

H -4.110779 -2.237843 -0.477281

H -2.827840 -3.093825 -1.327178

H -0.352213 -1.770097 1.439072

H -2.623072 -1.173116 -2.571411

C -2.832829 0.813114 0.243577

C -1.638307 0.587756 0.796967

H -1.328696 0.880691 1.798284

C -3.098561 1.379439 -2.242810

H -4.175383 1.578415 -2.336106

H -2.604316 2.326730 -2.002428

H -2.729928 1.024866 -3.208694

O -3.834901 1.631023 0.792793

C -5.135831 1.232975 0.684667

O -5.489336 0.202573 0.158943

C -6.049605 2.260462 1.309506

H -5.787255 2.406265 2.362538

H -5.929413 3.226280 0.807462

H -7.083721 1.924592 1.226123

H -2.743919 -1.810701 1.419337

P 1.497162 0.290547 0.132315

C 1.540828 0.493513 1.970875

C 1.401917 1.754859 2.569175

C 1.620685 -0.639223 2.800806

C 1.343586 1.880627 3.959292

H 1.344961 2.645272 1.951756

C 1.565661 -0.510744 4.188931

H 1.749048 -1.625451 2.365070

C 1.423986 0.750128 4.772766

H 1.239709 2.866941 4.403489

H 1.636119 -1.397463 4.813145

H 1.381049 0.849750 5.853898

C 2.948951 -0.798049 -0.212503

C 2.977619 -1.557691 -1.393222

C 4.054532 -0.844458 0.655695

C 4.087275 -2.351447 -1.690567

H 2.141663 -1.509980 -2.083703

C 5.159563 -1.641172 0.352438

H 4.056546 -0.262441 1.571576

C 5.177030 -2.399096 -0.820194

H 4.094583 -2.933404 -2.608124

H 6.005321 -1.667906 1.034445

H 6.036968 -3.021257 -1.053991

C 1.989093 1.948550 -0.514966

C 3.330520 2.297902 -0.728129

C 0.987026 2.891080 -0.796844

C 3.661232 3.566413 -1.207107

H 4.119066 1.578960 -0.532064

C 1.321396 4.160784 -1.268956

H -0.057239 2.627055 -0.654702

C 2.659424 4.500278 -1.476792

H 4.704277 3.821372 -1.374249

H 0.534714 4.878111 -1.486217

H 2.919688 5.485462 -1.854301

C -0.875167 -4.414539 0.281949

H -0.246950 -5.026783 0.937741

C -0.105685 -3.156903 -0.231882

H -0.195705 -3.075187 -1.320713

H 0.965221 -3.199555 -0.011666

C -2.119792 -3.880106 1.048952

H -3.027127 -4.457310 0.834482

H -1.951914 -3.944086 2.131036

H -1.167201 -5.055854 -0.556561

**14c**

Atom X Y Z

C -4.051921 -1.148191 0.093389

C -3.503607 -1.560990 -1.276804

C -2.679983 -0.528372 -2.020304

C -2.423324 0.825616 -1.710887

C -2.955732 -0.786990 1.110745

Rh -0.693246 -0.311246 -1.029778

Cl 0.302528 -1.610642 -2.775001

H -4.360029 -1.809732 -1.923839

H -2.923577 -2.491120 -1.202461

H -3.467701 -0.299386 1.957201

H -2.592145 -0.753349 -3.082533

C -2.031788 1.172205 -0.360755

C -1.897656 0.207086 0.676859

H -1.333228 0.598315 1.521914

C -2.312576 1.861581 -2.806806

H -3.259144 2.414781 -2.882356

H -1.516562 2.585153 -2.618202

H -2.132148 1.374540 -3.768973

O -1.608190 2.495606 -0.146932

C -2.348081 3.244695 0.735648

O -3.359720 2.851833 1.262859

C -1.704712 4.594150 0.933188

H -0.734825 4.464826 1.425937

H -1.521627 5.075943 -0.032307

H -2.353578 5.218082 1.548840

H -4.727658 -0.295650 -0.050792

P 1.388775 -0.158580 0.096252

C 1.445351 1.029239 1.517872

C 1.957850 2.325940 1.362127

C 0.889435 0.673331 2.759935

C 1.921355 3.238919 2.419502

H 2.397046 2.624005 0.415771

C 0.854313 1.585437 3.815589

H 0.499008 -0.329311 2.910850

C 1.369791 2.872926 3.648657

H 2.336623 4.234128 2.282133

H 0.426856 1.287601 4.769300

H 1.346589 3.581965 4.471772

C 2.048323 -1.722304 0.834371

C 1.676266 -2.948335 0.258843

C 2.928601 -1.726893 1.930523

C 2.174603 -4.148140 0.770603

H 1.015131 -2.958434 -0.602057

C 3.422217 -2.929128 2.439515

H 3.225009 -0.792495 2.396831

C 3.045107 -4.142900 1.861407

H 1.879145 -5.087922 0.311943

H 4.100468 -2.915057 3.288737

H 3.428467 -5.078621 2.259783

C 2.746078 0.451885 -0.999527

C 4.099705 0.202719 -0.728015

C 2.407315 1.220090 -2.123344

C 5.093327 0.719061 -1.560518

H 4.382526 -0.405385 0.125398

C 3.403324 1.742640 -2.950048

H 1.360483 1.385024 -2.359157

C 4.747715 1.492457 -2.670877

H 6.137908 0.511814 -1.343455

H 3.125707 2.329912 -3.821187

H 5.523100 1.890195 -3.320329

C -3.673861 -3.092565 1.578764

H -4.017477 -3.339332 2.588912

C -2.434703 -2.155007 1.610160

H -1.653353 -2.519737 0.934637

H -1.985135 -2.083132 2.607199

C -4.775433 -2.321064 0.794426

H -5.313106 -2.960369 0.084751

H -5.523147 -1.922228 1.490195

H -3.428663 -4.043432 1.093959

**TS2c***-endo-re*

Imaginary frequency: -231.0553 cm-1

Atom X Y Z

C 0.745415 1.236664 1.809281

C 2.162282 1.034218 1.595851

Rh 0.670987 -0.127681 0.208356

C 1.046025 0.694820 -1.691770

C 2.349210 1.212562 -2.037825

C 3.557500 0.679957 -1.646073

Cl 0.358619 -1.943830 1.908084

H 0.947512 -0.385537 -1.965760

H 0.241265 1.246708 -2.176009

H 2.389171 2.082925 -2.695169

C 4.851697 1.129608 -2.286261

H 5.297995 0.323305 -2.881070

H 5.596407 1.425428 -1.537711

H 4.677347 1.979781 -2.952704

C 2.648512 -0.557854 0.342100

H 2.881976 -1.331697 1.068743

C 3.602444 -0.304916 -0.611432

O 4.787118 -1.079284 -0.578712

C 5.662752 -0.874230 0.452256

O 5.535725 -0.006686 1.284168

C 6.787819 -1.878307 0.387375

H 7.219407 -1.907519 -0.617640

H 6.397459 -2.878768 0.604753

H 7.551301 -1.616914 1.120911

H 0.252984 0.708997 2.623049

H 2.708354 0.421812 2.306650

P -1.729264 -0.112034 -0.117372

C -2.055181 -1.409533 -1.394786

C -2.674374 1.366870 -0.725024

C -2.770143 -0.586786 1.334826

C -1.494887 -2.685883 -1.188845

C -2.753027 -1.158233 -2.585205

C -4.080772 1.350421 -0.756225

C -2.010608 2.529106 -1.142112

C -3.529825 -1.761297 1.386721

C -2.803271 0.291334 2.430068

C -1.654876 -3.687181 -2.147258

H -0.936125 -2.889183 -0.277992

C -2.898180 -2.162516 -3.546095

H -3.185110 -0.180646 -2.769929

C -4.796659 2.458761 -1.206649

H -4.617815 0.468677 -0.419635

C -2.728235 3.642211 -1.589799

H -0.928660 2.571180 -1.107946

C -4.302255 -2.054047 2.513771

H -3.519387 -2.455882 0.553858

C -3.576135 -0.000824 3.551829

H -2.225837 1.211484 2.407723

C -2.353859 -3.428457 -3.329072

H -1.223611 -4.668879 -1.970493

H -3.439886 -1.950785 -4.464301

C -4.121499 3.608482 -1.626152

H -5.882808 2.426871 -1.224229

H -2.194518 4.535030 -1.904663

C -4.327408 -1.177855 3.597908

H -4.882829 -2.972398 2.540307

H -3.588458 0.688655 4.391829

H -2.469709 -4.208189 -4.077229

H -4.680588 4.473595 -1.972438

H -4.926594 -1.409170 4.474437

C 1.598862 3.255811 0.766220

H 1.800615 4.312546 0.968953

C 0.420197 2.708762 1.597732

C 2.786795 2.346316 1.150666

H 3.530657 2.242352 0.359424

H 3.312567 2.748991 2.028832

H 1.386806 3.160471 -0.302628

H -0.558834 2.888944 1.145701

H 0.404290 3.188938 2.589721

**TS2c***-exo-re*

Imaginary frequency: -230.372 cm-1

Atom X Y Z

C 0.782956 0.987229 -1.851620

C 2.178385 1.054217 -1.472358

Rh 0.699132 0.174165 0.079255

C 0.962243 -1.762852 -0.702810

C 2.267556 -2.180874 -1.201725

C 3.481874 -1.861876 -0.649893

Cl 0.549403 1.856239 1.943120

H 0.779725 -2.165363 0.313392

H 0.159170 -2.087008 -1.364900

H 2.280144 -2.850701 -2.063474

C 4.746685 -2.581355 -1.061323

H 5.170901 -3.141927 -0.219306

H 5.518394 -1.883315 -1.406767

H 4.539796 -3.292451 -1.866615

C 2.693871 0.266441 0.405369

H 2.982564 1.101288 1.038781

C 3.579813 -0.769914 0.275345

O 4.760633 -0.732783 1.052853

C 5.737252 0.158694 0.701705

O 5.690952 0.855968 -0.284382

C 6.855958 0.131189 1.714556

H 7.679888 0.753280 1.363280

H 7.198333 -0.895091 1.878866

H 6.490587 0.511082 2.674982

H 0.466253 0.225607 -2.563684

H 2.862109 0.332638 -1.903632

P -1.726322 -0.137557 0.109501

C -2.311613 -0.887250 1.696395

C -2.309314 -1.326681 -1.184125

C -2.878150 1.301026 -0.105985

C -1.395407 -1.134974 2.728149

C -3.666342 -1.200542 1.900115

C -2.340596 -0.908400 -2.527373

C -2.598280 -2.668508 -0.895665

C -4.113485 1.181763 -0.765717

C -2.532044 2.535821 0.468354

C -1.821679 -1.702588 3.932059

H -0.355517 -0.848258 2.608989

C -4.089245 -1.767717 3.100944

H -4.394456 -0.997287 1.120303

C -2.667984 -1.801690 -3.546712

H -2.123413 0.126518 -2.776801

C -2.920973 -3.564412 -1.919206

H -2.576818 -3.019368 0.130780

C -4.978148 2.274030 -0.855505

H -4.405736 0.239635 -1.217308

C -3.402960 3.623403 0.376660

H -1.587643 2.637730 0.994961

C -3.165683 -2.023185 4.118965

H -1.100496 -1.880823 4.724864

H -5.140097 -2.004790 3.244681

C -2.958574 -3.135227 -3.245474

H -2.696902 -1.455559 -4.576554

H -3.144684 -4.599210 -1.673768

C -4.624893 3.498470 -0.286056

H -5.928721 2.164237 -1.371178

H -3.119782 4.570885 0.827342

H -3.497062 -2.461596 5.056584

H -3.212176 -3.831990 -4.039750

H -5.298832 4.348211 -0.357448

C 1.266441 3.298439 -1.285425

H 1.340166 4.301037 -1.718657

C 2.593599 2.517522 -1.381883

H 3.302979 2.737085 -0.581396

H 3.108318 2.741656 -2.329253

C 0.249971 2.405839 -2.030759

H -0.778007 2.544699 -1.684415

H 0.259056 2.629976 -3.110271

H 0.975923 3.397321 -0.236458

**TS3c***-endo-re*

Imaginary frequency: -241.7708 cm-1

Atom X Y Z

C -1.340834 -3.164945 -0.416621

C -2.508187 -1.835240 -1.345245

C -2.272058 -0.528908 -2.021354

C -2.454151 0.706865 -1.432200

C -0.272086 -2.660139 0.454706

Rh -0.507474 -0.872664 -0.589294

Cl 0.727985 -1.780481 -2.563656

H -3.278942 -1.752057 -0.583427

H -2.724643 -2.594091 -2.093282

H 0.739890 -2.981903 0.216337

H -2.027425 -0.565597 -3.079704

C -2.600501 0.775857 0.040170

C -1.744572 0.036590 0.748870

H -1.722739 0.033566 1.836824

C -2.535089 1.970220 -2.248691

H -3.561284 2.361355 -2.235750

H -1.889106 2.756967 -1.842311

H -2.261123 1.788206 -3.292297

O -3.540741 1.677438 0.591212

C -4.859622 1.349268 0.474407

O -5.260355 0.330710 -0.040165

C -5.726731 2.430642 1.075964

H -5.553127 3.381824 0.561719

H -6.776029 2.146913 0.987979

H -5.465745 2.578727 2.128944

H -1.012956 -3.675288 -1.317174

P 1.328681 0.306499 0.135099

C 0.990463 1.475175 1.529210

C 0.927709 2.861275 1.329291

C 0.705921 0.967861 2.809391

C 0.595291 3.717717 2.382334

H 1.141245 3.279162 0.351389

C 0.384684 1.823929 3.861880

H 0.747160 -0.102843 2.987917

C 0.326054 3.203845 3.650362

H 0.552620 4.789407 2.207153

H 0.177266 1.412430 4.846160

H 0.072243 3.871664 4.469110

C 2.778748 -0.687936 0.723954

C 3.180565 -1.791629 -0.049136

C 3.520916 -0.353996 1.868023

C 4.292755 -2.545998 0.326212

H 2.628594 -2.045957 -0.950185

C 4.631394 -1.116147 2.239865

H 3.240838 0.500950 2.473579

C 5.018292 -2.215261 1.472776

H 4.590786 -3.395179 -0.283007

H 5.193437 -0.844530 3.129718

H 5.881884 -2.807414 1.764126

C 2.086061 1.393181 -1.154432

C 3.362733 1.947661 -0.966778

C 1.377782 1.695162 -2.324653

C 3.911897 2.796636 -1.927122

H 3.934042 1.709590 -0.074403

C 1.929111 2.547027 -3.284400

H 0.409044 1.239426 -2.495613

C 3.194809 3.099522 -3.087564

H 4.902593 3.215594 -1.771934

H 1.373445 2.763960 -4.192625

H 3.626403 3.756165 -3.838370

C -2.223303 -3.147183 1.835948

H -2.572959 -3.763263 2.670956

C -2.377226 -3.850090 0.473364

H -3.398662 -3.834987 0.078440

H -2.081327 -4.906414 0.559327

C -0.713105 -2.829649 1.913056

H -0.495445 -1.965156 2.548591

H -0.170274 -3.683303 2.346055

H -2.806865 -2.220415 1.841766

**TS3c***-exo-re*

Imaginary frequency: -240.0786 cm-1

Atom X Y Z

C -1.544980 2.792919 -1.028411

C -2.445608 2.240845 0.653239

C -2.351648 1.095680 1.617705

C -2.660031 -0.216661 1.337489

C -0.199125 2.277865 -1.337234

Rh -0.512883 0.970438 0.248988

Cl 0.635439 2.213010 2.098603

H -3.389432 2.182307 0.114473

H -2.313207 3.173149 1.193556

H -0.116802 1.669280 -2.238708

H -2.109833 1.362714 2.643184

C -2.735886 -0.628723 -0.082716

C -1.764861 -0.176515 -0.880145

H -1.679327 -0.446175 -1.932049

C -2.884491 -1.235848 2.422646

H -3.935510 -1.554423 2.428137

H -2.276940 -2.135200 2.266797

H -2.656967 -0.821667 3.409409

O -3.725836 -1.556036 -0.479809

C -5.019536 -1.123351 -0.479720

O -5.356478 0.000354 -0.185288

C -5.948100 -2.238484 -0.900193

H -5.685903 -2.584337 -1.905577

H -5.841854 -3.093603 -0.224569

H -6.977604 -1.879044 -0.887574

H -2.342493 2.413406 -1.661043

P 1.240757 -0.490153 -0.077154

C 0.899855 -1.670596 -1.458988

C 0.451930 -2.977793 -1.223353

C 0.966077 -1.212402 -2.786861

C 0.095051 -3.810532 -2.287297

H 0.379685 -3.352356 -0.207839

C 0.614102 -2.045845 -3.848135

H 1.305886 -0.201686 -2.994956

C 0.177170 -3.349918 -3.601090

H -0.247610 -4.821493 -2.083677

H 0.680886 -1.675475 -4.867691

H -0.098309 -3.999461 -4.427459

C 2.928009 0.169120 -0.479582

C 3.404496 1.282465 0.233061

C 3.779957 -0.459886 -1.403287

C 4.698936 1.757284 0.014597

H 2.762709 1.769038 0.961716

C 5.072452 0.022874 -1.619140

H 3.443194 -1.327213 -1.960533

C 5.535140 1.133753 -0.912861

H 5.050456 2.619766 0.574561

H 5.716278 -0.474740 -2.339822

H 6.541288 1.508291 -1.082324

C 1.600273 -1.582658 1.371952

C 2.593372 -2.574456 1.297058

C 0.901585 -1.407873 2.573260

C 2.863439 -3.387908 2.395995

H 3.161475 -2.709399 0.381223

C 1.178299 -2.221675 3.675581

H 0.166994 -0.614163 2.652919

C 2.153789 -3.214080 3.588027

H 3.632551 -4.152437 2.324668

H 0.635776 -2.068844 4.604542

H 2.369342 -3.845299 4.446092

C 0.035203 4.548764 -0.479831

H 0.385577 5.551475 -0.746821

C 0.802834 3.431826 -1.218063

H 1.732818 3.154137 -0.717858

H 1.066832 3.760458 -2.235781

C -1.431848 4.311796 -0.886448

H -2.164359 4.762021 -0.208446

H -1.619731 4.737391 -1.884276

H 0.153428 4.420524 0.599573

**TS4c***-endo-re*

Imaginary frequency: -443.3771 cm-1

Atom X Y Z

C 1.139835 2.052502 0.617698

C 2.365204 1.531422 1.420257

Rh 0.738396 -0.177884 0.066902

C 1.096042 1.513821 -1.383414

C 2.517132 1.513124 -1.829838

C 3.391322 0.604582 -1.374685

Cl 0.621804 -2.430556 0.836114

H 0.555067 0.718747 -1.927153

H 0.554001 2.431136 -1.595098

H 2.834234 2.256729 -2.560715

C 4.807142 0.476302 -1.882472

H 4.956663 -0.486003 -2.386571

H 5.534120 0.521636 -1.061741

H 5.036658 1.272244 -2.597865

C 2.646315 0.091028 1.026026

H 2.758375 -0.633508 1.824646

C 2.990421 -0.311687 -0.265495

O 3.425780 -1.627323 -0.496022

C 4.365143 -2.215369 0.309452

O 5.011521 -1.625828 1.142681

C 4.456314 -3.685207 -0.010580

H 4.567430 -3.839471 -1.088168

H 3.520510 -4.166032 0.294934

H 5.297592 -4.125750 0.525975

H 0.187152 1.944304 1.137834

H 2.064228 1.499114 2.474294

P -1.655119 -0.163946 -0.040748

C -2.378363 -1.136416 -1.435583

C -2.375756 1.527085 -0.273534

C -2.517181 -0.771702 1.474031

C -1.669966 -2.251750 -1.913270

C -3.616444 -0.814465 -2.018728

C -2.894774 2.254378 0.809114

C -2.304497 2.154723 -1.530343

C -3.865670 -1.159726 1.456030

C -1.814410 -0.803000 2.687318

C -2.196098 -3.028381 -2.947622

H -0.718191 -2.515756 -1.462447

C -4.135761 -1.593673 -3.053558

H -4.173283 0.051756 -1.674765

C -3.333587 3.570069 0.639559

H -2.968703 1.789694 1.787314

C -2.749187 3.466160 -1.699128

H -1.915132 1.611195 -2.386546

C -4.499904 -1.561828 2.631645

H -4.421600 -1.160179 0.523624

C -2.453879 -1.198065 3.864032

H -0.759505 -0.548384 2.697780

C -3.426451 -2.702395 -3.520181

H -1.638053 -3.889340 -3.305543

H -5.093711 -1.331731 -3.495268

C -3.263805 4.179576 -0.613408

H -3.737647 4.113721 1.489499

H -2.696864 3.929335 -2.681027

C -3.796389 -1.577928 3.838366

H -5.542693 -1.866721 2.603427

H -1.895402 -1.225453 4.795732

H -3.830910 -3.306897 -4.327798

H -3.610439 5.200895 -0.745660

H -4.291263 -1.894746 4.752677

C 2.951985 3.729295 0.389577

H 3.248528 4.716276 0.761921

C 1.423013 3.556264 0.414069

C 3.514188 2.581769 1.264042

H 4.405413 2.133394 0.817416

H 3.808016 2.952263 2.252243

H 3.328187 3.642991 -0.633132

H 0.915969 4.013986 -0.440518

H 1.019525 4.056141 1.307839

**TS4c***-exo-re*

Imaginary frequency: -466.445 cm-1

Atom X Y Z

C 1.296295 -2.474049 0.459198

C 2.666561 -2.297987 -0.268511

Rh 0.858932 -0.201032 -0.095822

C 0.973521 -1.193948 1.970332

C 2.336598 -1.017661 2.541160

C 3.264273 -0.303282 1.884311

Cl 0.953480 1.319018 -1.931966

H 0.445692 -0.230498 2.032643

H 0.337516 -1.909854 2.485889

H 2.562359 -1.447165 3.516698

C 4.617852 0.046431 2.452506

H 4.721082 1.131439 2.576263

H 5.427603 -0.276249 1.786412

H 4.760662 -0.420734 3.431897

C 2.853764 -0.822765 -0.566447

H 3.100047 -0.525974 -1.580150

C 3.010281 0.106315 0.468547

O 3.436864 1.417113 0.199513

C 4.484350 1.657785 -0.653766

O 5.242588 0.807226 -1.053282

C 4.529898 3.124067 -0.996698

H 5.442923 3.343377 -1.551636

H 4.477691 3.735109 -0.090449

H 3.651488 3.362463 -1.606784

H 1.423349 -3.169250 1.286846

H 3.491228 -2.668422 0.353637

P -1.539313 0.203741 0.017960

C -1.976900 1.997226 0.074609

C -2.344374 -0.456815 1.553218

C -2.580911 -0.472790 -1.352819

C -1.004015 2.924605 0.475864

C -3.270995 2.450142 -0.224794

C -2.551807 -1.840036 1.704673

C -2.652644 0.384428 2.633619

C -3.889619 -0.943870 -1.153892

C -2.046533 -0.477517 -2.652893

C -1.326577 4.278169 0.592677

H 0.009247 2.587081 0.670353

C -3.587500 3.804404 -0.115504

H -4.032359 1.748416 -0.551730

C -3.063706 -2.361471 2.892940

H -2.325961 -2.516405 0.886006

C -3.156622 -0.140178 3.826660

H -2.505818 1.455808 2.545262

C -4.644033 -1.415846 -2.230018

H -4.323135 -0.949797 -0.159034

C -2.807746 -0.945742 -3.724949

H -1.043299 -0.096810 -2.820857

C -2.616826 4.720346 0.297133

H -0.562504 4.987213 0.899647

H -4.591594 4.143711 -0.356098

C -3.365757 -1.512546 3.960736

H -3.226950 -3.432280 2.983094

H -3.390674 0.530759 4.648956

C -4.104788 -1.419169 -3.517480

H -5.654080 -1.779163 -2.059046

H -2.381832 -0.939957 -4.724730

H -2.864224 5.775481 0.379491

H -3.762223 -1.918819 4.887269

H -4.692914 -1.787208 -4.354064

C 1.028983 -3.032428 -1.924659

H 0.700228 -3.821676 -2.609848

C 2.514596 -3.147440 -1.549007

H 3.193259 -2.819860 -2.344319

H 2.759819 -4.192546 -1.318148

C 0.305525 -3.099320 -0.563459

H -0.675524 -2.623103 -0.599202

H 0.139286 -4.150505 -0.282840

H 0.835728 -2.066628 -2.404995

**TS5c***-endo-re*

Imaginary frequency: -357.3205 cm-1

Atom X Y Z

C -2.210079 -2.603052 -1.146049

C -3.164864 -1.539352 -1.710883

C -2.515423 -0.201867 -2.027230

C -2.626904 0.896354 -1.154816

C -1.220006 -2.030406 -0.093622

Rh -0.547318 -0.046888 -0.946826

Cl 0.364341 1.114542 -2.848708

H -3.978109 -1.371084 -0.995793

H -3.624018 -1.931730 -2.626563

H -0.183319 -2.186223 -0.428780

H -2.360638 0.036347 -3.076902

C -2.685753 0.607917 0.279090

C -1.717228 -0.248896 0.706917

H -1.503769 -0.388973 1.760444

C -2.788688 2.311746 -1.650244

H -3.857285 2.524826 -1.790359

H -2.382997 3.044725 -0.945113

H -2.282966 2.433711 -2.611131

O -3.437952 1.380925 1.169208

C -4.789307 1.480100 0.953318

O -5.373417 0.890751 0.076070

C -5.409511 2.417083 1.959974

H -4.997250 3.424120 1.834633

H -6.490231 2.439812 1.816642

H -5.170839 2.091634 2.977514

H -1.617898 -2.992763 -1.982511

P 1.573519 0.114132 0.105110

C 1.590697 -0.323333 1.907139

C 1.478854 0.669337 2.893415

C 1.619254 -1.668791 2.312119

C 1.404945 0.326418 4.245278

H 1.458314 1.716111 2.607593

C 1.552566 -2.009881 3.663804

H 1.710468 -2.457285 1.570747

C 1.444115 -1.012864 4.635597

H 1.324532 1.110962 4.993054

H 1.586650 -3.056218 3.955928

H 1.393179 -1.278464 5.688046

C 2.915477 -0.943348 -0.601355

C 2.830069 -1.318964 -1.952375

C 4.022278 -1.368343 0.154188

C 3.831233 -2.102300 -2.530382

H 1.990794 -0.977509 -2.550288

C 5.018993 -2.152773 -0.428261

H 4.103238 -1.097488 1.202254

C 4.924735 -2.522464 -1.771674

H 3.752663 -2.382303 -3.577380

H 5.867581 -2.475332 0.169418

H 5.700469 -3.135128 -2.223520

C 2.253810 1.831056 0.097901

C 3.627929 2.091256 0.203938

C 1.359906 2.909740 0.027861

C 4.096274 3.405102 0.248335

H 4.338668 1.271930 0.239296

C 1.829888 4.222664 0.083291

H 0.299116 2.714843 -0.094403

C 3.198689 4.473106 0.192517

H 5.164263 3.592461 0.322211

H 1.126261 5.048559 0.022677

H 3.565572 5.495633 0.224214

C -2.873348 -3.397008 1.068146

H -3.066681 -4.252096 1.725241

C -2.969827 -3.749765 -0.425481

H -4.002377 -3.845842 -0.779811

H -2.472846 -4.711340 -0.607854

C -1.442245 -2.860584 1.200217

H -1.247341 -2.335135 2.137085

H -0.743321 -3.710134 1.172977

H -3.595382 -2.610614 1.322729

**TS5c***-exo-re*

Imaginary frequency: -338.1508 cm-1

Atom X Y Z

C -2.793699 -2.396985 0.285309

C -2.988673 -2.179884 -1.221625

C -2.553584 -0.829087 -1.758186

C -2.780022 0.439631 -1.181450

C -1.434613 -1.809152 0.714946

Rh -0.617453 -0.272384 -0.826657

Cl 0.220404 0.199828 -3.039442

H -4.046891 -2.337970 -1.482493

H -2.429924 -2.943707 -1.776929

H -1.354271 -1.824888 1.807205

H -2.487647 -0.806085 -2.844705

C -2.741012 0.638975 0.264526

C -1.673702 0.045487 0.877404

H -1.380360 0.293581 1.892438

C -3.087430 1.627337 -2.065698

H -4.168923 1.679767 -2.248256

H -2.768803 2.570771 -1.610610

H -2.578392 1.513182 -3.026300

O -3.486813 1.636977 0.894269

C -4.852784 1.611839 0.755653

O -5.449880 0.753682 0.152019

C -5.466839 2.798839 1.455371

H -5.137479 2.837836 2.498425

H -5.136052 3.725627 0.974028

H -6.553738 2.726103 1.405983

H -3.612873 -1.921062 0.831290

P 1.508444 0.222472 0.098188

C 1.502033 0.434981 1.941769

C 1.487166 1.712343 2.523441

C 1.390218 -0.683408 2.787411

C 1.366272 1.866633 3.906678

H 1.573110 2.593103 1.895651

C 1.276375 -0.528552 4.169542

H 1.409696 -1.685091 2.369056

C 1.261453 0.748762 4.734759

H 1.359907 2.865576 4.334778

H 1.202245 -1.407748 4.804351

H 1.171747 0.869880 5.810869

C 2.852049 -1.011358 -0.204527

C 2.851904 -1.695866 -1.432114

C 3.880139 -1.266875 0.717948

C 3.861011 -2.614694 -1.724744

H 2.069809 -1.492062 -2.158200

C 4.883843 -2.191561 0.421551

H 3.898660 -0.750109 1.672284

C 4.876044 -2.867693 -0.799664

H 3.850442 -3.133919 -2.679349

H 5.671468 -2.380684 1.146383

H 5.657453 -3.587503 -1.028971

C 2.227346 1.820757 -0.486214

C 3.600277 2.092731 -0.385436

C 1.373631 2.804993 -1.004746

C 4.106612 3.328882 -0.788078

H 4.279420 1.337527 -0.001934

C 1.881733 4.043963 -1.399697

H 0.317091 2.586293 -1.119179

C 3.247935 4.307909 -1.293102

H 5.172941 3.524465 -0.711134

H 1.209986 4.794964 -1.806625

H 3.644389 5.268978 -1.610095

C -1.146177 -4.205271 0.715505

H -0.857710 -4.435834 1.747966

C -0.426175 -2.914337 0.247528

H -0.292678 -2.924609 -0.836951

H 0.565091 -2.809274 0.692549

C -2.673377 -3.903159 0.640868

H -3.176319 -4.523153 -0.109753

H -3.157227 -4.116065 1.600089

H -0.856477 -5.067108 0.105507

**11d***-endo-re*

Atom X Y Z

C -0.350543 2.884864 -0.143272

C 0.777859 3.212755 -0.906675

C 2.113623 1.158971 -2.308175

C 1.918602 -0.275113 -2.303573

C 2.271804 -1.102844 -1.235157

Rh 0.502774 0.978790 -0.967989

Cl -1.065864 1.291953 -2.898667

H 2.999010 1.545094 -1.805573

H 1.852009 1.665598 -3.233914

H 1.481618 -0.741665 -3.183191

C 2.632484 -0.490287 0.043113

C 1.905716 0.577071 0.420648

H 2.081163 1.053950 1.382991

C 2.280302 -2.598682 -1.380438

H 3.311155 -2.929857 -1.562060

H 1.924947 -3.095268 -0.471650

H 1.669880 -2.918915 -2.229141

O 3.562752 -1.113931 0.898458

C 4.809619 -1.386194 0.416659

O 5.150028 -1.188579 -0.727277

C 5.678886 -1.964274 1.507523

H 5.810525 -1.227936 2.307647

H 5.198434 -2.842737 1.950022

H 6.650116 -2.235916 1.092662

P -1.221490 -0.464359 0.185683

H -1.304065 2.852804 -0.661346

H 0.620150 3.417940 -1.961563

C -0.560735 -1.381886 1.652019

C -0.423420 -2.778457 1.670195

C -0.079786 -0.651878 2.754257

C 0.160777 -3.424825 2.763290

H -0.779963 -3.369407 0.833567

C 0.488723 -1.298487 3.851016

H -0.153749 0.431681 2.759585

C 0.613004 -2.689718 3.858597

H 0.253355 -4.507832 2.757076

H 0.841087 -0.713718 4.696717

H 1.060845 -3.194219 4.710499

C -2.738219 0.389744 0.820280

C -3.451849 1.182959 -0.097310

C -3.217515 0.269266 2.132867

C -4.616239 1.840474 0.298771

H -3.093400 1.278901 -1.119756

C -4.382227 0.935949 2.524339

H -2.691107 -0.347046 2.854275

C -5.083225 1.722908 1.610667

H -5.158687 2.446907 -0.421771

H -4.741345 0.832303 3.545122

H -5.989469 2.238883 1.916783

C -1.985628 -1.804732 -0.837589

C -3.072760 -2.550331 -0.348301

C -1.488050 -2.092188 -2.114877

C -3.631075 -3.574309 -1.111916

H -3.486920 -2.327955 0.631049

C -2.050899 -3.117223 -2.880395

H -0.691286 -1.484516 -2.525561

C -3.118232 -3.862076 -2.380152

H -4.470687 -4.142423 -0.720086

H -1.662468 -3.319107 -3.875043

H -3.558204 -4.655899 -2.978093

C -0.445977 3.207308 1.337239

H -1.174150 4.031057 1.411921

H -0.898729 2.382336 1.897887

C 2.019026 3.819550 -0.267546

H 2.768570 3.047162 -0.052266

H 2.488131 4.506548 -0.982221

C 0.872495 3.666191 1.985664

C 1.676662 4.563649 1.036378

H 2.597479 4.908844 1.522805

H 1.087430 5.461339 0.800234

H 1.485226 2.800962 2.267614

H 0.648829 4.199848 2.918044

**11d***-exo-re*

Atom X Y Z

C -0.850619 2.269615 1.384746

C 0.055741 3.219286 0.887215

C 1.650855 2.493481 -1.354179

C 1.928401 1.195600 -1.946576

C 2.622224 0.183043 -1.284216

Rh 0.233809 1.349796 -0.342997

Cl -1.370713 1.872851 -2.198562

H 2.409337 2.894951 -0.682790

H 1.203018 3.223591 -2.023734

H 1.626853 1.019631 -2.976134

C 2.771901 0.273163 0.164366

C 1.740115 0.798467 0.856069

H 1.755451 0.827903 1.947382

C 3.182676 -0.994679 -2.028280

H 4.250441 -0.818302 -2.215210

H 3.094850 -1.923159 -1.454470

H 2.687097 -1.124602 -2.993473

O 3.863771 -0.349376 0.803032

C 5.124318 0.048314 0.457931

O 5.362249 0.860671 -0.405286

C 6.150420 -0.673428 1.298065

H 5.989606 -0.451249 2.358149

H 6.047184 -1.756379 1.172873

H 7.150644 -0.357917 0.999624

P -0.895451 -0.928278 0.098593

C -0.248218 -1.620502 1.682550

C 0.934491 -2.375994 1.732151

C -0.838543 -1.228584 2.898211

C 1.493512 -2.750789 2.955812

H 1.424162 -2.677419 0.812260

C -0.278363 -1.602643 4.120140

H -1.751152 -0.639626 2.891260

C 0.889653 -2.367805 4.153662

H 2.406014 -3.340795 2.968369

H -0.757930 -1.296671 5.046223

H 1.325009 -2.660573 5.105199

C -2.732982 -1.160303 0.237414

C -3.565937 -0.383743 -0.583717

C -3.309976 -2.151357 1.049281

C -4.948051 -0.586451 -0.576988

H -3.129751 0.370259 -1.233148

C -4.691698 -2.345157 1.055623

H -2.685152 -2.774373 1.681091

C -5.515076 -1.561508 0.244064

H -5.579201 0.023501 -1.217865

H -5.123108 -3.113296 1.692455

H -6.591133 -1.714744 0.249681

C -0.515893 -2.200583 -1.195956

C -0.491964 -3.580220 -0.934068

C -0.358592 -1.757349 -2.518921

C -0.282347 -4.493769 -1.967641

H -0.639316 -3.947101 0.077048

C -0.163591 -2.676520 -3.553141

H -0.416770 -0.694677 -2.739471

C -0.115573 -4.044085 -3.279736

H -0.259050 -5.558108 -1.748158

H -0.053981 -2.319141 -4.573762

H 0.041218 -4.757594 -4.084430

H 1.047115 3.259719 1.332748

H -0.514002 1.633241 2.201253

C -0.400517 4.531926 0.274085

H -0.097373 5.310813 0.993120

H 0.147539 4.752105 -0.648732

C -1.916643 4.633358 0.035946

H -2.174904 4.129722 -0.901246

H -2.191238 5.690439 -0.071634

C -2.355425 2.491257 1.288779

H -2.767306 1.979102 0.415780

H -2.836004 2.045590 2.169330

C -2.698005 3.988035 1.186336

H -3.777425 4.107694 1.031680

H -2.454218 4.491545 2.134107

**12d***-endo-re*

Atom X Y Z

C 2.876291 -1.531389 0.761442

C 3.525997 -0.170478 1.064959

Rh 1.005190 -0.859034 0.053677

C 1.565888 -0.936702 -1.912310

C 2.189410 0.339914 -2.359511

C 2.261420 1.420624 -1.571609

Cl 0.296950 -3.121369 0.021792

H 2.231855 -1.795133 -2.044914

H 0.643881 -1.148389 -2.467765

C 2.829705 2.748399 -2.014513

H 3.631167 3.084526 -1.346951

H 2.062227 3.532494 -2.010891

H 3.226298 2.675536 -3.031852

C 2.284860 0.712606 0.941035

H 1.813703 1.000044 1.881535

C 1.728796 1.313147 -0.180680

O 0.740233 2.308119 0.081106

C 1.129002 3.461891 0.714356

O 2.248147 3.669428 1.117953

C -0.039500 4.410582 0.833914

H -0.620787 4.434792 -0.091340

H 0.331464 5.404702 1.087913

H -0.710650 4.069614 1.630563

P -1.523549 -0.214131 -0.068135

H 2.473542 -1.958393 1.690319

H 3.850022 -0.134173 2.115734

C -2.236755 1.402630 -0.633880

C -1.732102 1.947402 -1.824793

C -3.297919 2.057709 0.009400

C -2.280125 3.112127 -2.364923

H -0.898206 1.464224 -2.326480

C -3.838775 3.229390 -0.524095

H -3.703976 1.657464 0.933330

C -3.334052 3.758068 -1.714111

H -1.880609 3.516791 -3.291255

H -4.659884 3.724697 -0.012167

H -3.759848 4.666528 -2.131900

C -1.853761 -0.275904 1.747452

C -2.191225 -1.492054 2.366429

C -1.595375 0.842836 2.559789

C -2.295360 -1.575023 3.755840

H -2.358157 -2.377299 1.761995

C -1.702732 0.756591 3.948756

H -1.313332 1.786085 2.102270

C -2.055946 -0.452750 4.551261

H -2.559525 -2.523444 4.215726

H -1.510155 1.635220 4.559139

H -2.138847 -0.520601 5.632583

C -2.763413 -1.376755 -0.802932

C -2.379137 -2.190940 -1.876770

C -4.100332 -1.406831 -0.373313

C -3.311752 -3.014287 -2.511714

H -1.344241 -2.203991 -2.198279

C -5.028411 -2.236345 -1.001287

H -4.420162 -0.784614 0.457381

C -4.635870 -3.040480 -2.074741

H -2.996205 -3.644251 -3.338937

H -6.058377 -2.252705 -0.654438

H -5.359875 -3.686406 -2.564467

C 3.733382 -2.600148 0.071526

H 4.183706 -3.216900 0.866757

H 3.093113 -3.282179 -0.498739

C 4.741733 0.230986 0.205983

H 4.416346 0.614028 -0.764165

H 5.276636 1.047435 0.706815

C 4.886900 -2.062904 -0.790976

H 5.550165 -2.894425 -1.061769

H 4.506804 -1.647752 -1.735017

C 5.664501 -0.966209 -0.052304

H 6.533080 -0.643296 -0.640221

H 6.055735 -1.363300 0.896781

H 2.605518 0.380227 -3.369772

**12d***-exo-re*

Atom X Y Z

C 2.949782 -1.643644 -0.886335

C 3.704813 -0.311640 -0.781436

Rh 0.988294 -0.863180 -0.597655

C 0.870265 -0.525849 -2.617496

C 1.327873 0.850029 -2.974483

C 1.662642 1.762524 -2.052116

Cl 0.172840 -3.088700 -0.756328

H 1.441965 -1.299697 -3.142441

H -0.186805 -0.673353 -2.870153

H 1.376856 1.110177 -4.035200

C 2.045309 3.188092 -2.370019

H 3.031183 3.440071 -1.963500

H 1.329611 3.896087 -1.932626

H 2.056924 3.351171 -3.452048

C 2.639557 0.490824 -0.055313

H 2.690347 0.504435 1.032065

C 1.693610 1.327251 -0.626885

O 0.989211 2.187954 0.259884

C 1.700224 3.149347 0.935751

O 2.895725 3.295255 0.845696

C 0.771483 3.974172 1.791835

H -0.064369 4.350513 1.195199

H 1.329618 4.799058 2.236417

H 0.342592 3.351163 2.584118

P -1.465678 -0.166452 0.035189

C -2.054246 1.585428 0.202403

C -1.945531 2.414864 -0.926100

C -2.641395 2.105645 1.364516

C -2.420233 3.725600 -0.896663

H -1.477532 2.035580 -1.830841

C -3.112065 3.422016 1.396203

H -2.736653 1.484689 2.249724

C -3.005281 4.234759 0.266943

H -2.332045 4.350846 -1.781407

H -3.568085 3.807384 2.304699

H -3.376378 5.255982 0.290690

C -1.507493 -0.812577 1.762880

C -2.303992 -1.898297 2.150744

C -0.607104 -0.265377 2.696381

C -2.211988 -2.416000 3.444888

H -2.989846 -2.347167 1.440360

C -0.523655 -0.778892 3.990661

H 0.023695 0.572590 2.408820

C -1.326095 -1.858824 4.367710

H -2.834126 -3.260591 3.728652

H 0.170913 -0.339465 4.702045

H -1.256854 -2.264990 5.373149

C -2.950121 -0.908723 -0.795821

C -2.791238 -1.822900 -1.847811

C -4.251869 -0.534837 -0.415732

C -3.907835 -2.349615 -2.502937

H -1.798418 -2.152324 -2.128794

C -5.363619 -1.067921 -1.066183

H -4.400081 0.182910 0.385225

C -5.193807 -1.975381 -2.115305

H -3.765499 -3.058546 -3.314144

H -6.362144 -0.769482 -0.757444

H -6.060742 -2.386872 -2.625688

H 3.868539 0.123979 -1.772609

H 2.951290 -2.073366 -1.890593

C 5.041608 -0.400957 -0.027092

H 5.712605 -1.018659 -0.638176

H 5.506803 0.590959 0.036567

C 3.405858 -2.685445 0.140929

H 2.640058 -3.455924 0.263234

H 4.293487 -3.195917 -0.270711

C 4.913218 -1.018922 1.386809

H 5.875262 -1.473970 1.651986

H 4.749450 -0.225248 2.126885

C 3.782196 -2.076894 1.499213

H 2.884305 -1.624917 1.943745

H 4.083029 -2.872165 2.191524

**13d***-endo-re*

Atom X Y Z

C -0.594240 -2.232027 -1.196592

C -1.990354 -2.857989 -1.026166

C -3.121917 -1.891242 -1.468277

C -2.676799 -0.494303 -1.849585

C -2.822719 0.637366 -1.057853

Rh -0.608221 -0.152717 -0.834439

Cl 0.248079 0.471359 -3.085543

H -3.858124 -1.806218 -0.662836

H -3.653056 -2.306183 -2.333530

H -2.521700 -0.309282 -2.910715

C -2.807084 0.500328 0.420206

C -1.681591 -0.091574 0.862785

H -1.432437 -0.225836 1.914732

C -2.973609 2.007985 -1.676187

H -4.043579 2.235855 -1.777230

H -2.515830 2.792597 -1.065204

H -2.521042 2.030975 -2.671272

O -3.740465 1.154155 1.241268

C -5.070306 1.047887 0.943908

O -5.507176 0.408915 0.015161

C -5.891865 1.836276 1.935298

H -5.704815 1.473232 2.951254

H -5.603566 2.892434 1.908337

H -6.949845 1.733633 1.691842

P 1.473440 0.306046 0.184805

H -0.373392 -2.127401 -2.263637

H -1.988213 -3.730566 -1.704153

C 1.645038 -0.010315 2.006019

C 1.211741 0.921174 2.962392

C 2.155313 -1.240293 2.456143

C 1.284364 0.630111 4.326596

H 0.829741 1.886833 2.650535

C 2.230994 -1.527859 3.819034

H 2.512500 -1.974149 1.741592

C 1.792490 -0.594525 4.760160

H 0.948433 1.369172 5.049075

H 2.634988 -2.483203 4.143044

H 1.851477 -0.818411 5.821786

C 3.081418 -0.304161 -0.486379

C 3.145243 -0.834459 -1.784065

C 4.264449 -0.203578 0.269154

C 4.366626 -1.266284 -2.307172

H 2.247519 -0.874854 -2.392093

C 5.480967 -0.633552 -0.260332

H 4.238375 0.208666 1.272867

C 5.533585 -1.170758 -1.549016

H 4.400300 -1.672827 -3.314175

H 6.386085 -0.549214 0.335262

H 6.481580 -1.508462 -1.959523

C 1.578599 2.150324 0.036651

C 2.732632 2.799754 -0.423871

C 0.448126 2.925836 0.350991

C 2.758740 4.189721 -0.553512

H 3.611465 2.225001 -0.693000

C 0.480783 4.315283 0.229344

H -0.465026 2.443768 0.689737

C 1.637039 4.951563 -0.225425

H 3.660402 4.675320 -0.916841

H -0.401529 4.897614 0.481446

H 1.660727 6.032876 -0.329533

C 0.496194 -3.079927 -0.540076

H 0.618198 -3.971468 -1.182913

H 1.463911 -2.572130 -0.576225

C -2.278803 -3.410323 0.382338

H -2.412497 -2.568163 1.073377

H -3.235335 -3.950251 0.356634

C 0.172601 -3.567103 0.878193

C -1.162200 -4.317972 0.905840

H -1.394611 -4.656165 1.924012

H -1.090625 -5.222429 0.282832

H 0.112961 -2.709964 1.559138

H 0.985974 -4.209061 1.242550

**13d***-exo-re*

Atom X Y Z

C 0.773826 1.941801 0.311494

C 2.240822 2.185611 0.694086

C 3.169906 1.927902 -0.513142

C 2.758574 0.791897 -1.434573

C 2.836051 -0.567092 -1.182275

Rh 0.594215 0.135177 -0.728863

Cl -0.150158 0.333885 -3.079939

H 4.184599 1.723361 -0.148333

H 3.229682 2.830016 -1.131287

H 2.682812 1.054489 -2.488410

C 2.740907 -1.095718 0.202775

C 1.572551 -0.782690 0.775080

H 1.247369 -1.100987 1.763674

C 3.002917 -1.557795 -2.312375

H 4.066113 -1.822284 -2.398947

H 2.444801 -2.483301 -2.132850

H 2.678731 -1.126574 -3.263000

O 3.667796 -2.021016 0.713283

C 4.997357 -1.725202 0.633617

O 5.438446 -0.699453 0.167932

C 5.819354 -2.856402 1.204322

H 5.536119 -3.038575 2.246224

H 5.626080 -3.779935 0.648409

H 6.878003 -2.601626 1.144936

P -1.526668 -0.336395 0.135462

C -1.526100 -0.562362 1.970887

C -1.380990 -1.839301 2.535786

C -1.547463 0.551472 2.828960

C -1.266365 -1.998069 3.918725

H -1.359964 -2.715663 1.896180

C -1.437075 0.390338 4.210681

H -1.671549 1.550227 2.422253

C -1.294338 -0.885404 4.760146

H -1.157337 -2.995595 4.335780

H -1.464674 1.263921 4.856410

H -1.207858 -1.010402 5.836009

C -2.951645 0.800413 -0.183385

C -3.046364 1.419013 -1.441855

C -3.979091 1.002224 0.754108

C -4.142629 2.228110 -1.745609

H -2.268150 1.256932 -2.182478

C -5.070546 1.815637 0.443686

H -3.933385 0.530068 1.729548

C -5.154010 2.432457 -0.805530

H -4.200822 2.699531 -2.722876

H -5.854992 1.964363 1.181150

H -6.003709 3.066477 -1.044790

C -2.138510 -1.960023 -0.499157

C -3.426354 -2.419350 -0.177414

C -1.314668 -2.755193 -1.307258

C -3.872970 -3.654088 -0.644783

H -4.084876 -1.809167 0.433484

C -1.764613 -3.992159 -1.775777

H -0.329978 -2.399475 -1.592418

C -3.041500 -4.443945 -1.444011

H -4.872100 -3.997170 -0.389900

H -1.117785 -4.594004 -2.407972

H -3.392826 -5.404341 -1.811654

H 2.508717 1.466157 1.471933

H 0.184506 1.852582 1.230145

C 2.432127 3.603640 1.292807

H 1.954528 3.626593 2.283108

H 3.502742 3.783230 1.462981

C 1.826774 4.720978 0.430973

H 2.387441 4.821602 -0.508808

H 1.923453 5.683887 0.949493

C 0.195194 3.049406 -0.572115

H 0.701305 3.068568 -1.544712

H -0.862402 2.864436 -0.779453

C 0.355888 4.428593 0.109814

H -0.061800 5.208539 -0.539986

H -0.231793 4.446596 1.040191

**14d**

Atom X Y Z

C 3.075238 0.546048 0.914158

C 3.675522 1.377423 -0.248530

C 3.871029 0.575040 -1.543161

C 2.684222 -0.151054 -2.168943

C 2.171116 -1.394396 -1.736547

Rh 0.717674 0.064445 -1.104924

Cl -0.189209 1.304858 -2.936455

H 4.660065 -0.171134 -1.364825

H 4.270009 1.260081 -2.300418

H 2.576961 0.022706 -3.238005

C 1.884237 -1.524730 -0.329775

C 1.944852 -0.434167 0.587217

H 1.405859 -0.691669 1.499721

C 1.785368 -2.489309 -2.701672

H 2.614803 -3.205063 -2.791928

H 0.900362 -3.040693 -2.375445

H 1.599992 -2.066479 -3.693035

O 1.297103 -2.734036 0.084251

C 2.025225 -3.494584 0.966338

O 3.126796 -3.193654 1.356651

C 1.248108 -4.727830 1.352173

H 0.956753 -5.288125 0.457930

H 1.861821 -5.352640 2.002048

H 0.328922 -4.435390 1.870695

P -1.349176 0.187641 0.073902

C -1.493975 -0.924514 1.549774

C -2.180497 -2.145825 1.471914

C -0.843175 -0.598742 2.753479

C -2.220860 -3.012715 2.567026

H -2.694434 -2.420394 0.556758

C -0.884895 -1.464575 3.847144

H -0.315604 0.345861 2.846587

C -1.574263 -2.676276 3.757737

H -2.769456 -3.947943 2.489213

H -0.381958 -1.188724 4.770171

H -1.612159 -3.348105 4.611034

C -1.812611 1.853337 0.739075

C -1.425088 2.989227 0.007749

C -2.561330 2.026958 1.915423

C -1.775159 4.266361 0.449446

H -0.868120 2.868796 -0.916613

C -2.904774 3.307107 2.354948

H -2.873799 1.165233 2.496520

C -2.510793 4.429650 1.624601

H -1.470144 5.133848 -0.129630

H -3.481574 3.424223 3.268745

H -2.777678 5.425329 1.968982

C -2.807699 -0.308562 -0.947020

C -4.113667 0.081327 -0.612861

C -2.607231 -1.131909 -2.064332

C -5.197050 -0.349956 -1.378757

H -4.287846 0.730431 0.239695

C -3.693655 -1.568426 -2.824862

H -1.596889 -1.408086 -2.348122

C -4.989442 -1.177856 -2.484460

H -6.202812 -0.034798 -1.113608

H -3.523247 -2.199734 -3.692740

H -5.834099 -1.509739 -3.082484

H 4.691891 1.656376 0.067083

H 3.885811 -0.076937 1.325205

C 2.903028 2.694882 -0.435673

H 3.243619 3.205108 -1.345036

H 1.841873 2.466418 -0.599108

C 2.572651 1.485795 2.049760

H 1.509446 1.707654 1.882467

H 2.637365 0.963581 3.012835

C 3.080984 3.619881 0.794396

H 2.184005 4.240573 0.913727

H 3.915900 4.312282 0.627697

C 3.331664 2.815641 2.091664

H 3.024341 3.401621 2.966313

H 4.405211 2.620317 2.218559

**TS2d***-endo-re*

Imaginary frequency: -329.0106 cm-1

Atom X Y Z

C -2.141251 -2.294681 -1.018988

C -2.833948 -1.026910 -1.174223

Rh -0.478760 -1.219358 -0.242018

C -0.936690 -1.265733 1.764596

C -2.100441 -0.480856 2.219091

C -2.578198 0.707985 1.744208

Cl 0.763858 -3.318994 -0.202581

H -1.034548 -2.319972 2.046039

H -0.013079 -0.884813 2.214458

C -3.598427 1.501388 2.535029

H -4.504368 1.704284 1.950904

H -3.189982 2.473698 2.834108

H -3.886894 0.964041 3.443131

C -1.683401 0.429270 -0.584635

H -1.456346 0.988615 -1.491262

C -2.208140 1.188790 0.434722

O -2.492287 2.554137 0.256574

C -3.156893 3.012840 -0.849694

O -3.653879 2.306397 -1.694245

C -3.174815 4.521860 -0.840636

H -3.608198 4.892009 0.094113

H -3.753783 4.883788 -1.690907

H -2.147877 4.898254 -0.897262

P 1.611915 0.081974 -0.005085

H -1.573104 -2.602660 -1.904799

H -2.785382 -0.614118 -2.181497

C 1.412281 1.918723 -0.102910

C 0.809713 2.597718 0.971122

C 1.755629 2.652178 -1.249228

C 0.584931 3.972773 0.911188

H 0.527010 2.052035 1.867107

C 1.520147 4.028000 -1.312784

H 2.221532 2.152499 -2.092276

C 0.941356 4.694094 -0.231331

H 0.129157 4.479679 1.757497

H 1.802568 4.579227 -2.206001

H 0.773901 5.767283 -0.275298

C 2.779796 -0.281708 -1.390037

C 4.146361 0.031265 -1.320244

C 2.279436 -0.867389 -2.561839

C 4.987759 -0.228116 -2.402336

H 4.556569 0.470706 -0.415918

C 3.119917 -1.121484 -3.647283

H 1.232070 -1.150517 -2.619698

C 4.475885 -0.802050 -3.568778

H 6.044899 0.014322 -2.332428

H 2.716703 -1.582738 -4.544603

H 5.133736 -1.007044 -4.409167

C 2.649291 -0.146640 1.511840

C 2.740817 -1.438708 2.059662

C 3.364743 0.899853 2.118657

C 3.530883 -1.671089 3.187205

H 2.206719 -2.261086 1.591464

C 4.150504 0.660738 3.247634

H 3.310729 1.905112 1.714119

C 4.234349 -0.624715 3.785932

H 3.592836 -2.676037 3.596241

H 4.698370 1.481398 3.703734

H 4.845857 -0.808845 4.665469

C -2.783045 -3.495016 -0.329208

H -2.956890 -4.252077 -1.108840

H -2.070933 -3.955989 0.364765

C -4.215697 -0.872894 -0.542712

H -4.133131 -0.526056 0.489052

H -4.766362 -0.105543 -1.096034

C -4.125559 -3.197515 0.356099

H -4.665927 -4.137604 0.522175

H -3.959206 -2.751790 1.347178

C -4.955917 -2.219633 -0.483557

H -5.947908 -2.066414 -0.040273

H -5.117146 -2.627561 -1.491891

H -2.620649 -0.889958 3.089558

**TS2d***-exo-re*

Imaginary frequency: -326.8008 cm-1

Atom X Y Z

C -1.872594 -2.746804 0.622640

C -2.630391 -1.831740 -0.221504

Rh -0.375966 -1.249251 0.498586

C -0.763936 -0.594751 2.435066

C -2.160635 -0.260047 2.717590

C -2.969480 0.544500 1.958080

Cl 1.339588 -2.798433 1.292058

H -0.366945 -1.356656 3.108109

H -0.119851 0.288854 2.457492

H -2.580853 -0.644653 3.649344

C -4.265925 1.102192 2.504676

H -5.120598 0.857946 1.862501

H -4.220179 2.195654 2.578165

H -4.463282 0.708586 3.506051

C -1.885737 -0.033980 -0.218476

H -1.791792 0.296069 -1.252937

C -2.634138 0.797952 0.585259

O -3.248403 1.938898 0.021158

C -4.241142 1.757573 -0.899608

O -4.709521 0.678717 -1.182300

C -4.654120 3.083066 -1.490545

H -4.926201 3.785892 -0.696671

H -5.497404 2.935017 -2.165844

H -3.809370 3.516921 -2.036606

P 1.429334 0.419405 -0.062219

C 0.808548 1.860332 -1.041356

C -0.009948 2.820490 -0.420014

C 1.017845 1.959156 -2.426740

C -0.573106 3.864321 -1.155059

H -0.201461 2.760535 0.647045

C 0.443680 2.997535 -3.163625

H 1.641736 1.230024 -2.933389

C -0.347884 3.957324 -2.530401

H -1.190612 4.601917 -0.649662

H 0.625894 3.059543 -4.233371

H -0.782812 4.772341 -3.103132

C 2.824317 -0.208032 -1.102825

C 3.958774 0.582267 -1.354649

C 2.751610 -1.488351 -1.668082

C 4.988240 0.106046 -2.164911

H 4.041094 1.570512 -0.911578

C 3.783402 -1.963579 -2.482215

H 1.907574 -2.129211 -1.439985

C 4.900703 -1.168149 -2.733451

H 5.861076 0.727144 -2.348530

H 3.716368 -2.962182 -2.905330

H 5.705871 -1.540304 -3.361527

C 2.317297 1.194829 1.366619

C 2.685119 0.352538 2.431225

C 2.672354 2.552493 1.421568

C 3.391233 0.862675 3.521788

H 2.427153 -0.702850 2.395260

C 3.374167 3.057674 2.518278

H 2.402765 3.222022 0.611359

C 3.734283 2.215155 3.571238

H 3.670273 0.197758 4.334864

H 3.641154 4.111098 2.545334

H 4.280005 2.610480 4.424036

H -3.574697 -1.485817 0.183495

H -2.244432 -2.844099 1.642520

C -2.668035 -2.188220 -1.701849

H -3.431297 -2.973286 -1.821555

H -3.005104 -1.338463 -2.302462

C -1.454353 -4.085930 0.024167

H -0.717831 -4.558274 0.678121

H -2.350961 -4.728238 0.042927

C -1.304771 -2.717689 -2.156667

H -1.283420 -2.860447 -3.243746

H -0.549792 -1.935687 -1.948347

C -0.914986 -4.019964 -1.426897

H 0.175456 -4.117288 -1.416633

H -1.301003 -4.878494 -1.990153

**TS3d***-endo-re*

Imaginary frequency: -197.757 cm-1

Atom X Y Z

C 0.423961 2.538805 -0.449191

C 1.765232 2.612390 -1.099587

C 2.510865 1.096227 -1.991292

C 2.138705 -0.352545 -2.187766

C 2.340495 -1.358744 -1.270000

Rh 0.485662 0.515720 -0.861476

Cl -0.743561 0.940919 -2.993075

H 3.462008 1.133374 -1.465280

H 2.558812 1.554311 -2.975719

H 1.811635 -0.622541 -3.188590

C 2.548810 -0.988127 0.147189

C 1.738392 -0.048888 0.643178

H 1.772870 0.259757 1.685920

C 2.333379 -2.813494 -1.662724

H 3.341567 -3.236519 -1.558729

H 1.673029 -3.404937 -1.017585

H 2.024075 -2.943519 -2.704285

O 3.495954 -1.714545 0.907675

C 4.812160 -1.445980 0.672962

O 5.203980 -0.616776 -0.116171

C 5.691102 -2.319914 1.536970

H 5.472235 -2.140654 2.594866

H 5.485414 -3.376771 1.337696

H 6.739053 -2.098830 1.331597

P -1.390661 -0.269558 0.180460

H -0.365280 2.859492 -1.128401

H 1.694101 3.189200 -2.021421

C -1.081328 -1.093453 1.809549

C -1.205208 -2.481117 1.968402

C -0.643205 -0.329409 2.905941

C -0.904268 -3.087295 3.191160

H -1.540234 -3.094908 1.139395

C -0.355774 -0.933618 4.128898

H -0.530203 0.745977 2.806451

C -0.483519 -2.317398 4.274732

H -1.005776 -4.164447 3.293417

H -0.027740 -0.323485 4.966327

H -0.255267 -2.789660 5.226381

C -2.727931 0.960595 0.539121

C -3.095617 1.850043 -0.486384

C -3.414725 1.009456 1.762358

C -4.122247 2.771789 -0.281384

H -2.582586 1.809377 -1.443759

C -4.438854 1.939224 1.961873

H -3.158437 0.324816 2.563658

C -4.793174 2.823441 0.942951

H -4.396701 3.451503 -1.083704

H -4.959461 1.966330 2.915640

H -5.589817 3.545922 1.100059

C -2.283618 -1.569473 -0.783107

C -3.612286 -1.904793 -0.476604

C -1.624685 -2.261912 -1.807453

C -4.263329 -2.920683 -1.176260

H -4.142924 -1.368314 0.304236

C -2.277465 -3.280750 -2.504208

H -0.611904 -1.983218 -2.076021

C -3.595954 -3.612082 -2.190264

H -5.293311 -3.167374 -0.932734

H -1.758567 -3.803143 -3.303286

H -4.105624 -4.399935 -2.738619

C 0.206955 3.161468 0.935921

H -0.305912 4.120782 0.761861

H -0.503746 2.566103 1.518923

C 2.945331 3.041894 -0.217430

H 3.350570 2.164844 0.302236

H 3.750079 3.423172 -0.858303

C 1.472538 3.459087 1.751429

C 2.552100 4.068456 0.852281

H 3.438414 4.343921 1.437418

H 2.177048 4.992650 0.388570

H 1.869001 2.541939 2.207060

H 1.218735 4.136468 2.576908

**TS3d***-exo-re*

Imaginary frequency: -258.0452 cm-1

Atom X Y Z

C 0.303874 2.264251 1.084930

C 1.691864 2.630292 0.748091

C 2.494154 1.925555 -0.927305

C 2.361224 0.691116 -1.770676

C 2.629942 -0.596449 -1.366323

Rh 0.534315 0.780265 -0.370821

Cl -0.539435 1.935733 -2.334337

H 3.453301 1.911685 -0.411961

H 2.347051 2.802266 -1.550924

H 2.120211 0.864272 -2.816427

C 2.691890 -0.879730 0.085908

C 1.739119 -0.326326 0.840333

H 1.648833 -0.500824 1.911822

C 2.812920 -1.721956 -2.349671

H 3.846157 -2.091846 -2.310777

H 2.159410 -2.571983 -2.119231

H 2.615245 -1.390773 -3.373517

O 3.653933 -1.797640 0.565854

C 4.956484 -1.391691 0.555968

O 5.321662 -0.299883 0.185181

C 5.854895 -2.490077 1.074516

H 5.575213 -2.747897 2.101445

H 5.735399 -3.394194 0.468587

H 6.892460 -2.155623 1.045268

P -1.283453 -0.558391 0.099792

C -0.999717 -1.584457 1.611591

C -0.631390 -2.934789 1.536116

C -1.025246 -0.964559 2.873843

C -0.314192 -3.651570 2.693113

H -0.588997 -3.433125 0.573615

C -0.713438 -1.682224 4.028102

H -1.298530 0.083743 2.957409

C -0.357009 -3.030659 3.940978

H -0.032347 -4.698083 2.613568

H -0.747688 -1.187101 4.994943

H -0.112049 -3.590052 4.839732

C -2.950575 0.200122 0.394475

C -3.394741 1.204011 -0.482544

C -3.819432 -0.256516 1.399520

C -4.674980 1.742054 -0.345305

H -2.737825 1.560310 -1.270392

C -5.097814 0.289566 1.533369

H -3.507639 -1.038867 2.082714

C -5.528807 1.291286 0.663025

H -5.001885 2.518406 -1.031921

H -5.755674 -0.074083 2.318573

H -6.524203 1.714951 0.768217

C -1.670351 -1.788857 -1.226149

C -2.689902 -2.740864 -1.053868

C -0.969000 -1.753604 -2.438085

C -2.980814 -3.654585 -2.065215

H -3.261402 -2.766533 -0.130445

C -1.267254 -2.667081 -3.453211

H -0.214331 -0.990589 -2.596050

C -2.267217 -3.620847 -3.267153

H -3.769501 -4.387829 -1.918718

H -0.721918 -2.622628 -4.391987

H -2.499087 -4.330301 -4.057139

H 2.399845 2.115213 1.393085

H 0.241377 1.735914 2.038363

C 2.036046 4.115474 0.599444

H 2.326906 4.452130 1.607055

H 2.924379 4.254264 -0.028153

C 0.865975 4.982452 0.124080

H 0.610457 4.741435 -0.916200

H 1.165436 6.037871 0.149429

C -0.825690 3.279381 0.880172

H -1.248305 3.165181 -0.120499

H -1.632197 3.065558 1.591561

C -0.355058 4.739193 1.018770

H -1.177324 5.412283 0.746134

H -0.092071 4.966039 2.063304

**TS4d***-endo-re*

Imaginary frequency: -347.0053 cm-1

Atom X Y Z

C 2.726625 -1.742776 0.553725

C 2.955724 -0.420423 1.309181

Rh 0.758574 -0.903350 -0.323772

C 2.526405 -1.344569 -1.651208

C 3.187405 -0.058028 -1.964573

C 2.741838 1.107462 -1.471060

Cl -0.018456 -3.118797 -0.874245

H 3.166996 -2.215433 -1.742892

H 1.669136 -1.530769 -2.310518

H 4.055070 -0.070139 -2.626221

C 3.321563 2.441307 -1.877494

H 3.729385 2.983328 -1.016699

H 2.557524 3.085632 -2.330100

H 4.117341 2.301772 -2.615617

C 1.813819 0.493401 0.875777

H 1.241428 0.967672 1.666448

C 1.687912 1.072936 -0.409285

O 0.790444 2.185464 -0.509033

C 1.077461 3.326957 0.188765

O 2.023984 3.453797 0.930423

C 0.042562 4.392082 -0.086358

H -0.346789 4.318411 -1.104380

H 0.485025 5.374435 0.089587

H -0.794043 4.256260 0.607538

P -1.472095 -0.200855 0.073782

H 1.918577 -2.319607 1.010999

H 2.783860 -0.658237 2.371486

C -2.251783 0.652069 -1.370649

C -1.459761 1.035501 -2.462018

C -3.636864 0.890295 -1.421113

C -2.034896 1.660088 -3.571407

H -0.394112 0.835284 -2.441938

C -4.208325 1.520168 -2.526642

H -4.272680 0.568717 -0.601446

C -3.407462 1.907878 -3.604163

H -1.408258 1.944207 -4.412672

H -5.280244 1.697738 -2.550899

H -3.854912 2.390772 -4.468929

C -1.683861 0.955885 1.506421

C -1.068714 0.609594 2.722752

C -2.444480 2.132630 1.453867

C -1.218283 1.410931 3.855656

H -0.475732 -0.299664 2.783478

C -2.588067 2.938691 2.586982

H -2.930321 2.424664 0.528985

C -1.976807 2.581547 3.789571

H -0.738315 1.122278 4.786850

H -3.184433 3.845606 2.527336

H -2.090047 3.209792 4.668735

C -2.741629 -1.504958 0.439168

C -3.161282 -2.344246 -0.607607

C -3.289179 -1.696311 1.715274

C -4.108765 -3.340233 -0.380873

H -2.743104 -2.225303 -1.600391

C -4.233473 -2.701636 1.940914

H -2.995013 -1.059267 2.541739

C -4.646698 -3.524956 0.894757

H -4.417724 -3.980475 -1.202612

H -4.647954 -2.831713 2.937250

H -5.382246 -4.305579 1.070341

C 3.933296 -2.669450 0.438086

H 3.978519 -3.198071 1.406478

H 3.760476 -3.455908 -0.304380

C 4.377282 0.186240 1.221930

H 4.501582 0.723126 0.279428

H 4.485283 0.928096 2.022439

C 5.280324 -1.963203 0.243489

H 6.087744 -2.704470 0.292937

H 5.336938 -1.501400 -0.751381

C 5.473524 -0.885020 1.319917

H 5.453898 -1.359298 2.313115

H 6.458590 -0.412539 1.219303

**TS4d***-exo-re*

Imaginary frequency: -351.6236 cm-1

Atom X Y Z

C 2.929898 -1.734563 -0.568351

C 3.321133 -0.658016 0.456864

Rh 0.842052 -0.652166 -0.843348

C 2.479248 -0.717946 -2.444962

C 3.208852 0.567348 -2.366077

C 2.840547 1.535138 -1.507229

Cl -0.265138 -2.328804 -2.222813

H 3.013986 -1.532712 -2.926978

H 1.532864 -0.616947 -2.987003

H 4.045989 0.736260 -3.044305

C 3.463956 2.910752 -1.516243

H 3.919236 3.156840 -0.550154

H 2.713622 3.683851 -1.725526

H 4.231794 2.977216 -2.293121

C 2.108157 0.249203 0.588279

H 1.693470 0.429840 1.573593

C 1.850509 1.206395 -0.430805

O 1.005682 2.315663 -0.116043

C 1.378720 3.175237 0.881045

O 2.342486 3.020528 1.594106

C 0.449560 4.365368 0.922297

H 0.760388 5.082832 0.153208

H 0.521345 4.846137 1.899140

H -0.580270 4.072253 0.712137

P -1.272106 -0.101914 0.054858

C -2.239048 1.027033 -1.042229

C -1.589323 1.687248 -2.094446

C -3.618338 1.231269 -0.861066

C -2.298177 2.544543 -2.939112

H -0.530289 1.515630 -2.254440

C -4.323681 2.091012 -1.703146

H -4.145107 0.707587 -0.068867

C -3.664059 2.750668 -2.743604

H -1.781542 3.042601 -3.755177

H -5.389798 2.238191 -1.551945

H -4.216264 3.414194 -3.403919

C -1.277045 0.728241 1.714788

C -0.525901 0.148473 2.752649

C -2.010195 1.890588 1.995087

C -0.515491 0.706347 4.031763

H 0.050474 -0.752294 2.559990

C -1.993471 2.454007 3.273849

H -2.601357 2.360946 1.216918

C -1.248288 1.865087 4.295555

H 0.070171 0.238468 4.818435

H -2.568821 3.355337 3.468824

H -1.236435 2.305213 5.288832

C -2.469658 -1.498562 0.322938

C -3.152661 -2.034597 -0.783126

C -2.696755 -2.061289 1.587667

C -4.038576 -3.098758 -0.621784

H -2.986388 -1.627097 -1.772439

C -3.579231 -3.132870 1.744045

H -2.202830 -1.661643 2.465781

C -4.252832 -3.655144 0.640628

H -4.554442 -3.498651 -1.490469

H -3.742840 -3.550089 2.734295

H -4.941223 -4.487272 0.762766

H 4.131065 -0.063317 0.018447

H 3.800413 -2.021059 -1.156162

C 3.870999 -1.245662 1.778930

H 4.922197 -1.520301 1.613040

H 3.876028 -0.455173 2.540350

C 2.270104 -3.012442 -0.039048

H 1.240090 -2.846942 0.294082

H 2.212891 -3.750295 -0.844670

C 3.130724 -2.493418 2.276902

H 2.108511 -2.234263 2.590477

H 3.638206 -2.890526 3.165280

C 3.064125 -3.553595 1.171166

H 4.084201 -3.832581 0.867840

H 2.579092 -4.468505 1.533514

**TS5d***-endo-re*

Imaginary frequency: -333.2025 cm-1

Atom X Y Z

C 1.298344 1.818324 0.053260

C 2.097488 2.370574 -1.158277

C 3.122770 1.359790 -1.686653

C 2.535009 -0.003576 -2.001681

C 2.653903 -1.098109 -1.134534

Rh 0.544541 -0.154185 -0.917940

Cl -0.361778 -1.292418 -2.832464

H 3.929867 1.242446 -0.953528

H 3.577394 1.764558 -2.599848

H 2.380286 -0.240867 -3.051540

C 2.693481 -0.834910 0.304602

C 1.703130 -0.007990 0.743991

H 1.481098 0.091431 1.800644

C 2.809437 -2.510917 -1.641302

H 3.877898 -2.734141 -1.764873

H 2.383718 -3.245666 -0.950144

H 2.319488 -2.618943 -2.612020

O 3.436924 -1.626521 1.186531

C 4.791495 -1.711817 0.989132

O 5.384179 -1.105767 0.129043

C 5.404221 -2.659984 1.990284

H 5.145729 -2.354513 3.009164

H 5.004348 -3.668771 1.840985

H 6.487329 -2.669995 1.864449

P -1.585542 -0.215668 0.087166

H 0.234164 2.045140 -0.085439

H 1.366083 2.534524 -1.960449

C -1.617629 0.269710 1.877385

C -1.490614 -0.700717 2.883953

C -1.665195 1.622859 2.255374

C -1.420320 -0.329814 4.228427

H -1.455959 -1.752770 2.619109

C -1.601609 1.992040 3.600276

H -1.771579 2.395073 1.499049

C -1.477684 1.016561 4.591785

H -1.328548 -1.097867 4.991841

H -1.651783 3.043399 3.871397

H -1.429807 1.303979 5.638610

C -2.881109 0.865697 -0.669149

C -2.749093 1.224710 -2.020772

C -4.000846 1.322358 0.048597

C -3.717266 2.020810 -2.636627

H -1.899871 0.860117 -2.589921

C -4.964460 2.119084 -0.571387

H -4.117873 1.067793 1.097386

C -4.824120 2.470755 -1.915638

H -3.602603 2.286610 -3.684007

H -5.823242 2.465366 -0.002228

H -5.574334 3.092576 -2.397086

C -2.323781 -1.909619 0.116909

C -3.703395 -2.121087 0.256722

C -1.469222 -3.019463 0.042958

C -4.215380 -3.416907 0.331629

H -4.385027 -1.277570 0.294402

C -1.982853 -4.314659 0.127899

H -0.405554 -2.863127 -0.105631

C -3.356250 -4.516127 0.272011

H -5.287191 -3.566202 0.432211

H -1.309308 -5.164997 0.063628

H -3.757144 -5.524776 0.327789

C 1.710369 2.497227 1.373954

H 2.773247 2.314649 1.572590

H 1.155063 2.089399 2.222517

C 2.761492 3.731208 -0.846468

H 3.684014 3.574826 -0.269641

H 3.065198 4.201651 -1.790201

C 1.442017 4.020544 1.303333

C 1.810674 4.644283 -0.066888

H 0.902534 4.795754 -0.666091

H 2.253487 5.637561 0.072094

H 2.016353 4.499094 2.106713

H 0.384700 4.211990 1.526531

**TS5d***-exo-re*

Imaginary frequency: -336.4316 cm-1

Atom X Y Z

C 1.396472 1.970176 1.037640

C 2.171885 2.975789 0.145333

C 3.205157 2.263486 -0.728852

C 2.646897 1.082753 -1.510385

C 2.794153 -0.254675 -1.078055

Rh 0.686091 0.463744 -0.684858

Cl -0.112276 0.305345 -2.959469

H 4.027717 1.908646 -0.096428

H 3.638656 2.983910 -1.434075

H 2.561003 1.213574 -2.586311

C 2.776491 -0.540095 0.358211

C 1.728141 0.038871 1.015455

H 1.440596 -0.253336 2.021760

C 3.061274 -1.372004 -2.058439

H 4.141423 -1.441773 -2.244599

H 2.717346 -2.342244 -1.684597

H 2.560125 -1.161246 -3.006810

O 3.514892 -1.585151 0.922258

C 4.877870 -1.570582 0.762482

O 5.477683 -0.688390 0.196836

C 5.487851 -2.800125 1.388873

H 5.119230 -3.698851 0.882768

H 6.573868 -2.748484 1.305457

H 5.193983 -2.874735 2.440742

P -1.433637 -0.424588 0.043002

C -1.518801 -0.936410 1.827896

C -1.012129 -2.190250 2.209697

C -1.970726 -0.068406 2.836318

C -0.961890 -2.564103 3.553511

H -0.661392 -2.885498 1.453231

C -1.924680 -0.445287 4.180497

H -2.378501 0.902290 2.575060

C -1.418319 -1.693662 4.544885

H -0.570385 -3.541581 3.822782

H -2.290260 0.239414 4.941447

H -1.384004 -1.987025 5.590603

C -2.948503 0.608993 -0.205440

C -2.913608 1.600585 -1.200253

C -4.135638 0.426735 0.526109

C -4.038222 2.388847 -1.453291

H -2.013744 1.729981 -1.793776

C -5.256168 1.219115 0.271661

H -4.188053 -0.328404 1.304057

C -5.209629 2.203244 -0.717953

H -3.995295 3.146697 -2.230908

H -6.164948 1.065504 0.847785

H -6.082764 2.819749 -0.914891

C -1.819478 -2.021357 -0.815954

C -3.120695 -2.542361 -0.873213

C -0.770669 -2.757842 -1.384888

C -3.364121 -3.775753 -1.478373

H -3.952847 -1.983604 -0.458320

C -1.014005 -3.996179 -1.981738

H 0.232390 -2.345375 -1.379086

C -2.311030 -4.507862 -2.030477

H -4.379198 -4.161819 -1.521281

H -0.190313 -4.550935 -2.423177

H -2.502686 -5.467211 -2.503917

H 2.731902 3.628595 0.835778

H 1.989312 1.852862 1.948844

C 1.201930 3.882099 -0.627596

H 1.767381 4.530547 -1.308783

H 0.555992 3.257626 -1.257401

C 0.020715 2.501626 1.492132

H -0.766473 2.315794 0.757558

H -0.277064 1.998288 2.416489

C 0.353894 4.741170 0.343858

H -0.610506 4.974555 -0.124123

H 0.849046 5.703009 0.526944

C 0.115754 4.025702 1.694925

H -0.804287 4.394391 2.164422

H 0.930267 4.251066 2.397152

**14e**

Atom X Y Z

C -3.566981 -2.030031 -1.814233

C -2.502330 -1.033803 -2.270598

Rh -0.819996 -1.123111 0.335358

C -3.085896 -2.968157 -0.708795

C -2.698203 -2.305481 0.597754

C -2.883052 -0.968011 1.012813

Cl 0.397500 -2.855146 1.450931

H -3.889497 -3.684265 -0.475471

H -2.243376 -3.577857 -1.064174

H -2.624327 -3.012879 1.422381

C -3.217676 -0.638134 2.450056

H -4.294411 -0.436521 2.537807

H -2.681591 0.242494 2.810436

H -2.982457 -1.488531 3.095738

C -1.960366 -0.123101 -1.183101

H -1.498131 0.776818 -1.584695

C -2.524414 0.102106 0.104354

O -2.572299 1.406824 0.624544

C -3.429672 2.288046 0.010435

O -4.178876 1.980525 -0.883603

C -3.278901 3.660527 0.616877

H -3.374554 3.610982 1.705963

H -4.037271 4.325143 0.201556

H -2.279487 4.048466 0.391383

P 1.183224 0.085011 -0.057364

H -3.889408 -2.628098 -2.675659

H -1.665618 -1.567103 -2.743370

C 2.201011 0.327744 1.465723

C 1.568880 0.261855 2.716096

C 3.573971 0.611086 1.410751

C 2.292699 0.491085 3.887690

H 0.515289 0.006046 2.766968

C 4.297085 0.831471 2.583381

H 4.085919 0.648591 0.454452

C 3.657531 0.775208 3.823748

H 1.791432 0.429307 4.849840

H 5.361807 1.042182 2.527084

H 4.223307 0.942976 4.736417

C 0.960537 1.817053 -0.678731

C 0.658563 2.054531 -2.031746

C 0.995035 2.910422 0.200070

C 0.409115 3.348180 -2.492539

H 0.637696 1.227161 -2.735773

C 0.743999 4.204978 -0.261961

H 1.228310 2.754579 1.248361

C 0.450782 4.428832 -1.608558

H 0.185634 3.510477 -3.543580

H 0.790177 5.039779 0.432800

H 0.261687 5.436688 -1.968082

C 2.347268 -0.659422 -1.288845

C 2.331281 -2.052475 -1.468498

C 3.263206 0.109169 -2.028500

C 3.211084 -2.658457 -2.367565

H 1.644980 -2.660421 -0.887473

C 4.139128 -0.501411 -2.927491

H 3.289853 1.188260 -1.913061

C 4.114004 -1.886783 -3.100290

H 3.187331 -3.737658 -2.492494

H 4.839747 0.107627 -3.492816

H 4.794908 -2.360866 -3.802316

H -2.931598 -0.385734 -3.049554

H -4.449107 -1.476312 -1.466492

**TS2e**

Imaginary frequency: -358.7646 cm-1

Atom X Y Z

C -1.619812 -2.906158 -1.467868

C -2.303744 -1.700055 -1.865935

Rh -0.304760 -1.487556 -0.562262

C -0.870109 -2.011801 1.365773

C -2.315990 -2.130360 1.537378

C -3.237426 -1.163111 1.225761

Cl 1.493604 -3.121316 -0.679136

H -0.333061 -2.932612 1.598072

H -0.453660 -1.175730 1.933501

H -2.689246 -3.042812 2.006312

C -4.651049 -1.215180 1.762443

H -5.395453 -1.161331 0.959432

H -4.842105 -0.372931 2.438768

H -4.815973 -2.138358 2.325534

C -1.933189 -0.214668 -0.688421

H -1.840984 0.675490 -1.311433

C -2.884078 -0.119202 0.307601

O -3.673595 1.049767 0.392332

C -4.601751 1.272744 -0.585213

O -4.868846 0.472060 -1.451790

C -5.232385 2.632073 -0.410129

H -5.691737 2.710049 0.580772

H -5.985799 2.786999 -1.182998

H -4.461572 3.407230 -0.474774

P 1.273213 0.290239 0.038054

H -0.934053 -3.386291 -2.161929

H -2.000844 -1.251082 -2.811413

C 0.506182 1.972799 0.055670

C -0.431887 2.285315 1.056447

C 0.729168 2.908698 -0.966365

C -1.107769 3.505346 1.047486

H -0.629765 1.575817 1.854660

C 0.045108 4.127074 -0.978972

H 1.445851 2.692426 -1.752026

C -0.871406 4.431444 0.028519

H -1.821116 3.728776 1.836142

H 0.237978 4.841155 -1.775373

H -1.395336 5.383692 0.021938

C 2.676752 0.479260 -1.146112

C 3.802990 1.259881 -0.838891

C 2.609128 -0.152808 -2.395817

C 4.831303 1.413863 -1.768128

H 3.881057 1.742238 0.131059

C 3.637571 0.005705 -3.327535

H 1.767874 -0.798090 -2.629360

C 4.748729 0.789314 -3.015672

H 5.699474 2.017187 -1.516185

H 3.574888 -0.497643 -4.288365

H 5.553070 0.906491 -3.737097

C 2.107260 0.167931 1.684346

C 2.534690 -1.103634 2.105392

C 2.358711 1.278511 2.506664

C 3.199934 -1.253639 3.323088

H 2.354697 -1.967442 1.470496

C 3.020668 1.120328 3.726167

H 2.036717 2.269001 2.201580

C 3.441886 -0.145359 4.137365

H 3.527162 -2.242110 3.634345

H 3.207145 1.988930 4.352502

H 3.956225 -0.266814 5.087135

H -3.374321 -1.625739 -1.710755

H -2.149747 -3.598178 -0.818814

**TS3e**

Imaginary frequency: -251.6372 cm-1

Atom X Y Z

C -0.011184 -1.960510 -2.198299

C 1.066717 -2.918370 -1.962904

C 2.398692 -2.624870 -0.543941

C 2.358000 -1.953981 0.788512

C 2.677741 -0.627105 1.007282

Rh 0.501174 -1.247491 -0.332446

Cl -0.742269 -3.065062 0.833871

H 3.172928 -2.189985 -1.171166

H 2.507621 -3.697728 -0.406636

H 2.158765 -2.588854 1.647705

C 2.768432 0.274545 -0.165566

C 1.787763 0.154133 -1.063329

H 1.714217 0.769746 -1.957844

C 2.969258 -0.097947 2.386550

H 4.044745 0.100181 2.489215

H 2.446777 0.845933 2.579057

H 2.691272 -0.822706 3.157777

O 3.785945 1.252604 -0.217811

C 5.075996 0.817350 -0.297306

O 5.394194 -0.348960 -0.338250

C 6.026783 1.991033 -0.327213

H 5.804981 2.630468 -1.187998

H 5.900299 2.602259 0.572479

H 7.052748 1.626573 -0.388966

P -1.183006 0.279115 -0.028286

H -1.023305 -2.357884 -2.159681

H 0.727831 -3.900531 -1.646474

C -0.750960 1.990999 -0.577377

C -0.491730 3.019632 0.338545

C -0.598657 2.257036 -1.949958

C -0.098203 4.284924 -0.106274

H -0.599495 2.839064 1.402797

C -0.216840 3.522483 -2.392595

H -0.788774 1.472949 -2.678197

C 0.036771 4.541308 -1.470092

H 0.098220 5.070050 0.618991

H -0.113673 3.711637 -3.457797

H 0.338382 5.526804 -1.814432

C -2.790914 -0.060927 -0.886517

C -3.301368 -1.370447 -0.859462

C -3.545951 0.950372 -1.502801

C -4.536176 -1.655591 -1.443684

H -2.735205 -2.158494 -0.370579

C -4.779905 0.656647 -2.088149

H -3.178357 1.970351 -1.531144

C -5.277365 -0.646422 -2.061872

H -4.917206 -2.672888 -1.413478

H -5.350293 1.451035 -2.562539

H -6.237335 -0.873563 -2.518120

C -1.702580 0.500905 1.731344

C -2.860618 1.227780 2.053685

C -0.931383 -0.045945 2.764814

C -3.228302 1.414775 3.385534

H -3.480838 1.642137 1.264393

C -1.301919 0.142485 4.098404

H -0.060000 -0.642988 2.520862

C -2.447958 0.873565 4.410915

H -4.127824 1.976809 3.622180

H -0.700789 -0.296312 4.890086

H -2.739128 1.014535 5.448460

H 1.856484 -2.942133 -2.711259

H 0.141803 -1.226331 -2.990134

**TS4e**

Imaginary frequency: -408.6729 cm-1

Atom X Y Z

C -2.190958 -2.703392 -1.415426

C -2.758213 -1.431215 -2.056571

Rh -0.770734 -1.442389 -0.182366

C -2.368157 -2.752833 0.703740

C -3.591308 -1.948470 0.937632

C -3.593658 -0.622712 0.721306

Cl 0.721945 -3.305105 0.095068

H -2.508363 -3.827776 0.623831

H -1.629345 -2.599849 1.504409

H -4.487054 -2.445477 1.309706

C -4.765058 0.261957 1.074613

H -5.144784 0.798326 0.198347

H -4.478177 1.020488 1.814135

H -5.576082 -0.332048 1.506923

C -2.106756 -0.268120 -1.333354

H -1.666641 0.530831 -1.919505

C -2.419287 -0.006225 0.019550

O -2.079416 1.280892 0.536537

C -2.645308 2.393728 -0.025977

O -3.405484 2.371756 -0.965440

C -2.172630 3.640150 0.683302

H -2.023165 3.458994 1.750315

H -2.896682 4.441023 0.523037

H -1.211702 3.944102 0.254247

P 1.052831 0.067748 -0.011611

H -1.293460 -3.109282 -1.884700

H -2.506393 -1.405753 -3.123736

C 1.260012 0.790053 1.679253

C 0.248639 0.626151 2.635996

C 2.431964 1.477298 2.042589

C 0.395920 1.151779 3.921964

H -0.650553 0.081545 2.370754

C 2.574231 2.007829 3.324686

H 3.241055 1.585774 1.326355

C 1.555071 1.847038 4.267183

H -0.394399 1.009491 4.654271

H 3.485442 2.537184 3.590308

H 1.670566 2.253418 5.268532

C 0.969867 1.520879 -1.159080

C 0.702911 1.269523 -2.516916

C 1.183180 2.846242 -0.754048

C 0.660949 2.312129 -3.443778

H 0.536027 0.248219 -2.850517

C 1.133641 3.890835 -1.681787

H 1.393027 3.068786 0.286707

C 0.873066 3.628079 -3.027131

H 0.457951 2.095553 -4.489072

H 1.304491 4.911548 -1.349281

H 0.834809 4.441667 -3.746155

C 2.760777 -0.584364 -0.327602

C 3.333123 -1.447318 0.623120

C 3.499660 -0.259551 -1.473284

C 4.613458 -1.962255 0.432190

H 2.776233 -1.723076 1.511190

C 4.780455 -0.784795 -1.665729

H 3.090571 0.412044 -2.219405

C 5.341075 -1.635605 -0.714512

H 5.037495 -2.629627 1.177420

H 5.338422 -0.518918 -2.559774

H 6.337848 -2.042213 -0.863860

H -3.851253 -1.393376 -1.974547

H -2.929165 -3.495603 -1.325812

**TS5e**

Imaginary frequency: -372.3828 cm-1

Atom X Y Z

C 1.116963 -1.172590 -2.029704

C 1.665860 -2.602112 -1.964504

C 2.886232 -2.694187 -1.040740

C 2.607894 -2.132462 0.346092

C 2.942778 -0.829217 0.738591

Rh 0.678435 -0.915950 0.200955

Cl -0.012755 -1.816257 2.313260

H 3.729855 -2.152345 -1.484380

H 3.192661 -3.742099 -0.942598

H 2.505186 -2.855398 1.151860

C 2.922633 0.241521 -0.257803

C 1.789941 0.282167 -1.020202

H 1.545824 1.145499 -1.633071

C 3.370048 -0.516208 2.152781

H 4.457544 -0.642384 2.240626

H 3.119521 0.509814 2.441078

H 2.884032 -1.203442 2.849822

O 3.790484 1.335627 -0.187661

C 5.139388 1.088922 -0.236575

O 5.615242 -0.011519 -0.379455

C 5.908988 2.377567 -0.082695

H 5.582427 3.106495 -0.831165

H 5.713168 2.812672 0.903342

H 6.975427 2.178282 -0.192401

P -1.400540 0.155639 -0.013900

H 0.042928 -1.145721 -2.227151

H 0.887421 -3.272080 -1.586631

C -1.468411 1.381519 -1.403867

C -1.167116 2.734171 -1.178266

C -1.698430 0.959268 -2.725289

C -1.103144 3.637860 -2.240847

H -0.987935 3.086853 -0.167436

C -1.636681 1.864362 -3.786252

H -1.950082 -0.077568 -2.929112

C -1.337537 3.207191 -3.547746

H -0.874782 4.681807 -2.042864

H -1.827367 1.518329 -4.798759

H -1.291297 3.912203 -4.373298

C -2.861188 -0.933012 -0.323926

C -2.817707 -2.259401 0.136581

C -4.018793 -0.478334 -0.980072

C -3.909529 -3.108249 -0.056967

H -1.936940 -2.613905 0.662731

C -5.105899 -1.332149 -1.172924

H -4.070764 0.539762 -1.353574

C -5.052964 -2.649530 -0.712586

H -3.861908 -4.130875 0.307394

H -5.992584 -0.966697 -1.684361

H -5.899282 -3.314127 -0.865295

C -1.857239 1.174008 1.457109

C -3.183840 1.537900 1.731876

C -0.836197 1.627196 2.305246

C -3.480446 2.348051 2.828436

H -3.991021 1.179623 1.101034

C -1.134676 2.445330 3.396177

H 0.186449 1.314230 2.119850

C -2.456644 2.806569 3.660192

H -4.513212 2.616398 3.034716

H -0.334588 2.784315 4.048690

H -2.690231 3.434638 4.515861

H 1.927893 -2.941545 -2.978437

H 1.575495 -0.632986 -2.862129

**10e**

Atom X Y Z

C -1.921240 -3.199624 -0.806743

C -1.947311 -2.323144 -1.849811

Rh -0.430045 -1.277763 -0.371568

C -0.875925 -1.497819 1.662387

C -2.312056 -1.699669 1.912557

C -3.294521 -0.886335 1.437820

Cl 1.292583 -3.013088 -0.770275

H -0.256539 -2.337735 1.986877

H -0.514870 -0.572813 2.120179

H -2.600298 -2.559656 2.518015

C -4.732670 -1.040438 1.864688

H -5.389027 -1.217323 1.003003

H -5.095354 -0.131116 2.359627

H -4.846270 -1.874801 2.563082

C -1.838943 0.153913 -0.310762

H -1.725895 1.023282 -0.963846

C -2.959795 0.124821 0.442295

O -3.940664 1.147887 0.285754

C -4.760564 1.081173 -0.799478

O -4.737569 0.197761 -1.626384

C -5.700591 2.264996 -0.815597

H -6.278692 2.302049 0.113456

H -6.373028 2.184943 -1.670406

H -5.126750 3.195616 -0.879063

P 1.270454 0.282267 0.036530

H -1.157192 -3.965133 -0.731521

H -1.206628 -2.395587 -2.646106

C 0.707124 2.039433 0.145681

C -0.110781 2.434890 1.219981

C 1.018701 2.982123 -0.844995

C -0.589432 3.741247 1.306134

H -0.371956 1.723004 1.996335

C 0.529626 4.288622 -0.761159

H 1.647462 2.702598 -1.683341

C -0.272218 4.672026 0.313545

H -1.215866 4.028941 2.145940

H 0.783656 5.005864 -1.537000

H -0.648852 5.689241 0.378953

C 2.478252 0.298441 -1.353106

C 3.831766 0.604082 -1.153380

C 2.023189 0.031497 -2.653508

C 4.711922 0.642217 -2.236362

H 4.203109 0.804335 -0.153430

C 2.902618 0.077609 -3.734724

H 0.981617 -0.228496 -2.822432

C 4.249898 0.380983 -3.527215

H 5.760467 0.872337 -2.067878

H 2.537940 -0.138639 -4.734991

H 4.937864 0.406742 -4.367924

C 2.290297 0.049918 1.558006

C 2.650925 -1.253265 1.942403

C 2.748178 1.138784 2.318738

C 3.449403 -1.455583 3.068665

H 2.319501 -2.099975 1.348271

C 3.544978 0.927403 3.445786

H 2.485658 2.152777 2.037172

C 3.895096 -0.369363 3.824526

H 3.721988 -2.468127 3.353615

H 3.890627 1.779217 4.025477

H 4.513611 -0.532046 4.703276

H -2.787375 -1.653897 -2.006504

H -2.737283 -3.226621 -0.094740

**11e**

Atom X Y Z

C -0.578494 -2.031890 -2.309740

C 0.428011 -2.984566 -2.112472

C 2.015864 -2.862848 0.167025

C 2.023811 -1.868995 1.218490

C 2.445044 -0.551080 1.030151

Rh 0.428970 -1.606695 -0.425054

Cl -1.157753 -3.100597 0.795223

H 2.829084 -2.833863 -0.555845

H 1.688142 -3.858298 0.454745

H 1.667255 -2.150916 2.206195

C 2.728861 -0.078364 -0.333496

C 1.858439 -0.468207 -1.277329

H 1.941070 -0.144701 -2.315359

C 2.615487 0.385516 2.193221

H 3.682215 0.441634 2.446111

H 2.276061 1.397333 1.948405

H 2.078484 0.028325 3.075796

O 3.742911 0.869760 -0.567657

C 5.006274 0.596325 -0.131239

O 5.301015 -0.386829 0.509064

C 5.955702 1.691450 -0.553838

H 5.986151 1.758362 -1.646469

H 5.607290 2.659142 -0.178426

H 6.952657 1.476929 -0.167576

P -1.098753 0.354190 -0.030568

H -1.611071 -2.276006 -2.085347

H 0.174428 -3.963872 -1.718047

C -0.342750 1.960848 -0.543553

C 0.034561 2.952004 0.374874

C -0.033445 2.156862 -1.901950

C 0.685885 4.111948 -0.054895

H -0.188559 2.828403 1.429195

C 0.603981 3.320019 -2.331370

H -0.302896 1.398098 -2.631757

C 0.967792 4.302592 -1.407173

H 0.965140 4.869460 0.672854

H 0.821334 3.455995 -3.387513

H 1.468511 5.207608 -1.740274

C -2.731866 0.323588 -0.904800

C -3.514097 -0.837650 -0.766667

C -3.236463 1.399478 -1.650234

C -4.771486 -0.913321 -1.365722

H -3.134210 -1.678416 -0.190870

C -4.495022 1.314119 -2.252242

H -2.655661 2.308812 -1.763858

C -5.264971 0.159518 -2.112378

H -5.364434 -1.816567 -1.249113

H -4.871621 2.156145 -2.827406

H -6.243578 0.095777 -2.580856

C -1.634778 0.653048 1.714514

C -2.558047 1.668468 2.020349

C -1.138286 -0.147683 2.751219

C -2.952644 1.892638 3.338288

H -2.972941 2.282256 1.225892

C -1.539274 0.075612 4.071503

H -0.475801 -0.972641 2.517639

C -2.440748 1.097260 4.367861

H -3.665892 2.681888 3.560868

H -1.156356 -0.562102 4.863702

H -2.754807 1.267723 5.394277

H 1.372196 -2.911692 -2.645323

H -0.428197 -1.209828 -3.006123

**12e**

Atom X Y Z

C 2.187327 -3.202504 0.411385

C 3.480790 -2.408758 0.298709

Rh 0.831786 -1.633800 0.105566

C 1.059729 -1.880863 -1.917642

C 2.114487 -0.974995 -2.466270

C 2.759685 -0.073333 -1.713362

Cl -0.851370 -3.267445 0.430737

H 1.276166 -2.935460 -2.124444

H 0.074916 -1.657546 -2.345607

H 2.355399 -1.062919 -3.528963

C 3.770933 0.911620 -2.247971

H 4.732232 0.821957 -1.729550

H 3.427228 1.944381 -2.106252

H 3.931219 0.756934 -3.319378

C 2.900976 -1.053512 0.644400

H 2.878333 -0.790030 1.702047

C 2.487491 -0.074403 -0.247332

O 2.207995 1.204976 0.302897

C 3.237762 1.903099 0.885726

O 4.367305 1.486992 0.981148

C 2.741356 3.239850 1.377425

H 2.235612 3.778028 0.570409

H 3.583461 3.819712 1.757130

H 2.004633 3.093100 2.174594

P -1.055647 0.174644 -0.024690

H 1.989526 -3.579431 1.419482

H 4.286468 -2.697881 0.985981

C -0.728091 1.965922 -0.383564

C -0.120038 2.287724 -1.608634

C -1.086675 3.008043 0.482540

C 0.115881 3.615828 -1.960797

H 0.178923 1.494383 -2.288154

C -0.845539 4.340235 0.131441

H -1.561899 2.785229 1.432488

C -0.245987 4.648733 -1.089987

H 0.582485 3.845363 -2.915305

H -1.135458 5.135639 0.813373

H -0.064715 5.684450 -1.364937

C -1.703928 0.196224 1.701816

C -3.038783 -0.072150 2.029894

C -0.780860 0.419836 2.739346

C -3.443114 -0.109481 3.366809

H -3.762887 -0.259591 1.243956

C -1.188659 0.392139 4.072582

H 0.259666 0.626316 2.497531

C -2.522945 0.123649 4.389290

H -4.481012 -0.324339 3.606709

H -0.464746 0.571301 4.863282

H -2.840818 0.091981 5.427866

C -2.542641 -0.099445 -1.102584

C -2.779135 -1.359235 -1.675685

C -3.440076 0.947690 -1.380869

C -3.883785 -1.563196 -2.506628

H -2.119848 -2.188230 -1.446501

C -4.543455 0.738397 -2.207830

H -3.275228 1.932960 -0.956989

C -4.766447 -0.517795 -2.776740

H -4.050620 -2.546007 -2.939341

H -5.226206 1.559486 -2.410569

H -5.623878 -0.678623 -3.425083

H 3.878277 -2.409775 -0.720145

H 2.030454 -3.995135 -0.321491

**13e**

Atom X Y Z

C 0.425837 -1.548699 -1.906359

C 1.430135 -2.672790 -2.127306

C 2.816009 -2.247047 -1.618276

C 2.784947 -2.023472 -0.127910

C 3.060507 -0.867135 0.565606

Rh 0.642966 -0.858980 0.029828

Cl -0.156290 -2.761056 1.371015

H 3.147613 -1.341619 -2.136094

H 3.552429 -3.036731 -1.823476

H 2.715257 -2.931548 0.469813

C 2.956812 0.460390 -0.096214

C 1.734525 0.716472 -0.583872

H 1.453022 1.655679 -1.057608

C 3.434106 -0.912384 2.030690

H 4.525246 -0.829490 2.124663

H 2.987107 -0.086973 2.595517

H 3.125488 -1.858224 2.484522

O 3.973025 1.424962 0.011895

C 5.272069 1.061358 -0.199156

O 5.627737 -0.059026 -0.481480

C 6.180001 2.256224 -0.026205

H 5.897584 3.047637 -0.728269

H 6.076778 2.665588 0.984272

H 7.213173 1.954348 -0.201304

P -1.374672 0.230953 -0.012370

H -0.606483 -1.896559 -2.007287

H 1.106222 -3.574796 -1.595242

C -1.393015 1.816877 -0.962770

C -1.571254 3.063976 -0.348054

C -1.195044 1.770463 -2.354263

C -1.549547 4.237992 -1.106670

H -1.730953 3.124731 0.723137

C -1.178638 2.942248 -3.108978

H -1.060269 0.815012 -2.852004

C -1.353449 4.181162 -2.486102

H -1.690206 5.196304 -0.613925

H -1.027149 2.887545 -4.183622

H -1.337544 5.094720 -3.074175

C -2.878490 -0.637510 -0.634751

C -2.991544 -2.030058 -0.487541

C -3.934283 0.077536 -1.226835

C -4.140263 -2.689044 -0.930289

H -2.189730 -2.588360 -0.012900

C -5.079060 -0.588882 -1.665683

H -3.866338 1.153508 -1.348484

C -5.183167 -1.973870 -1.521004

H -4.214767 -3.766496 -0.811217

H -5.887253 -0.023914 -2.122514

H -6.073786 -2.491887 -1.867067

C -1.763420 0.722486 1.719088

C -3.035983 0.555413 2.281714

C -0.731128 1.257390 2.509202

C -3.271719 0.920366 3.608896

H -3.841772 0.133867 1.690103

C -0.972473 1.627518 3.831681

H 0.263350 1.385571 2.088062

C -2.243721 1.456819 4.385059

H -4.261152 0.780158 4.035547

H -0.165511 2.039215 4.431660

H -2.429641 1.736019 5.418571

H 1.478338 -2.924095 -3.199674

H 0.605877 -0.721706 -2.600494

**10f***-exo-re*

Atom X Y Z

C -2.313381 -2.975663 0.034335

C -2.203172 -2.416648 -1.206833

Rh -0.453797 -1.103616 -0.007600

C -0.839286 -0.797307 2.022910

C -2.276164 -0.734179 2.354593

C -3.186800 0.041375 1.710626

Cl 1.146755 -2.988802 0.094907

H -0.321096 -1.619431 2.523855

H -0.337076 0.144478 2.262184

H -2.622865 -1.357708 3.179957

C -4.604934 0.180359 2.206317

H -5.328664 -0.136692 1.444435

H -4.836795 1.224273 2.451052

H -4.764598 -0.422113 3.105522

C -1.736474 0.414889 -0.297763

H -1.583388 1.062641 -1.165553

C -2.806857 0.709560 0.468864

O -3.683528 1.765764 0.077228

C -4.576857 1.517083 -0.918298

O -4.704697 0.452243 -1.479325

C -5.385106 2.759920 -1.216074

H -5.897891 3.103411 -0.311708

H -6.113799 2.542179 -1.997675

H -4.720857 3.567950 -1.540315

P 1.364387 0.352794 -0.000841

C 0.947407 2.114552 -0.377108

C 0.169397 2.849817 0.535969

C 1.334088 2.728585 -1.577521

C -0.193478 4.167509 0.261741

H -0.152528 2.394736 1.466967

C 0.960406 4.046524 -1.854431

H 1.932870 2.184370 -2.299681

C 0.199874 4.769720 -0.936148

H -0.790259 4.720270 0.981986

H 1.271840 4.505762 -2.788798

H -0.086728 5.795681 -1.151041

C 2.584153 -0.080967 -1.312405

C 3.939364 0.260028 -1.193366

C 2.142763 -0.722346 -2.478917

C 4.832796 -0.033090 -2.224999

H 4.301544 0.747225 -0.293542

C 3.035530 -1.007542 -3.511834

H 1.102995 -1.020522 -2.573579

C 4.383117 -0.664704 -3.385826

H 5.881802 0.229337 -2.117456

H 2.680288 -1.511618 -4.406309

H 5.081121 -0.895638 -4.186012

C 2.366312 0.463463 1.548934

C 2.658546 -0.716108 2.255503

C 2.882438 1.684031 2.015576

C 3.446191 -0.667459 3.406039

H 2.280325 -1.666759 1.891318

C 3.668481 1.724389 3.169229

H 2.674775 2.605993 1.483721

C 3.950163 0.550067 3.868240

H 3.664682 -1.587657 3.941157

H 4.059306 2.676579 3.518150

H 4.560620 0.583526 4.766841

H -2.856550 -1.575288 -1.422672

H -3.000021 -2.484886 0.715821

C -1.483056 -3.004772 -2.393469

H -2.206974 -3.537277 -3.027903

H -1.046565 -2.216243 -3.019202

C -1.808714 -4.315018 0.488817

H -1.372891 -4.257378 1.491252

H -2.664166 -5.005519 0.541462

H -1.054821 -4.737916 -0.174383

H -0.688097 -3.695799 -2.108078

**11f***-endo-re*

Atom X Y Z

C -0.481341 -2.817870 -1.065489

C 0.561532 -3.522993 -0.439452

C 2.080665 -2.190720 1.532958

C 2.011299 -0.828533 2.020411

C 2.427077 0.277750 1.276969

Rh 0.476580 -1.457009 0.394516

Cl -1.105695 -2.307912 2.136145

H 2.929740 -2.442228 0.900575

H 1.786974 -2.962945 2.239898

H 1.632161 -0.652638 3.024271

C 2.712759 0.105957 -0.148117

C 1.888055 -0.709916 -0.830477

H 2.005198 -0.847117 -1.905437

C 2.568838 1.632280 1.912948

H 3.627639 1.804274 2.146628

H 2.240710 2.430853 1.239594

H 2.004550 1.692877 2.847570

O 3.679620 0.909936 -0.784248

C 4.951306 0.912257 -0.290870

O 5.290921 0.314780 0.704766

C 5.848707 1.762564 -1.157738

H 5.907562 1.335066 -2.164422

H 5.435141 2.771307 -1.256473

H 6.844996 1.806259 -0.716514

P -1.146403 0.432958 -0.089881

H -1.466837 -2.924892 -0.619343

H 0.288711 -4.094261 0.445128

C -0.438880 1.785207 -1.136197

C -0.239861 3.087793 -0.653223

C 0.002968 1.480624 -2.436641

C 0.369058 4.059934 -1.451974

H -0.568754 3.351171 0.346149

C 0.596706 2.455381 -3.237840

H -0.124932 0.476318 -2.829603

C 0.784051 3.749606 -2.746764

H 0.510214 5.063580 -1.059196

H 0.919246 2.201190 -4.244143

H 1.251561 4.508041 -3.368935

C -2.719531 -0.036996 -0.947880

C -3.482262 -1.063849 -0.361157

C -3.192480 0.580044 -2.115712

C -4.689334 -1.459564 -0.937075

H -3.127074 -1.546962 0.546248

C -4.400400 0.173859 -2.690485

H -2.627358 1.381523 -2.580252

C -5.150753 -0.845634 -2.104502

H -5.269120 -2.251817 -0.470890

H -4.753983 0.662395 -3.594911

H -6.090264 -1.158523 -2.552396

C -1.809269 1.340618 1.380699

C -2.888758 2.231427 1.250097

C -1.236989 1.151291 2.645050

C -3.368926 2.929990 2.357089

H -3.358555 2.375748 0.281557

C -1.719841 1.851266 3.753800

H -0.442382 0.425817 2.767375

C -2.782759 2.742868 3.612095

H -4.205018 3.614611 2.241093

H -1.274220 1.683050 4.730551

H -3.162242 3.281970 4.476156

C -0.496711 -2.468382 -2.535379

H -0.835108 -3.342203 -3.112590

H -1.201150 -1.657935 -2.744855

C 1.803267 -4.004970 -1.148890

H 2.179783 -3.291623 -1.886780

H 2.611811 -4.222332 -0.444190

H 0.486401 -2.188892 -2.923314

H 1.577852 -4.941600 -1.679684

**11f***-exo-re*

Atom X Y Z

C -0.409232 2.422493 1.743843

C 0.672547 3.218930 1.322729

C 2.053992 2.469437 -1.016902

C 2.041251 1.233460 -1.782059

C 2.509675 0.018657 -1.284751

Rh 0.441161 1.520964 -0.093612

Cl -1.047770 2.661925 -1.766871

H 2.892235 2.611777 -0.335241

H 1.756324 3.359598 -1.564683

H 1.692492 1.263116 -2.811337

C 2.699548 -0.118503 0.156950

C 1.820818 0.517109 0.957815

H 1.869985 0.407154 2.042714

C 2.787377 -1.142905 -2.194853

H 3.868563 -1.196737 -2.379732

H 2.477551 -2.095203 -1.751601

H 2.284391 -1.019775 -3.156870

O 3.642800 -1.038262 0.661344

C 4.954840 -0.867858 0.320771

O 5.348278 -0.012659 -0.437611

C 5.815243 -1.893890 1.018486

H 5.723243 -1.782978 2.104036

H 5.479600 -2.904788 0.764628

H 6.855080 -1.760168 0.718645

P -1.126468 -0.505175 0.076865

C -0.694881 -1.493086 1.576267

C 0.382002 -2.395618 1.580865

C -1.329168 -1.209334 2.799291

C 0.789194 -3.018014 2.762709

H 0.909615 -2.616811 0.659595

C -0.917633 -1.828006 3.980550

H -2.159985 -0.510863 2.828887

C 0.140660 -2.738858 3.966169

H 1.619436 -3.718755 2.739029

H -1.429159 -1.599082 4.911742

H 0.458095 -3.223884 4.885232

C -2.978236 -0.391928 0.163246

C -3.624575 0.722105 -0.391884

C -3.754631 -1.446151 0.675813

C -5.020882 0.783493 -0.418664

H -3.034925 1.529023 -0.816489

C -5.146801 -1.376505 0.651734

H -3.274876 -2.323798 1.097756

C -5.784189 -0.259212 0.105360

H -5.507294 1.652610 -0.853523

H -5.733430 -2.197710 1.055567

H -6.869707 -0.207237 0.085054

C -0.954559 -1.659576 -1.364651

C -1.049535 -3.056545 -1.266350

C -0.828236 -1.074547 -2.635878

C -0.990092 -3.852463 -2.412091

H -1.172446 -3.530016 -0.297314

C -0.784966 -1.873926 -3.780042

H -0.784317 0.008120 -2.727787

C -0.856526 -3.264059 -3.671223

H -1.056903 -4.933434 -2.318957

H -0.696843 -1.406054 -4.757145

H -0.816038 -3.885361 -4.562008

H 1.637663 3.022806 1.786442

H -0.194594 1.673131 2.506193

C 0.518994 4.617533 0.772470

H 0.326832 5.315949 1.600847

H 1.434926 4.949190 0.273644

C -1.842436 2.897930 1.719853

H -2.080356 3.440879 0.803274

H -2.545237 2.065484 1.809468

H -0.302929 4.691918 0.058021

H -2.016137 3.569751 2.574971

**12f***-endo-re*

Atom X Y Z

C 2.753497 -2.278138 0.540425

C 3.593229 -1.087623 1.050780

Rh 1.005056 -1.215792 0.003792

C 1.434533 -1.323830 -1.998816

C 2.457394 -0.314836 -2.393321

C 2.813324 0.691336 -1.582913

Cl -0.141271 -3.281941 0.003277

H 1.739737 -2.349395 -2.220320

H 0.477043 -1.142521 -2.504559

C 3.763889 1.798289 -1.973063

H 4.601641 1.875292 -1.271343

H 3.260749 2.773323 -1.972945

H 4.161030 1.628005 -2.978594

C 2.563894 0.032248 0.917438

H 2.148823 0.425277 1.846055

C 2.199515 0.733849 -0.222917

O 1.457937 1.933884 -0.005945

C 2.062285 2.958161 0.679386

O 3.183994 2.906884 1.123804

C 1.119616 4.129986 0.802740

H 0.579252 4.299342 -0.132384

H 1.683699 5.018056 1.091480

H 0.378383 3.906954 1.578300

P -1.327313 -0.009635 -0.057684

H 2.352635 -2.849233 1.385838

H 3.778231 -1.207967 2.126816

C -1.798370 1.475538 -1.062896

C -0.906695 1.957793 -2.031714

C -3.058035 2.087545 -0.940238

C -1.257343 3.038557 -2.845966

H 0.064384 1.490851 -2.148626

C -3.403697 3.171971 -1.746011

H -3.776789 1.705762 -0.221043

C -2.502336 3.651069 -2.700815

H -0.556936 3.396577 -3.596125

H -4.380408 3.635922 -1.636261

H -2.775018 4.491134 -3.334185

C -1.467448 0.516505 1.707532

C -1.257506 -0.475854 2.685560

C -1.670041 1.839933 2.124016

C -1.259868 -0.147939 4.041917

H -1.107572 -1.509993 2.381863

C -1.666295 2.165176 3.483856

H -1.839590 2.618753 1.387714

C -1.460330 1.174881 4.445360

H -1.104732 -0.927953 4.782565

H -1.831888 3.195118 3.790095

H -1.459143 1.430417 5.501468

C -2.825741 -1.062992 -0.312880

C -2.886304 -1.830995 -1.486144

C -3.910214 -1.096056 0.574830

C -4.013000 -2.599125 -1.774073

H -2.041566 -1.842622 -2.168339

C -5.035086 -1.874098 0.289442

H -3.880629 -0.520646 1.494987

C -5.090839 -2.623450 -0.885965

H -4.041605 -3.191841 -2.684349

H -5.867075 -1.892010 0.988759

H -5.965601 -3.229662 -1.105904

C 3.393058 -3.244935 -0.443073

H 4.221178 -3.788889 0.036926

H 2.658132 -3.985988 -0.772065

C 4.954241 -0.817921 0.396925

H 4.886002 -0.721988 -0.688890

H 5.386176 0.107311 0.795222

H 2.910967 -0.400382 -3.384279

H 5.647044 -1.637115 0.619136

H 3.805827 -2.748128 -1.327365

**12f***-exo-re*

Atom X Y Z

C 2.817551 -2.335138 -0.209153

C 3.788297 -1.157038 -0.351115

Rh 1.030145 -1.194006 -0.308203

C 1.207354 -1.101047 -2.346701

C 1.831613 0.184260 -2.783237

C 2.216242 1.134014 -1.919586

Cl -0.152377 -3.254006 -0.370212

H 1.776361 -1.971840 -2.695422

H 0.186874 -1.204724 -2.733613

H 1.975556 0.340165 -3.855640

C 2.781357 2.472180 -2.330352

H 3.762343 2.651470 -1.876259

H 2.124844 3.291623 -2.010223

H 2.880354 2.529495 -3.418661

C 2.870658 -0.098194 0.230174

H 2.880238 -0.001287 1.316364

C 2.111019 0.832216 -0.463097

O 1.529765 1.876350 0.307956

C 2.367560 2.768893 0.931566

O 3.572753 2.707550 0.890129

C 1.559258 3.813710 1.660288

H 0.842292 4.283014 0.980284

H 2.232929 4.560551 2.082165

H 0.978731 3.345281 2.462325

P -1.291347 -0.016929 -0.013959

C -1.552786 1.818465 -0.095574

C -1.226028 2.463841 -1.299843

C -2.091773 2.580492 0.950493

C -1.439459 3.832912 -1.456214

H -0.793205 1.893289 -2.117387

C -2.301562 3.954319 0.795336

H -2.352207 2.104823 1.890861

C -1.977769 4.584319 -0.406976

H -1.184886 4.314116 -2.397021

H -2.724594 4.528736 1.615664

H -2.145679 5.651184 -0.528462

C -1.567348 -0.426039 1.764427

C -2.571768 -1.301198 2.199400

C -0.646112 0.069744 2.706396

C -2.661849 -1.659711 3.546575

H -3.278646 -1.711328 1.486229

C -0.744112 -0.283116 4.052378

H 0.145043 0.740855 2.381084

C -1.753446 -1.151478 4.475859

H -3.444531 -2.341920 3.867356

H -0.029791 0.115173 4.768449

H -1.827001 -1.433365 5.522666

C -2.828567 -0.584976 -0.883563

C -2.767776 -1.620331 -1.827459

C -4.065123 0.041120 -0.643514

C -3.917519 -2.020073 -2.514297

H -1.831912 -2.138470 -1.997059

C -5.211425 -0.364902 -1.324846

H -4.132807 0.855342 0.071624

C -5.139277 -1.395951 -2.265664

H -3.852841 -2.826343 -3.239987

H -6.159403 0.127995 -1.125336

H -6.032376 -1.709157 -2.800153

H 3.936669 -0.942491 -1.414543

H 2.819533 -3.021561 -1.059708

C 5.160246 -1.234562 0.329246

H 5.757258 -2.047015 -0.101376

H 5.711172 -0.296682 0.191704

C 2.882277 -3.102300 1.107142

H 2.084476 -3.846628 1.154845

H 3.844966 -3.627226 1.206271

H 5.072454 -1.417872 1.405750

H 2.783317 -2.450696 1.986135

**13f***-endo-re*

Atom X Y Z

C -0.433677 -2.734988 0.066022

C -1.674714 -3.198807 0.844286

C -2.964470 -2.583387 0.228012

C -2.721367 -1.686249 -0.967848

C -2.945881 -0.315615 -1.004484

Rh -0.654067 -0.715075 -0.516712

Cl 0.061157 -1.281360 -2.827305

H -3.491037 -2.004754 0.991910

H -3.648963 -3.380540 -0.091065

H -2.647968 -2.176324 -1.937334

C -2.842423 0.451238 0.255762

C -1.690835 0.221899 0.922419

H -1.422957 0.736351 1.844217

C -3.267469 0.395260 -2.297083

H -4.357488 0.429270 -2.426121

H -2.889920 1.422801 -2.306903

H -2.833600 -0.143258 -3.143975

O -3.744178 1.476820 0.574775

C -5.087807 1.230495 0.482928

O -5.558360 0.166821 0.155995

C -5.869862 2.469617 0.845211

H -5.594683 2.810126 1.848624

H -5.630828 3.280420 0.148698

H -6.937000 2.249093 0.804179

P 1.399449 0.265873 0.091260

H -0.474092 -3.162365 -0.939586

H -1.702599 -4.293196 0.699251

C 1.565151 0.875090 1.831063

C 2.234908 2.073399 2.128218

C 1.043542 0.114214 2.888336

C 2.379322 2.496480 3.450584

H 2.645644 2.678157 1.325883

C 1.192700 0.535871 4.210847

H 0.505534 -0.802707 2.674725

C 1.859690 1.728787 4.494963

H 2.898370 3.427286 3.663132

H 0.779839 -0.065132 5.016615

H 1.970776 2.060581 5.523762

C 3.022345 -0.557398 -0.226436

C 3.234863 -1.195842 -1.461899

C 4.046495 -0.570215 0.733837

C 4.455318 -1.818139 -1.727992

H 2.437993 -1.218521 -2.200341

C 5.261659 -1.202446 0.462048

H 3.904109 -0.090748 1.695844

C 5.470712 -1.824815 -0.768997

H 4.605408 -2.306457 -2.687080

H 6.043564 -1.205978 1.216905

H 6.417458 -2.315898 -0.978311

C 1.463310 1.834330 -0.889471

C 2.523343 2.151662 -1.748936

C 0.386592 2.731854 -0.778404

C 2.505504 3.339978 -2.483710

H 3.362988 1.473511 -1.852415

C 0.375403 3.919554 -1.508075

H -0.445657 2.502165 -0.118802

C 1.434435 4.225547 -2.366497

H 3.333670 3.569736 -3.148673

H -0.463609 4.603160 -1.408946

H 1.422764 5.148024 -2.940656

C 0.890888 -3.129183 0.694444

H 0.872246 -4.209606 0.915885

H 1.724825 -2.953514 0.013096

C -1.638159 -2.963078 2.359988

H -1.608165 -1.891007 2.586593

H -2.539978 -3.376434 2.827826

H -0.773970 -3.443886 2.831180

H 1.102126 -2.616754 1.638216

**13f***-exo-re*

Atom X Y Z

C -0.660977 -2.204541 0.556683

C -2.106031 -2.460745 1.015173

C -3.067322 -2.400614 -0.190042

C -2.736438 -1.360650 -1.247967

C -2.901266 0.011435 -1.160184

Rh -0.616619 -0.491255 -0.649589

Cl 0.149705 -0.922431 -2.962579

H -4.090153 -2.220579 0.165373

H -3.071073 -3.376914 -0.689361

H -2.649528 -1.744330 -2.262977

C -2.833507 0.705501 0.151535

C -1.649058 0.526266 0.749599

H -1.344095 0.979263 1.691069

C -3.133825 0.844105 -2.400561

H -4.213760 1.006371 -2.522491

H -2.655198 1.827095 -2.331272

H -2.760481 0.331009 -3.290680

O -3.807897 1.633525 0.556398

C -5.122974 1.276994 0.484386

O -5.513890 0.194667 0.111102

C -6.000204 2.422759 0.929821

H -5.739247 2.722766 1.949960

H -5.839481 3.292300 0.283664

H -7.045994 2.116768 0.886576

P 1.467092 0.225084 0.133875

C 1.449255 0.734432 1.912114

C 1.325174 2.084539 2.274041

C 1.451141 -0.235164 2.930986

C 1.211148 2.455291 3.616077

H 1.317954 2.852602 1.507936

C 1.342145 0.137497 4.271015

H 1.556624 -1.286827 2.683276

C 1.220071 1.484842 4.618145

H 1.117873 3.506656 3.874528

H 1.353560 -0.627111 5.043072

H 1.133783 1.774548 5.661787

C 2.954841 -0.860184 -0.021083

C 3.124472 -1.588626 -1.211559

C 3.951489 -0.926314 0.967121

C 4.264777 -2.371173 -1.398597

H 2.367785 -1.533613 -1.989528

C 5.087583 -1.715284 0.773595

H 3.850270 -0.363929 1.888990

C 5.246092 -2.440833 -0.407833

H 4.381238 -2.929025 -2.323722

H 5.848336 -1.757898 1.548650

H 6.130437 -3.054965 -0.555992

C 1.973068 1.763645 -0.752646

C 3.291930 2.241020 -0.686139

C 1.025441 2.484089 -1.493536

C 3.649716 3.419003 -1.340946

H 4.043513 1.688473 -0.130433

C 1.385585 3.663771 -2.148104

H 0.007585 2.115529 -1.575448

C 2.697155 4.133044 -2.072414

H 4.674716 3.775559 -1.284921

H 0.642309 4.206258 -2.725627

H 2.979462 5.047567 -2.587037

H -2.373486 -1.646684 1.694081

H -0.048999 -1.991676 1.438189

C -2.276238 -3.775718 1.799804

H -1.585470 -3.823525 2.650836

H -3.296204 -3.850849 2.196496

C -0.033466 -3.331064 -0.258354

H -0.607447 -3.560595 -1.161063

H 0.984259 -3.088419 -0.570415

H 0.023162 -4.247183 0.350703

H -2.100129 -4.656553 1.172221

**14f**

Atom X Y Z

C 2.897282 -2.456464 0.596998

C 2.767006 -1.178744 1.454424

Rh 0.703578 -0.648139 -0.863144

C 3.472532 -2.195269 -0.804533

C 2.726275 -1.228129 -1.714239

C 2.579281 0.169287 -1.603435

Cl -0.400395 -2.029682 -2.463182

H 4.517736 -1.859235 -0.716252

H 3.511669 -3.155686 -1.331157

H 2.663593 -1.580897 -2.742180

C 2.598519 1.056340 -2.827176

H 3.593595 1.511245 -2.930355

H 1.864702 1.863451 -2.767023

H 2.405459 0.464922 -3.726260

C 1.921578 -0.068446 0.822381

H 1.421793 0.529258 1.583945

C 2.205702 0.725437 -0.324667

O 1.908743 2.103616 -0.284012

C 2.874224 2.924292 0.238069

O 3.965492 2.537770 0.580113

C 2.367668 4.343563 0.307637

H 2.080521 4.690876 -0.690408

H 3.148376 4.987162 0.714535

H 1.473711 4.387322 0.938552

P -1.364973 -0.045129 0.112189

H 1.875432 -2.830719 0.451248

H 2.210997 -1.481778 2.353818

C -2.579061 0.591638 -1.126512

C -2.091769 1.138266 -2.323138

C -3.962856 0.580823 -0.896421

C -2.970449 1.678836 -3.263545

H -1.024762 1.115277 -2.523273

C -4.839764 1.114238 -1.841687

H -4.361749 0.145035 0.014102

C -4.345539 1.667299 -3.025033

H -2.579554 2.092304 -4.189344

H -5.910145 1.092412 -1.654630

H -5.030595 2.078540 -3.761759

C -1.289339 1.325109 1.358112

C -0.835688 1.067952 2.664377

C -1.591779 2.648079 1.003775

C -0.695747 2.103232 3.589421

H -0.607746 0.049972 2.968387

C -1.449698 3.684445 1.930329

H -1.947526 2.872802 0.003603

C -1.001403 3.416737 3.224912

H -0.351584 1.881651 4.596192

H -1.701733 4.701056 1.639341

H -0.896969 4.222601 3.946378

C -2.265710 -1.381820 1.020811

C -2.042167 -2.718553 0.652051

C -3.182633 -1.106566 2.050589

C -2.721492 -3.752480 1.299846

H -1.352445 -2.944643 -0.154968

C -3.857738 -2.143743 2.695888

H -3.365686 -0.082075 2.359530

C -3.627537 -3.469534 2.322814

H -2.538764 -4.781027 1.000659

H -4.561969 -1.913716 3.491191

H -4.151843 -4.276621 2.827712

C 3.694651 -3.561959 1.306310

H 3.313859 -3.739379 2.319656

H 3.617625 -4.505081 0.752770

C 4.111799 -0.591265 1.933136

H 4.747567 -0.302687 1.090225

H 3.948960 0.312943 2.528546

H 4.657355 -1.309574 2.554579

H 4.759641 -3.314624 1.387605

**TS2f***-endo-re*

Imaginary frequency: -342.7862 cm-1

Atom X Y Z

C -1.734901 -2.877909 -1.056937

C -2.574190 -1.743375 -1.400940

Rh -0.355429 -1.447057 -0.298161

C -0.844814 -1.643811 1.699724

C -2.245940 -1.372324 2.051744

C -3.026194 -0.338787 1.615966

Cl 1.324725 -3.215881 -0.282674

H -0.532775 -2.648576 1.996508

H -0.171739 -0.914336 2.160139

C -4.322765 0.009011 2.316146

H -5.171489 0.040070 1.622413

H -4.258843 0.998240 2.784761

H -4.544091 -0.720050 3.101213

C -1.880920 -0.078774 -0.598289

H -1.744067 0.612455 -1.428071

C -2.670299 0.394656 0.424543

O -3.269761 1.667171 0.376733

C -3.887239 2.152039 -0.744133

O -4.093265 1.512911 -1.748750

C -4.270594 3.595686 -0.529798

H -4.820189 3.712668 0.409268

H -4.877383 3.938388 -1.368621

H -3.361033 4.201779 -0.457575

P 1.399612 0.273466 0.007785

H -1.083857 -3.220506 -1.866272

H -2.397376 -1.349491 -2.401321

C 0.787774 2.018165 -0.059644

C 0.019567 2.511696 1.010166

C 0.974998 2.839681 -1.182310

C -0.516936 3.798487 0.971370

H -0.147649 1.893466 1.887728

C 0.427334 4.124433 -1.225496

H 1.561707 2.483777 -2.023060

C -0.313184 4.611232 -0.147213

H -1.094769 4.164360 1.815917

H 0.591862 4.747833 -2.100491

H -0.723370 5.617570 -0.174888

C 2.667136 0.223991 -1.334711

C 3.899767 0.887301 -1.225060

C 2.379613 -0.478760 -2.513562

C 4.816150 0.855793 -2.276061

H 4.149172 1.422469 -0.313651

C 3.294647 -0.505581 -3.568041

H 1.449045 -1.032512 -2.598250

C 4.513960 0.162159 -3.450841

H 5.768681 1.369422 -2.175588

H 3.059109 -1.061465 -4.471374

H 5.230861 0.135556 -4.267192

C 2.403024 0.243822 1.563545

C 2.724662 -1.007559 2.117504

C 2.878883 1.407697 2.191540

C 3.505637 -1.086156 3.272166

H 2.376319 -1.915483 1.632642

C 3.656807 1.321941 3.347598

H 2.641062 2.384730 1.783449

C 3.971214 0.074990 3.891330

H 3.748037 -2.061191 3.686422

H 4.017522 2.231590 3.820954

H 4.576240 0.009872 4.791966

C -2.226605 -4.028457 -0.203644

H -2.839415 -4.722419 -0.799387

H -1.369514 -4.590578 0.179505

C -4.050055 -1.786013 -1.059655

H -4.223421 -1.986309 -0.000295

H -4.542510 -0.853380 -1.339263

H -2.692997 -2.041062 2.791206

H -4.498589 -2.615195 -1.625182

H -2.829740 -3.698459 0.646446

**TS2f***-exo-re*

Imaginary frequency: -322.6854 cm-1

Atom X Y Z

C -1.593729 -3.131261 -0.308327

C -2.543011 -2.098833 -0.713329

Rh -0.313790 -1.513469 0.093722

C -0.845567 -1.406576 2.098065

C -2.258014 -1.146204 2.399437

C -3.059881 -0.205099 1.811466

Cl 1.520912 -3.027582 0.627327

H -0.478554 -2.318826 2.572345

H -0.207417 -0.566142 2.389330

H -2.691852 -1.717806 3.222966

C -4.375284 0.210755 2.433844

H -5.213903 0.084902 1.738832

H -4.351181 1.268230 2.724442

H -4.580313 -0.376971 3.333279

C -1.919728 -0.299469 -0.410708

H -1.831277 0.224280 -1.363165

C -2.707682 0.332612 0.525327

O -3.318554 1.566034 0.204981

C -4.361720 1.587269 -0.675560

O -4.873650 0.595558 -1.141337

C -4.772769 3.010543 -0.963132

H -5.024033 3.527973 -0.031555

H -5.631722 3.012950 -1.634863

H -3.935864 3.548514 -1.420952

P 1.358868 0.376523 -0.026011

C 0.685875 1.959805 -0.708924

C -0.222258 2.709123 0.059883

C 0.965308 2.388486 -2.016699

C -0.807354 3.867534 -0.452228

H -0.467035 2.394752 1.070039

C 0.368074 3.540791 -2.533259

H 1.660856 1.828662 -2.633281

C -0.514382 4.288110 -1.751671

H -1.495118 4.437714 0.166417

H 0.603935 3.858384 -3.545632

H -0.967835 5.191866 -2.150476

C 2.772464 0.015172 -1.158767

C 3.958040 0.766439 -1.126408

C 2.648943 -1.015331 -2.101499

C 4.990864 0.498113 -2.024543

H 4.077822 1.558113 -0.392700

C 3.680513 -1.279272 -3.005015

H 1.757712 -1.635477 -2.110376

C 4.852533 -0.523270 -2.968016

H 5.905264 1.084237 -1.984762

H 3.572513 -2.086924 -3.723789

H 5.659592 -0.734202 -3.664657

C 2.188108 0.894055 1.545666

C 2.574318 -0.119331 2.441253

C 2.469752 2.231565 1.869876

C 3.227746 0.205057 3.631432

H 2.376429 -1.159012 2.193186

C 3.119442 2.549053 3.064501

H 2.183251 3.029911 1.193155

C 3.498907 1.537420 3.948466

H 3.523259 -0.589433 4.311525

H 3.330782 3.588927 3.300463

H 4.004112 1.786697 4.877982

H -3.370317 -2.007311 -0.015884

H -1.855250 -3.643545 0.618505

C -2.971804 -2.003964 -2.169694

H -3.467546 -2.943565 -2.449606

H -3.676888 -1.184348 -2.317590

C -0.960168 -4.045688 -1.340863

H -0.186132 -4.659577 -0.877781

H -1.717469 -4.708096 -1.790559

H -0.492230 -3.492786 -2.165450

H -2.119144 -1.873306 -2.843867

**TS3f***-endo-re*

Imaginary frequency: -231.8165 cm-1

Atom X Y Z

C 0.106683 -2.642383 -0.923341

C 1.240746 -3.312239 -0.254693

C 2.477870 -2.244952 0.838368

C 2.347114 -1.043301 1.717956

C 2.605093 0.254136 1.322159

Rh 0.517526 -1.052142 0.349017

Cl -0.710042 -2.193318 2.198026

H 3.245011 -2.087522 0.083847

H 2.668035 -3.114529 1.463712

H 2.142884 -1.232760 2.768506

C 2.699647 0.539902 -0.126808

C 1.773209 -0.034621 -0.899150

H 1.722801 0.128953 -1.973676

C 2.820600 1.360984 2.320674

H 3.881974 1.642972 2.341862

H 2.253031 2.261457 2.059228

H 2.542731 1.044703 3.330759

O 3.660558 1.466810 -0.590773

C 4.975212 1.128635 -0.464510

O 5.362824 0.081779 0.001483

C 5.856959 2.238601 -0.987706

H 5.633601 2.428381 -2.042796

H 5.659111 3.167109 -0.442025

H 6.904027 1.955546 -0.874448

P -1.256210 0.337927 -0.084741

H -0.855727 -3.006104 -0.562651

H 0.890383 -3.936741 0.564351

C -0.889003 1.746937 -1.226976

C -0.837363 3.071435 -0.769801

C -0.590139 1.490955 -2.577157

C -0.501100 4.112143 -1.639871

H -1.063116 3.297147 0.266666

C -0.266718 2.531417 -3.446834

H -0.617258 0.472987 -2.953798

C -0.218901 3.847268 -2.979350

H -0.466307 5.131894 -1.265791

H -0.047671 2.313145 -4.488724

H 0.037441 4.658213 -3.655698

C -2.767188 -0.450909 -0.811415

C -3.245499 -1.630746 -0.212933

C -3.474285 0.097762 -1.892888

C -4.399338 -2.246936 -0.697708

H -2.717079 -2.054075 0.637326

C -4.627047 -0.527613 -2.375624

H -3.135556 1.014907 -2.362027

C -5.090715 -1.701738 -1.782232

H -4.757184 -3.156829 -0.223237

H -5.161825 -0.090389 -3.214843

H -5.987357 -2.186786 -2.159119

C -1.925116 1.180830 1.418260

C -3.211886 1.742633 1.418062

C -1.130183 1.298497 2.565966

C -3.687641 2.416695 2.542854

H -3.847052 1.648485 0.542525

C -1.606858 1.977139 3.689101

H -0.150004 0.835399 2.586366

C -2.885009 2.536942 3.679857

H -4.687440 2.842539 2.532204

H -0.983934 2.052531 4.576210

H -3.258790 3.057605 4.557559

C 0.109681 -2.442556 -2.426527

H 0.033078 -3.410763 -2.944944

H -0.758490 -1.849252 -2.732067

C 2.285742 -3.990822 -1.125627

H 2.734263 -3.300465 -1.847096

H 3.090324 -4.425420 -0.523274

H 1.014120 -1.948256 -2.792330

H 1.812686 -4.803477 -1.692004

**TS3f***-exo-re*

Imaginary frequency: -216.3866 cm-1

Atom X Y Z

C -0.270981 2.268240 -1.526814

C -1.569858 2.881872 -1.143256

C -2.492012 2.331154 0.473393

C -2.358767 1.253871 1.518872

C -2.651499 -0.076824 1.338628

Rh -0.512620 1.079719 0.146543

Cl 0.573285 2.599114 1.813399

H -3.429424 2.183775 -0.060760

H -2.435353 3.290211 0.979150

H -2.132759 1.604422 2.522677

C -2.709954 -0.591752 -0.047712

C -1.745863 -0.179013 -0.874987

H -1.661508 -0.519989 -1.906364

C -2.866002 -1.016457 2.494855

H -3.905442 -1.370594 2.505441

H -2.225135 -1.903164 2.420648

H -2.672836 -0.520215 3.450583

O -3.685540 -1.558009 -0.383974

C -4.983251 -1.140434 -0.430818

O -5.335062 -0.001858 -0.223785

C -5.895649 -2.293339 -0.778654

H -5.612524 -2.714996 -1.748754

H -5.795695 -3.091542 -0.035704

H -6.927884 -1.943179 -0.812246

P 1.248966 -0.380721 -0.065687

C 0.954509 -1.609302 -1.414443

C 0.468249 -2.898628 -1.157706

C 1.092452 -1.196048 -2.751425

C 0.146724 -3.760058 -2.210080

H 0.336617 -3.236231 -0.135280

C 0.773216 -2.057484 -3.800626

H 1.459360 -0.198194 -2.975522

C 0.300018 -3.344705 -3.532599

H -0.226105 -4.756919 -1.990620

H 0.893906 -1.722111 -4.827281

H 0.050657 -4.016052 -4.349779

C 2.966532 0.229317 -0.412609

C 3.420031 1.389532 0.236419

C 3.861222 -0.490010 -1.223314

C 4.737611 1.819266 0.069046

H 2.740605 1.953730 0.868318

C 5.176251 -0.051612 -1.389313

H 3.539449 -1.392737 -1.731189

C 5.618252 1.104303 -0.744247

H 5.071567 2.719731 0.577553

H 5.853707 -0.618074 -2.023091

H 6.642403 1.444195 -0.874406

C 1.528389 -1.400217 1.453404

C 2.430593 -2.478138 1.452169

C 0.878178 -1.059345 2.646894

C 2.653561 -3.214660 2.614232

H 2.964628 -2.742785 0.544269

C 1.109229 -1.795554 3.812498

H 0.216971 -0.199385 2.669095

C 1.990266 -2.876077 3.797389

H 3.350616 -4.048309 2.598121

H 0.604542 -1.514349 4.732763

H 2.169007 -3.448089 4.704015

H -2.362882 2.521818 -1.796670

H -0.371881 1.608513 -2.392925

C -1.603697 4.391162 -0.958377

H -1.283728 4.885175 -1.884684

H -2.614965 4.740777 -0.725158

C 0.970283 3.135231 -1.629506

H 1.155887 3.704119 -0.716414

H 1.853845 2.521794 -1.826167

H 0.869613 3.843936 -2.467080

H -0.931078 4.706435 -0.155261

**TS4f***-endo-re*

Imaginary frequency: -363.1388 cm-1

Atom X Y Z

C -2.626333 -2.252818 -0.808484

C -2.915168 -0.948245 -1.595434

Rh -0.815611 -1.161265 0.179140

C -2.525595 -1.977680 1.372295

C -3.514532 -0.906361 1.632878

C -3.279512 0.350331 1.226273

Cl 0.295398 -3.260025 0.585550

H -2.895575 -2.994581 1.443636

H -1.683621 -1.913226 2.077634

H -4.428463 -1.146676 2.176253

C -4.180120 1.511726 1.571477

H -4.556081 2.012449 0.671868

H -3.644077 2.267242 2.159733

H -5.032082 1.171064 2.167940

C -2.002120 0.118989 -1.006133

H -1.473315 0.750474 -1.711469

C -2.111808 0.598370 0.319788

O -1.459736 1.836221 0.619909

C -1.823764 2.967762 -0.055238

O -2.637670 3.002039 -0.948720

C -1.103617 4.174040 0.502705

H -1.790007 4.712160 1.167392

H -0.838036 4.844295 -0.317850

H -0.216269 3.891758 1.070867

P 1.265293 -0.056640 -0.054588

H -1.774758 -2.786757 -1.233716

H -2.535719 -1.138944 -2.610375

C 1.832066 0.780649 1.494761

C 0.946006 0.930295 2.570667

C 3.151124 1.248260 1.631734

C 1.363408 1.546337 3.753229

H -0.067812 0.556500 2.478999

C 3.564364 1.868229 2.810779

H 3.860201 1.116240 0.819837

C 2.670290 2.019242 3.874264

H 0.666498 1.648345 4.580865

H 4.587091 2.224289 2.902176

H 2.995745 2.495109 4.795589

C 1.300461 1.260230 -1.357409

C 0.861555 0.918475 -2.649882

C 1.737574 2.571727 -1.125868

C 0.875717 1.856552 -3.682528

H 0.509874 -0.091194 -2.848566

C 1.741036 3.514058 -2.158393

H 2.079925 2.864297 -0.139248

C 1.313597 3.160258 -3.438083

H 0.538057 1.570340 -4.674942

H 2.084382 4.525832 -1.958706

H 1.318028 3.894115 -4.239265

C 2.759664 -1.074921 -0.471336

C 3.257480 -1.955327 0.505247

C 3.409964 -1.007779 -1.711335

C 4.377748 -2.741717 0.244973

H 2.764436 -2.033427 1.467193

C 4.528458 -1.804387 -1.971630

H 3.060614 -0.328725 -2.480785

C 5.015425 -2.672523 -0.995770

H 4.744933 -3.418448 1.011653

H 5.019711 -1.736055 -2.938793

H 5.885883 -3.290949 -1.198459

C -3.777513 -3.236232 -0.681993

H -4.117728 -3.489377 -1.697042

H -3.456967 -4.169004 -0.209515

C -4.384014 -0.512074 -1.756826

H -4.879974 -0.360832 -0.796246

H -4.420798 0.433753 -2.307926

H -4.955000 -1.254553 -2.325224

H -4.639557 -2.837418 -0.138221

**TS4f***-exo-re*

Imaginary frequency: -369.4836 cm-1

Atom X Y Z

C 2.565087 -2.526000 0.527582

C 2.999874 -1.298408 1.370405

Rh 0.818853 -1.181704 -0.350300

C 2.509000 -2.084592 -1.573302

C 3.579596 -1.069316 -1.714649

C 3.415751 0.171654 -1.226994

Cl -0.549246 -2.948801 -1.294422

H 2.803769 -3.119249 -1.734113

H 1.686144 -1.894754 -2.274222

H 4.494360 -1.332248 -2.246214

C 4.406087 1.285545 -1.469071

H 4.781918 1.705627 -0.529239

H 3.945102 2.109867 -2.028292

H 5.254500 0.921577 -2.056703

C 2.106357 -0.143735 0.955180

H 1.641117 0.446626 1.735601

C 2.246328 0.443254 -0.327208

O 1.694333 1.744199 -0.537257

C 2.138611 2.793074 0.220162

O 2.929692 2.696032 1.128858

C 1.544333 4.089720 -0.278253

H 2.218440 4.515924 -1.031316

H 1.468654 4.794367 0.551829

H 0.569497 3.932629 -0.742199

P -1.163626 0.054480 0.031437

C -1.728528 0.960239 -1.477488

C -0.853284 1.104529 -2.563172

C -3.022357 1.502532 -1.571584

C -1.256611 1.786538 -3.713609

H 0.139561 0.671287 -2.505756

C -3.421881 2.186468 -2.719765

H -3.723446 1.379791 -0.751433

C -2.538697 2.330842 -3.793103

H -0.569103 1.883040 -4.549639

H -4.425587 2.599034 -2.778800

H -2.853748 2.857628 -4.690010

C -1.079054 1.352828 1.354663

C -0.615616 0.979493 2.629439

C -1.462775 2.686307 1.152135

C -0.552201 1.905506 3.671268

H -0.305677 -0.046265 2.810307

C -1.390755 3.616004 2.193248

H -1.823171 3.006140 0.180662

C -0.937719 3.229928 3.454716

H -0.194184 1.592785 4.648378

H -1.693857 4.644309 2.013990

H -0.882139 3.954340 4.262541

C -2.695323 -0.885467 0.507361

C -3.385683 -1.604550 -0.484611

C -3.182392 -0.922735 1.822280

C -4.528602 -2.335758 -0.165030

H -3.026686 -1.599978 -1.506082

C -4.323757 -1.663062 2.140063

H -2.690293 -0.363446 2.609247

C -5.000391 -2.372001 1.148504

H -5.045265 -2.885293 -0.947161

H -4.684515 -1.674316 3.165312

H -5.889646 -2.945965 1.395551

H 4.035527 -1.059121 1.095323

H 3.459842 -3.026962 0.164697

C 2.973203 -1.546362 2.885684

H 3.622787 -2.383851 3.163627

H 3.331462 -0.654699 3.412629

C 1.660096 -3.584988 1.142224

H 0.753443 -3.183919 1.600533

H 1.348401 -4.305284 0.382875

H 2.228819 -4.126441 1.915203

H 1.962777 -1.770878 3.244722

**TS5f***-endo-re*

Imaginary frequency: -329.4911 cm-1

Atom X Y Z

C -1.202751 -2.010829 0.724615

C -1.766155 -2.933514 -0.386517

C -2.951911 -2.266602 -1.100906

C -2.574382 -0.960360 -1.775897

C -2.822410 0.302328 -1.228186

Rh -0.595718 -0.313273 -0.828162

Cl 0.188206 0.345092 -3.004327

H -3.767136 -2.092515 -0.387675

H -3.337560 -2.952038 -1.866035

H -2.450082 -0.992702 -2.855836

C -2.814043 0.454134 0.226289

C -1.725480 -0.096051 0.837768

H -1.479120 0.143451 1.866402

C -3.130950 1.497011 -2.098209

H -4.215050 1.554760 -2.263883

H -2.803578 2.435389 -1.639008

H -2.636680 1.391459 -3.067167

O -3.626417 1.387594 0.878773

C -4.984196 1.287128 0.713459

O -5.520066 0.414585 0.073236

C -5.680877 2.414697 1.434706

H -5.363491 2.451192 2.481510

H -5.408291 3.372209 0.977467

H -6.760237 2.272310 1.372711

P 1.507124 0.176586 0.076799

H -0.122384 -2.153310 0.803191

H -0.971023 -3.041348 -1.133976

C 1.555086 0.169318 1.931300

C 1.313138 1.349393 2.652805

C 1.713351 -1.030776 2.646142

C 1.237692 1.331104 4.046982

H 1.189871 2.289648 2.124486

C 1.641871 -1.047009 4.040465

H 1.914249 -1.957019 2.115707

C 1.402502 0.134081 4.745893

H 1.055657 2.257264 4.585724

H 1.777671 -1.984129 4.573991

H 1.348757 0.121626 5.831095

C 2.891028 -0.954056 -0.396546

C 2.799330 -1.650167 -1.613252

C 4.038152 -1.123999 0.399058

C 3.834070 -2.494433 -2.021725

H 1.927324 -1.509676 -2.244308

C 5.068054 -1.970741 -0.013174

H 4.126460 -0.605371 1.348726

C 4.967657 -2.658439 -1.224494

H 3.750045 -3.023282 -2.967268

H 5.947351 -2.093018 0.613911

H 5.769656 -3.318877 -1.543726

C 2.103086 1.875951 -0.337206

C 3.455578 2.236214 -0.242558

C 1.164838 2.845901 -0.719600

C 3.858802 3.543608 -0.515657

H 4.200601 1.496112 0.031004

C 1.569753 4.155482 -0.984172

H 0.123277 2.562617 -0.832598

C 2.916491 4.506722 -0.883210

H 4.910768 3.807549 -0.445846

H 0.832915 4.894977 -1.285929

H 3.232987 5.523803 -1.099122

C -1.782415 -2.284481 2.123372

H -2.865287 -2.128925 2.170374

H -1.309473 -1.668351 2.891891

C -2.128242 -4.353441 0.091063

H -2.987289 -4.356876 0.771096

H -2.390902 -4.969990 -0.776658

H -1.578596 -3.329445 2.387493

H -1.288617 -4.842033 0.599413

**TS5f***-exo-re*

Imaginary frequency: -346.3673 cm-1

Atom X Y Z

C -1.365677 -1.791444 1.661609

C -1.914780 -3.077341 1.003158

C -3.068759 -2.684645 0.069221

C -2.636836 -1.713824 -1.022256

C -2.858004 -0.323683 -0.973883

Rh -0.682009 -0.780577 -0.424037

Cl 0.114188 -1.228896 -2.663044

H -3.877576 -2.232059 0.655314

H -3.482798 -3.584309 -0.402494

H -2.540144 -2.129831 -2.022140

C -2.833119 0.360255 0.318765

C -1.758049 0.033961 1.097396

H -1.497879 0.597154 1.990288

C -3.181219 0.457606 -2.225787

H -4.265933 0.440196 -2.396586

H -2.863803 1.503239 -2.153749

H -2.688613 -0.000516 -3.087523

O -3.616042 1.489917 0.577660

C -4.978354 1.369528 0.459572

O -5.538920 0.335391 0.187317

C -5.642044 2.700323 0.713698

H -5.335780 3.421118 -0.052221

H -6.725050 2.575428 0.689760

H -5.329570 3.101796 1.682841

P 1.409778 0.256628 0.028995

C 1.499130 1.284840 1.577131

C 0.951967 2.579370 1.567515

C 1.998832 0.785948 2.791859

C 0.908569 3.348752 2.730802

H 0.564836 2.995919 0.642861

C 1.960085 1.558910 3.955132

H 2.433516 -0.207066 2.834177

C 1.413264 2.842239 3.930047

H 0.484625 4.348883 2.695560

H 2.361495 1.153717 4.880406

H 1.384118 3.443375 4.834788

C 2.907829 -0.824936 0.108936

C 2.848579 -2.080676 -0.517423

C 4.108346 -0.434969 0.729656

C 3.960644 -2.925381 -0.516325

H 1.939771 -2.378414 -1.030335

C 5.216510 -1.283423 0.730544

H 4.181164 0.529988 1.221483

C 5.144280 -2.532060 0.109279

H 3.898533 -3.891120 -1.010550

H 6.135563 -0.966653 1.216696

H 6.007602 -3.192278 0.111107

C 1.813036 1.521659 -1.265447

C 3.128417 1.926346 -1.533506

C 0.761960 2.131729 -1.966397

C 3.384894 2.925581 -2.473574

H 3.961082 1.455312 -1.022357

C 1.019045 3.138881 -2.897522

H -0.256542 1.796863 -1.797869

C 2.331420 3.538059 -3.154250

H 4.411057 3.221449 -2.675485

H 0.193092 3.597159 -3.435014

H 2.533166 4.314496 -3.887476

H -2.335689 -3.665483 1.837918

H -2.140109 -1.504707 2.378795

C -0.887928 -3.975449 0.301217

H -1.379248 -4.895473 -0.039637

H -0.448999 -3.478751 -0.570653

C -0.082830 -1.947040 2.465911

H 0.773869 -2.258919 1.865344

H 0.174106 -1.018303 2.980513

H -0.246969 -2.714132 3.238663

H -0.075892 -4.267459 0.975795

**10g**

Atom X Y Z

C 1.595215 -3.101738 1.358929

C 2.335679 -2.162114 1.624338

Rh 0.443893 -1.284504 0.467301

C 0.890585 -1.665285 -1.534819

C 2.331058 -1.900791 -1.736465

C 3.308305 -1.041762 -1.346146

Cl -1.355040 -2.996755 0.757072

H 0.269337 -2.524338 -1.795872

H 0.547233 -0.778348 -2.074602

H 2.623366 -2.827664 -2.230571

C 4.753022 -1.247640 -1.726628

H 5.390560 -1.354246 -0.839300

H 5.137802 -0.392655 -2.296193

H 4.869205 -2.144920 -2.341430

C 1.846475 0.161805 0.291649

H 1.735728 1.076626 0.877336

C 2.960874 0.067434 -0.464355

O 3.950913 1.092736 -0.373536

C 4.744350 1.084213 0.728368

O 4.684546 0.256509 1.613662

C 5.711004 2.244418 0.693723

H 6.360925 2.205270 1.568464

H 5.157110 3.189085 0.682725

H 6.311113 2.208440 -0.221262

P -1.252271 0.256632 -0.035491

H 1.043047 -4.011993 1.258201

H 3.110931 -1.455730 1.854233

C -0.672837 2.008503 -0.139889

C 0.129827 2.407015 -1.224480

C -0.944757 2.938812 0.873735

C 0.628635 3.706618 -1.300096

H 0.362151 1.702665 -2.017119

C -0.435276 4.238156 0.800389

H -1.559270 2.655208 1.721447

C 0.348595 4.626141 -0.286039

H 1.241354 3.997745 -2.148834

H -0.659669 4.946566 1.593306

H 0.740044 5.638239 -0.343771

C -2.512771 0.301314 1.307350

C -3.824217 0.738545 1.070522

C -2.142056 -0.072018 2.607794

C -4.744510 0.804464 2.117680

H -4.132341 1.019714 0.068302

C -3.061505 0.001686 3.654266

H -1.140002 -0.443650 2.802141

C -4.364920 0.438517 3.410395

H -5.759905 1.137421 1.920268

H -2.762700 -0.298813 4.654592

H -5.084366 0.486027 4.223432

C -2.223080 0.030329 -1.592523

C -2.670691 1.124614 -2.352601

C -2.564413 -1.269578 -2.003688

C -3.436000 0.921847 -3.502698

H -2.424235 2.137277 -2.052773

C -3.330045 -1.464044 -3.153949

H -2.246690 -2.120768 -1.409642

C -3.764960 -0.372440 -3.907786

H -3.773705 1.778676 -4.079715

H -3.586732 -2.475163 -3.458179

H -4.358948 -0.528731 -4.804440

**11g**

Atom X Y Z

C 0.416944 -2.721028 -2.373831

C -0.617078 -2.029792 -2.342437

Rh 0.540743 -1.507530 -0.584281

C 2.134353 -2.800091 -0.092036

C 2.143418 -1.836755 0.983404

C 2.509565 -0.500237 0.806612

Cl -0.972413 -3.043710 0.715121

H 2.927689 -2.737843 -0.834596

H 1.807191 -3.803799 0.163837

H 1.130721 -3.450932 -2.700105

H -1.606264 -1.693990 -2.586743

H 1.780483 -2.138177 1.962513

C 2.616775 0.433958 1.979657

H 3.673272 0.512620 2.268334

H 2.260186 1.439518 1.735378

H 2.055397 0.059845 2.839224

C 1.941235 -0.374091 -1.492187

H 1.995888 -0.077298 -2.538886

C 2.822384 -0.009154 -0.552195

O 3.855064 0.924551 -0.765259

C 5.105398 0.636420 -0.296712

O 5.372776 -0.354252 0.342727

C 6.075433 1.726135 -0.684843

H 5.733310 2.691991 -0.298748

H 7.062183 1.492668 -0.283703

H 6.127063 1.810634 -1.775428

P -1.095230 0.366554 -0.015605

C -1.015217 2.028110 -0.838752

C 0.202748 2.497376 -1.352830

C -2.142383 2.867314 -0.915816

C 0.292175 3.763979 -1.936652

H 1.085889 1.872486 -1.299286

C -2.051994 4.130828 -1.499367

H -3.094624 2.530647 -0.519314

C -0.834007 4.582589 -2.014013

H 1.244949 4.105452 -2.332556

H -2.934563 4.763013 -1.550773

H -0.765774 5.566380 -2.470746

C -1.061372 0.814970 1.788020

C -1.001838 -0.213013 2.747627

C -1.049967 2.148676 2.227942

C -0.957479 0.092915 4.109302

H -0.992099 -1.250579 2.423309

C -0.992227 2.447307 3.591483

H -1.085465 2.962517 1.512714

C -0.950405 1.422372 4.536847

H -0.924245 -0.715309 4.835444

H -0.982774 3.486268 3.910949

H -0.909786 1.657132 5.597286

C -2.845214 -0.127908 -0.358938

C -3.667790 -0.697585 0.621422

C -3.340724 -0.012069 -1.670766

C -4.955456 -1.131698 0.300459

H -3.308234 -0.808816 1.637309

C -4.627078 -0.449280 -1.989164

H -2.731914 0.449418 -2.444115

C -5.438840 -1.012651 -1.002505

H -5.578504 -1.570029 1.075224

H -4.994240 -0.343756 -3.006727

H -6.440578 -1.354417 -1.248268

**12g**

Atom X Y Z

C 0.881140 0.644666 -1.629310

C 2.044377 1.207870 -2.037499

Rh 0.569436 -0.528849 -0.109051

C 1.494694 0.919996 1.159320

C 2.352618 -0.225749 1.261545

C 3.353130 -0.624652 0.382383

Cl 0.249918 -2.750094 -0.984424

H 1.844076 1.783491 0.598778

H 0.924386 1.182956 2.048877

H 2.139640 -0.931911 2.067324

C 4.039715 -1.953141 0.627740

H 4.943409 -1.823917 1.237561

H 4.332295 -2.429682 -0.312309

H 3.369812 -2.634089 1.159390

C 3.400237 1.061581 -1.561578

H 4.141981 1.586847 -2.159564

C 3.945856 0.243480 -0.625210

O 5.355435 0.110408 -0.745590

C 6.140457 0.514956 0.297678

O 5.716246 0.990914 1.323210

C 7.595627 0.273750 -0.029679

H 7.770434 -0.795210 -0.191852

H 8.216612 0.630682 0.792617

H 7.865542 0.790953 -0.956029

P -1.614647 0.109899 0.049085

H 0.022769 0.746745 -2.296519

H 1.979334 1.778587 -2.966830

C -2.426368 -1.048370 1.225390

C -3.503786 -1.864729 0.857410

C -1.895420 -1.157156 2.523058

C -4.042058 -2.771516 1.773135

H -3.918709 -1.799349 -0.142748

C -2.438095 -2.060894 3.434892

H -1.060925 -0.528452 2.824567

C -3.512114 -2.872310 3.059862

H -4.876078 -3.401269 1.475972

H -2.019719 -2.134664 4.434927

H -3.931567 -3.581576 3.768020

C -1.935780 1.782927 0.759308

C -1.140288 2.866728 0.353157

C -2.986354 2.010429 1.662510

C -1.393987 4.149751 0.838366

H -0.317068 2.703020 -0.335892

C -3.232320 3.295335 2.151663

H -3.613711 1.186175 1.986652

C -2.437912 4.366714 1.740840

H -0.768946 4.978497 0.517206

H -4.045917 3.455744 2.853950

H -2.628577 5.365356 2.124385

C -2.628184 0.082967 -1.490493

C -3.621962 1.045975 -1.733085

C -2.400404 -0.925772 -2.443207

C -4.379793 0.995640 -2.903731

H -3.806419 1.835889 -1.012993

C -3.166572 -0.970663 -3.609395

H -1.628905 -1.670579 -2.267956

C -4.154705 -0.012355 -3.843196

H -5.144669 1.747129 -3.080403

H -2.982024 -1.755485 -4.337756

H -4.744515 -0.047893 -4.755361

**13g**

Atom X Y Z

C -0.046328 -2.077304 1.645644

Rh -0.616651 -1.136750 -0.009425

C -0.930193 -2.831550 2.302373

C -2.365875 -2.890993 1.832685

C -2.485292 -2.632290 0.341794

C -2.996791 -1.510117 -0.280810

Cl 0.170898 -2.605724 -1.815081

H -2.973051 -2.165664 2.390006

H -2.789620 -3.884804 2.033837

H 0.999751 -2.010920 1.943295

H -0.640120 -3.406862 3.183544

H -2.326978 -3.503311 -0.291790

C -3.507343 -1.560811 -1.703240

H -3.146561 -2.455714 -2.216428

H -4.605670 -1.582564 -1.694938

H -3.199174 -0.681029 -2.277888

C -1.833801 0.167042 0.920360

H -1.640592 1.085040 1.467080

C -3.028848 -0.217225 0.445718

O -4.256456 0.463910 0.474805

C -4.322940 1.753668 0.014529

O -3.393769 2.362982 -0.457994

C -5.727047 2.282865 0.182211

H -5.783406 3.297248 -0.213947

H -6.439398 1.635799 -0.339755

H -6.002016 2.280761 1.242286

P 1.213742 0.253860 0.002259

C 1.181910 1.594379 1.271587

C 0.888177 1.259699 2.604432

C 1.475414 2.928935 0.954712

C 0.894890 2.239515 3.596751

H 0.640252 0.234783 2.863589

C 1.476960 3.908492 1.951202

H 1.701315 3.207687 -0.069244

C 1.188428 3.566610 3.272578

H 0.663126 1.966303 4.622649

H 1.703345 4.938712 1.690016

H 1.187980 4.329923 4.045997

C 1.232003 1.134532 -1.610051

C 2.336901 1.092750 -2.470631

C 0.071858 1.826178 -2.004845

C 2.285925 1.738497 -3.707747

H 3.232376 0.553160 -2.180547

C 0.031321 2.473638 -3.239237

H -0.799396 1.865900 -1.354808

C 1.136325 2.428970 -4.093413

H 3.146383 1.697213 -4.369995

H -0.868068 3.007458 -3.533305

H 1.099254 2.928084 -5.058002

C 2.885637 -0.489823 0.226194

C 3.932833 0.255947 0.795399

C 3.127219 -1.810568 -0.185976

C 5.199282 -0.309253 0.945073

H 3.762264 1.275718 1.124682

C 4.398275 -2.368349 -0.031773

H 2.326696 -2.389705 -0.636594

C 5.434044 -1.623101 0.533472

H 6.000162 0.277233 1.387387

H 4.572815 -3.391165 -0.354150

H 6.420353 -2.063255 0.655209

**14g**

Atom X Y Z

C -1.178124 1.301227 -1.529709

C -1.428639 1.659823 -0.180367

Rh -0.706694 -0.388159 -0.315440

C -2.297056 0.690129 -2.356610

C -2.564339 -0.641447 -1.674230

C -3.160587 -0.734687 -0.432286

Cl -0.316530 -2.705778 0.103063

H -3.199383 1.321028 -2.369434

H -1.974308 0.527770 -3.388427

H -2.384912 -1.561665 -2.224476

C -3.661338 -2.057164 0.101841

H -4.731408 -2.166247 -0.119027

H -3.525219 -2.133061 1.184747

H -3.123288 -2.883875 -0.364375

C -2.756113 1.570177 0.448162

H -3.096442 2.389652 1.077803

C -3.542276 0.486747 0.314379

O -4.734735 0.442968 1.051166

C -5.910440 0.372684 0.350329

O -5.971343 0.396355 -0.855013

C -7.077103 0.263488 1.300701

H -7.006393 -0.669314 1.870485

H -8.008750 0.280623 0.734402

H -7.058154 1.087410 2.021027

P 1.519749 0.024912 0.109021

H -0.316459 1.731842 -2.035134

H -0.746561 2.367723 0.281923

C 2.659233 -1.030599 -0.890972

C 3.965447 -1.330369 -0.478319

C 2.207082 -1.516071 -2.127568

C 4.804915 -2.092686 -1.292216

H 4.328635 -0.985059 0.484141

C 3.052076 -2.268678 -2.944106

H 1.182545 -1.323370 -2.431511

C 4.352540 -2.559033 -2.527867

H 5.811802 -2.325628 -0.955995

H 2.686538 -2.642680 -3.896658

H 5.007232 -3.154609 -3.158577

C 2.068195 1.745134 -0.310461

C 1.804319 2.806313 0.573949

C 2.662895 2.036518 -1.547782

C 2.135110 4.118260 0.233199

H 1.356490 2.604527 1.543100

C 2.989620 3.351363 -1.888979

H 2.882559 1.234144 -2.244947

C 2.727730 4.395230 -1.000992

H 1.931453 4.923290 0.934317

H 3.455269 3.555563 -2.849460

H 2.985588 5.416912 -1.266206

C 2.071956 -0.168685 1.862780

C 3.212214 0.489127 2.358451

C 1.345342 -1.007731 2.722228

C 3.616215 0.308466 3.681788

H 3.781605 1.153655 1.715974

C 1.754272 -1.184925 4.045733

H 0.474348 -1.533830 2.346343

C 2.886897 -0.528700 4.528946

H 4.498964 0.824824 4.049760

H 1.182194 -1.839272 4.697880

H 3.200429 -0.666698 5.560398

**TS2g**

Imaginary frequency: -328.443 cm-1

Atom X Y Z

C -1.680915 -2.513498 -1.727662

C -2.208257 -1.380457 -1.941354

Rh -0.400328 -1.323172 -0.594398

C -0.861357 -1.826706 1.377347

C -2.262926 -2.241732 1.424434

C -3.309842 -1.407788 1.139970

Cl 1.281362 -3.088795 -0.847467

H -0.154370 -2.614434 1.635675

H -0.674485 -0.922912 1.960730

H -2.482310 -3.261694 1.741051

C -4.739101 -1.765798 1.465433

H -5.372496 -1.755651 0.569145

H -5.173325 -1.049059 2.172960

H -4.796148 -2.760906 1.916132

C -1.963648 -0.002775 -0.421885

H -1.900900 0.970890 -0.905399

C -3.035789 -0.196627 0.411942

O -4.014090 0.813457 0.559541

C -4.677388 1.281732 -0.538866

O -4.542982 0.844309 -1.658540

C -5.594408 2.414352 -0.144906

H -6.249607 2.107740 0.676346

H -6.188404 2.718038 -1.007466

H -4.999088 3.262285 0.211170

P 1.280367 0.252569 0.060016

H -1.567097 -3.552888 -1.979082

H -2.813339 -0.693573 -2.507081

C 0.598061 1.964688 0.211101

C -0.252483 2.277912 1.287540

C 0.800343 2.932362 -0.784545

C -0.861339 3.529741 1.375782

H -0.434173 1.545034 2.068249

C 0.181586 4.182461 -0.699709

H 1.446927 2.714643 -1.628157

C -0.646813 4.486829 0.380801

H -1.505339 3.754644 2.221841

H 0.355294 4.919556 -1.479228

H -1.121662 5.461978 0.448517

C 2.616605 0.432142 -1.200402

C 3.799710 1.131521 -0.911396

C 2.443400 -0.113439 -2.480233

C 4.782464 1.290938 -1.887744

H 3.957866 1.545818 0.079949

C 3.427993 0.049804 -3.457352

H 1.553636 -0.692082 -2.705628

C 4.597070 0.751993 -3.163662

H 5.695210 1.830927 -1.650198

H 3.283893 -0.386153 -4.442043

H 5.365903 0.872569 -3.922293

C 2.207169 -0.000754 1.640073

C 2.621545 -1.306567 1.956400

C 2.548281 1.051257 2.505739

C 3.358639 -1.547758 3.116401

H 2.375911 -2.125550 1.285611

C 3.283477 0.801909 3.666717

H 2.241137 2.067081 2.279618

C 3.688889 -0.497336 3.975357

H 3.674236 -2.561720 3.347124

H 3.539171 1.626181 4.327536

H 4.260444 -0.689625 4.879603

**TS3g**

Imaginary frequency: -259.664 cm-1

Atom X Y Z

C 0.965958 -2.828381 -2.062830

C 2.321455 -2.686511 -0.437153

C 2.323076 -1.954904 0.841489

C 2.642557 -0.612296 0.989395

C -0.033867 -2.047501 -2.159849

Rh 0.503775 -1.294679 -0.340702

Cl -0.776959 -3.029030 0.928513

H 3.098574 -2.372170 -1.130218

H 2.230279 -3.758843 -0.291393

H -0.875998 -1.818724 -2.794552

H 2.084368 -2.531588 1.731010

C 2.771810 0.222162 -0.227883

C 1.801835 0.064839 -1.130118

H 1.742841 0.616083 -2.065206

C 2.915171 -0.014257 2.343387

H 3.994747 0.149771 2.462424

H 2.423720 0.957248 2.464431

H 2.591270 -0.681948 3.147287

O 3.800625 1.184344 -0.314834

C 5.088744 0.735076 -0.313271

O 5.396309 -0.432358 -0.238935

C 6.050700 1.895003 -0.418028

H 5.879084 2.438660 -1.353072

H 5.884452 2.600316 0.402751

H 7.074961 1.521802 -0.388798

H 1.512948 -3.658347 -2.471810

P -1.170037 0.273660 -0.022136

C -0.702247 1.988178 -0.532054

C -0.456213 2.999105 0.407306

C -0.506282 2.273168 -1.895028

C -0.032624 4.265319 -0.005896

H -0.599913 2.803926 1.464799

C -0.094508 3.539638 -2.306554

H -0.685757 1.502687 -2.640126

C 0.145929 4.540385 -1.361188

H 0.152011 5.036944 0.736784

H 0.042532 3.743580 -3.365186

H 0.470712 5.526756 -1.681130

C -2.733496 -0.052551 -0.959717

C -3.202099 -1.375719 -1.038014

C -3.490751 0.974661 -1.546535

C -4.402152 -1.657399 -1.693114

H -2.632432 -2.176124 -0.572943

C -4.688824 0.684330 -2.203279

H -3.149457 2.003420 -1.498043

C -5.146759 -0.631688 -2.279159

H -4.752905 -2.684737 -1.743215

H -5.262335 1.490210 -2.653894

H -6.078895 -0.856069 -2.791105

C -1.754116 0.486655 1.716875

C -2.932234 1.196659 2.000564

C -1.004980 -0.041863 2.775653

C -3.342326 1.384489 3.319994

H -3.535492 1.597675 1.191621

C -1.416702 0.149115 4.096523

H -0.120007 -0.629058 2.560180

C -2.583497 0.862639 4.371006

H -4.257718 1.932613 3.526510

H -0.832151 -0.275465 4.908127

H -2.907272 1.004550 5.398713

**TS4g**

Imaginary frequency: -431.4931 cm-1

Atom X Y Z

C -1.053149 1.738732 0.249502

C -2.105715 2.136968 1.018757

Rh -0.727353 -0.262493 -0.078577

C -1.651687 1.236607 -1.565654

C -2.266371 -0.088436 -1.549297

C -3.134886 -0.502697 -0.529798

Cl -0.546948 -2.344402 0.998167

H -2.341385 2.070752 -1.483767

H -0.901249 1.404163 -2.334451

H -1.989514 -0.808749 -2.316297

C -3.646517 -1.930198 -0.578069

H -4.633552 -1.977589 -1.055089

H -3.725641 -2.359822 0.424184

H -2.962720 -2.559058 -1.153366

C -3.404336 1.516954 1.062340

H -4.147470 1.984286 1.703305

C -3.827782 0.406824 0.404574

O -5.129901 -0.020655 0.763837

C -6.122483 0.105412 -0.166339

O -5.962165 0.578090 -1.266492

C -7.421608 -0.432796 0.385239

H -7.321331 -1.503088 0.595511

H -8.219062 -0.271900 -0.340998

H -7.666830 0.062308 1.330172

P 1.606686 -0.030758 -0.003636

H -0.169438 2.369698 0.205085

H -1.980540 3.033412 1.625388

C 2.541562 -1.167917 -1.124399

C 2.045338 -2.457507 -1.376585

C 3.758939 -0.781725 -1.713047

C 2.753285 -3.336681 -2.198083

H 1.114879 -2.773992 -0.917626

C 4.461075 -1.664781 -2.534928

H 4.159875 0.211469 -1.538420

C 3.959135 -2.943984 -2.780849

H 2.355999 -4.331202 -2.382253

H 5.399604 -1.349617 -2.983324

H 4.504879 -3.629627 -3.423646

C 2.192580 1.640141 -0.537598

C 2.718206 2.572757 0.367913

C 2.022404 2.024846 -1.880088

C 3.068266 3.857439 -0.058841

H 2.860330 2.297683 1.407874

C 2.377951 3.303877 -2.304970

H 1.623733 1.314280 -2.599580

C 2.899995 4.226178 -1.393042

H 3.475902 4.566686 0.656487

H 2.247367 3.581108 -3.347590

H 3.174047 5.224349 -1.723370

C 2.372334 -0.253271 1.659234

C 3.730054 -0.567432 1.823683

C 1.569313 -0.069759 2.795359

C 4.275398 -0.685762 3.102431

H 4.361279 -0.730984 0.955633

C 2.120133 -0.181818 4.073134

H 0.510430 0.139134 2.676943

C 3.472529 -0.490079 4.228646

H 5.326583 -0.936169 3.218096

H 1.486679 -0.043209 4.944888

H 3.898609 -0.586769 5.223740

**TS5g**

Imaginary frequency: -332.5726 cm-1

Atom X Y Z

C 0.805278 -1.450420 -1.904159

Rh 0.630796 -1.182948 0.107659

C 1.483985 -2.542607 -2.310006

C 2.527450 -3.138385 -1.410490

C 2.365980 -2.692222 0.042188

C 2.893931 -1.495843 0.542800

Cl -0.091108 -1.987164 2.254693

H 3.532239 -2.870790 -1.782069

H 2.473852 -4.234083 -1.442231

H 0.123596 -0.936396 -2.577028

H 1.316129 -2.981870 -3.294229

H 2.148050 -3.470886 0.769101

C 3.376871 -1.357074 1.966779

H 2.914832 -2.113962 2.604324

H 4.467106 -1.492405 1.995093

H 3.145333 -0.370411 2.379056

C 1.892176 -0.077075 -1.055171

H 1.724702 0.796025 -1.675711

C 3.035123 -0.358841 -0.380361

O 4.231701 0.365470 -0.369260

C 4.213495 1.716524 -0.132029

O 3.216539 2.357474 0.097372

C 5.618268 2.263844 -0.200309

H 5.603585 3.332206 0.017317

H 6.261092 1.743182 0.516791

H 6.037249 2.092166 -1.197476

P -1.253893 0.194374 0.009875

C -1.334982 1.277410 -1.493434

C -1.861351 0.761462 -2.691711

C -0.793726 2.572090 -1.502067

C -1.849875 1.521756 -3.861775

H -2.299853 -0.232170 -2.707883

C -0.782786 3.330559 -2.675179

H -0.386159 2.998414 -0.591790

C -1.308752 2.809165 -3.857691

H -2.267679 1.107054 -4.775358

H -0.364640 4.333462 -2.658519

H -1.301169 3.401836 -4.768447

C -1.262224 1.372922 1.428657

C -2.416718 1.660153 2.169346

C -0.051197 1.994104 1.781060

C -2.365124 2.563313 3.233153

H -3.355697 1.172878 1.930945

C -0.009284 2.906945 2.834990

H 0.861793 1.764581 1.238490

C -1.165052 3.192503 3.564975

H -3.266380 2.769583 3.804386

H 0.932815 3.384288 3.090477

H -1.127953 3.894770 4.393582

C -2.905213 -0.623463 -0.007124

C -4.066049 0.092862 -0.352169

C -3.015275 -1.983374 0.318787

C -5.309722 -0.537827 -0.362622

H -3.998491 1.142681 -0.622167

C -4.263321 -2.611254 0.302774

H -2.128682 -2.539595 0.602752

C -5.409981 -1.892853 -0.035700

H -6.198391 0.027921 -0.629688

H -4.334180 -3.664805 0.558858

H -6.379159 -2.384654 -0.047124

**RhClPPh_3_**

Atom X Y Z

Rh -1.577488 0.237567 -0.908632

Cl -1.690052 -0.403916 -3.107010

P 0.356204 0.005250 0.012845

C 1.171732 -1.614050 0.204126

C -0.903412 0.306087 1.269096

C 1.664363 1.278195 0.005310

C 0.519895 -2.750773 -0.301739

C 2.417235 -1.748763 0.839514

C -1.654274 1.504244 1.014158

C -1.395021 -0.652839 2.205654

C 2.094857 1.896631 1.189777

C 2.230370 1.656398 -1.223029

C 1.101969 -4.010188 -0.153381

H -0.428881 -2.634416 -0.818885

C 2.991048 -3.011225 0.985393

H 2.938676 -0.872443 1.212865

C -2.897493 1.676171 1.692890

H -1.183413 2.379018 0.573343

C -2.592046 -0.436839 2.850054

H -0.816279 -1.551250 2.400522

C 3.090888 2.874175 1.144258

H 1.650085 1.619765 2.141773

C 3.228902 2.629066 -1.260132

H 1.877512 1.201115 -2.144724

C 2.334022 -4.141274 0.490954

H 0.596982 -4.885942 -0.550928

H 3.953194 -3.111920 1.480008

C -3.360617 0.724646 2.575218

H -3.462323 2.589254 1.523881

H -2.957041 -1.163366 3.570887

C 3.659546 3.237958 -0.078166

H 3.419195 3.352376 2.063051

H 3.662878 2.919036 -2.212847

H 2.787799 -5.122582 0.600551

H -4.307774 0.871974 3.086774

H 4.432403 4.000965 -0.111065
